# Predictive proteomic signatures for response of pancreatic cancer patients receiving chemotherapy

**DOI:** 10.1186/s12014-019-9251-3

**Published:** 2019-07-17

**Authors:** Hong Peng, Ru Chen, Teresa A. Brentnall, Jimmy K. Eng, Vincent J. Picozzi, Sheng Pan

**Affiliations:** 1 Institute of Molecular Medicine, the University of Texas Health Science Center at Houston, Houston, TX 77030 USA; 2 Division of Gastroenterology, Department of Medicine, Baylor College of Medicine, Houston, TX 77030 USA; 3 Division of Gastroenterology, Department of Medicine, The University of Washington, Seattle, WA 98195 USA; 4 Proteomics Resource, The University of Washington, Seattle, WA 98109 USA; 5 Virginia Mason Medical Center, Seattle, WA 98101 USA

**Keywords:** Proteomics, Pancreatic cancer, Plasma, Biomarker, Mass spectrometry, Chemotherapy

## Abstract

**Background:**

Pancreatic ductal adenocarcinoma (PDAC) is a lethal cancer that is characterized by its poor prognosis, rapid progression and development of drug resistance. Chemotherapy is a vital treatment option for most of PDAC patients. Stratification of PDAC patients, who would have a higher likelihood of responding to chemotherapy, could facilitate treatment selection and patient management.

**Methods:**

A quantitative proteomic study was performed to characterize the protein profiles in the plasma of PDAC patients undergoing chemotherapy to determine if specific biomarkers could be used to predict likelihood of therapeutic response.

**Results:**

By comparing the plasma proteome of the PDAC patients with positive therapeutic response and longer overall survival (Good-responders) to those who did not respond as well with shorter survival time (Limited-responders), we identified differential proteins and protein variants that could effectively segregate Good-responders from Limited-responders. Functional clustering and pathway analysis further suggested that many of these differential proteins were involved in pancreatic tumorigenesis. Four proteins, including vitamin-K dependent protein Z (PZ), sex hormone-binding globulin (SHBG), von Willebrand factor (VWF) and zinc-alpha-2-glycoprotein (AZGP1), were further evaluated as single or composite predictive biomarker with/without inclusion of CA 19-9. A composite biomarker panel that consists of PZ, SHBG, VWF and CA 19-9 demonstrated the best performance in distinguishing Good-responders from Limited-responders.

**Conclusion:**

Based on the cohort investigated, our data suggested that systemic proteome alterations involved in pathways associated with inflammation, immunoresponse, coagulation and complement cascades may be reporters of chemo-treatment outcome in PDAC patients. For the majority of the patients involved, the panel consisting of PZ, SHBG, VWF and CA 19-9 was able to segregate Good-responders from Limited-responders effectively. Our data also showed that dramatic fluctuations of biomarker concentration in the circulating system of a PDAC patient, which might result from biological heterogeneity or confounding complications, could diminish the performance of a biomarker. Categorization of PDAC patients in terms of their tumor stages and histological types could potentially facilitate patient stratification for treatment.

**Electronic supplementary material:**

The online version of this article (10.1186/s12014-019-9251-3) contains supplementary material, which is available to authorized users.

## Background

Pancreatic ductal adenocarcinoma (PDAC) is one of the most notorious and lethal types of cancer with the worst 5-year survival rate (8%) [1, 2]. Combination chemotherapy such as FOLFIRINOX (fluorouracil, irinotecan, oxaliplatin, and leucovorin) or a combination of gemcitabine and nab-paclitaxel improved survival of metastatic pancreatic cancer patients [3, 4]. Unfortunately, many patients with advanced and metastatic pancreatic cancer have poor performance status and may have difficulty tolerating combination chemotherapy [4].

So far the studies in the predictive markers to chemotherapy for PDAC are quite scarce. A few reported studies include: FKBP51, a scaffolding protein for Akt and PHLPP, which promotes cell death in response to gemcitabine [5]; hENT1, a gemcitabine transporter, which predicts the patients who can potentially benefit from gemcitabine treatment [6]; and hyaluronidase—an enzyme degrades hyaluronic acid to enhance drug delivery [7]. In addition to proteins, therapeutically actionable oncogenes, such as lBRCA1/2, PALB2, and ATM, were also studied as genomic biomarkers for predicting treatment response [8].

Carbohydrate antigen 19-9 (CA 19-9), which detects the epitope of sialyl Lewis(a) on mucins and other adhesive molecules, is currently the primary biochemical test for monitoring the clinical course of pancreatic cancer treatment. Using the decline in CA 19-9 from its initial value to that after 8 weeks of chemotherapy is now well established in metastatic (stage IV), but not locally advanced (stage III) or earlier stages of pancreatic cancer, as a prognostic marker for chemotherapy response/overall survival for both gemcitabine/abraxane and FOLFIRINOX, the two most common treatment regimens [9, 10].

Efforts have been constantly invested to mine the blood proteome of PDAC patients for biomarker development, including plasma or serum, as well as exosomes [11–13]. Nonetheless, little information is available regarding robust biomarkers that are clinical applicable for therapeutic selection and prediction at the time of treatment initiation.

In this study, using the spectral library-based proteomic approach [14–16] and a unique PDAC cohort, we sought to explore the plasma proteome in PDAC patients who received chemotherapy to reveal proteome signals associated with treatment response and survival time. The information gained in this study may benefit future efforts in developing clinical applications for treatment selection and prediction for PDAC patients in the context of personalized medicine.

## Materials and methods

### Patients and plasma samples

The study was approved by the Institutional Review Board at the University of Washington and Virginia Mason Hospital (Seattle, WA). The current study cohort included 16 metastatic (stage IV) PDAC patients for proteomic study, and additional 19 locally advanced (stage III) and 17 metastatic (stage IV) PDAC patients for the validation study (Table 1). Patients were staged according to histology, imaging and clinical assessment. The patients were recruited prospectively for this study and inclusion criteria required newly diagnosed patients who were not treated prior to the first blood draw, while the second blood draw was performed after the chemotherapy. The days between the first and second draws were 62 ± 12 days. Chemotherapy was given according to the clinical acumen of the oncologist (author VJP) and included standard approved agents for pancreatic cancer. For the purposes of this study, the patients were stratified into Good-responders (≥ 12 months) and Limited-responders (< 12 months) in terms of their overall survival. The demographic information of the patients is provided in Additional file 1: Table S1. The blood samples were drawn into purple top tubes (Becton–Dickinson, Franklin Lakes, NJ, USA), with EDTA as an anticoagulant, and then centrifuged at 1200 rpm for 20 min within 4 h. The aliquoted plasma was stored at − 80 °C until analysis.

**Table 1:** Characteristics of PDAC patients at baseline and after the chemotherapy

Stage(s)	Classification	n	Survival (month) (mean ± SD)	CA 19-9 (units/ml) (median (range))	
				Baseline	After chemotherapy
III	Good-responder	11	23.5 ± 4.9	179.5 (85.7, 371.2)	25.6 (9.7, 148.2)
	Limited-responder	8	7.3 ± 3.2	447.4 (50.4, 17,089.3)	517.3 (19.8, 1388.5)
IV	Good-responder	15	20.1 ± 8.1	1116.8 (112.3, 5229.2)	250.1 (13.3, 1851.8.4)
	Limited-responder	18	6.7 ± 3.8	5532.8 (426.0, 64,178.0)	4642.0 (195.7, 88,904.1)
III and IV	Good-responder	26	21.6 ± 7.0	395.9 (67.1, 4451.9)	125.3 (9.0, 795.0)
	Limited-responder	26	6.9 ± 3.5	3164.5 (80.2, 51,831.1)	1123.9 (26.0, 55,992.8)

### Sample preparation for proteomic analysis

Equal volume (6 µl) of plasma from each sample was depleted to remove the top 12 abundant proteins using depletion spin columns (ThermoFisher Scientific, Waltham, MA, USA). The proteins were deglycosylated by PNGase F (New England Biolabs, Ipswich, MA, USA), reduced by 10 mM dithiothreitol at 50 °C for 1 h and alkylated by 25 mM iodoacetimide at room temperature in the dark for 30 min. After buffer exchanged (Vivaspin^®^ 500 filter), the samples were digested with sequencing grade modified trypsin at 1:30 ratio (w:w) at 37 °C for 18 h. The samples were dried down and re-suspended in 50 µl 0.1% formic acid for MS analysis.

### LC–MS/MS analysis

The samples were analyzed in a random order. The LC MS/MS system includes a nanoACQUITY UPLC (Waters, Milford, MA, USA) coupled with a Q Exactive™ plus mass spectrometer (ThermoFisher Scientific). The samples were separated by a C18 analytical column (75 µm × 30 cm) using a linear gradient from 5 to 30% B for 90 min with a flow rate of 0.3 µl/min. Electrospray ionization was operated in a positive mode at a voltage of 2.1 kV. Data-dependent acquisition (DDA) was performed with a mass range from 400 to 1200 m/z. Higher-energy collisional dissociation (HCD) was used for peptide fragmentation.

### Data analysis

The MS data was searched against the UniProt human protein database (2015-07-23) for peptide/protein identification using the Comet algorithm [17] embedded in the Trans-Proteomic Pipeline (TPP v4.6) [18]. Carbamidomethylation of cysteine was set as fixed modification, and oxidation of methionine and deamidation of asparagine were set as variable modifications. The peptide assignment was validated with PeptideProphet [19], and a probability score ≥ 0.9 in correspondence with an FDR of 0.01 was applied to filter the peptides.

The Skyline software (v3.6) [20] was used for quantitative analysis of the DDA data. The spectral library was built using all of the DDA data collected from the 32 samples analyzed. Quantification was made at MS1 level using the sum of the first 3 monoisotopic peaks. The abundance of each peptide was normalized to total ion current (TIC) and presented as ion per million (IPM) using the following formula: Normalized Intensity (IPM) = Peptide Intensity/TIC * 1,000,000. Protein quantification was achieved by summation of the normalized intensities of the corresponding peptides.

Single amino acid variants and global post-translational modifications were analyzed using Comet to search a PSI Extended FASTA Format (PEFF) (http://www.psidev.info/peff) human sequence database from neXtProt [21]. The search results were filtered by a maximum E-value of 10E−4. The spectral counts of peptides were used to compare their abundances.

### CA 19-9 data

CA 19-9 data was provided as part of clinical information. It was measured using chemiluminescence magnetic microparticle immunoassay (CMIA) on the Abott ARCHITECT ci16200 integrated system (Abott, Abott Park, Illinois, USA) according to the clinical protocol established at the Virginia Mason Medical Center.

### Elisa

Four proteins, including PZ, AZGP1, SHBG and VWF, were analyzed in the plasma of a cohort of PDAC patients, using the following testing kits, ZYMUTEST Protein Z kit (Hyphen Biomed, France), zinc-α-2-glycoprotein (human) TurboELISA kit (Adipogen, San Diego, CA, USA), human vWF-A2 DuoSet ELISA kit, and human SHBG DuoSet ELISA kit (R&D systems, Minneapolis, MN, USA), respectively. The absorbance was read on a Synergy H1 Multi-Mode plate reader (BioTek, Winooski, VT, USA) at 450 nm. The samples were tested in duplicate in a randomized and blinded fashion.

### Statistics and bioinformatics analysis

The statistical analysis was performed using SPSS v19.0 (IBM, Armonk, NY, USA). The means of protein abundance or ratio between Good-responders and Limited-responders were compared using Students’ *T* test, while Chi square test was used to compare categorical data. Log-rank test was used to compare the survival between the biomarker-positive and biomarker-negative patients. A P-value < 0.05 was considered statistically significant. The R version 3.3.2 was used to perform principal component analysis (PCA) with prcomp function and plot receiving operating characteristic (ROC) curve with ROCR package [22]. A binary logistic regression model was used to conduct the ROC analysis. Hierarchical data clustering analysis with heat map was done by a R package heatmap.plus with a method introduced by Key [23]. Functional annotation and enrichment analysis were performed using the DAVID v6.8 and the KEGG pathway analysis [24].

## Results

### Evaluation of CA19-9

CA 19-9 was measured in the plasma samples of our study cohort. The value of CA 19-9 at baseline did not show significant difference between Good-responders and Limited-responders regardless PDAC stages (Additional file 2: Figure S1A). The ROC analysis indicated that the AUC values of CA 19-9 for stage III & IV, III and IV are 0.65, 0.58, and 0.65, respectively, in distinguishing Good-responders from Limited-responders (Additional file 2: Figure S1B). As a single biomarker, CA19-9 was not effective in separating Good-responders from Limited-responders at baseline.

### Proteomic analysis and evaluation

**Fig. 1:**
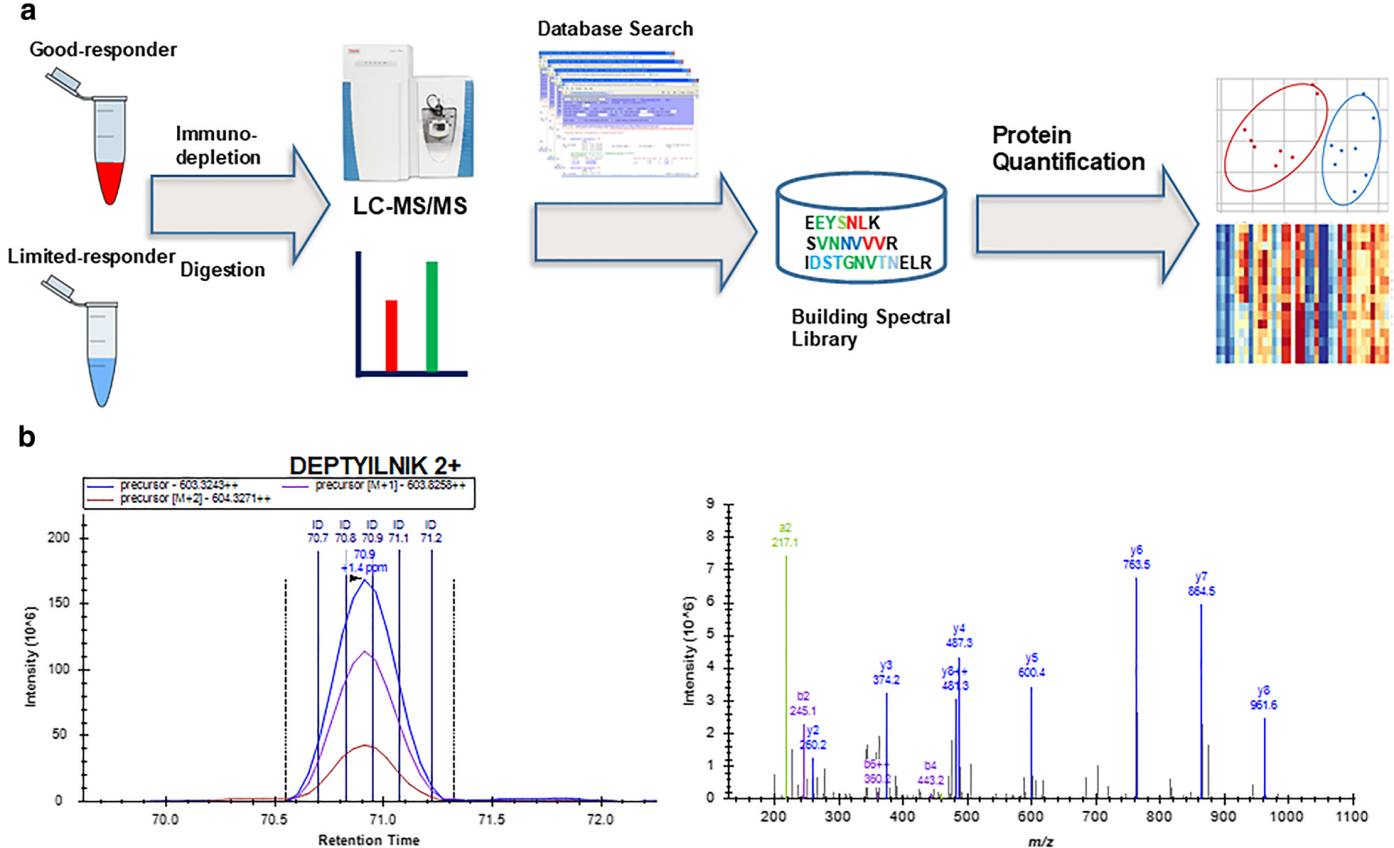
The proteomic work flow applied in the study. **a** A spectral-library-based proteomic approach was used to compare the plasma proteomes between Good-responders and Limited-responders. Plasma samples were immunodepleted, digested and analyzed by LC–MS/MS. A spectral library was constructed for peptide/protein identification and quantification. **b** An example of peptide identification and quantification using spectral library based approach—the elution profile and MS/MS library matching of a doubly charged peptide DEPTYILNIK. The number of lines on the peptide elution profile indicates the number of the times the peptide has been fragmented and identified during the acquisition

A spectral-library based proteomic analysis (Fig. 1) was performed on paired plasma of each of the 16 Stage IV PDAC patients, which included one sample prior to and one sample after chemotherapy treatment (N = 32 samples). The raw data of each sample were analyzed by spectral matching using the cohort specific plasma library, which stores the identification information of peptides, including masses for precursors and product ions, retention times, and charge states. An apparent setback of conventional DDA analysis for a complex sample is its intermitted detection of low abundant peaks for MS/MS analysis in a single analysis, although its ion selection for fragmentation is highly specific. To work around this caveat, we implemented a combined spectral library, which was constructed using all the peptides and proteins identified in the cohort samples (n = 32) with stringent identification criteria. In such a setting, for any peptide that is identified in any one of the samples, its precursor signal will be extracted for all samples within the defined retention time window (5 min) and assigned identification based on precise mass matching.

**Fig. 2:**
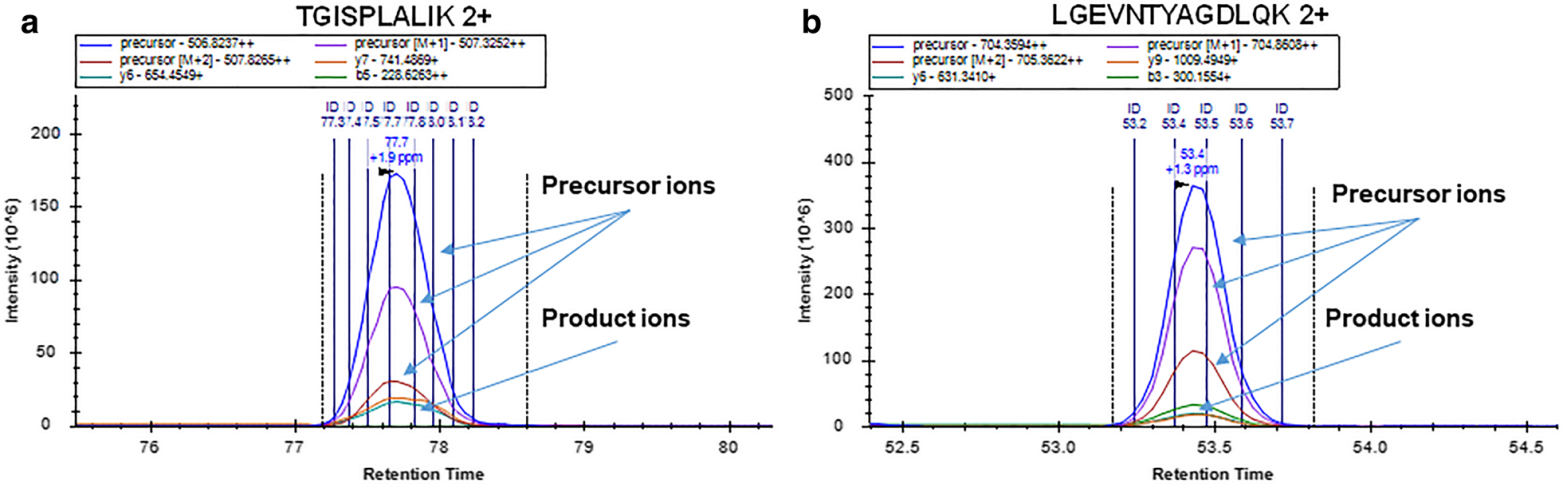
The elution profiles of two peptides with quantifiable precursor and product ions. **a** Peptide TGISPLALIK derived from APOB. **b** Peptide LGEVNTYAGDLQK derived from APOA4

**Fig. 3:**
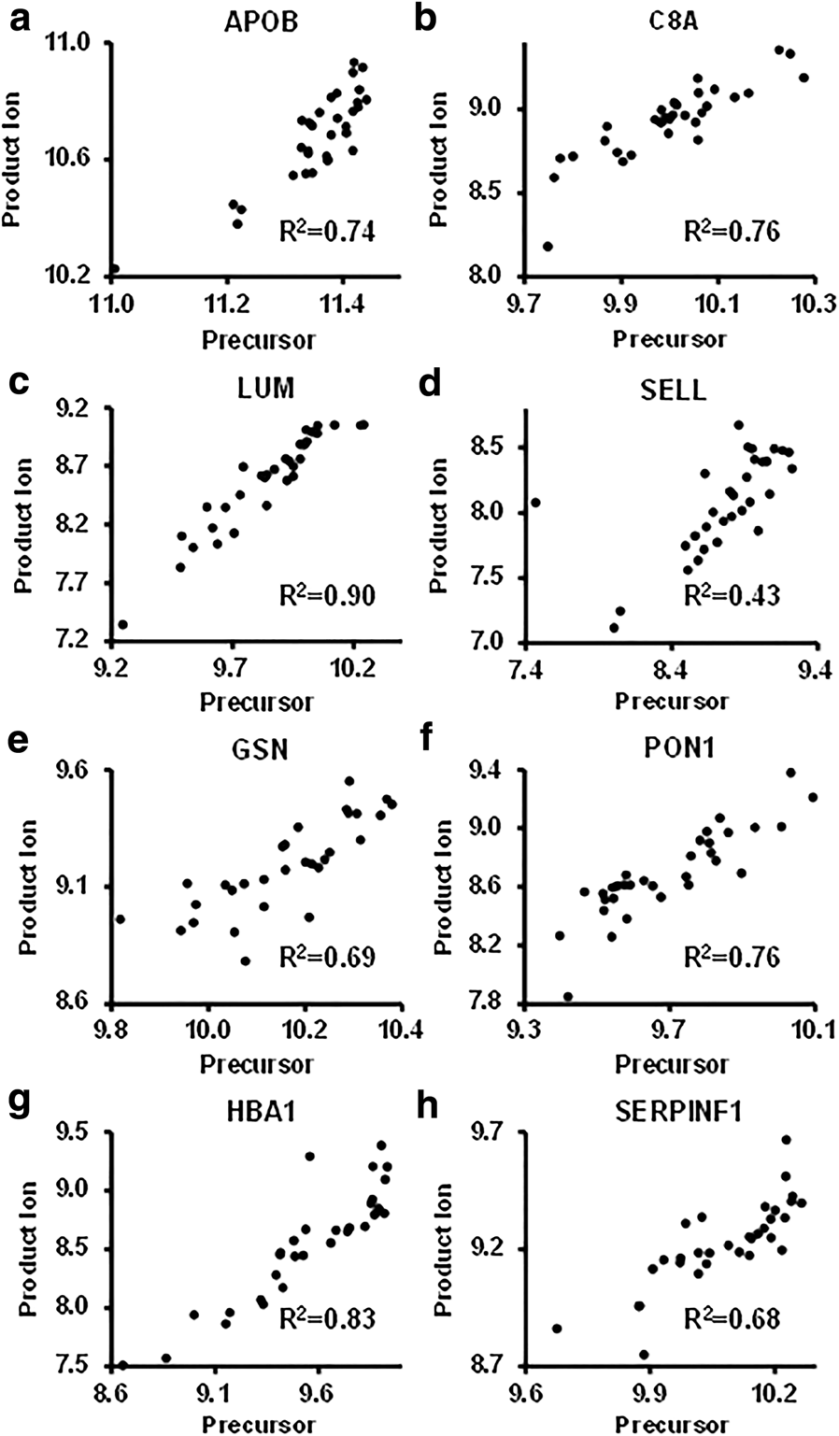
The correlation of protein intensities quantified by precursor and product ions of 8 plasma proteins. The intensities were displayed as the log10 values. **a** APOB. **b** C8A. **c** LUM. **d** SELL. **e** GSN. **f** PON1. **g** HBA1. **h** SERPINF1

For quantification, we compared the detection sensitivity and consistency using either precursor or product ions. As shown in Fig. 2, MS1 signal provides more sensitive detection of the peptides compared to the corresponding product ion peaks. This is particularly obvious for those peptides with low intensities. For quality assurance, we selected 8 proteins with a plasma concentration spanning from 17 to 7.2 × 10^5^ ng/ml [25] to compare the quantification using the intensities of either precursors or product ions. The protein intensities by precursor and product ions of 8 proteins were well correlated in the 32 samples (Fig. 3).

In total, 563 proteins in the patients’ plasma were identified and quantified (Additional file 3: Table S2). Peptides with a normalized intensity ≥ 16 IPM and consistently identified across all 32 samples were considered highly quantifiable (HQ) peptides (Additional file 4: Table S3). The majority of the HQ peptides showed a precise mass matching (deviation < 3 ppm) with theoretical values across all samples (Additional file 5: Figure S2). Such an approach not only enhances the detection of low abundant peptides, which may be missed in a single DDA run for MS/MS identification, but also improves the analytical coverage, sensitivity, and robustness by using a combined spectral library.

Four analytical and biological replicates were prepared to assess the reproducibility and robustness of the method in peptide and protein quantification. The correlations of the intensities and retention time of the HQ peptides, the abundances of their resulting proteins for the analytical and biological replicates showed high linearity (Additional file 6: Figure S3).

### Proteins associated with treatment efficacy and patient prognosis

In the analysis, we were interested in revealing two groups of proteins that are relevant to the patient’s response to the treatment: (1) proteins that expressed differentially at baseline between Good-responders and Limited-responders (baseline differential proteins − *BD* proteins); (2) proteins that showed different abundance changes associated with the first chemotherapy treatment between Good-responders and Limited-responders (Treatment-induced differential proteins − *TID* proteins).

For baseline comparison, two-sample T-test identified 37 *BD* proteins that showed significantly differential expression between the two groups (Additional file 7: Table S4). While the differential expression of these proteins may be influenced by biological heterogeneity, a number of them have been implicated in PDAC [13, 26–36]. Enrichment analysis by KEGG pathway further indicated that the most significant pathways involved were coagulation and complement cascades and glycolysis/gluconeogenesis (Additional file 8: Table S5). It was also notable that at baseline many inflammatory proteins had a lower concentration in Good-responders, including C-reactive protein (CRP), which had lower plasma level (more than 40% lower compared to the mean of all 16 patients) in 6 out of the 8 Good-responders, consistent with previous studies [37].

Another comparison of the plasma proteomes between Good-responders and Limited-responders was made to reveal the plasma proteins that showed differential abundance changes associated with the chemotherapy, i.e. the *TID* proteins. A total of 22 *TID* proteins were identified (Additional file 9: Table S6**)**, and half of them were among the *BD* protein list showing differential abundances at baseline as well (Additional file 7: Table S4). Similarly, the most significant KEGG pathways for *TID* proteins were also coagulation and complement cascades and glycolysis/gluconeogenesis (Additional file 10: Table S7).

**Fig. 4:**
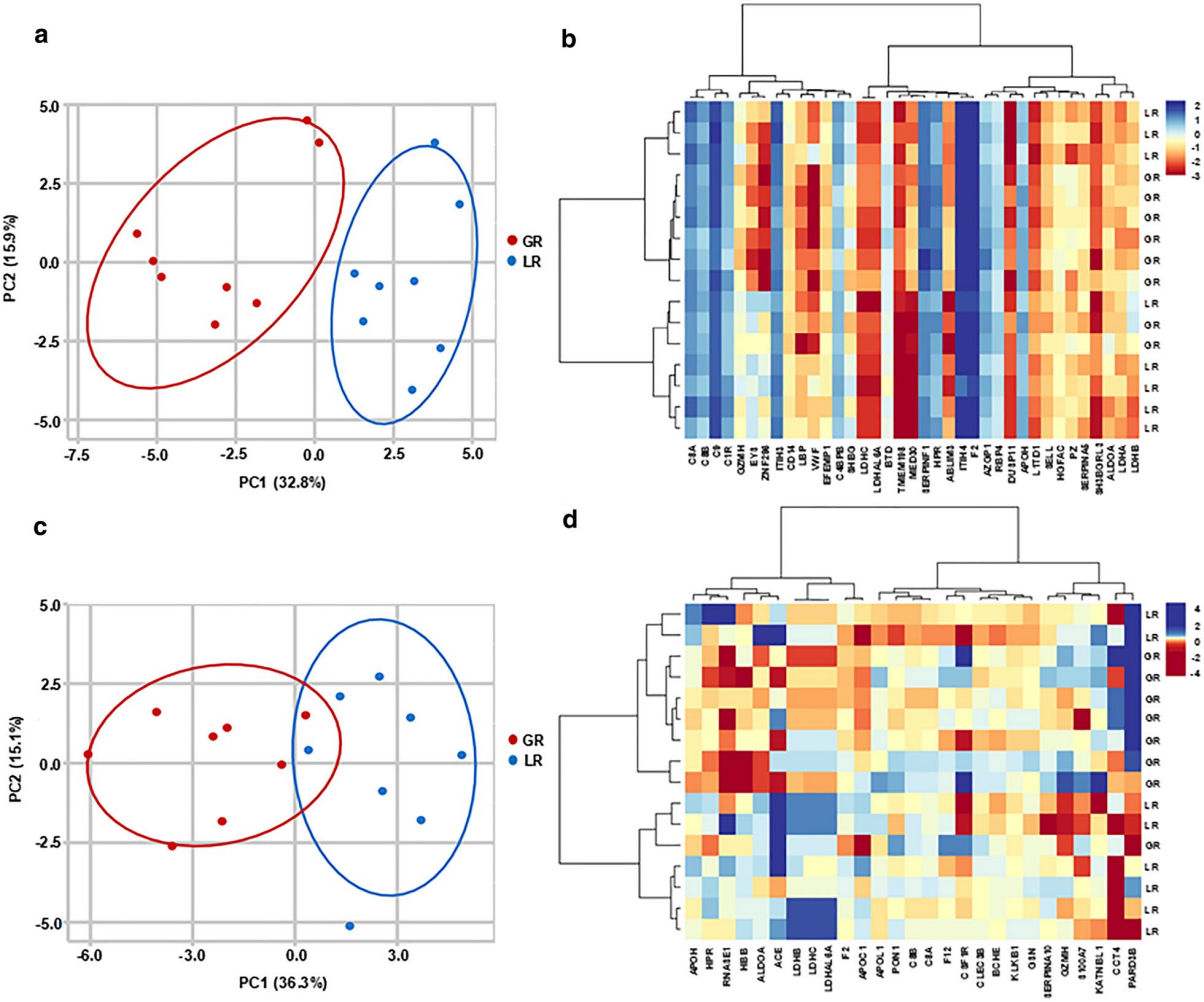
PCA and hierarchical cluster analysis of *BD* and *TIC* proteins. **a** PCA showed that PDAC Good-responders and Limited-responders segregated from each based on the intensities of the *BD* proteins. **b** Heat map representation of the *BD* proteins in PDAC Good-responders versus Limited-responders. The *BD* proteins were ordered by hierarchical clustering with color depicting intensity (log2 value). **c** PCA showed that PDAC Good-responders and Limited-responders did not segregate completely from each based on the ratios of the *TIC* proteins. **d** Heat map representation of the *TIC* proteins in PDAC Good-responders versus Limited-responders. The *TIC* proteins were ordered by hierarchical clustering with color depicting intensity (log2 value). *GR* Good-responders, *LR* Limited-responders

The PCA analysis indicated that complete discrimination between Good-responders and Limited-responders were achieved with the *BD* proteins, but not the *TID* proteins, which were able to separate the two groups at some level, but not to delimit Good-responders and Limited-responders completely (Fig. 4a, c). Inclusion of CA 19-9 in the PCA analysis did not demonstrate significant improvement in discriminating the two groups using either *BD* or *TID* proteins. Hierarchical clustering analysis was not able to completely segregate Good-responders from Limited-responders based upon the intensities of either *BD* or *TID* proteins (Fig. 4b, d).

### Glycopeptides

Using PNGase F to cleave the N-linked glycans from proteins allowed us to interrogate the N-glycosylation abundance change in the plasma proteome of PDAC patients using existing data sets. Without enrichment, 245 deglycosylated N-linked glycopeptides were identified as an additional gain in our experiments. Six N-glycosylated peptides showed differential abundance changes between PDAC Good-responders and Limited-responders at baseline (Additional file 11: Table S8), while seven glycopeptides have different abundance changes associated with the chemotherapy between Good-responders and Limited-responders (Additional file 12: Table S9). The PCA analysis demonstrated that the use of either the *BD* glycopeptides or the *TID* glycopeptides can largely segregate Good-responders and Limited-responders, but not completely (Additional file 13: Figure S4).

### Peptide variant analysis

We also carried out the analysis to identify the single amino acid variants of the plasma proteins to examine whether polymorphism might be a factor affecting the response to chemotherapy in PDAC patients. A total of 2993 variant peptides with amino acid substitution were identified in the plasma of 16 patients, among which 64 peptides have differential abundances between Good-responders and Limited-responders (Additional file 14: Table S10). PCA demonstrated that these peptides could lead to a complete segregation between Good-responders and Limited-responders (Additional file 15: Figure S5).

### ELISA results

**Fig. 5:**
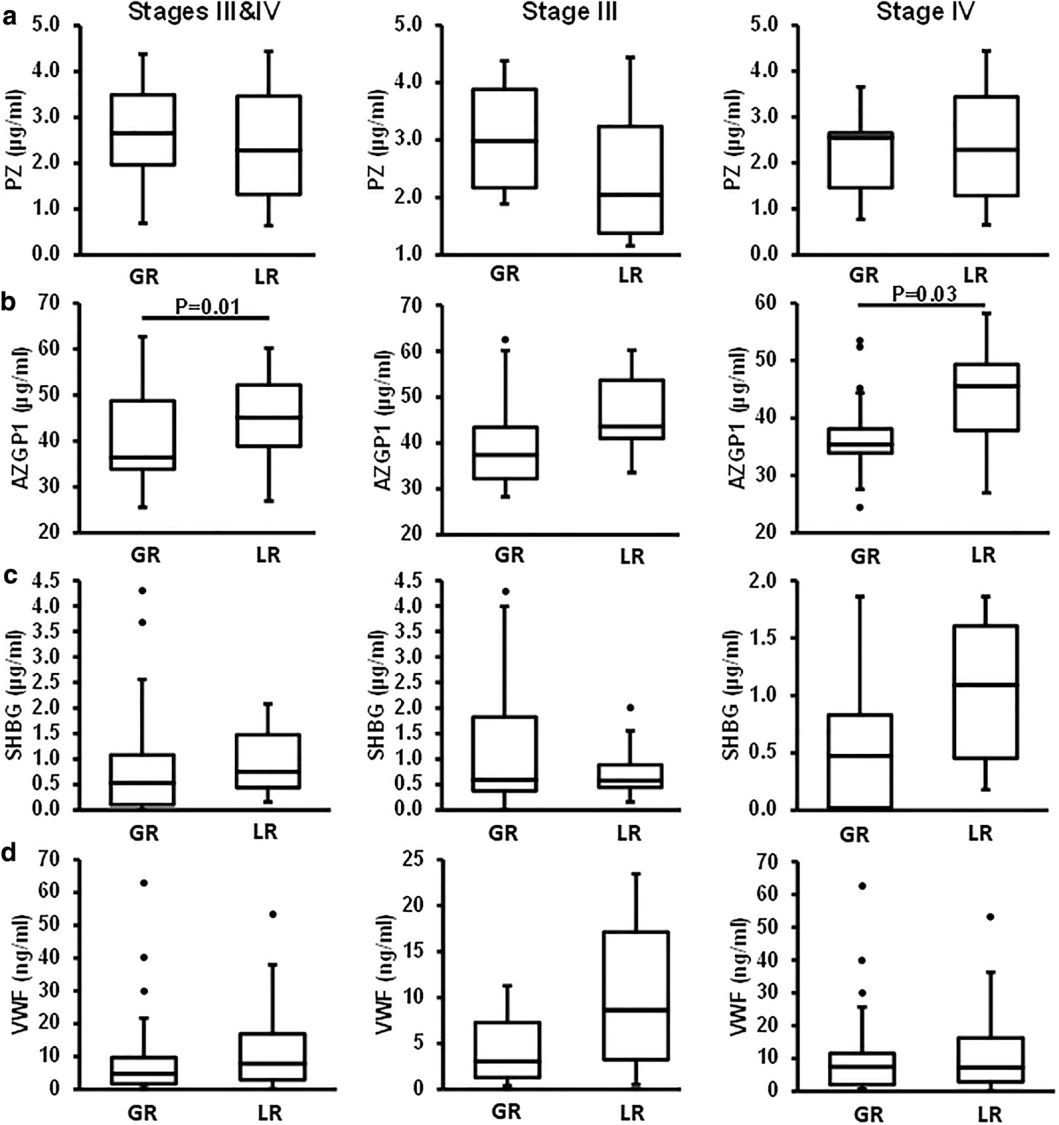
ELISA analysis of four selected proteins. The plasma levels of PZ, AZGP1, SHBG, and VWF at baseline were determined by ELISA in a cohort of PDAC patients (**a**–**d**). The plasma concentrations were illustrated as box plots. *GR* good-esponders, *LR* Limited-responders

**Fig. 6:**
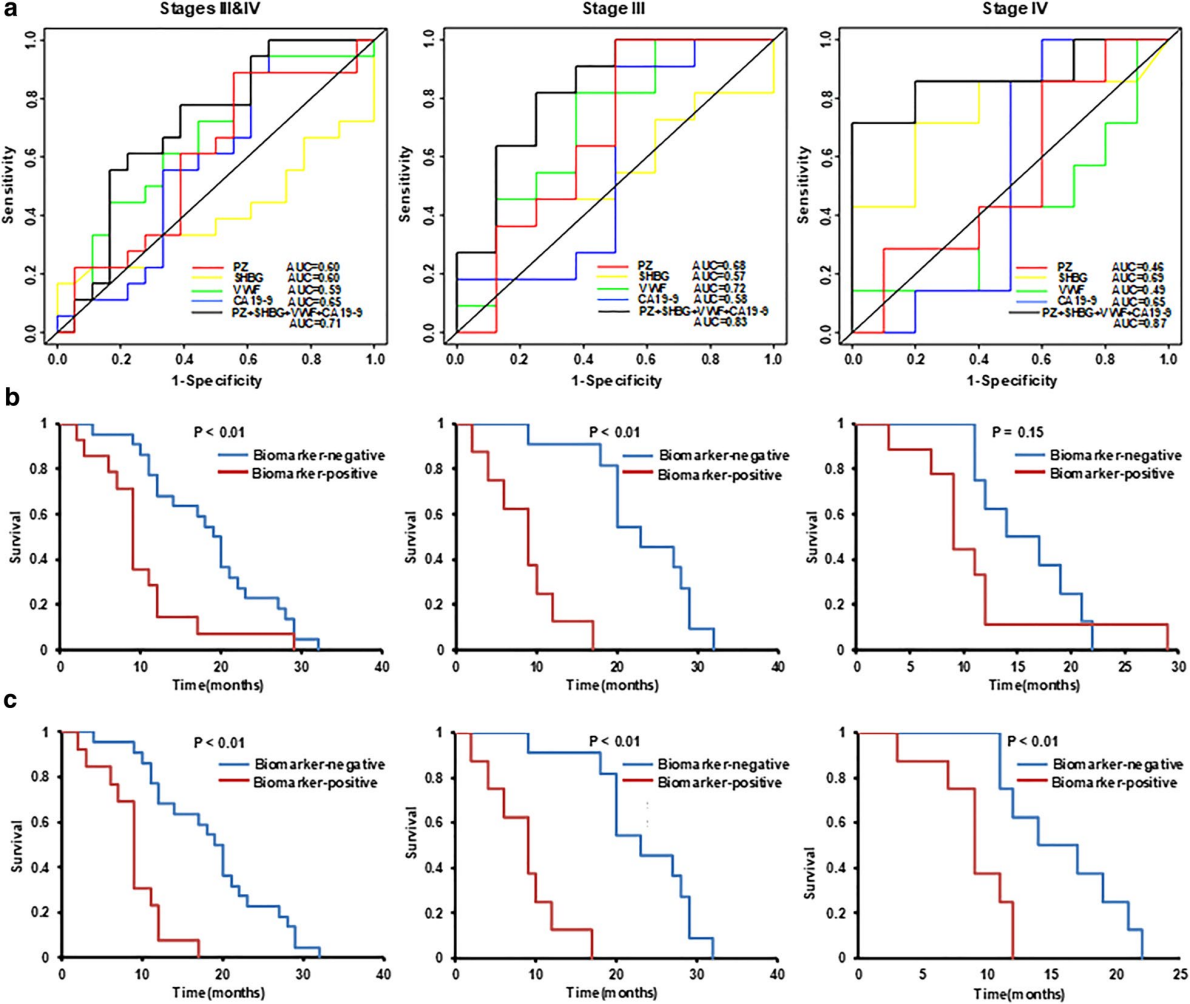
ROC and survival analysis. **a** ROC curves for PZ, SHBG, VWF and CA19-9 as single biomarker, as well as the best-performing composite biomarker in stages III & IV, III, and IV PDAC patients. **b** For all the patients included, the survival curves of PDAC patients stratified by predictive composite biomarker for stages III & IV, III, and IV PDAC patients. **c** After exclusion of one stage IV patient who was Good-responder, but had exceptional high VWF and CA 19-9 concentrations, the survival curves of PDAC patients were significantly improved

Four *BD* proteins, including PZ, AZGP1, SHBG, and VWF, were selected for further testing by ELISA using a cohort of 52 PDAC patients, including 19 stage III and 33 stage IV with metastatic disease (Fig. 5). These four proteins were selected based on their abundance differences between Good-responders and Limited-responders from proteomic data (Additional file 16: Figure S6) and their implications in pancreatic cancer. Notably, the ELISA measurement of AZGP1 was inconsistent with the proteomic data, possibly due in part to its glycosylation forms, which may affect the antibody based detection. For this reason, AZGP1 was excluded from further evaluation. The ROC analyses of PZ, SHBG, VWF and CA 19-9 as a single or composite biomarker are summarized in Fig. 6a and Table 2. To ensure that every patient who would benefit from chemotherapy will get chemotherapy, we anticipate a good predictive biomarker to have high sensitivity and acceptable specificity. When used alone, VWF showed better performance than the other two proteins or CA 19-9 for stage III patients, while SHBG was the best one for stage IV patients. Addition of CA19-9 to each of these proteins was able to improve the AUC values for both stage III and IV. Overall, combination of two or three proteins as a composite biomarker outperformed the single markers and benefitted from the inclusion of CA 19-9, leading to an increase of the AUC values. For all patients combined, one of the best composite biomarkers was the panel of PZ + SHBG + VWF + CA19-9 with an AUC value of 0.71. This panel only achieved a specificity of 39% at 90% sensitivity in distinguishing Good-responders from Limited-responders. However, when the patients were grouped based on their tumor stages, the performance of the composite biomarkers improved substantially for stage III patients. It distinguished Good-responders from Limited-responders with an AUC value of 0.83, and yielded a specificity of 63% at 90% sensitivity. For stage IV patients, it yielded an AUC value of 0.87, leading to a specificity of 30% at 90% sensitivity. Notably, we found that the low specificity for stage IV patients was caused by one Good-responder with a stage IV disease, who had exceptionally high levels of VWF and CA19-9, leading to a false classification of this subject as a Biomarker-positive patient and hence made the separation obscure. By excluding this patient for the analysis, the performance of the biomarker panel improved dramatically. The AUC values for all combined (stage III and IV) and stage IV patients increased to 0.77 (61% specificity at 90% sensitivity) and 0.95 (70% specificity at 90% sensitivity), respectively.

**Table 2:** The summary of ROC analysis of PZ, SHBG, VWF and CA19-9

Marker	Stages III and IV			Stage III			Stage IV		
	AUC	Sensitivity at 90% specificity	Specificity at 90% sensitivity	AUC	Sensitivity at 90% specificity	Specificity at 90% sensitivity	AUC	Sensitivity at 90% specificity	Specificity at 90% sensitivity
PZ	0.60	0.22	0.06	0.68	0	0.50	0.46	0	0.10
SHBG	0.60	0.27	0	0.57	0.27	0	0.69	0.33	0.28
VWF	0.59	0.08	0.04	0.72	0.09	0.38	0.49	0.20	0.17
CA19-9	0.65	0.23	0.38	0.58	0.18	0.50	0.65	0.20	0.44
PZ + CA19-9	0.66	0.22	0.33	0.73	0	0.625	0.70	0	0.40
SHBG + CA19-9	0.62	0.12	0.38	0.72	0.45	0.25	0.78	0.33	0.22
VWF + CA19-9	0.67	0.19	0.38	0.73	0.09	0.5	0.69	0.20	0.50
PZ + SHBG	0.60	0.17	0.22	0.70	0.18	0.5	0.79	0.43	0.30
PZ + VWF	0.64	0.11	0.22	0.72	0	0.50	0.46	0	0.10
SHBG + VWF	0.58	0.08	0.04	0.80	0.36	0.38	0.72	0.47	0.33
PZ + SHBG + CA19-9	0.64	0.17	0.33	0.78	0.27	0.50	0.87	0.86	0.40
PZ + VWF + CA19-9	0.73	0.11	0.39	0.77	0	0.75	0.70	0.29	0.40
SHBG + VWF + CA19-9	0.68	0.23	0.38	0.83	0.36	0.50	0.77	0.47	0.22
PZ + SHBG + VWF	0.65	0.17	0.33	0.75	0.18	0.50	0.80	0.86	0.40
PZ + SHBG + VWF + CA19-9	0.71	0.11	0.39	0.83	0.27	0.63	0.87	0.71	0.30

These proteins were tested as single or composite biomarker for predicting Good-responders from Limited-responders with or without inclusion of CA 19-9. PZ is a vitamin K-dependent glycoprotein, which regulates blood coagulation. SHBG is a glycoprotein that binds hormones, which is an index of testosterone level and an inflammatory marker. VWF is a blood glycoprotein maintaining hemostasis, which also acts in inflammation and antitumor

After deriving the cutoff points of the proteins for the best composite biomarkers (Additional file 17: Table S11), the cohort of PDAC patients were divided into two groups based on the biomarker threshold: Biomarker-positive and Biomarker-negative. If a composite biomarker meets the cut point criteria, i.e. “Biomarker-positive”, it suggests a potential Limited-responder, and vice versa.

The survivals between the Biomarker-positive patients and Biomarker-negative patients at different stages were compared by log-rank test as shown in the Fig. 6b. When all the patients (stages III and IV) were tested together, the median survival was 19.2 months (95% confidence interval (CI) 11.4–22.1 months) for the Biomarker-negative patients, as compared to 8.7 months (95% CI 6–11.7 months) for the Biomarker-positive patients. For stage III, Biomarker-negative patients (median 22.1 months, 95% CI 17.4–28.8 months) showed significant improved survival compared to Biomarker-positive patients (median 8.7 months, 95% CI 1.9–17 months) with a P < 0.01. However, for stage IV, the separation between the Biomarker-negative patients (median 15.3 months, 95% CI 10.5–21.3 months) and Biomarker-positive patients (median 8.9 months, 95% CI 6.7–11.7 months) was not significant. Again, after exclusion of the outlier stage IV patient who had exceptional high level of VWF and CA 19-9, for all patent groups, the biomarker panel was able to separate Good-responders from Limited-responders significantly based on their survival time (Fig. 6c). These data exemplified the challenges in blood biomarker development in PDAC, as the blood concentration of a protein could be influenced by many factors, including biological heterogeneity and other confounding complications due to malignancies and inflammatory diseases.

## Discussion

In this study, we applied the spectral library based quantitative proteomics to investigate the plasma proteome of the PDAC patients with different treatment responses and survival time. The PCA analysis using the *BD* proteins was able to segregate Good-responders from Limited-responders with statistical significance. The *BD* and *TID* proteins showed significant overlap and were implicated in pathways related to coagulation, complement cascades and glycolysis/gluconeogenesis. Many of these proteins have previously been reported with an involvement in pancreatic tumorigenesis and drug resistance. The level of many inflammatory proteins were lower in the plasma from Good-responders.

For predictive biomarkers, we are looking for biomarkers with high sensitivity. For a biomarker with ≥ 90% sensitivity and ≥ 50% specificity, it will ensure at least 90% of Good-responders would get treatment and benefit from the chemotherapy, while save half of Limited-responders from the unnecessary toxicity from treatment. For the PDAC patients that we tested, as an individual biomarker, the ELISA of the three protein candidates PZ, SHBG and VWF, as well as CA19-9, were not robust enough to distinguish Good-responders from Limited-responders. Combination of two or more proteins as a composite biomarker boosted the performance in a cohort comprised of stage III and IV PDAC patients. While the panel that consists of PZ, SHBG, VWF and CA 19-9 demonstrated the best performance in segregating Good-responders from the Limited-responders, a stage IV patient who was a Good-responder, but had exceptionally high levels of VWF and CA 19-9 readings, was falsely categorized. Just like CA 19-9, which does not work well on the patients who lack of sialyl Lewis(a) antigen, the performance of the biomarker could be diminished due to biological heterogeneity and/or other confounding factors, including chronic pancreatitis, jaundice, diabetes and other diseases. Compared to the stage III patients who have locally advanced PDAC, this may be more of the case for stage IV patients, many of whom have a metastatic disease and other complications. Classification of PDAC patients with clinical parameters, including tumor stages and histological types might give rise to the improvement in the sensitivity and specificity of biomarkers.

It is also notable that compared to mass spectrometric data, the ELISA measurement showed dramatically compressed differences between Good-responders and Limited-responders (Additional file 18: Figure S7). The discrepancies could be attributed to several factors, including: (1) The dynamic range and sensitivity of the two approaches vary due to different measuring mechanisms; 2) Proteomics tends to measure total proteins in the plasma, whereas ELISA may only measure proteins in free form; (3) Splice variants and post-translational modifications, such as glycosylation, could affect detection specificity of an antibody based measurement.

All three individual candidates included in the biomarker panel have been previously related to the progression and prognosis of PDAC. The serum levels of SHBG were elevated in advanced pancreatic cancer patients, which may imply a worsened survival as compared to the controls [36]. It is hypothesized that SHBG may correlate with inflammation during acute phase [38]. Abnormal coagulation is one of the most frequently encountered complications by oncologists, which is attributed to multiple factors, such as tumor type and disease stage. PZ, which was of higher abundance in Good-responders, is involved in the regulation of blood coagulation. Descending PZ concentrations in the patients with malignant tumors were observed, which coincided with tumor progression and may imply poor prognosis [39]. VWF has essential functions in platelet adhesion and inflammation [40]. The relatively lower plasma concentration of VWF in Good-responders compared to Limited-responders appeared to be consistent with other inflammatory proteins, such as CRP, and might imply a different immunoresponse and a less degree of angiogenesis complication [41].

The sequence variant analysis demonstrated that amino acid polymorphism is not a rare event in plasma proteins. Among the variants that were identified with differential plasma level between Good-responders and Limited responder, many of them are derived from a few proteins, including ceruloplasmin, plasminogen, retinol-binding protein 4, transthyretin, apolipoprotein B-100, complement component C7. Interestingly, two transthyretin variants (Gln89 and Tyr114), detected with higher level in Good-responders, were reported to be associated with amyloid polyneuropathy [42–44]. Since only a handful of these variations have been sparsely studied previously due to the lack of technology for global analysis, the changes of these variants in association with the difference in patient response warrant further investigation. These single amino acid polymorphisms could be traced back to genome-wide single nucleotide polymorphism (SNP) studies, and may add new knowledge towards how and whether these mutations contribute to malignancies and chemoresistance.

## Additional files

**Additional file 1: Table S1.** Demographic information of the PDAC patients involved in the proteomic study.

**Additional file 2: Figure S1.** Evaluation of baseline CA19-9 in distinguishing Good-responders from Limited-responders.

**Additional file 3: Table S2.** Proteins identified in the plasma from the PDAC patients.

**Additional file 4: Table S3.** The HQ peptide list.

**Additional file 5: Figure S2.** Histograms for the mass deviations of the HQ peptides.

**Additional file 6: Figure S3.** The correlation of the intensities and retention time of the HQ peptides, and the intensities of their resulting proteins for the analytical (A-C) and biological replicates (D-F).

**Additional file 7: Table S4.** The *BD* proteins between Good-responders and Limited-responders.

**Additional file 8: Table S5.** KEGG pathway analysis of the *BD* proteins.

**Additional file 9: Table S6.** The *TID* proteins between Good-responders and Limited-responders.

**Additional file 10: Table S7.** KEGG pathway analysis of the *TID* proteins.

**Additional file 11: Table S8.** The *BD* N-linked glycopeptides.

**Additional file 12: Table S9.** The *TIC* N-linked glycopeptides.

**Additional file 13: Figure S4.** PCA analysis of glycopeptides.

**Additional file 14: Table S10.** The variant peptides with significant difference (p < 0.05) between Good-responders and Limited-responders.

**Additional file 15: Figure S5.** PCA analysis of significant variant peptides.

**Additional file 16: Figure S6.** Four plasma proteins PZ, AZGP1, SHBG, and VWF showed statistically significance in the volcano plot.

**Additional file 17: Table S11.** The cut points of candidate biomarkers for stratifying PDAC patients.

**Additional file 18: Figure S7.** Concentration ratios of PZ, SHBG and VWF between Good-responders and Limited-responders at baseline by proteomics and ELISA.

## Data Availability

The datasets used during the current study are available from the corresponding author on reasonable request.

## Abbreviations

AUC: area under the curve
BD: baseline differential
BMI: body mass index
DDA: data-dependent acquisition
ELISA: enzyme-linked immunosorbent assay
FDR: false discovery rate
FOLFIRINOX: fluorouracil, irinotecan, oxaliplatin, and leucovorin
HCD: higher-energy collisional dissociation
HQ: highly quantifiable
IPM: ion per million
LC–MS/MS: liquid chromatography coupled with tandem mass spectrometry
PCA: principal component analysis
PDAC: pancreatic ductal adenocarcinoma
PEFF: PSI extended fasta format
SNP: single nucleotide polymorphism
TIC: total ion count
TID: treatment-induced differential
TPP: trans-proteomics pipeline
ROC: receiving operating characteristic
