# Supplementary material for: Predictive proteomic signatures for response of pancreatic cancer patients receiving chemotherapy
Source: Clin Proteomics. 2019 Jul 17;16:31. doi: 10.1186/s12014-019-9251-3 (PMC6636003; doi:10.1186/s12014-019-9251-3)
Supplement: Supplementary file 1 — Additional file 1: Table S1. Demographic information of the PDAC patients involved in the proteomic study. [file 12014_2019_9251_MOESM1_ESM.pdf]

Table S1. The demographic information of the PDAC patients involved in the study. No significant differences in age, gender and BMI were found between Good-responders and Limited-responders (P>0.05).

stage	classification	age (mean±std)	gender		BMI (mean±std)
			male	female	
III	Good-responder	66.8±12.9	6	5	27.3±7.6
	Limited-responder	62.4±8.5	5	3	23.2±4.6
IV	Good-responder	64.0±10.5	7	8	26.3±5.2
	Limited-responder	66.0±8.3	7	11	26.0±5.3
III&IV	Good-responder	63.0±9.5	13	13	26.7±6.2
	Limited-responder	66.0±9.7	12	14	25.1±5.2

BMI: body mass index
