# Supplementary material for: Predictive proteomic signatures for response of pancreatic cancer patients receiving chemotherapy
Source: Clin Proteomics. 2019 Jul 17;16:31. doi: 10.1186/s12014-019-9251-3 (PMC6636003; doi:10.1186/s12014-019-9251-3)

Figure S1. Evaluation of baseline CA19-9 in distinguishing Good-responders from Limited-responders. (A) Measurement of CA19-9. (B) ROC analysis.

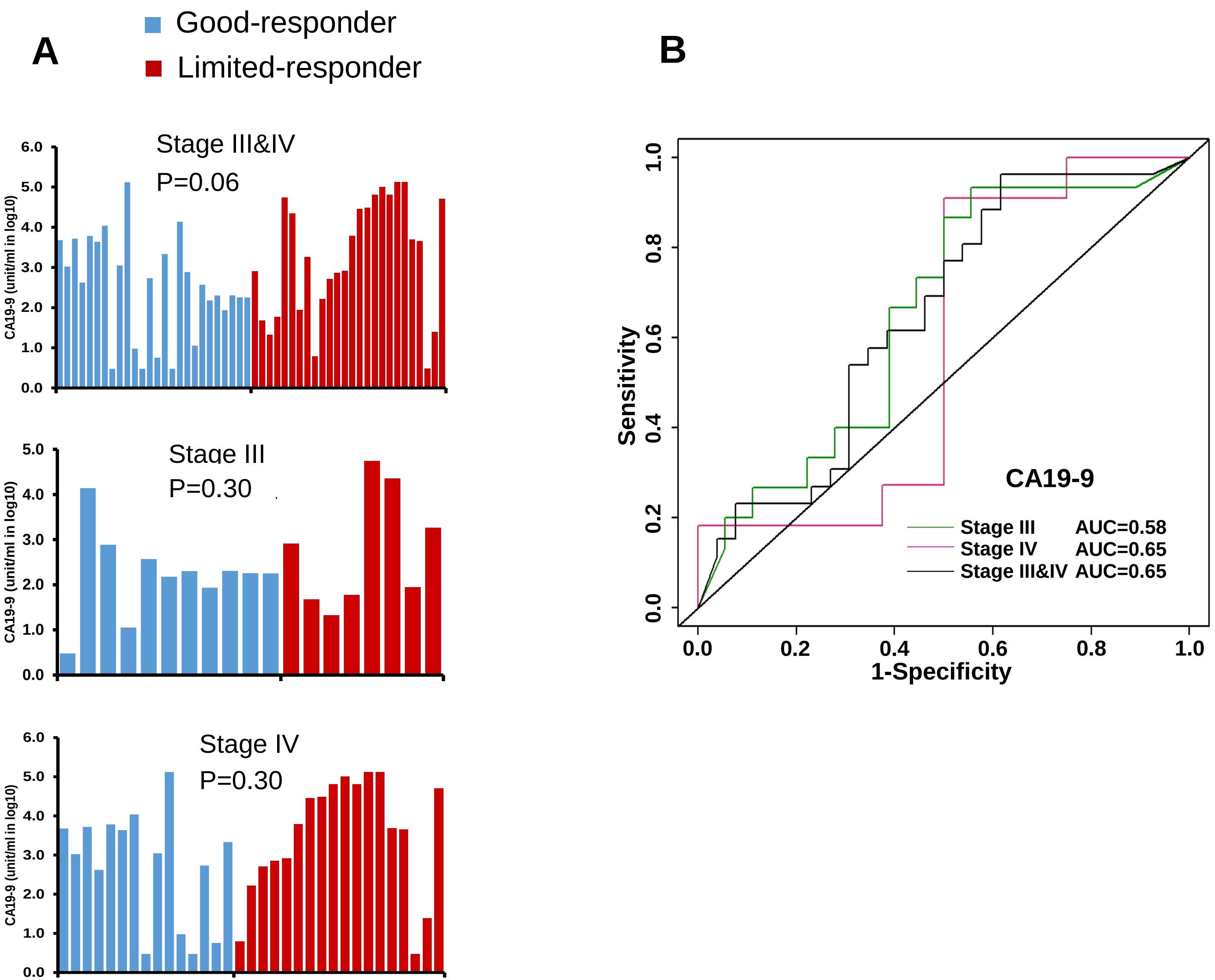

Supplement: Supplementary file 2 — Additional file 2: Figure S1. Evaluation of baseline CA19-9 in distinguishing Good-responders from Limited-responders. [file 12014_2019_9251_MOESM2_ESM.pdf]
