# Supplementary material for: Predictive proteomic signatures for response of pancreatic cancer patients receiving chemotherapy
Source: Clin Proteomics. 2019 Jul 17;16:31. doi: 10.1186/s12014-019-9251-3 (PMC6636003; doi:10.1186/s12014-019-9251-3)
Supplement: Supplementary file 3 — Additional file 3: Table S2. Proteins identified in the plasma from the PDAC patients. [file 12014_2019_9251_MOESM3_ESM.pdf]

Table S2. List of protein identification

Uniprot ID	protein probability	protein name	gene name	protein length	percent coverage	peptides	# peptide identified
A1L162	0.9407	Glutamate-rich protein 2	ERICH2	156	25.6	QEKNNEYCLQDIDDKLSESAEDDGEDDTNDEDDDEDSNPK	1
A1L4H1	1	Soluble scavenger receptor cysteine-rich domain-containing protein SSC5D	SSC5D	1573	3.1	GNEGQLGLCHHR+SNCDHSEDAGLVCTGPAPR+THYGPGTGPIWLDDMGCK	3
A2A368	0.9407	Melanoma-associated antigen B16	MAGEB16	324	5.6	GVPKTGLLIIVLGVIFMK	1
A6BM72	1	Multiple epidermal growth factor-like domains protein 11	MEGF11	1044	3	WGPNCSVSCSCENGGSCSPEDGSCECAPGFR	1
A6NFE3	0.9297	EF-hand calcium-binding domain-containing protein 10	EFCAB10	127	23.6	EVVSYLTSALLFFRPEKPKEYLISLLERLR	1
A6NMU1	0.9407	Putative olfactory receptor 52A4	OR52A4	304	10.9	QLVTYIVVGVTLRPAILVIPCLLLIKCHLKLYR	1
A8MQ27	0.9407	E3 ubiquitin-protein ligase NEURL1B	NEURL1B	555	5.9	NGECTVCFDGEVDTVIYTCGHMCLCHSCGLRLK	1
A8MUU1	0.9407	Putative fatty acid-binding protein 5-like protein 3	FABP5P3	101	20.8	TQTVCNFTDGALVQHQEWDGK	1
O00151	1	PDZ and LIM domain protein 1	PDLIM1	329	23.1	AALANLCIGDVITAIDGENTSNMTHLEAQNR+QSTSFLVLQEILESEEK+VITNQYNNPAGLYSSENISNFNNALESK	3
O00161	0.9407	Synaptosomal-associated protein 23	SNAP23	211	6.2	AHQITDESLESTR	1
O00299	1	Chloride intracellular channel protein 1	CLIC1	241	25.7	EEFASTCPDDEEIELAYEQVAK+IEEFLEAVLCPPR+VLDNYLTSPLPEEVDETSAEDEGVSQR	3
O00391	1	Sulfhydryl oxidase 1	QSOX1	747	15.5	AAPGQEPPEHMAELQR+AHFSPSNIILDFPAAGSAAR+DCASHFEQMAAASMHR+DVQNVAAAPELAMGALELESR+ELCSACHNER+SALYSPSDPLTLLQADTVR+VNWIGCQGSEPHFR	7
O00468	0.9407	Agrin	AGRN	2067	1.5	DPCSNVTCSFGSTCARSADGLTASCLCPATCR	1
O00469	0.9407	Procollagen-lysine,2-oxoglutarate 5-dioxygenase 2	PLOD2	737	3.3	ACDELVEEMEHYGKWSGGKHHDSR	1
O00533	1	Neural cell adhesion molecule L1-like protein	CHL1	1208	12.3	DGNPFYFTDHR+KPQSAVYSTGSNGILLCEAEGEPQPTIK+SQPSQPSDHHETPPAAPDR+SQPSQPSDHHETPPAAPDRNPQNIR+VNGSPVDNHPFAGDVVFPR+VQAINQLGSGPDPQSVTLYSGEDYPDTAPVIHGVDVINSTLVK+VTWKPQGAPVEWEEETVTNHTLR	7
O00602	0.9352	Ficolin-1	FCN1	326	4.3	VDLVDFEGNHQFAK	1
O14514	0.9189	Brain-specific angiogenesis inhibitor 1	BAI1	1584	2	DCGGGLQTRTRTCLPAPGVEGGGCEGVLEEGR	1
O14786	1	Neuropilin-1	NRP1	923	3.7	IESPGYLTSPGYPHSYHPSEK+IKPATWETGISMR	2
O14791	0.9975	Apolipoprotein L1	APOL1	398	9.5	ANLQSVPHASASRPR+LKSELEDNIR+WWTQAQAHDLVIK	3
O14926	0.9407	Fascin-2	FSCN2	492	5.5	QGVNVSANQDDELDHETFLMQIDQETK	1
O14950	1	Myosin regulatory light chain 12B	MYL12B	172	21.1	ATSNVFAMFDQSQIQEFK+DGFIDKEDLHDMLASLGK+EDLHDMLASLGK	3
O15143	0.9955	Actin-related protein 2/3 complex subunit 1B	ARPC1B	372	11	STVLSLDWHPNNVLLAAGSCDFK+VAWVSHDSTVCLADADKK	2
O15144	0.9992	Actin-related protein 2/3 complex subunit 2	ARPC2	300	7.3	DNTINLIHTFR+DSIVHQAGMLK	2
O15145	0.9352	Actin-related protein 2/3 complex subunit 3	ARPC3	178	6.2	VFDPQNDKPSK	1
O15204	0.9407	ADAM DEC1	ADAMDEC1	470	7.2	CLLQAPIPTNIMTTPVCGNHLLEVGEDCDCGSPK	1

O15511	0.9407	Actin-related protein 2/3 complex subunit 5	ARPC5	151	12.6	GFESPSDNSSAMLLQWHEK	1
O43315	0.9352	Aquaporin-9	AQP9	295	12.2	NLGAPRGLEPIAIGLLIIVIASSLGLNSGCAMNPAR	1
O43707	1	Alpha-actinin-4	ACTN4	911	12.5	HEAFESDLAAHQDR+HRPELIEYDK+HTNYTMEHIR+KHEAFESDLAAHQDR+MLDAEDIVNTARPDEK+RDHALLEEQSK+TFTAWCNSHLR+TINEVENQILTR+VGWEQLLTTIAR+VLAVNQENEHLMEDYEK	10
O43852	0.9407	Calumenin	CALU	315	5.1	DWILPSDYDHAEAEAR	1
O43866	1	CD5 antigen-like	CD5L	347	15.9	CSGEEQSLEQCQHR+GVWGSVCDDNWGEKEDQVVCK+NTCNHDEDTWVECEDPFDLR	3
O60315	0.9407	Zinc finger E-box-binding homeobox 2	ZEB2	1214	1.3	METKSDHEEDNMEDGM	1
O75074	0.9032	Low-density lipoprotein receptor-related protein 3	LRP3	770	3.1	CDGLQDCGDGSDEAGCPDLACGRR	1
O75083	1	WD repeat-containing protein 1	WDR1	606	19.6	AHDGGIYAISWSPDSTHLLSASGDK+CFSIDNPGYEPEVVAVHPGGDTVAIGGVDGNVR+NIDNPALADIYTEHAHQVVVAK+NNPSKPLHVIK+SYIYSGSHDGHINYWDSETGENDSFAGK	5
O75096	0.9144	Low-density lipoprotein receptor-related protein 4	LRP4	1905	3.2	FTDPGMGNLTYSNPSYR+SLWHCDGDNDCGDNSDEQCDMR+SWVCDGDNDCEDDSDEQDCPPR	3
O75122	0.9032	CLIP-associating protein 2	CLASP2	1294	2.1	KTYMGLRNHFPGEAETLYNSLEPSYQK	1
O75159	0.9407	Suppressor of cytokine signaling 5	SOCS5	536	2.8	YGVSSVHDMDSVSSR	1
O75319	0.903	RNA/RNP complex-1-interacting phosphatase	DUSP11	330	2.424	HAVNGFLK	1
O75369	0.9717	Filamin-B	FLNB	2602	1.5	GQHVTGSPFQFTVGPLGEGGAHK+QMQLENVSVALEFLDR	2
O75636	1	Ficolin-3	FCN3	299	31.4	AGFGNQESEFWLGNENLHQLTLQGNWELR+ALPVFCDMDTEGGGWLVFQR+ELLSQGATLSGWYHLCLPEGR+LLGEVDHYQLALGK+RQDGSVDFFR	5
O75874	0.9407	Isocitrate dehydrogenase [NADP] cytoplasmic	IDH1	414	3.1	TVEAEAAHGTVTR	1
O75881	0.9407	25-hydroxycholesterol 7-alpha-hydroxylase	CYP7B1	506	4.9	LGLPGLALAAALLLLALCLLVRRTR	1
O75882	1	Attractin	ATRN	1429	25.6	AATCINPLNGSVCERPANHSAK+CDQHTDCYSCTANTNDCHWCNDHCVPR+CNPGTGQCVCPAGWVGEQCQHCGGR+ECDRPCVNGGR+EQYAVVGHSAHIVTLK+GCSCFSDWQGPGCSVPVPANQSFWTR+GEACDIPHCTDNCGFPHR+GVKGDECQLCEVENR+HCETCISGFYGDPTNGGK+LTLTPWVGLRK+MPSQAPTGNFYPQPLLNSSMCLEDSR+NHNALLASLTTQK+NHSCSEGQISIFR+NQECIALPENICGIGWHLVGNSCLK+NTWSILHTQGALVQGGYGHSSVYDHR+SCALDQNCQWEPR+SEAACLAAGPGIR+VFHIHNESWVLLTPK+WSVLPRPDLHHDVNR+YNWSFIHCPACQCNGHSK	20
O94875	0.9297	Sorbin and SH3 domain-containing protein 2	SORBS2	1100	1.5	SCDDLLNDDCDSFPDPK	1
O94929	0.8981	Actin-binding LIM protein 3	ABLIM3	683	1.903	HFHIPAGDSNIYR	1
O95391	0.9352	Pre-mRNA-splicing factor SLU7	SLU7	586	6.8	HQWGEEEPNSQMEKDHNSEDEDEDKYADDIDMPGQNFDSK	1
O95445	1	Apolipoprotein M	APOM	188	45.2	EELATFDPVDNIVFNMAAGSAPMQLHLR+EFPEVHLGQWYFIAGAAPTK+KWIYHLTEGSTDLR+TELFSSSCPGGIMLNETGQGYQR+WIYHLTEGSTDLR	5
O95497	1	Pantetheinase	VNN1	513	4.7	MSENIPNEVYALGAFDGLHTVEGR	1
O95789	0.9407	Zinc finger MYM-type protein 6	ZMYM6	1325	2.4	MCSYCSQTSPNLVENRLEGKLEEFCCEDCMSK	1

O95810	1	Serum deprivation-response protein	SDPR	425	16.9	DNSQVNAVTVLTLLDK+LENNHAQLLR+QEKPSSPSPMPSSTPSPSLNLGNTEEAIR+VLIFQEENEIPASVFVK	4
O95998	0.9407	Interleukin-18-binding protein	IL18BP	194	14.9	ALVLEQLTPALHSTNFSCVLVDPEQVVQR	1
O96014	0.9407	Protein Wnt-11	WNT11	354	5.4	QCNKTSNGSDSCDLMCCGR	1
P00338	1	L-lactate dehydrogenase A chain	LDHA	332	4.8	TLHPDLGTDKDKEQWK	1
P00390	1	Glutathione reductase, mitochondrial	GSR	522	7.5	ADFDNTVAIHPTSSEELVTLR+GHAAFTSDPKPTIEVSGK	2
P00414	1	Cytochrome c oxidase subunit 3	MT-CO3	261	5.4	ESTYQGHHTPPVQK	1
P00441	1	Superoxide dismutase [Cu-Zn]	SOD1	154	23.4	DGVADVSIEDSVISLSGDHCIIGR+HVGDLGNVTADKDGVADVSIEDSVISLSGDHCIIGR	2
P00450	1	Ceruloplasmin	CP	1065	62.4	[illegible] TR+AEEEHLGILGPQLHADVGDK+AEEEHLGILGPQLHADVGDKVK+AETGDKVYVHLK+AGLQAFFQVQECNK+ALYLQYTDETFR+DIASGLIGPLIICK+DIASGLIGPLIICKK+DIFTGLIGPMK+DLYSGLIGPLIVCR+DTANLFPQTSLTLHMWPDTEGTFNVECLTTDHYTGGMK+DVDKEFYLFPTVFDENESLLLEDNIR+EFYLFPTVFDENESLLLEDNIR+EHEGAIYPDNTTDFQR+ELHHLQEQNVSNAFLDK+ELHHLQEQNVSNAFLDKGEFYIGSK+ENLTAPGSDSAVFFEQGTTR+ERGPEEEHLGILGPVIWAEVGDTIR+EVGPTNADPVCLAK+EYTDASFTNRK+FNKNNEGTYYSPNYNPQSR+GAYPLSIEPIGVR+GPEEEHLGILGPVIWAEVGDTIR+HYYIGIIETTWDYASDHGEK+KAEEEHLGILGPQLHADVGDK+KAEEEHLGILGPQLHADVGDKVK+KALYLQYTDETFR+KLISVDTEHSNIYLQNGPDR+LISVDTEHSNIYLQNGPDR+MFTTAPDQVDKEDEDFQESNK+MHSMNGFMYGNQPGLTMCK+MYSVNGYTFGSLPGLSMCAEDR+MYYSAVDPTKDIFTGLIGPMK+NLASRPYTFHSHGITYYK+NMATRPYSIHAHGVQTESSTVTPTLPGETLTYVWK+NNEGTYYSPNYNPQSR+QKDVDKEFYLFPTVFDENESLLLEDNIR+QSEDSTFYLGER+QYTDSTFRVPVER+RDTANLFPQTSLTLHMWPDTEGTFNVECLTTDHYTGGMK+RQSEDSTFYLGER+SGAGTEDSACIPWAYYSTVDQVK+SVPPSASHVAPTETFTYEWTVPK+TPGIWLL	50
P00451	0.9407	Coagulation factor VIII	F8	2351	0.6	EDFDIYDEDENQSPR	1
P00488	1	Coagulation factor XIII A chain	F13A1	732	14.5	AVPPNNSNAAEDDLPTVELQGVVPR+DGTHVVENVDATHIGK+EIRPNSTVQWEEVCRPWVSGHR+GVNLQEFLNVTSVHLFK+KDGTHVVENVDATHIGK+NPETDTYILFNPWCEDDAVYLDNEK	6
P00558	1	Phosphoglycerate kinase 1	PGK1	417	11.5	AHSSMVGVNLPQK+ALESPERPFLAILGGAK+ITLPVDFVTADKFDENAK	3
P00724	1	Invertase 2	SUC2	532	9.4	FSLNTEYQANPETELINLK+LESAFANEGFLGYQYECPGLIEVPTEQDPSK	2

P00734	1	Prothrombin	F2	622	51.6	DKLAACLEGNCAEGLGTNYR+ETAASLLQAGYK+GQPSVLQVVNLPIVERPVCK+HQDFNSAVQLVENFCR+ITDNMFCAGYKPDEGK+ITDNMFCAGYKPDEGKR+IVEGSDAEIGMSPWQVMLFR+KPVAFSDYIHPVCLPDR+KPVAFSDYIHPVCLPDRETAASLLQAGYK+KSPQELLCGASLISDR+LAACLEGNCAEGLGTNYR+LAVTTHGLPCLAWASAQAK+LKKPVAFSDYIHPVCLPDR+LKKPVAFSDYIHPVCLPDRETAASLLQAGYK+NPDSSTTGPWCYTTDPTVR+QECSIPVCGQDQVTVAMTPR+RGDACEGDSGGPFVMK+RQECSIPVCGQDQVTVAMTPR+SEGSSVNLSPPLEQCVPDR+SEGSSVNLSPPLEQCVPDRGQQYQGR+SPQELLCGASLISDR+SRYPHKPEINSTTHPGADLQENFCR+TATSEYQTFFNPR+TFGSGEADCGLRPLFEK+WVLTAAHCLLYPPWDK+WVLTAAHCLLYPPWDKNFTENDLLVR+YPHKPEINSTTHPGADLQENFCR	27
P00736	1	Complement C1r subcomponent	C1R	705	38.7	CLPVCGKPVNPVEQR+EHEAQSNASLDVFLGHTNVEELMK+ESEQGVYTCTAQGIWK+FCGQLGSPLGNPPGKK+FLEPFDIDDHQQVHCPYDQLQIYANGK+IQYYCHEPYYK+LFGEVTSPLFPKPYPNNFETTTVITVPTGYR+LVFQQFDLEPSEGCFYDYVK+MDVFSQNMFCAGHPSLK+MGNFPWQVFTNIHGR+QGYQLIEGNQVLHSFTAVCQDDGTWHR+QRPPDLDTSSNAVDLLFFTDESGDSR+TLDEFTIIQNLQPQYQFR+VLNYVDWIKK+WILTAAHTIYPK	15
P00739	1	Haptoglobin-related protein	HPR	348	46.8	AVGDKLPECEAVCGKPK+DIAPTLTLYVGK+DIAPTLTLYVGKK+LPECEAVCGKPK+LRTEGDGVYTLNDK+LRTEGDGVYTLNDKK+MVSHHNLTTGATLINEQWLLTTAK+NLFLNHSENATAK+SCAVAEYGVYVK+SPVGVQPILNEHTFCVGMSK+TEGDGVYTLNDKK+VTSIQHWVQK+VVLHPNYHQVDIGLIK+YVMLPVADQYDCITHYEGSTCPK	14
P00740	1	Coagulation factor IX	F9	461	20.8	IIPHHNYNAAINK+ITVVAGEHNIEETEHTEQK+ITVVAGEHNIEETEHTEQKR+VVGGEDAKPGQFPWQVVLNGK+WIVTAAHCVETGVK+YNHDIALLELDEPLVLNSYVTPICIADK	6
P00742	1	Coagulation factor X	F10	488	22.1	CKDGLGEYTCTCLEGFEGK+DGDQCETSPCQNQGK+DWAESTLMTQK+LCSLDNGDCDQFCHEEQNSVVCSCAR+NTEQEEGGEAVHEVEVVIK+QEDACQGDSGGPHVTR+YKDGDQCETSPCQNQGK	7
P00746	1	Complement factor D	CFD	253	40.7	AVPHPDSQPDTIDHDLLLLQLSEK+DVAPGTLCDVAGWGIVNHAGR+GDSGGPLVCGGVLEGVVTSGSR+RPDSLQHVLLPVLDR+VDRDVAPGTLCDVAGWGIVNHAGR+VQVLLGAHSLSQPEPSK+VQVLLGAHSLSQPEPSKR	7

P00747	1	Plasminogen	PLG	810	60.9	AGLEKNYCRNPDGDVGGPWCYTTNPR+APWCHTTNSQVR+ATTVTGTPCQDWAAQEPHR+CQSWSSMTPHR+CSGTEASVVAPPPVVLLPDVETPSEEDCMFGNGK+CTTPPPSSGPTYQCLK+ELRPWCFTTDPNK+ELRPWCFTTDPNKR+EQQCVIMAENR+EQQCVIMAENRK+FGMHFCGGTLISPEWVLTAAHCLEK+FSPATHPSEGLEENYCR+GNVAVTVSGHTCQHWSAQTPHTHNR+HSIFTPETNPR+KCQSWSSMTPHR+KLYDYCDVPQCAAPSFDCGKPQVEPK+KQLGAGSIEECAAK+LYDYCDVPQCAAPSFDCGKPQVEPK+NPDADKGPWCFTTDPSVR+NPDGDVGGPWCYTTNPR+NPDNDPQGPWCYTTDPEK+NPDNDPQGPWCYTTDPEKR+RAPWCHTTNSQVR+RATTVTGTPCQDWAAQEPHR+RWELCDIPR+RYDYCDILECEEECMHCSGENYDGK+TECFITGWGETQGTFGAGLLK+TMSGLECQAWDSQSPHAHGYIPSK+TPENYPNAGLTMNYCR+VILGAHQEVNLEPHVQEIEVSR+VIPACLPSPNYVVADR+VQSTELCAGHLAGGTDSCQGDSGGPLVCFEK+VVGGCVAHPHSWPWQVSLR+WSSTSPHRPR+YDYCDILECEEECMHCSGENYDGK	35
P00748	1	Coagulation factor XII	F12	615	17.2	AEEHTVVLTVTGEPCHFPFQYHR+GRPGPQPWCATTPNFDQDQR+LHEAFSPVSYQHDLALLR+NWGLGGHAFCR+RNHSCEPCQTLAVR+TTLSGAPCQPWASEATYR+YKAEEHTVVLTVTGEPCHFPFQYHR	7
P00751	1	Complement factor B	CFB	764	49.2	AIHCPRPHDFENGEYWPR+ALQAVYSMMSWPDDVPPEGWNR+DLEIEVVLFHPNYNINGK+EAGIPEFYDYDVALIK+EDYLDVYVFGVGPLVNQVNINALASK+FLCTGGVSPYADPNTCR+FLCTGGVSPYADPNTCRGDSGGPLIVHK+FLCTGGVSPYADPNTCRGDSGGPLIVHKR+GDSGGPLIVHKR+GHESCMGAVVSEYFVLTAAHCFTVDDK+HVIILMTDGLHNMGGDPITVIDEIR+IVLDPSGSMNIYLVLDGSDSIGASNFTGAK+IVLDPSGSMNIYLVLDGSDSIGASNFTGAKK+KALQAVYSMMSWPDDVPPEGWNR+KEAGIPEFYDYDVALIK+KIVLDPSGSMNIYLVLDGSDSIGASNFTGAK+LEDSVTYHCSR+LKYGQTIRPICLPCTEGTTR+LLQEGQALEYVCPSGFYPYPVQTR+LPPTTTCQQQK+QLNEINYEDHK+QLNEINYEDHKLK+SPYYNVSDEISFHCYDGYTLR+VKDISEVVTPR+VSEADSSNADWVTK+WSGQTAICDNGAGYCSNPGIPIGTR+YGLVTYATYPK+YGQTIRPICLPCTEGTTR	28
P00915	1	Carbonic anhydrase 1	CA1	261	37.2	EIINVGHSFHVNFEDNDNR+HDTSLKPISVSYNPATAK+LYPIANGNNQSPVDIK+SLLSNVEGDNAVPMQHNNRPTQPLK+TSETKHDTSLKPISVSYNPATAK+YSAELHVAHWNSAK	6
P00918	0.9989	Carbonic anhydrase 2	CA2	260	11.9	QSPVDIDTHTAK+YDPSLKPLSVSYDQATSLR	2
P00995	0.9407	Pancreatic secretory trypsin inhibitor	SPINK1	79	30.4	IYDPVCGTDGNTYPNECVLCFENR	1

P01008	1	Antithrombin-III	SERPINC1	464	57.3	ADGESCSASMMYQEGK+AFLEVNEEGSEAAASTAVVIAGR+ANRPFLVFIR+ATEDEGSEQKIPEATNR+ATEDEGSEQKIPEATNRR+DDLYVSDAFHK+DIPMNPMCIYR+EQLQDMGLVDLFSPEK+EVPLNTIIFMGR+FATTFYQHLADSK+FATTFYQHLADSKNDNDNIFLSPLSISTAFAMTK+FRIEDGFSLK+GDDITMVLILPKPEK+KATEDEGSEQKIPEATNR+LGACNDTLQQLMEVFK+LGACNDTLQQLMEVFKFDTISEK+LVSANRLFGDK+RVAEGTQVLELPFK+RVAEGTQVLELPFKGDDITMVLILPKPEK+SKLPGIVAEGRDDLYVSDAFHK+SLTFNETYQDISELVYGAK+TSDQIHFFFAK+VAEGTQVLELPFK+VAEGTQVLELPFKGDDITMVLILPKPEK	24
P01011	1	Alpha-1-antichymotrypsin	SERPINA3	423	51.3	AKWEMPFDPQDTHQSR+AVLDVFEEGTEASAATAVK+DYNLNDILLQLGIEEAFTSK+FNLTETSEAEIHQSFQHLLR+FNRPFLMIIVPTDTQNIFFMSKVTNPKQA+GTHVDLGLASANVDFAFSLYK+ITLLSALVETR+LYGSEAFATDFQDSAAAK+LYGSEAFATDFQDSAAAKK+MEEVEAMLLPETLK+MEEVEAMLLPETLKR+NLAVSQVVHKAVLDVFEEGTEASAATAVK+RLYGSEAFATDFQDSAAAK+RLYGSEAFATDFQDSAAAKK+TLNQSSDELQLSMGNAMFVK+WEMPFDPQDTHQSR+YTGNASALFILPDQDK	17
P01019	1	Angiotensinogen	AGT	485	28.7	ALQDQLVLVAAK+ANAGKPKDPTFIPAPIQAK+DPTFIPAPIQAK+QPFVQGLALYTPVVLPR+SLDFTELDVAAEK+TGCSLMGASVDSTLAFNTYVHFQGK+VEGLTFQQNSLNWMK+VLSALQAVQGLLVAQGR+VYIHPFHLVIHNESTCEQLAK	9
P01024	1	Complement C3	C3	1663	70.8	[illegible] PGVDYVYK+ADIGCTPGSGKDYAGVFSDAGLTFTSSSGQQTAQR+AEDLVGKSLYVSATVILHSGSDMVQAER+AGDFLEANYMNLQR+AKDQLTCNKFDLK+APSTWLTAYVVK+AVLYNYRQNQELK+AYYENSPQQVFSTEFEVK+DAPDHQELNLDVSLQLPSR+DICEEQVNSLPGSITK+DKNRWEDPGK+DQLTCNKFDLK+DSITTWEILAVSMSDK+DSITTWEILAVSMSDKK+DTWVEHWPEEDECQDEENQK+DTWVEHWPEEDECQDEENQKQCQDLGAFTESMVVFGCPN+DYAGVFSDAGLTFTSSSGQQTAQR+EDIPPADLSDQVPDTESETR+EGVQKEDIPPADLSDQVPDTESETR+EPGQDLVVLPLSITTDFIPSFR+EVVADSVWVDVK+EYVLPSFEVIVEPTEK+FVTVQATFGTQVVEK+FYHPEKEDGK+FYHPEKEDGKLNK+GDQDATMSILDISMMTGFAPDTDDLK+GQGTLSVVTMYHAK+HLIVTPSGCGEQNMIGMTPTVIAVHYLDETEQWEK+HQQTVTIPPKSSLSVPYVIVPLK+IHWESASLLR+ILLQGTPVAQMTEDAVDAER+IPIEDGSGEVVLSR+IPIEDGSGEVVLSRK+IRYYTYLIMNK+KGYTQQLAFR+KGYTQQLAFRQPSSAFAAFVK+KQELSEAEQATR+KSLKVVPEGIR+KVEGTAFVIFGIQDGEQR+KVFLDCCNYITELR+KVLLDGVQNPR+LDKACEPGVDYVYK+LESEETMVLEAHDAQGDVPVTVTVHDFPGK+LESEETMVLEAHDAQGDVPVTVTVHDFPGKK+LKGPLLNKFLTTAK+LKHLIVTPSGCGEQNMI	102

P01031	1	Complement C5	C5	1676	35.8	AFTECCVVASQLR+ALLVGEHLNIIVTPK+ALVEGVDQLFTDYQIK+CCYDGACVNNDETCEQR+DGHVILQLNSIPSSDFLCVR+DSLDQLVGGVPVTLNAQTIDVNQETSDLDPSK+DVFLEMNIPYSVVR+EESSSGSSHAVMDISLPTGISANEEDLK+EKFSDASYQSINIPVTQNMVPSSR+ELSYYSLEDLNNK+EYVLPHFSVSIEPEYNFIGYK+FQNSAILTIQPK+FSDASYQSINIPVTQNMVPSSR+FSYSSGHVHLSSENK+GGSASTWLTAFALR+IDTQDIEASHYR+ITHYNYLILSK+IVACASYKPSR+KCCYDGACVNNDETCEQR+KFSYSSGHVHLSSENK+KQTACKPEIAYAYK+KVTCTNAELVK+LKEGMLSIMSYR+LNLVATPLFLKPGIPYPIK+MPITYDNGFLFIHTDKPVYTPDQSVK+MSAVEGICTSESPVIDHQGTK+MVETTAYALLTSLNLK+QLPGGQNPVSYVYLEVVSK+QTACKPEIAYAYK+RESYSGVTLDPR+RMPITYDNGFLFIHTDKPVYTPDQSVK+SDLGCGAGGGLNNANVFHLAGLTFLTNANADDSQENDEPCK+SYFPESWLWEVHLVPR+TDAPDLPEENQAR+TLLPVSKPEIR+TSTSEEVCSFYLK+VDDGVASFVLNLPSGVTVLEFNVK+VFKDVFLEMNIPYSVVR+VRVYSLNDDLKPAKR+VYSLNDDLKPAK+VYSLNDDLKPAKR+YKEDFSTTGTAYFEVK+YVLSPYKLNLVATPLFLKPGIPYPIK	43
P01034	1	Cystatin-C	CST3	146	23.3	LVGGPMDASVEEEGVRR+TQPNLDNCPFHDQPHLK	2
P01042	1	Kininogen-1	KNG1	644	43.5	AATGECTATVGKR+DIPTNSPELEETLTHTITK+ENFLFLTPDCK+ESNEELTESCETKK+FKLDDDLEHQGGHVLDHGHK+FSVATQTCQITPAEGPVVTAQYDCLGCVHPISTQSPDLEPILR+GHGLGHGHEQQHGLGHGHK+HGIQYFNNNTQHSSLFMLNEVK+IASFSQNCDIYPGK+IASFSQNCDIYPGKDFVQPPTK+ITYSIVQTNCSK+IYPTVNCQPLGMISLMK+KIYPTVNCQPLGMISLMK+KLGQSLDCNAEVYVVPWEK+KYNSQNQSNNQFVLYR+LDDDLEHQGGHVLDHGHK+LGQSLDCNAEVYVVPWEK+LGQSLDCNAEVYVVPWEKK+SLWNGDTGECTDNAYIDIQLR+TWQDCEYKDAAK+YNSQNQSNNQFVLYR	21
P01344	0.9407	Insulin-like growth factor II	IGF2	180	8.9	SCDLALLETYCATPAK	1
P01833	1	Polymeric immunoglobulin receptor	PIGR	764	4.8	GSVTFHCALGPEVANVAK+NADLQVLKPEPELVYEDLR	2
P01889	0.9998	HLA class I histocompatibility antigen, B-7 alpha chain	HLA-B	362	9.9	DGEDQTQDTELVETRPAGDR+THVTHHPISDHEATLR	2
P01891	1	HLA class I histocompatibility antigen, A-68 alpha chain	HLA-A	365	11	DGEDQTQDTELVETRPAGDGTFQK+THMTHHAVSDHEATLR	2
P02042	1	Hemoglobin subunit delta	HBD	147	49.7	FFESFGDLSSPDAVMGNPK+GTFSQLSELHCDK+KVLGAFSDGLAHLDNLK+LLVVYPWTQR+VLGAFSDGLAHLDNLK+VVAGVANALAHKYH	6
P02649	1	Apolipoprotein E	APOE	317	43.2	AATVGSLAGQPLQER+AKLEEQAQQIR+AYKSELEEQLTPVAEETR+GEVQAMLGQSTEELR+LDEVKEQVAEVR+SELEEQLTPVAEETR+SWFEPLVEDMQR+VEQAVETEPEPELR+VQAAVGTSAAPVPSDNH+WVQTLSEQVQEELLSSQVTQELR	10
P02654	1	Apolipoprotein C-I	APOC1	83	26.5	LKEFGNTLEDK+MREWFSETFQK	2
P02655	0.9407	Apolipoprotein C-II	APOC2	101	20.8	STAAMSTYTGIFTDQVLSVLK	1

P02656	1	Apolipoprotein C-III	APOC3	99	37.4	DALSSVQESQVAQQAR+DKFSEFWDLDPEVRPTSAVAA+FSEFWDLDPEVRPTSAVAA	3
P02730	1	Band 3 anion transport protein	SLC4A1	911	10.6	GLDLNGGPDDPLQQTGQLFGGLVR+HSHAGELEALGGVKPAVLTR+SGDPSQPLLPQHSSLETQLFCEQGDGGTEGHSPSGILEK+YQSSPAKPDSSFYK	4
P02741	0.9407	C-reactive protein	CRP	224	5.4	RQDNEILIFWSK	1
P02743	1	Serum amyloid P-component	APCS	223	21.5	AYSLFSYNTQGR+DNELLVYKER+ERVGEYSLYIGR+IVLGQEQDSYGGKFDR	4
P02745	1	Complement C1q subcomponent subunit A	C1QA	245	28.2	DQPRPAFSAIR+GLFQVVSGGMVLQLQQGDQVWVEK+KGHIYQGSEADSVFSGFLIFPSA+RSLGFCDTTNK	4
P02746	1	Complement C1q subcomponent subunit B	C1QB	253	22.1	DQTIRFDHVITNMNNNYEPR+FDHVITNMNNNYEPR+LEQGENVFLQATDK+VVTFCDYAYNTFQVTTGGMVLK	4
P02747	1	Complement C1q subcomponent subunit C	C1QC	245	12.7	FNAVLTNPQGDYDTSTGK+QTHQPPAPNSLIR	2
P02748	1	Complement component C9	C9	559	43.6	AEQCCEETASSISLHGK+AIEDYINEFSVR+CLCACPFKFEGIACEISK+CNGDNDCGDFSDEDDCESEPRPPCR+CTDAVGDRRQCVPTEPCEDAEDDCGNDFQCSTGR+DRDGNTLTYYR+DRVVEESELAR+DVVLTTTFVDDIK+FTPTETNKAEQCCEETASSISLHGK+GTVIDVTDFVNWASSINDAPVLISQK+KCHTCQNGGTVILMDGK+LRCNGDNDCGDFSDEDDCESEPRPPCR+LSPIYNLVPVK+NRDVVLTTTFVDDIK+QCVPTEPCEDAEDDCGNDFQCSTGR+RPWNVASLIYETK+RQCVPTEPCEDAEDDCGNDFQCSTGR+SIEVFGQFNGKR+TEHYEEQIEAFK	19
P02749	1	Beta-2-glycoprotein 1	APOH	345	67.2	ATFGCHDGYSLDGPEEIECTK+CPFPSRPDNGFVNYPAKPTLYYK+CSYTEDAQCIDGTIEVPK+CTEEGKWSPELPVCAPIICPPPSIPTFATLR+DKATFGCHDGYSLDGPEEIECTK+DTAVFECLPQHAMFGNDTITCTTHGNWTK+KCSYTEDAQCIDGTIEVPK+KFICPLTGLWPINTLK+TCPKPDDLPFSTVVPLK+TFYEPGEEITYSCKPGYVSR+VCPFAGILENGAVR+VYKPSAGNNSLYR+WSPELPVCAPIICPPPSIPTFATLR+YTTFEYPNTISFSCNTGFYLNGADSAK	14
P02750	1	Leucine-rich alpha-2-glycoprotein	LRG1	347	37.8	DGFDISGNPWICDQNLSDLYR+ENQLEVLEVSWLHGLK+KLPPGLLANFTLLR+LHLEGNKLQVLGK+LQELHLSSNGLESLSPEFLRPVPQLR+NALTGLPPGLFQASATLDTLVLK+TLDLGENQLETLPPDLLR	7

P02751	1	Fibronectin	FN1	2386	34.4	[illegible]IGDSWEK+DDKESVPISDTIIPAVPPPTDLR+DQCIVDDITYNVNDTFHK+DTLTSRPAQGVVTTLENVSPPR+EATIPGHLNSYTIK+EESPLLIGQQSTVSDVPR+EINLAPDSSSVVVSGLMVATK+ESKPLTAQQTTK+ESVPISDTIIPAVPPPTDLR+FGFCPMAAHEEICTTNEGVMYR+FLATTPNSLLVSWQPPR+FTQVTPTSLSAQWTPPNVQLTGYR+GDSPASSKPISINYR+GEWTCKPIAEK+GFNCESKPEAEETCFDK+GLKPGVVYEGQLISIQQYGHQEVTR+HHPEHFSGRPR+HTSVQTTSSGSGPFTDVR+ITYGETGGNSPVQEFTVPGSK+KKTDELPQLVTLPHPNLHGPEILDVPSTVQK+LDAPTNLQFVNETDSTVLVR+LGVRPSQGGEAPR+LLCQCLGFGSGHFR+NLQPASEYTVSLVAIK+NSITLTNLTPGTEYVVSIVALNGR+NTFAEVTGLSPGVTYYFK+QDGHLWCSTTSNYEQDQK+RHEEGHMLNCTCFGQGR+RPGGEPSPEGTTGQSYNQYSQR+SSPVVIDASTAIDAPSNLR+TEIDKPSQMQVTDVQDNSISVK+TETITGFQVDAVPANGQTPIQR+TKTETITGFQVDAVPANGQTPIQR+TPFVTHPGYDTGNGIQLPGTSGQQPSVGQQMIFEEHGFR+TYHVGEQWQK+TYLGNALVCTCYGGSR+VDVIPVNLPGEHGQR+VEYELSEEGDEPQYLDLPSTATSVNIPDLLPGR+VPGTSTSATLTGLTR+VTIMWTPPESAVTGYR+VTWAPPPSIDLTNFLVR+VVTPLSPPTNLHLEANPDTGVLTVSWER+WKEATIPGHLNSYTIK+WSRPQA	46
P02753	1	Retinol-binding protein 4	RBP4	201	56.2	GNDDHWIVDTDYDTYAVQYSCR+KDPEGLFLQDNIVAEFSVDETGQMSATAK+LIVHNGYCDGR+LLNLDGTCADSYSFVFSR+LLNNWDVCADMVGTFTDTEDPAK+QRQEELCLAR	6
P02760	1	Protein AMBP	AMBP	352	38.6	AFIQLWAFDAVK+EDSCQLGYSAGPCMGMTSR+EYCGVPGDGDEELLR+GECVPGEQEPEPILIPR+GVCEETSGAYEKTDTDGK+KEDSCQLGYSAGPCMGMTSR+KGVCEETSGAYEK+KGVCEETSGAYEKTDTDGK+MTVSTLVLGEGATEAEISMTSTR+TVAACNLPIVR+VVAQGVGIPEDSIFTMADR	11
P02765	1	Alpha-2-HS-glycoprotein	AHSG	367	46.6	AALAAFNAQNNGSNFQLEEISR+AQLVPLPPSTYVEFTVSGTDCVAK+CDSSPDSAEDVRK+EHAVEGDCDFQLLK+HTFMGVVSLGSPSGEVSHPR+HTLNQIDEVK+KVCQDCPLLAPLNDTR+TVVQPSVGAAAGPVVPPCPGR+VCQDCPLLAPLNDTR+VWPQQPSGELFEIEIDTLETTCHVLDPTPVAR	10
P02766	1	Transthyretin	TTR	147	73.5	AADDTWEPFASGK+ALGISPFHEHAEVVFTANDSGPR+ALGISPFHEHAEVVFTANDSGPRR+GSPAINVAVHVFR+GSPAINVAVHVFRK+GSPAINVAVHVFRKAADDTWEPFASGK+KAADDTWEPFASGK+RYTIAALLSPYSYSTTAVVTNPK+RYTIAALLSPYSYSTTAVVTNPKE+TSESGELHGLTTEEEFVEGIYK+TSESGELHGLTTEEEFVEGIYKVEIDTK+VLDAVRGSPAINVAVHVFRK+YTIAALLSPYSYSTTAVVTNPK+YTIAALLSPYSYSTTAVVTNPKE	14

P02774	1	Vitamin D-binding protein	GC	474	60.8	EDFTSLSLVLYSR+ELSSFIDKGQELCADYSENTFTEYK+ELSSFIDKGQELCADYSENTFTEYKK+EVVSLTEACCAEGADPDCYDTR+FPSGTFEQVSQLVK+GQELCADYSENTFTEYK+GQELCADYSENTFTEYKK+HLSLLTTLSNR+HQPQEFPTYVEPTNDEICEAFR+HQPQEFPTYVEPTNDEICEAFRK+KFPSGTFEQVSQLVK+LCDNLSTKNSK+NSKFEDCCQEK+RSDFASNCCSINSPPLYCDSEIDAELK+RTHLPEVFLSK+SCESNSPFPVHPGTAECCTK+SDFASNCCSINSPPLYCDSEIDAELK+SLGECCDVEDSTTCFNAK+SYLSMVGSCCTSASPTVCFLK+TAMDVFVCTYFMPAAQLPELPDVELPTNK+VMDKYTFELSR+VPTADLEDVLPLAEDITNILSK	22
P02775	1	Platelet basic protein	PPBP	128	22.7	EESLDSDLYAELR+GKEESLDSDLYAELR+GTHCNQVEVIATLK	3
P02776	1	Platelet factor 4	PF4	101	26.7	AGPHCPTAQLIATLK+ICLDLQAPLYK+KICLDLQAPLYK	3
P02790	1	Hemopexin	HPX	462	69.7	ALPQPQNVTSLLGCTH+CSDGWSFDATTLDDNGTMLFFK+CSPHLVLSALTSDNHGATYAFSGTHYWR+DGWHSWPIAHQWPQGPSAVDAAFSWEEK+EVGTPHGIILDSVDAAFICPGSSR+EWFWDLATGTMK+FDPVRGEVPPR+FDPVRGEVPPRYPR+GDKVWVYPPEK+GDKVWVYPPEKK+GECQAEGVLFFQGDR+GHGHRNGTGHGNSTHHGPEYMR+LLQDEFPGIPSPLDAAVECHR+LLQDEFPGIPSPLDAAVECHRGECQAEGVLFFQGDR+LYLVQGTQVYVFLTK+NFPSPVDAAFR+NFPSPVDAAFRQGHNSVFLIK+NGTGHGNSTHHGPEYMR+SGAQATWTELPWPHEK+SLGPNSCSANGPGLYLIHGPNLYCYSDVEK+SWPAVGNCSSALR+WKNFPSPVDAAFR+YYCFQGNQFLR	23
P02795	0.9407	Metallothionein-2	MT2A	61	34.4	KSCCSCCPVGCAKCAQGCICK	1
P03372	0.9407	Estrogen receptor	ESR1	595	5.9	GSMAMESAKETRYCAVCNDYASGYHYGVWSCEGCK	1
P03950	1	Angiogenin	ANG	147	24.5	NVVVACENGLPVHLDQSIFR+YTHFLTQHYDAKPQGR	2
P03951	1	Coagulation factor XI	F11	625	15	CTDDVHCHFFTYATR+DACKGDSGGPLSCK+EDTSFFGVQEIIIHDQYK+GEWPWQVTLHTTSPTQR+LETTVNYTDSQRPICLPSK+YCQVVCTYHPR	6
P03952	1	Plasma kallikrein	KLKB1	638	32	CLLFSFLPASSINDMEK+CQFFSYATQTFHK+CQFFTYSLLPEDCK+DACKGDSGGPLVCK+EGGKDACKGDSGGPLVCK+EKGEIQNILQK+IVGGTNSSWGEWPWQVSLQVK+IYPGVDFGGEELNVTFVK+LQAPLNYTEFQKPICLPSK+QCGHQISACHR+RTLPEPCHSK+TSESGTPSSSTPQENTISGYSLLTCK+VNIPLVTNEECQK+VNIPLVTNEECQKR+VSEGNHDIALIK	15
P03973	0.9407	Antileukoproteinase	SLPI	132	11.4	YKKPECQSDWQCPGK	1

P04003	1	C4b-binding protein alpha chain	C4BPA	597	49.9	CHPGYKPTTDEPTTVICQK+CRHPGELRNGQVEIK+DQYVEPENVTIQCDSGYGVVGPQSITCSGNR+EDVYVVGTVLR+FSAICQGDGTWSPR+FSLLGHASISCTVENETIGVWRPSPPTCEK+GSSVIHCDADSK+GVGWSHPLPQCEIVK+KPDVSHGEMVSGFGPIYNYK+LSCSYSHWSAPAPQCK+LSLEIEQLELQR+LSVDKDQYVEPENVTIQCDSGYGVVGPQSITCSGNR+SHSTQTLTCNSDGEWVYNTFCIYK+SRPANHCVYFYGDEISFSCHETSR+WNPSPPACEPNSCINLPDIPHASWETYPRPTK+WTPYQGCEALCCPEPK+YRCHPGYKPTTDEPTTVICQK	17
P04004	1	Vitronectin	VTN	478	42.5	CQCDELCSYYQSCCTDYTAECKPQVTR+CTEGFNVDKK+DVWGIEGPIDAAFTR+DWHGVPGQVDAAMAGR+FEDGVLDPDYPR+IYISGMAPRPSLAK+KCQCDELCSYYQSCCTDYTAECKPQVTR+NISDGFDGIPDNVDAALALPAHSYSGR+NNATVHEQVGGPSLTSDLQAQSK+QYWEYQFQHQPSQEECEGSSLSAVFEHFAMMQR+RVDTVDPPYPR+SIAQYWLGCPAPGHL	12
P04040	1	Catalase	CAT	527	12.7	DPILFPSFIHSQK+GAGAFGYFEVTHDITK+GPLLVQDVVFTDEMAHFDR+NFTEVHPDYGSHIQALLDK	4
P04066	0.9407	Tissue alpha-L-fucosidase	FUCA1	466	3.6	FFHPEEWADLFQAAGAK	1
P04070	1	Vitamin K-dependent protein C	PROC	461	30.8	ELNQAGQETLVTGWGYHSSR+EVFVHPNYSK+EVSFLNCSLDNGGCTHYCLEEVGWR+LACGAVLIHPSWVLTAAHCMDESKK+LGDDLLQCHPAVK+RGDSPWQVVLLDSK+STTDNDIALLHLAQPATLSQTIVPICLPDSGLAER	7
P04075	1	Fructose-bisphosphate aldolase A	ALDOA	364	26.9	ADDGRPFPQVIK+FSHEEIAMATVTALR+GVVPLAGTNGETTTQGLDGLSER+IGEHTPSALAIMENANVLAR+YASICQQNGIVPIVEPEILPDGDHDLK+YASICQQNGIVPIVEPEILPDGDHDLKR	6
P04114	1	Apolipoprotein B-100	APOB	4563	52.3	[illegible] NVQGSGETTYDHK+AEFTGRHDAHLNGK+AEPLAFTFSHDYK+AEPLAFTFSHDYKGSTSHHLVSR+AEPLAFTFSHDYKGSTSHHLVSRK+AHLDIAGSLEGHLR+ALVEQGFTVPEIK+ALYWVNGQVPDGVSK+ANLFNKLVTELR+AQNLYQELLTQEGQASFQGLK+ASGSLPYTQTLQDHLNSLK+ATFQTPDFIVPLTDLR+ATGVLYDYVNK+ATLELSPWQMSALVQVHASQPSSFHDFPDLGQEVALNANTK+ATLYALSHAVNNYHK+ATVAVYLESLQDTK+AVSMPSFSILGSDVR+CVQSTKPSLMIQK+DAVEKPQEFTIVAFVK+DDKHEQDMVNGIMLSVEK+DEPTYILNIK+DEPTYILNIKR+DFSAEYEEDGKYEGLQEWEGK+DKAQNLYQELLTQEGQASFQGLK+DKDQEVLLQTFLDDASPGDK+DKDQEVLLQTFLDDASPGDKR+DLKVEDIPLAR+DPNTGRLNGESNLR+DQEVLLQTFLDDASPGDK+DQEVLLQTFLDDASPGDKR+EALKESQLPTVMDFR+EALKESQLPTVMDFRK+EEYFDPSIVGWTVK+EFNLQNMGLPDFHIPENLFLK+EFQVPTFTIPK+ENFAGEATLQR+EQHLFLPFSYK+ESQLPTVMDFRK+EVGTVLSQVYSK+EYSGTIASEANTYLNSK+FDHTNSLNIAGLSLDFSSK+FEVDSPVYNATWSASLK+FFSLLSGSLNSHGLELNADILGTDK+FNSSYLQGTNQITGR+FQFPGKPGIYTR+FSDEGTHESQISFTIEGPLTSFGLSNK+FSVPAGIVIPSFQALTAR+FVEGSHNSTVSLTTK+GAVDHKLSLESLTSYFSIESSTKGDVK+GAY	193
P04179	0.9991	Superoxide dismutase [Mn], mitochondrial	SOD2	222	13.1	AIWNVINWENVTER+HHAAYVNNLNVTEEK	2

P04180	1	Phosphatidylcholine-sterol acyltransferase	LCAT	440	11.8	AELSNHTRPVILVPGCLGNQLEAK+TYIYDHGFPYTDPVGVLYEDGDDTVATR	2
P04196	1	Histidine-rich glycoprotein	HRG	525	52.4	ADLFYDVEALDLESPK+DHHHPHKPHEHGPPPPPDER+DHSHGPPLPQGPPPLLPMSCSSCQHATFGTNGAQR+DSPVLIDFFEDTER+GEVLPLPEANFPSFPLPHHK+GGEGTGYFVDFSVR+HPLKPDNQPFPQSVSESCPGK+HSHNNNSSDLHPHK+KGEVLPLPEANFPSFPLPHHK+KYWNDCEPPDSR+RDGYLFQLLR+RPSEIVIGQCK+SGFPQVSMFFTHTFPK+SSTTKPPFKPHGSR+VENTTVYYLVLDVQESDCSVLSR+VIDFNCTTSSVSSALANTK+YKEENDDFASFR+YKEENDDFASFRVDR	18
P04217	1	Alpha-1B-glycoprotein	A1BG	495	61.6	CEGPIPDVTFELLR+EGDHEFLEVPEAQEDVEATFPVHQPGNYSCSYR+FQSPAGTEALFELHNISVADSANYSCVYVDLKPPFGGSAPSER+HQFLLTGDTQGR+IFFHLNAVALGDGGHYTCR+LELHVDGPPPRPQLR+LHDNQNGWSGDSAPVELILSDETLPAPEFSPEPESGR+NGVAQEPVHLDSPAIK+NGVAQEPVHLDSPAIKHQFLLTGDTQGR+REGDHEFLEVPEAQEDVEATFPVHQPGNYSCSYR+SGLSTGWTQLSK+SLPAPWLSMAPVSWITPGLK+TDGEGALSEPSATVTIEELAAPPPPVLMHHGESSQVLHPGNK+TPGAAANLELIFVGPQHAGNYR+VTLTCVAPLSGVDFQLR+YRLHDNQNGWSGDSAPVELILSDETLPAPEFSPEPESGR	16
P04275	1	von Willebrand factor	VWF	2813	24.6	AHLLSLVDVMQR+AMYSIDINDVQDQCSCCSPTR+DETHFEVVESGR+DETLQDGCDTHFCK+EGGPSQIGDALGFAVR+EQAPNLVYMVTGNPASDEIKR+EQDLEVILHNGACSPGAR+FSEEACAVLTSPTFEACHR+GLQPTLTNPGECRPNFTCACR+GLRPSCPNSQSPVK+HCDGNVSSCGDHPSEGCFCPPDK+HIVTFDGQNFK+IEDLPTMVTLGNSFLHK+ILDELLQTCVDPEDCPVCEVAGR+IPGTCCDTCEEPECNDITAR+KTTCNPCPLGYK+KVPLDSSPATCHNNIMK+LHGDCQDLQK+LPGDIQVVPIGVGPNANVQELER+NSQWICSNEECPGECLVTGQSHFK+NVSCPQLEVPVCPSGFQLSCK+QNADQCCPEYECVCDPVSCDLPPVPHCER+RDETLQDGCDTHFCK+RPGDVWTLPDQCHTVTCQPDGQTLLK+RVSPPSCPPHR+RVTGCPPFDEHK+SGFTYVLHEGECCGR+SKEFMEEVIQR+STIYPVGQFWEEGCDVCTCTDMEDAVMGLR+SVGSQWASPENPCLINECVR+TCAQEGMVLYGWTDHSACSPVCPAGMEYR+TCQSLHINEMCQER+TEPMQVALHCTNGSVVYHEVLNAMECK+VAQCSQKPCEDSCR+VKEEVFIQQR+VPLDSSPATCHNNIMK+VTGCPPFDEHK+YLFPGECQYVLVQDYCGSNPGTFR+YLTSEMHGARPGASK+YNSCAPACQVTCQHPEPLACPVQCVEGCHAHCPPGK	40
P04278	1	Sex hormone-binding globulin	SHBG	402	28.1	DGRPEIQLHNHWAQLTVGAGPR+DIPQPHAEPWAFSLDLGLK+QAAGSGHLLALGTPENPSWLSLHLQDQK+SCDVESNPGIFLPPGTQAEFNLR+SHEIWTHSCPQSPGNGTDASH	5
P04279	1	Semenogelin-1	SEMG1	462	7.4	EQDLLSHEQK+HLGGSQQLLHNK+VQTSLCPAHQDK	3

P04406	1	Glyceraldehyde-3-phosphate dehydrogenase	GAPDH	335	27.2	GILGYTEHQVVSSDFNSDTHSSTFDAGAGIALNDHFVK+VIISAPSADAPMFVMGVNHEK+VVDLMAHMASK+WGDAGAEYVVESTGVFTTMEK	4
P04745	0.9407	Alpha-amylase 1	AMY1A	511	2.3	ALVFVDNHDNQR	1
P04792	0.9407	Heat shock protein beta-1	HSPB1	205	7.8	VSLDVNHFAPDELTVK	1
P05062	1	Fructose-bisphosphate aldolase B	ALDOB	364	8.5	IADQCPSSLAIQENANALAR+IKVENTEENR+IKVENTEENRR	3
P05090	1	Apolipoprotein D	APOD	189	20.1	ADGTVNQIEGEATPVNLTEPAK+ADGTVNQIEGEATPVNLTEPAKLEVK+KMTVTDQVNCPK	3
P05106	1	Integrin beta-3	ITGB3	788	7.9	CECGSCVCIQPGSYGDTCEK+GECLCGQCVCHSSDFGK+HVLTLTDQVTR+NDASHLLVFTTDAK	4
P05109	1	Protein S100-A8	S100A8	93	24.7	ALNSIIDVYHK+KLLETECPQYIR+LLETECPQYIR	3
P05154	1	Plasma serine protease inhibitor	SERPINA5	406	10.8	EDQYHYLLDR+GFQQLLQELNQPR+VVGVPYQGNATALFILPSEGK	3
P05155	1	Plasma protease C1 inhibitor	SERPING1	500	35.2	DFTCVHQALK+DFTCVHQALKGFTTK+GVTSVSQIFHSPDLAIR+HRLEDMEQALSPSVFK+IKVTTSQDMLSIMEK+KYPVAHFIDQTLK+LEDMEQALSPSVFK+LVLLNAIYLSAK+MLFVEPILEVSSLPTTNSTTNSATK+NSVIKVPMMNSK+TNLESILSYPK+TNLESILSYPKDFTCVHQALK+VGQLQLSHNLSLVILVPQNLK+VLSNNSDANLELINTWVAK+VPMMNSKKYPVAHFIDQTLK+VTTSQDMLSIMEK+YPVAHFIDQTLK	17
P05156	1	Complement factor I	CFI	583	32.1	ACDGINDCGDQSDELCCK+ADSPMDDFFQCVNGK+AQLGDLPWQVAIK+EANVACLDLGFQQGADTQR+EANVACLDLGFQQGADTQRR+GLETSLAECTFTK+HGNTDSEGIVEVK+IIFHENYNAGTYQNDIALIEMK+LSDLSINSTECLHVHCR+RAQLGDLPWQVAIK+RSFPTYCQQK+TMGYQDFADVVCYTQK+VTYTSQEDLVEKK+YQIWTTVVDWIHPDLK	14
P05160	1	Coagulation factor XIII B chain	F13B	661	10.9	KEHETCLAPELYNGNYSTTQK+SGYLLHGSNEITCNR+TTGGKDEEVVQCLSDGWSSQPTCR+VLHGDLIDFVCK	4
P05362	0.9352	Intercellular adhesion molecule 1	ICAM1	532	1.9	DHHGANFSCR	1
P05452	1	Tetranectin	CLEC3B	202	30.2	GGTLGTPQTGSENDALYEYLR+LDTLAQEVALLK+NWETEITAQPDGGK+SRLDTLAQEVALLK+TFHEASEDCISR	5
P05543	1	Thyroxine-binding globulin	SERPINA7	415	35.9	AQWANPFDPSKTEDSSSFLIDK+EGQMESVEAAMSSK+GTEAAAVPEVELSDQPENTFLHPIIQIDR+KELELQIGNALFIGK+MGIQHAYSENADFSGLTEDNGLK+QEINSHVEMQTK+TLYETEVFSTDFSNISAAK+VTACHSSQPNATLYK	8
P05546	1	Heparin cofactor 2	SERPIND1	499	31.7	ENTVTNDWIPEGEEDDDYLDLEK+EYYFAEAQIADFSDPAFISK+FPVEMTHNHNFR+FTVDRPFLFLIYEHR+GETHEQVHSILHFK+GGETAQSADPQWEQLNNK+GGETAQSADPQWEQLNNKNLSMPLLPADFHK+HQGTITVNEEGTQATTVTTVGFMPLSTQVR+NLSMPLLPADFHK+VREYYFAEAQIADFSDPAFISK+YEITTIHNLFR	11
P05556	0.9407	Integrin beta-1	ITGB1	798	2	DKLPQPVQPDPVSHCK	1
P06276	1	Cholinesterase	BCHE	602	15.1	AILQSGSFNAPWAVTSLYEAR+EALGDVVGDYNFICPALEFTK+ESILFHYTDWVDDQRPENYR+SVTLFGESAGAASVSLHLLSPGSHSLFTR	4

P06396	1	Gelsolin	GSN	782	39.4	AGKEPGLQIWR+AQPVQVAEGSEPDGFWEALGGK+ATEVPVSWESFNNGDCFILDLGNNIHQWCGSNSNR+DPDQTDGLGLSYLSSHIANVER+EPAHLMSLFGGKPMIIYK+EVQGFESATFLGYFK+HVVPNEVVVQR+NWRDPDQTDGLGLSYLSSHIANVER+QTQVSVLPEGGETPLFK+RYIETDPANR+SEDCFILDHGK+SEDCFILDHGKDGK+TPSAAYLWVGTGASEAEK+VHVSEEGTEPEAMLQVLGPKPALPAGTEDTAK+VHVSEEGTEPEAMLQVLGPKPALPAGTEDTAKEDAANR+VHVSEEGTEPEAMLQVLGPKPALPAGTEDTAKEDAANRK+VPEARPNSMVVEHPEFLK+VPFDAATLHTSTAMAAQHGMDDDGTGQK+VSNGAGTMSVSLVADENPFAQGALK+YIETDPANRDR	20
P06454	0.9407	Prothymosin alpha	PTMA	111	12.6	AAEDDEDDDVDTKK	1
P06681	1	Complement C2	C2	752	32.7	ALHQVFEHMLDVSK+DMTEVISSLENANYK+ECQGNGVWSGTEPICR+EVVTDQFLCSGTQEDESPCK+EVVTDQFLCSGTQEDESPCKGESGGAVFLER+GALISDQWVLTAAHCFR+KNQGILEFYGDDIALLK+LGSYPVGGNVSFECEDGFILR+LLGMETMAWQEIR+NDYLDIYAIGVGK+NQGILEFYGDDIALLK+QCRPNGMWDGETAVCDNGAGHCPNPGISLGAVR+QPYSYDFPEDVAPALGTSFSHMLGATNPTQK+QSVPAHFVALNGSK+RNDYLDIYAIGVGK+TPWHVTIKPK	16
P06702	1	Protein S100-A9	S100A9	114	56.1	LGHPDTLNQGEFK+MHEGDEGPGHHHKPGLGEGTP+NIETIINTFHQYSVK+VIEHIMEDLDTNADK	4
P06727	1	Apolipoprotein A-IV	APOA4	396	61.4	AKIDQNVEELK+ALVQQMEQLR+DKVNSFFSTFK+EEIGKELEELR+ENADSLQASLRPHADELK+IDQNVEELKGR+IDQTVEELRR+KLVPFATELHER+LGEVNTYAGDLQK+LGPHAGDVEGHLSFLEK+LKEEIGKELEELR+LLPHANEVSQK+LNHQLEGLTFQMK+LVPFATELHER+RVEPYGENFNK+RSLAPYAQDTQEK+RVEPYGENFNK+SELTQQLNALFQDK+SLAELGGHLDQQVEEFR+SLAELGGHLDQQVEEFRR+TLSLPELEQQQEQQQEQQQEQVQMLAPLES+TQVNTQAEQLR+TQVNTQAEQLRR+VKIDQTVEELR+VKIDQTVEELRR+VLRENADSLQASLRPHADELK	26
P06733	0.9407	Alpha-enolase	ENO1	434	4.8	DYPVVSIEDPFDQDDWGAWQK	1
P07195	1	L-lactate dehydrogenase B chain	LDHB	334	17.1	GEMMDLQHGSLFLQTPK+IVADKDYSVTANSK+LKDDEVAQLKK+SLADELALVDVLEDK	4
P07225	1	Vitamin K-dependent protein S	PROS1	676	32.5	ASFTCTCKPGWQGEK+DCKDVDECSLKPSICGTAVCK+DVDECSLKPSICGTAVCK+EAVMDINKPGPLFKPENGLLETK+IETISHEDLQR+IQALSLCSDQQSHLEFR+ITTGGDVINNGLWNMVSVEELEHSISIK+KVESELIKPINPR+SCEVVSVCLPLNLDTK+SCVNAIPDQCSPLPCNEDGYMSCK+SQDILLSVENTVIYR+VESELIKPINPR+YNLKSKSCEDIDECSENMCAQLCVNYPGGYTCYCDGK	13
P07237	0.9999	Protein disulfide-isomerase	P4HB	508	7.3	QFLQAAEAIDDIPFGITSNSDVFSK+YKPESEELTAER	2
P07333	1	Macrophage colony-stimulating factor 1 receptor	CSF1R	972	7.8	VLTLNLDQVDFQHAGNYSCVASNVQGK+VMVEAYPGLQGFNWTYLGPFSDHQPEPK+VTVQSLLTVETLEHNQTYECR	3
P07355	0.9407	Annexin A2	ANXA2	339	5.3	RAEDGSVIDYELIDQDAR	1

P07357	1	Complement component C8 alpha chain	C8A	584	32.2	AIDEDCSQYEPIPGSQK+ALDQYLMEFNACR+CGPCFNNGVPILEGTSCR+ECDNPAPQNGGASCPGR+ECDNPAPQNGGASCPGRK+FGGTICSGDIWDQASCSSSTTCVR+HLVCNGDQDCLDGSDEDDCEDVR+INVGGGLSGDHCK+INVGGGLSGDHCKK+RECDNPAPQNGGASCPGR+RECDNPAPQNGGASCPGRK+RHLVCNGDQDCLDGSDEDDCEDVR+VRGGSSGWSGGLAQNR+YHFEALADTGISSEFYDNANDLLSK+YNPVVIDFEMQPIHEVLR	15
P07358	1	Complement component C8 beta chain	C8B	591	38.7	CDCICPVGSQGLACEVSYR+DTMVEDLVVLVR+EVEDCVTNRPCR+EVSSCHCAPCQGNGVPVLK+FRKPYNVESYTPQTQGK+GDYTLNNVHACAK+GGASEHITTLAYQELPTADLMQEWGDAVQYNPAIIK+KPYNVESYTPQTQGK+LLCNGDNDCGDQSDEANCR+LLCNGDNDCGDQSDEANCRR+RLLCNGDNDCGDQSDEANCR+RLPLEYSYGEYR+SLMLHYEFLQR+VKVEPLYELVTATDFAYSSTVR+YAYLLQPSQFHGEPCNFSDK+YAYLLQPSQFHGEPCNFSDKEVEDCVTNRPCR+YYAGGCSPHYILNTR	17
P07359	1	Platelet glycoprotein Ib alpha chain	GP1BA	652	5.1	AMTSNVASVQCDNSDKFPVYK+VASHLEVNCDK+VASHLEVNCDKR	3
P07360	1	Complement component C8 gamma chain	C8G	202	45.5	AEATTLHVAPQGTAMAVSTFR+KLDGICWQVR+RPASPISTIQPK+SLPVSDSVLSGFEQR+VQEAHLTEDQIFYFPK+YGFCEAADQFHVLDEVR+YGFCEAADQFHVLDEVRR	7
P07437	0.9407	Tubulin beta chain	TUBB	444	4.1	GHYTEGAELVDSVLDVVR	1
P07476	0.9407	Involucrin	IVL	585	2.2	HLEHPEQQDGQLK	1
P07492	0.9407	Gastrin-releasing peptide	GRP	148	13.5	GRELPLVLLALVLCLAPRGR	1
P07602	0.995	Prosaposin	PSAP	524	2.7	TNSTFVQALVEHVK	1
P07737	0.9407	Profilin-1	PFN1	140	11.4	TFVNITPAEVGVLVGK	1
P07738	0.9407	Bisphosphoglycerate mutase	BPGM	259	7.3	AVGPHQFLGDQEAIQAAIK	1
P07858	0.9407	Cathepsin B	CTSB	339	5	GQDHCGIESEVVAGIPR	1
P07864	0.9029	L-lactate dehydrogenase C chain	LDHC	332	3.614	VIGSGCNLDSAR	1
P07900	0.9407	Heat shock protein HSP 90-alpha	HSP90AA1	732	2	HLEINPDHSIIETLR	1
P07942	0.9407	Laminin subunit beta-1	LAMB1	1786	0.7	LNVEGEHCDVCK	1
P07996	1	Thrombospondin-1	THBS1	1170	33.4	DDFDHDSVPDIDDICPENVDISETDFR+DGKGDACDHDDDNDGIPDDKDNCR+DHSGQVFSVVSNGK+DNCPFHYNPAQYDYDR+DTDMDGVGDQCDNCPLEHNPDQLDSDSDR+EVPDACFNHNGEHR+FTGSQPFGQGVEHATANK+GDACDHDDDNDGIPDDK+GDACDHDDDNDGIPDDKDNCR+GDACKDDFDHDSVPDIDDICPENVDISETDFR+GGVNDNFQGVLQNVR+IEDANLIPPVPDDK+IEDANLIPPVPDDKFQDLVDAVR+IGDTCDNNQDIDEDGHQNNLDNCPYVPNANQADHDK+IPESGGDNSVFDIFELTGAAR+KDHSGQVFSVVSNGK+KDNCPNLPNSGQEDYDK+LCNSPSPQMNGKPCEGEAR+MENAELDVPIQSVFTR+NALWHTGNTPGQVR+NPCTDGTHDCNK+QHVVSVEEALLATGQWK+RPPLCYHNGVQYR+SCDSLNNRCEGSSVQTR+TCHIQECDKR+VVNSTTGPGEHLR	26
P07998	1	Ribonuclease pancreatic	RNASE1	156	43.6	CKPVNTFVHEPLVDVQNVCFQEK+HIIVACEGSPYVPVHFDASVEDST+QHMDSDSSPSSSSTYCNQMMR	3

P08107	0.9407	Heat shock 70 kDa protein 1A/1B	HSPA1A	641	2.5	IINEPTAAAIAYGLDR	1
P08185	1	Corticosteroid-binding globulin	SERPINA6	405	27.4	AQLLQGLGFNLTER+AVLQLNEEGVDTAGSTGVTLNLTSKPIILR+GTWTQPFDLASTR+HYYESEVLAMNFQDWATASR+SETEIHQGFQHLHQLFAK+WSAGLTSSQVDLYIPK	6
P08195	0.9352	4F2 cell-surface antigen heavy chain	SLC3A2	630	3	IGDLQAFQGHGAGNLAGLK	1
P08294	1	Extracellular superoxide dismutase [Cu-Zn]	SOD3	240	22.5	AGLAASLAGPHSIVGR+AVVVHAGEDDLGR+RDDDGALHAACQVQPSATLDAAQPR	3
P08514	1	Integrin alpha-IIb	ITGA2B	1039	5.6	HDLLVGAPLYMESR+LQDPVLVSCDSAPCTVVQCDLQEMAR+TLGPSQEETGGVFLCPWR	3
P08519	1	Apolipoprotein(a)	LPA	4548	12.1	ATTVTGTPCQEWAAQEPHR+CQSWSSMTPHR+EAQLLVIENEVCNHYK+KLFDYCDIPLCASSSFDCGKPQVEPK+LFDYCDIPLCASSSFDCGKPQVEPK+SPVVQDCYHGDGR+TCQAWSSMTPHQHSR+TCQAWSSMTPHSHSR	8
P08567	1	Pleckstrin	PLEK	350	10.9	EDPAYLHYYDPAGAEDPLGAIHLR+QQDHFFQAAFLEER	2
P08571	1	Monocyte differentiation antigen CD14	CD14	375	17.3	AFPALTSLDLSDNPGLGER+LKELTLEDLK+LTVGAAQVPAQLLVGALR+VLSIAQAHSPAFSCEQVR	4
P08603	1	Complement factor H	CFH	1231	53.4	AGEQVTYTCATYYK+AQTTVTCMENGWSPTPR+AVYTCNEGYQLLGEINYR+CFEGFGIDGPAIAK+CGPPPPIDNGDITSFPLSVYAPASSVEYQCQNLYQLEGNK+CGPPPPIDNGDITSFPLSVYAPASSVEYQCQNLYQLEGNKR+CTLKPCDYPDIK+CTSTGWIPAPR+CYFPYLENGYNQNYGR+DTSCVNPPTVQNAYIVSR+ECDTDGWTNDIPICEVVK+EEYGHSEVVEYYCNPR+EGWIHTVCINGR+EQVQSCGPPPELLNGNVK+FSCKPGFTIVGPNSVQCYHFGLSPDLPICK+GDAVCTESGWRPLPSCEEK+GEWVALNPLRK+HGGLYHENMR+IEGDEEMHCSDDGFWSK+IPCSQPPQIEHGTINSSR+IVSSAMEPDREYHFGQAVR+KEFDHNSNIR+KGEWVALNPLR+KGEWVALNPLRK+LGYVTADGETSGSITCGK+LNDTLDYECHDGYESNTGSTTGSIVCGYNGWSDLPICYER+NTEILTGSWSDQTYPEGTQAIYK+RNTEILTGSWSDQTYPEGTQAIYK+RPCGHPGDTPFGTFTLTGGNVFEYGVK+SCDIPVFMNAR+SCDNPYIPNGDYSPLR+SIDVACHPGYALPK+SITCIHGVWTQLPQCVAIDK+SPPEISHGVVAHMSDSYQYGEEVTYK+SSIDIENGFISESQYTYALK+SSNLIILEEHLK+TDCLSLPSFENAIPMGEK+TGESVEFVCKR+TKEEYGHSEVVEYYCNPR+TTCWDGKLEYPTCAK+VSVLCQENYLIQEGEEITCK+VSVLCQENYLIQEGEEITCKDGR	42
P08697	1	Alpha-2-antiplasmin	SERPINF2	491	41.8	DSFHLDEQFTVPVEMMQAR+EDFLEQSEQLFGAKPVSLTGK+ELKEQQDSPGNK+ELKEQQDSPGNKDFLQSLK+EQQDSPGNKDFLQSLK+GFPIKEDFLEQSEQLFGAKPVSLTGK+GISEQSLVVSGVQHQSTLELSEVGVEAAAATSIAMSR+LCQDLGPGAFR+LQQVLHAGSGPCLPHLLSR+LVPPMEEDYPQFGSPK+NKFDPSLTQR+QEDDLANINQWVK+QLTSGPNQEQVSPLTLLK+WFLLEQPEIQVAHFPFK	14
P08709	0.9407	Coagulation factor VII	F7	466	4.5	NLIAVLGEHDLSEHDGDEQSR	1

P09172	1	Dopamine beta-hydroxylase	DBH	617	25	AFYYPEEAGLAFGGPGSSR+ALYSFAPISMHCNK+EWEIVNQDNHYSPHFQEIR+GNEALVHHMEVFQCAPEMDSVPHFSGPCDSK+GQIHLDPQQDYQLLQVQR+LEVHYHNPLVIEGR+VISTLEEPTPQCPTSQGR+VVSVHPGDVLITSCTYNTEDR	8
P09211	1	Glutathione S-transferase P	GSTP1	210	18.6	ALPGQLKPFETLLSQNQGGK+DQQEAALVDMVNDGVEDLR	2
P09467	0.9084	Fructose-1,6-bisphosphatase 1	FBP1	338	4.1	EAVLDVIPTDIHQR	1
P09486	1	SPARC	SPARC	303	21.5	LEAGDHPVELLAR+LHLDYIGPCK+RLEAGDHPVELLAR+TFDSSCHFFATK+VCELDENNTPMCVCQDPTSCPAPIGEFEK	5
P09871	1	Complement C1s subcomponent	C1S	688	46.5	CQPVDCGIPESIENGKVEDPESTLFGSVIR+DVVQITCLDGFEVVEGR+EDFDVEAADSAGNCLDSLVFVAGDR+GDSGGAFAVQDPNDKTK+GMDSCKGDSGGAFAVQDPNDKTK+MGPTVSPICLPGTSSDYNLMDGDLGLISGWGR+MLTPEHVFIHPGWK+NCGVNCSGDVFTALIGEIASPNYPKPYPENSR+QFGPYCGHGFPGPLNIETK+REDFDVEAADSAGNCLDSLVFVAGDR+SNALDIIFQTDLTGQK+SSNNPHSPIVEEFQVPYNK+TNFDNDIALVR+VEDPESTLFGSVIR+VEKPTADAEAYVFTPNMICAGGEK+VKNYVDWIMK+YHGDPMPCPK+YTCEEPYYYMENGGGGEYHCAGNGSWVNEVLGPELPK	18
P0C0L4	1	Complement C4-A	C4A	1744	50.3	[illegible]DEDDIPVR+ASAGLLGAHAAAITAYALTLTK+AVGSGATFSHYYYMILSR+DDPDAPLQPVTPLQLFEGR+DFALLSLQVPLK+DFALLSLQVPLKDAK+DSSTWLTAFVLK+ECVGFEAVQEVPVGLVQPASATLYDYYNPER+EELVYELNPLDHR+EGAIHREELVYELNPLDHR+EPFLSCCQFAESLR+EPFLSCCQFAESLRK+EYLIMGLDGATYDLEGHPQYLLDSNSWIEEMPSER+FEQLELRPVLYNYLDK+FSDGLESNSSTQFEVK+FSDGLESNSSTQFEVKK+GCGEQTMIYLAPTLAASR+GHLFLQTDQPIYNPGQR+GLCVATPVQLR+GLEEELQFSLGSK+GPEVQLVAHSPWLK+GSFEFPVGDAVSK+GSFEFPVGDAVSKVLQIEK+HLVPGAPFLLQALVR+ITPGKPYILTVPGHLDEMQLDIQAR+KADGSYAAWLSR+KEVYMPSSIFQDDFVIPDISEPGTWK+KKEVYMPSSIFQDDFVIPDISEPGTWK+KYVLPNFEVK+LEPGKEYLIMGLDGATYDLEGHPQYLLDSNSWIEEMPSER+LHLETDSLALVALGALDTALYAAGSK+LLATLCSAEVCQCAEGK+LLLFSPSVVHLGVPLSVGVQLQDVPR+LNMGITDLQGLR+LQETSNWLLSQQQADGSFQDPCPVLDR+LTVAAPPSGGPGFLSIERPDSRPPR+LVNGQSHISLSK+MRPSTDTITVMVENSHGLR+NTTCQDLQIEVTVK+RFEQLELRPVLYNYLDK+RGHLFLQTDQPIYNPGQR+SHALQLNNRQIRGLEEELQFSLGSK+STQDTVIALDALSAYWIASHTTEER+TLEIPGNSDPNMIPDGDFNSYVR+TTNIQ	56

P0C0L5	1	Complement C4-B	C4B	1744	49.5	[illegible]WLTR+ALEILQEEDLIDEDDIPVR+ASAGLLGAHAAAITAYALTLTK+AVGSGATFSHYYYMILSR+DDPDAPLQPVTPLQLFEGR+DFALLSLQVPLK+DFALLSLQVPLKDAK+ECVGFEAVQEVPVGLVQPASATLYDYYNPER+EELVYELNPLDHR+EGAIHREELVYELNPLDHR+EPFLSCCQFAESLR+EPFLSCCQFAESLRK+EYLIMGLDGATYDLEGHPQYLLDSNSWIEEMPSER+FEQLELRPVLYNYLDK+FSDGLESNSSTQFEVK+FSDGLESNSSTQFEVKK+GCGEQTMIYLAPTLAASR+GHLFLQTDQPIYNPGQR+GLCVATPVQLR+GLEEELQFSLGSK+GPEVQLVAHSPWLK+GSFEFPVGDAVSK+GSFEFPVGDAVSKVLQIEK+HLVPGAPFLLQALVR+ITPGKPYILTVPGHLDEMQLDIQAR+KADGSYAAWLSR+KEVYMPSSIFQDDFVIPDISEPGTWK+KKEVYMPSSIFQDDFVIPDISEPGTWK+KYVLPNFEVK+LEPGKEYLIMGLDGATYDLEGHPQYLLDSNSWIEEMPSER+LHLETDSLALVALGALDTALYAAGSK+LLATLCSAEVCQCAEGK+LLLFSPSVVHLGVPLSVGVQLQDVPR+LNMGITDLQGLR+LQETSNWLLSQQQADGSFQDLSPVIHR+LTVAAPPSGGPGFLSIERPDSRPPR+LVNGQSHISLSK+MRPSTDTITVMVENSHGLR+NTTCQDLQIEVTVK+RFEQLELRPVLYNYLDK+RGHLFLQTDQPIYNPGQR+SHALQLNNRQIRGLEEELQFSLGSK+STQDTVIALDALSAYWIASHTTEER+TLEIPGNSDPNMIPDGDFNSYVR+T	55
P0CG39	0.9795	POTE ankyrin domain family member J	POTEJ	1038	4.6	IWHHTFYNELR+LCYVALDFEQEMAMVASSSSLEK+QEYDESGPSIVHR+QEYDESGPSIVHRK	4
P0DJI8	1	Serum amyloid A-1 protein	SAA1	122	41.8	DPNHFRPAGLPEK+DPNHFRPAGLPEKY+EANYIGSDKYFHAR+FFGHGAEDSLADQAANEWGR+SGKDPNHFRPAGLPEK+SGKDPNHFRPAGLPEKY	6
P0DJI9	1	Serum amyloid A-2 protein	SAA2	122	38.5	DPNHFRPAGLPEK+DPNHFRPAGLPEKY+EANYIGSDKYFHAR+GPGGAWAAEVISNAR+RGPGGAWAAEVISNAR+SGRDPNHFRPAGLPEK+SGRDPNHFRPAGLPEKY	7
P10451	1	Osteopontin	SPP1	314	12.1	AIPVAQDLNAPSDWDSR+DSYETSQLDDQSAETHSHK+GKDSYETSQLDDQSAETHSHK	3
P10643	1	Complement component C7	C7	843	43.2	DGFVQDEGTMFPVGK+ELSHLPSLYDYSAYR+EQTMSECEAGALR+GGGAGFISGLSYLELDNPAGNK+GGGAGFISGLSYLELDNPAGNKR+HTSTEHTSSSR+IACVLPVLMDGIQSHPQKPFYTVGEK+LIDQYGTHYLQSGSLGGEYR+LKQNDFNSVEEK+LLEPHCFPLSLVPTEFCPSPPALK+LSGNVLSYTFQVK+MPYECGPSLDVCAQDER+NVVYTCNEGYSLIGNPVAR+RPSCDIDKPPPNIELTGNGYNELTGQFR+SCVGETTESTQCEDEELEHLR+SLVCNGDSDCDEDSADEDR+SVAVYGQYGGQPCVGNAFETQSCEPTR+SYTSHTNEIHK+VTVSCSGGMSLEGPSAFLCGSSLK+WLVGEMHCQK+YSAWAESVTNLPQVIK	21
P10645	1	Chromogranin-A	CHGA	457	38.3	AEGNNQAPGEEEEEEEEATNTHPPASLPSQK+CIVEVISDTLSKPSPMPVSQECFETLR+LEGQEEEEDNRDSSMK+REEEEEEEEEAEAGEEAVPEEEGPTVVLNPHPSLGYK+RLEGQEEEEDNRDSSMK+RPEDQELESLSAIEAELEK+SEALAVDGAGKPGAEEAQDPEGK+SGEATDGARPQALPEPMQESK	8

P10909	1	Clusterin	CLU	449	42.8	ASSIIDELFQDR+EILSVDCSTNNPSQAK+ELPGVCNETMMALWEECKPCLK+EPQDTYHYLPFSLPHR+KKEDALNETR+KTLLSNLEEAK+KTLLSNLEEAKK+LANLTQGEDQYYLR+LFDSDPITVTVPVEVSR+LFDSDPITVTVPVEVSRK+LKELPGVCNETMMALWEECKPCLK+QLEEFLNQSSPFYFWMNGDR+QQTHMLDVMQDHFSR+RELDESLQVAER+TLLSNLEEAKK+VTTVASHTSDSDVPSGVTEVVVK	16
P11021	1	78 kDa glucose-regulated protein	HSPA5	654	12.2	IEWLESHQDADIEDFK+IINEPTAAAIAYGLDKR+KSDIDEIVLVGGSTR+TKPYIQVDIGGGQTK+VTHAVVTVPAYFNDAQR	5
P11142	1	Heat shock cognate 71 kDa protein	HSPA8	646	9.4	NQTAEKEEFEHQQK+QTQTFTTYSDNQPGVLIQVYEGER+SINPDEAVAYGAAVQAAILSGDK	3
P11226	0.9407	Mannose-binding protein C	MBL2	248	19.8	LGPPGNPGPSGSPGPKGQKGDPGK+LTYTNWNEGEPNNAGSDEDCVLLLK	2
P12111	0.998	Collagen alpha-3(VI) chain	COL6A3	3177	0.8	IIDELNVKPEGTR+SVHNGAPAPVSGEK	2
P12259	1	Coagulation factor V	F5	2224	13.4	AADIEQQAVFAVFDENK+ADKPLSIHPQGIR+ASKPGWWLLNTEVGENQR+AWGESTPLANKPGK+DIHSGLIGPLLICQK+EDNAVQPNSSYTYVWHATER+EKPQSTISGLLGPTLYAEVGDIIK+ETDIEDSDDIPEDTTYKK+FCENPDEVKR+GEYEEHLGILGPIIR+HEDTLTLFPMR+HLSQDTGSPSGMRPWEDLPSQDTGSPSR+LEPEDEESDADYDYQNR+LSEGASYLDHTFPAEK+NKADKPLSIHPQGIR+NSVLNSSTAEHSSPYSEDPIEDPLQPDVTGIR+QHQLGVWPLLPGSFK+SQHLDNFSNQIGK	18
P12814	1	Alpha-actinin-1	ACTN1	892	24.4	ACLISLGYDIGNDPQGEAEFAR+DDPLTNLNTAFDVAEK+DGLGFCALIHR+DHSGTLGPEEFK+HEAFESDLAAHQDR+HRPELIDYGK+HTNYTMEHIR+IDQLEGDHQLIQEALIFDNK+KDDPLTNLNTAFDVAEK+KHEAFESDLAAHQDR+LLETIDQLYLEYAK+MLDAEDIVGTARPDEK+RDQALTEEHAR+TFTAWCNSHLR+TINEVENQILTR+VEQIAAIAQELNELDYYDSPSVNAR+VGWEQLLTTIAR	17
P12882	0.9407	Myosin-1	MYH1	1939	1.3	ALQEAHQQTLDDLQAEEDKVNTLTK	1
P12955	0.9407	Xaa-Pro dipeptidase	PEPD	493	3.2	FEVNNTILHPEIVECR	1
P13591	0.9352	Neural cell adhesion molecule 1	NCAM1	858	3	DGEQIEQEEDDEKYIFSDDSSQLTIK	1
P13598	0.9189	Intercellular adhesion molecule 2	ICAM2	275	4.4	GNETLHYETFGK	1
P13671	1	Complement component C6	C6	934	39.7	AKDLHLSDVFLK+ALNHLPLEYNSALYSR+CLNNQQLHFLHIGSCQDGR+CPINCLLGDFGPWSDCDPCIEK+DLTSLGHNENQQGSFSSQGGSSFSVPIFYSSK+EVDLPEIEADSGCPQPVPPENGFIR+HEGSFIQGAEK+KLECNGENDCGDNSDER+KLECNGENDCGDNSDERDCGR+KYNPIPSVQLMGNGFHFLAGEPR+LECNGENDCGDNSDER+QSGSECICMSPEEDCSHHSEDLCVFDTDSNDYFTSPACK+RQEEDCTFSIMENNGQPCINDDEEMK+RSENINHNSAFK+SENINHNSAFK+SVLRPSQFGGQPCTAPLVAFQPCIPSK+TECIKPVVQEVLTITPFQR+TLNICEVGTIR+TRECNNPAPQR+VPANLENVGFEVQTAEDDLK+VPANLENVGFEVQTAEDDLKTDFYK+YNPIPSVQLMGNGFHFLAGEPR+YTCQGNSWTPPISNSLTCEK	23
P13716	0.9407	Delta-aminolevulinic acid dehydratase	ALAD	330	5.8	GSAADSEESPAIEAIHLLR	1

P13796	1	Plastin-2	LCP1	627	9.6	AYYHLLEQVAPK+EGICAIGGTSEQSSVGTQHSYSEEEK+YPALHKPENQDIDWGALEGETR	3
P14151	1	L-selectin	SELL	372	12.6	NKEDCVEIYIK+SLTEEAENWGDGEPNNKK+TICESSGIWSNPSPICQK	3
P14618	1	Pyruvate kinase PKM	PKM	531	14.9	KGVNLPGAAVDLPAVSEK+LAPITSDPTEATAVGAVEASFK+LNFSHGTHEYHAETIK+TATESFASDPILYRPVAVALDTK	4
P14625	0.9407	Endoplasmin	HSP90B1	803	2.4	FQSSHHPTDITSLDQYVER	1
P14770	0.9352	Platelet glycoprotein IX	GP9	177	7.3	GHGLTALPALPAR	1
P14923	1	Junction plakoglobin	JUP	745	5.4	HPEAEMAQNSVR+ISEDKNPDYR+SAIVHLINYQDDAELATR	3
P15086	0.9955	Carboxypeptidase B	CPB1	417	7.4	NPCDETYCGPAAESEK+STHTGSSCIGTDPNR	2
P15144	1	Aminopeptidase N	ANPEP	967	16.9	AEFNITLIHPK+AQIINDAFNLASAHK+EATDVIIIHSK+GPSTPLPEDPNWNVTEFHTTPK+GVGGSQPPDIDKTELVEPTEYLVVHLK+KDNEETGFGSGTR+KLNYTLSQGHR+KQVTPLFIHFR+KVVATTQMQAADAR+QVTPLFIHFR+QWMENPNNNPIHPNLR+TELVEPTEYLVVHLK+VTLRPYLTPNDR	13
P15169	1	Carboxypeptidase N catalytic chain	CPN1	458	31.2	EALIQFLEQVHQGIK+GMVLDENYNNLANAVISVSGINHDVTSGDHGDYFR+HLYVLEFSDHPGIHEPLEPEVK+IHILPSMNPDGYEVAAAQGPNKPGYLVGR+TASTPTPDDKLFQK+YGGPNHHLPLPDNWK+YVGNMHGNEALGR	7
P15924	1	Desmoplakin	DSP	2871	8.3	AEMDMVAWGVDLASVEQHINSHR+ALLQAILQTEDMLK+AQQIHSQTSQQYPLYDLDLGK+FLEFQYLTGGLVDPEVHGR+GYFNEELSEILSDPSDDTK+LKQESDQLVLNQHPASDK+LLEAQIASGGVVDPVNSVFLPK+NHYNEEMSNLR+NLPLADQGSSHHITVK+RVEEDIQQQK+SVEEVASEIQPFLR+SVQNDSQAIAEVLNQLK+TLELQGLINDLQR+TMIQSPSGVILQEAADVHAR	14
P15941	0.9407	Mucin-1	MUC1	1255	1.4	EGTINVHDVETQFNQYK	1
P16070	0.9407	CD44 antigen	CD44	742	1.6	YGFIEGHVVIPR	1
P16109	0.9407	P-selectin	SELP	830	3	LECLASGIWTNKPPQCLAAQCPPLK	1
P16284	0.9407	Platelet endothelial cell adhesion molecule	PECAM1	738	2.8	DNPTEDVEYQCVADNCHSHAK	1
P16581	0.9084	E-selectin	SELE	610	2.6	YGSSCEFSCEQGFVLK	1
P17020	0.9352	Zinc finger protein 16	ZNF16	682	2.2	SHMSEKPYECNECGK	1
P17301	0.9407	Integrin alpha-2	ITGA2	1181	2.8	AEVPTGVIIGSIIAGILLLLALVAILWKLGFFK	1
P17936	1	Insulin-like growth factor-binding protein 3	IGFBP3	291	42.6	ALAQCAPPPAVCAELVR+AYLLPAPPAPGNASESEEDR+CQPSPDEARPLQALLDGR+EMEDTLNHLK+EPGCGCCLTCALSEGQPCGIYTER+SAGSVESPSVSSTHR+YKVDYESQSTDTQNFSSESK	7
P18065	1	Insulin-like growth factor-binding protein 2	IGFBP2	325	16.9	GDPECHLFYNEQQEAR+GPLEHLYSLHIPNCDK+LAACGPPPVAPPAAVAAVAGGAR	3
P18206	1	Vinculin	VCL	1134	13.8	ILLRNPGNQAAYEHFETMK+LTDELAPPKPPLPEGEVPPPRPPPPEEK+LVQAAQMLQSDPYSVPAR+MQEAMTQEVSDVFSDTTTPIK+QQELTHQEHR+TNISDEESEQATEMLVHNAQNLMQSVK+VAMANIQPQMLVAGATSIAR+VDQLTAQLADLAAR	8
P18428	1	Lipopolysaccharide-binding protein	LBP	481	6.2	SPVTLLAAVMSLPEEHNK+VQLYDLGLQIHK	2
P18669	0.9407	Phosphoglycerate mutase 1	PGAM1	254	8.3	SYDVPPPPMEPDHPFYSNISK	1

P19320	1	Vascular cell adhesion protein 1	VCAM1	739	16.8	DPEIHLSGPLEAGKPITVK+DTTVLVSPSSILEEGSSVNMTCLSQGFPAPK+LHIDDMEFEPK+QLPNGELQPLSENATLTLISTK+SEGTNSTLTLSPVSFENEHSYLCTVTCGHK+SQEFLEDADRK	6
P19652	1	Alpha-1-acid glycoprotein 2	ORM2	201	51.2	EHVAHLLFLR+EQLGEFYEALDCLCIPR+NWGLSFYADKPETTK+SDVMYTDWKK+SVQEIQATFFYFTPNK+SVQEIQATFFYFTPNKTEDTIFLR+TLMFGSYLDDEKNWGLSFYADKPETTK+WFYIASAFRNEEYNKSVQEIQATFFYFTPNK	8
P19823	1	Inter-alpha-trypsin inhibitor heavy chain H2	ITIH2	946	34.2	AEDHFSVIDFNQNIR+AHVSFKPTVAQQR+DKHADPDFTR+ETAVDGELVVLYDVK+FLHVPDTFEGHFDGVPVISK+FYNQVSTPLLR+HLEVDVWVIEPQGLR+IQPSGGTNINEALLR+IYGNQDTSSQLK+IYGNQDTSSQLKK+KFYNQVSTPLLR+LDQIESVITATSANTQLVLETLAQMDDLQDFLSK+LSNENHGIAQR+MLADAPPQDPSCCSGALYYGSK+NILFVIDVSGSMWGVK+NVQFNYPHTSVTDVTQNNFHNYFGGSEIVVAGK+RLSNENHGIAQR+SILQMSLDHHIVTPLTSLVIENEAGDER+SLPGESEEMMEEVDQVTLYSYK+VQFELHYQEVK+VVNNSPQPQNVVFDVQIPK	21
P19827	1	Inter-alpha-trypsin inhibitor heavy chain H1	ITIH1	911	40.2	ADVQAHGEGQEFSITCLVDEEEMK+ADVQAHGEGQEFSITCLVDEEEMKK+ANLSSQALQMSLDYGFVTPLTSMSIR+ERGHMLENHVER+FAHYVVTSQVVNTANEAR+GFSLDEATNLNGGLLR+GHMLENHVER+GHVLFRPTVSQQQSCPTCSTSLLNGHFK+GIEILNQVQESLPELSNHASILIMLTDGDPTEGVTDR+GMADQDGLKPTIDKPSEDSPPLEMLGPR+GSLVQASEANLQAAQDFVR+KAAISGENAGLVR+KGHVLFRPTVSQQQSCPTCSTSLLNGHFK+NHMQYEIVIK+QAVDTAVDGVFIR+QLVHHFEIDVDIFEPQGISK+QYYEGSEIVVAGR+RNHMQYEIVIK+TAFISDFAVTADGNAFIGDIK+TFVLSALQPSPTHSSSNTQR+TMEQFTIHLTVNPQSK+VTFQLTYEEVLKR+VTGVDTDPHFIIHVPQK	23
P19971	0.9407	Thymidine phosphorylase	TYMP	482	3.1	ALPLALVLHELGAGR	1
P20718	0.902	Granzyme H	GZMH	246	4.065	GDSGGPLVCK	1
P20742	1	Pregnancy zone protein	PZP	1482	26.9	AGAFCLSEDAGLGISSTASLR+AVDQSVLLMKPEAELSVSSVYNLLTVK+DLFHCVSFTLPR+EAVKEDNLVHWERPQRPK+EDNLVHWERPQRPK+EGTHGSHVYTK+GSFALSFPVESDVAPIAR+HQDGSYSTFGER+IQHPFTVEEFVLPK+IREEGTDLEVTANR+LEAGINQLSFPLSSEPIQGSYR+MFIFAILPDGEVVGDSEK+MVSGFIPLKPTVK+NELIPLIYLENPR+NQGNTWLTAFVLK+QQNAQGGFSSTQDTVVALHALSR+SGTHTLPVESGDMK+TLLVEAEGIEQEK+VDLSFSPAQSPPASHAHLQVAAAPQSLCALR+VNITVCGEYTYGKPVPGLATVSLCR+VQTVPQTCDGHK+VVSVDENFRPR+VYDYYETDESVVAEYIAPCSTDTEHGNV+YNILPEKEDSPFALK	24
P20851	1	C4b-binding protein beta chain	C4BPB	252	48	DCDPPGNPVHGYFEGNNFTLGSTISYYCEDR+ITFMCNDHYILK+LGHCPDPVLVNGEFSSSGPVNVSDK+LIQEAPKPECEK+SQCLEDHTWAPPFPICK+YYLVGVQEQQCVDGEWSSALPVCK	6

P20929	0.9087	Nebulin	NEB	6669	0.9	HKYTMSPDLPQFLQAKCNAYNISDVCYK+SLFYPYNDSPELR+SLQDDPKLVLSMNVAK	3
P20930	1	Filaggrin	FLG	4061	11.5	HAETSSGGQAASSHEQARSSPGER+HAETSSGGQAASSQEQARSSPGER+HHEASTHADISR+HSASQDGQDTIRGHPGSSR+HSASQEGQDTIRGHPGSSR+HSGIGHGQASSAVR+HSGTGHGQASSAVR+HSQAVQGQSEGSR+HSTSQEGQDTIHGHPGSSSGGR+NQGSSVSQDSDSQGHSEDSER+QGSSVSQDSDSEGHSEDSER	11
P21291	0.9407	Cysteine and glycine-rich protein 1	CSRP1	193	8.8	HEEAPGHRPTTNPNASK	1
P21333	1	Filamin-A	FLNA	2647	32.1	[illegible]VVPCFDASK+ALGALVDSCAPGLCPDWDSWDASKPVTNAR+APSVANVGSHCDLSLK+ATCAPQHGAPGPGPADASK+CAPGVVGPAEADIDFDIIR+DAGEGLLAVQITDPEGKPK+DGSCGVAYVVQEPGDYEVSVK+DGSCSVEYIPYEAGTYSLNVTYGGHQVPGSPFK+DVDIIDHHDNTYTVK+EEGPYEVEVTYDGVPVPGSPFPLEAVAPTKPSK+EGPYSISVLYGDEEVPR+FGGEHVPNSPFQVTALAGDQPSVQPPLR+FNEEHIPDSPFVVPVASPSGDAR+FNGTHIPGSPFK+FVPAEMGTHTVSVK+GLVEPVDVVDNADGTQTVNYVPSR+GQHVPGSPFQFTVGPLGEGGAHK+HTAMVSWGGVSIPNSPFR+IECDDKGDGSCDVR+IPEISIQDMTAQVTSPSGK+KDGSCGVAYVVQEPGDYEVSVK+KTHIQDNHDGTYTVAYVPDVTGR+LQVEPAVDTSGVQCYGPGIEGQGVFR+LVSNHSLHETSSVFVDSLTK+QMQLENVSVALEFLDR+SAGQGEVLVYVEDPAGHQEEAK+SPYTVTVGQACNPSACR+TFSVWYVPEVTGTHK+TGVAVNKPAEFTVDAK+TGVELGKPTHFTVNAK+THEAEIVEGENHTYCIR+THIQDNHDGTYTVAYVPDVTGR+VDVGKDQEFTVK+VEYTPYEEGLHSVDVTYDGSPVPSSPFQVPVTEGCDPSR+VHGPGIQSGTTNKPNK+VHSPSGALEECYVTEIDQDK+VSGQGLHEGHTFEPAEFIIDTR+VTVLFAGQHIAK+VTYTPMAPGSYLISIK+YAPSEAGLHEMDIR+YGGPYHIGGSPFK+YKGQHVPGSPFQFTVGPL	46
P22352	0.9407	Glutathione peroxidase 3	GPX3	226	7.5	YVRPGGGFVPNFQLFEK	1
P22392	1	Nucleoside diphosphate kinase B	NME2	152	28.9	EISLWFKPEELVDYK+TFIAIKPDGVQR+VMLGETNPADSKPGTIR	3
P22692	1	Insulin-like growth factor-binding protein 4	IGFBP4	258	22.5	CRPPVGCEELVR+EDARPVPQGSCQSELHR+GELDCHQLADSFR+GELDCHQLADSFRE+THEDLYIIPIPNCDR	5
P22792	1	Carboxypeptidase N subunit 2	CPN2	545	30.1	DHLGFQVTWPDESK+LEDLEVTGSSFLNLSTNIFSNLTSLGK+LLNIQTYCAGPAYLK+LSNNALSGLPQGVFGK+LYLGSNNLTALHPALFQNLSK+RLFQPLTHLK+SLMLSYNAITHLPAGIFR+SQCTYSNPEGTVVLACDQAQCR+VVFLNTQLCQFRPDAFGGLPR	9
P22891	1	Vitamin K-dependent protein Z	PROZ	400	20	DFAEHLLIPR+QCVPHDQCACGVLTSEK+RAPDLQDLPWQVK+SSVAAMHWMDGSVVTR+TDGCQHFCLPGQESYTCSCAQGYR	5
P23142	1	Fibulin-1	FBLN1	703	12.4	CATPHGDNASLEATFVK+EFTRPEEIIFLR+GYQLSDVDGVTCEDIDECALPTGGHICSYR+MVQEQCCHSQLEELHCATGISLANEQDR	4
P23470	1	Receptor-type tyrosine-protein phosphatase gamma	PTPRG	1445	1.8	DNSALDPIIHGLK+HQSPIDILDQYAR	2
P23528	1	Cofilin-1	CFL1	166	28.9	AVLFCLSEDKK+EILVGDVGQTVDDPYATFVK+KEDLVFIFWAPESAPLK	3

P24592	1	Insulin-like growth factor-binding protein 6	IGFBP6	240	10	HLDSVLQQLQTEVYR+RGPCWCVDR	2
P24593	0.9352	Insulin-like growth factor-binding protein 5	IGFBP5	272	4.4	FVGGAENTAHPR	1
P24821	0.995	Tenascin	TNC	2201	3.1	GHEHSIQFAEMK+GRCINGTCYCEEGFTGEDCGKPTCPHACHTQGR+TVSGNTVEYALTDLEPATEYTLR	3
P25311	1	Zinc-alpha-2-glycoprotein	AZGP1	298	53	AREDIFMETLK+AYLEEECPATLRK+DIVEYYNDSNGSHVLQGR+EIPAWVPFDPAAQITK+HVEDVPAFQALGSLNDLQFFR+NILDRQDPPSVVVTSHQAPGEK+QDPPSVVVTSHQAPGEK+QDPPSVVVTSHQAPGEKK+QKWEAEPVYVQR+QVEGMEDWKQDSQLQK+WEAEPVYVQR+YSKNILDRQDPPSVVVTSHQAPGEK+YSLTYIYTGLSK+YYYDGKDYIEFNK	14
P26038	0.9999	Moesin	MSN	577	4.7	RKPDTIEVQQMK+TQEQLALEMAELTAR	2
P26927	1	Hepatocyte growth factor-like protein	MST1	711	28	CTDDVRPQDCYHGAGEQYR+GTELQHLLHAVVPGPWQEDVADAEECAGR+GTMATTVGGLPCQAWSHK+NPDGSERPWCYTTDPQIER+NPDGSERPWCYTTDPQIEREFCDLPRCGSEAQPR+QGQHFCGGSLVK+RCTDDVRPQDCYHGAGEQYR+VALICLPPEWYVVPPGTK+VSVFVDWIHK+VVGGHPGNSPWTVSLR+WDLQHPHQHPFEPGK+WSAETPHKPQFTFTSEPHAQLEENFCR	12
P26992	0.9407	Ciliary neurotrophic factor receptor subunit alpha	CNTFR	372	3.5	HSPQEAPHVQYER	1
P27169	1	Serum paraoxonase/arylesterase 1	PON1	355	53.5	EVQPVELPNCNLVK+FDVSSFNPHGISTFTDEDNAMYLLVVNHPDAK+GIETGSEDLEILPNGLAFISSGLK+IFFYDSENPPASEVLR+ILLMDLNEEDPTVLELGITGSK+SLDFNTLVDNISVDPETGDLWVGCHPNGMK+VTQVYAENGTVLQGSTVASVYK+VVAEGFDFANGINISPDGK+YVYIAELLAHK	9
P27348	0.9407	14-3-3 protein theta	YWHAQ	245	7.8	TAFDEAIAELDTLNEDSYK	1
P27797	1	Calreticulin	CALR	417	19.4	DEDEEDEEDKEEDEEEDVPGQAK+EDDEDKDEDEEDEEDK+HEQNIDCGGGYVK+IDDPTDSKPEDWDKPEHIPDPDAK+IKDPDASKPEDWDER	5
P27918	1	Properdin	CFP	469	22.4	CSAPEPSQKPPGKPCPGLAYEQR+GLLGGGVSVEDCCLNTAFAYQK+HCYSIQHCPLK+KCSAPEPSQKPPGKPCPGLAYEQR+NVTFWGRPLPR+RPCLHVPACK+RPCLHVPACKDPEEEEL+TCNHPVPQHGGPFCAGDATR	8
P29508	0.9407	Serpin B3	SERPINB3	390	3.6	VLHFDQVTENTTGK	1
P29622	1	Kallistatin	SERPINA4	427	36.5	ALWEKPFISSR+ATLDVDEAGTEAAAATSFAIK+EIEEVLTPEMLMR+FSISGSYVLDQILPR+GDATVFFILPNQGK+GFQHLLHTLNLPGHGLETR+LEASKSFHKATLDVDEAGTEAAAATSFAIK+LFHTNFYDTVGTIQLINDHVK+SQILEGLGFNLTELSESDVHR+VGSALFLSHNLK	10
P30101	0.9407	Protein disulfide-isomerase A3	PDIA3	505	2.8	EATNPPVIQEEKPK	1
P30203	0.9407	T-cell differentiation antigen CD6	CD6	668	4.8	ELMLLIPSIVLGILLLGSLIFIAFILLRIKGK	1
P30740	0.9407	Leukocyte elastase inhibitor	SERPINB1	379	5	TYGADLASVDFQHASEDAR	1
P31146	0.9407	Coronin-1A	CORO1A	461	6.3	KSDLFQEDLYPPTAGPDPALTAEEWLGGR	1
P31151	1	Protein S100-A7	S100A7	101	34.7	ENFPNFLSACDKK+KGTNYLADVFEK+SIIGMIDMFHK	3
P31946	0.9955	14-3-3 protein beta/alpha	YWHAB	246	7.7	TAFDEAIAELDTLNEESYK	1

P31947	1	14-3-3 protein sigma	SFN	248	13.3	SNEEGSEEKGPEVR+TTFDEAMADLHTLSEDSYK	2
P32119	1	Peroxiredoxin-2	PRDX2	198	26.3	KEGGLGPLNIPLLADVTR+LVQAFQYTDEHGEVCPAGWKPGSDTIKPNVDDSK	2
P33151	0.9407	Cadherin-5	CDH5	784	1.7	DWIWNQMHIDEEK	1
P33908	1	Mannosyl-oligosaccharide 1,2-alpha-mannosidase IA	MAN1A1	653	4.4	GYAWGLNELKPISK+MYFDAVQAIETHLIR	2
P35125	0.9407	Ubiquitin carboxyl-terminal hydrolase 6	USP6	1406	1.2	MADTSSTDEDSESDYEK	1
P35228	0.9991	Nitric oxide synthase, inducible	NOS2	1153	4.3	SCSTAREMFEHICR+WYALPAVANMLLEVGGLEFPGCPFNGWYMGTEIGVR	2
P35240	0.9407	Merlin	NF2	595	7.2	AKQKLLEIATKPTYPPMNPIPAPLPPDIPSFNLIGDSLSFDFK	1
P35443	1	Thrombospondin-4	THBS4	961	6.9	DTDGDGRGDACDDDMDGDGIK+LVPNPAQEDSNSDGVGDICESDFDQDQVIDR+NSLWHTGDTSDQVR	3
P35542	1	Serum amyloid A-4 protein	SAA4	130	11.5	AYWDIMISNHQNSNR	1
P35555	0.9854	Fibrillin-1	FBN1	2871	3.5	CSCSPGWIGDGIKCTDLDECSNGTHMCSQHADCKNTMGSYR+HRMDACCCSVGAAWGTEECEECPMR+ISPDLCGRGQCVNTPGDFECKCDEGYESGFMMMK	3
P35556	0.9955	Fibrillin-2	FBN2	2912	3.2	CLCNEGYELTPDGKNCIDTNECVALPGSCSPGTCQNLEGSFR+LLCDNGLCRNTPGSYSCTCPPGYVFR+VCLDIRMEQCYLKWDEDECIHPVPGK	3
P35579	1	Myosin-9	MYH9	1960	23.8	AGVLAHLEEER+DFSALESQLQDTQELLQEENR+DLEAHIDSANK+DVLLQVDDERR+EEVGEEAIVELVENGKK+ELESQISELQEDLESER+EQLEEEEEAKHNLEK+FLSNGHVTIPGQQDK+HEAMITDLEER+HEMPPHIYAITDTAYR+HSQAVEELAEQLEQTK+HSQAVEELAEQLEQTKR+IIGLDQVAGMSETALPGAFK+IRELESQISELQEDLESER+KEEELQAALAR+KLEEEQIILEDQNCK+KLEGDSTDLSDQIAELQAQIAELK+KMEDSVGCLETAEEVKR+KQELEEICHDLEAR+KVEAQLQELQVK+LDPHLVLDQLR+LQQELDDLLVDLDHQR+LQQLFNHTMFILEQEEYQR+MEDSVGCLETAEEVKR+MQQNIQELEEQLEEEESAR+NMDPLNDNIATLLHQSSDK+QELEEICHDLEAR+QIATLHAQVADMK+SMEAEMIQLQEELAAAER+THEAQIQEMR+TQLEELEDELQATEDAK+VEDMAELTCLNEASVLHNLK+VIQYLAYVASSHK+VSHLLGINVTDFTR	34
P35609	0.921	Alpha-actinin-2	ACTN2	894	6.5	HEAFESDLAAHQDR+HLDIPKMLDAEDIVNTPKPDER+HTNYTMEHIR+KHEAFESDLAAHQDR+TFTAWCNSHLR	5
P35858	1	Insulin-like growth factor-binding protein complex acid labile subunit	IGFALS	605	30.9	AFWLDVSHNR+DLHFLEELQLGHNR+FVQAICEGDDCQPPAYTYNNITCASPPEVVGLDLR+IRPHTFTGLSGLR+LAYLQPALFSGLAELR+LHSLHLEGSCLGR+LSHNAIASLRPR+LWLEGNPWDCGCPLK+NLIAAVAPGAFLGLK+SFEGLGQLEVLTLDHNQLQEVK+SLALGTFAHTPALASLGLSNNR	11
P36222	0.9998	Chitinase-3-like protein 1	CHI3L1	383	10.2	SFTLASSETGVGAPISGPGIPGR+THGFDGLDLAWLYPGR	2
P36955	1	Pigment epithelium-derived factor	SERPINF1	418	23	ALYYDLISSPDIHGTYK+DTDTGALLFIGK+IKSSFVAPLEK+ITGKPIKLTQVEHR+KTSLEDFYLDEER+LAAAVSNFGYDLYR+LDLQEINNWVQAQMK	7
P37802	1	Transgelin-2	TAGLN2	199	14.6	DDGLFSGDPNWFPKK+QMEQISQFLQAAER	2
P40197	1	Platelet glycoprotein V	GP5	560	5	NLSSLESVQLDHNQLETLPGDVFGALPR	1
P40925	0.9407	Malate dehydrogenase, cytoplasmic	MDH1	334	6	NVIIWGNHSSTQYPDVNHAK	1

P40926	0.9407	Malate dehydrogenase, mitochondrial	MDH2	338	6.5	LTLYDIAHTPGVAADLSHIETK	1
P41222	1	Prostaglandin-H2 D-isomerase	PTGDS	190	38.9	AQGFTEDTIVFLPQTDK+MATHHTLWMGLALLGVLGDLQAAPEAQVSVQPNFQQDK+SVVAPATDGGLNLTSTFLR	3
P43251	1	Biotinidase	BTD	543	20.4	DAQEVHCDEATK+DVQIIVFPEDGIHGFNFTR+LSSGLVTAALYGR+NPVGLIGAENATGETDPSHSK+QEALELMNQNLDIYEQQVMTAAQK+VDLITFDTPFAGR+WNPCLEPHR	7
P43652	1	Afamin	AFM	599	40.1	AESPEVCFNEESPK+ELISLVEDVSSNYDGCCEGDVVQCIR+ESLLNHFLYEVAR+FTDSENVCQER+GQCIINSNKDDRPK+HELTDEELQSLFTNFANVVDK+IAPQLSTEELVSLGEK+ICAMEGLPQKHNFSHCCSK+KSDVGFLPPFPTLDPEEK+LKHELTDEELQSLFTNFANVVDK+RHPDLSIPELLR+RLCFFYNKK+SCCEEQNKVNCLQTR+SDVGFLPPFPTLDPEEK+TINPAVDHCCK+TYVPPPFSQDLFTFHADMCQSQNEELQR+YAEDKFNETTEK	17
P46721	0.9974	Solute carrier organic anion transporter family member 1A2	SLCO1A2	670	2.4	CGESGACRIYDSTTFR	1
P47755	0.9407	F-actin-capping protein subunit alpha-2	CAPZA2	286	6.6	IDGQQTIIACIESHQFQAK	1
P47929	1	Galectin-7	LGALS7	136	30.9	AVVGDAQYHHFR+GQPFEVLIIASDDGFK+SSLPEGIRPGTVLR	3
P48059	0.9407	LIM and senescent cell antigen-like-containing domain protein 1	LIMS1	325	4.9	NDPYHPDHFNCANCGK	1
P48163	0.9942	NADP-dependent malic enzyme	ME1	572	3.1	ILGLGDLGCNGMGIPVGK	1
P48740	1	Mannan-binding lectin serine protease 1	MASP1	699	15.7	AAGNECPELQPPVHGK+ETTDTEQTPGQEVVLSPGSFMSITFR+FGYILHTDNR+FPETLMEIEIPIVDHSTCQK+LRSDENEQHLGVK+SDENEQHLGVK+VETEDQVLATFCGR+YGVYSYIHHNK	8
P49411	0.9297	Elongation factor Tu, mitochondrial	TUFM	452	3.8	DKPHVNVGTIGHVDHGK	1
P49747	1	Cartilage oligomeric matrix protein	COMP	757	19.8	AFQTVVLDPEGDAQIDPNWVVLNQGR+DTDQDGRGDACDDDIDGDR+FCPDGSPSECHEHADCVLER+GSFQCGPCQPGFVGDQASGCQR+KDNCVTVPNSGQEDVDR+NALWHTGDTESQVR+SNPDQADVDHDFVGDACDSDQDQDGDGHQDSR	7
P49908	1	Selenoprotein P	SEPP1	381	16	CGNCSLTTLKDEDFCKR+EGYSNISYIVVNHQGISSR+VSEHIPVYQQEENQTDVWTLLNGSK	3
P49913	0.9407	Cathelicidin antimicrobial peptide	CAMP	170	7.6	TTQQSPEDCDFKK	1
P50395	0.9407	Rab GDP dissociation inhibitor beta	GDI2	445	3.4	EIRPALELLEPIEQK	1
P50552	1	Vasodilator-stimulated phosphoprotein	VASP	380	13.2	SSSSVTTSETQPCTPSSSDYSDLQR+VKEEIIEAFVQELR+VKQELLEEVKK	3
P50991	0.9407	T-complex protein 1 subunit delta	CCT4	539	2.4	DALSDLALHFLNK	1
P51659	0.9407	Peroxisomal multifunctional enzyme type 2	HSD17B4	736	1.9	IDSEGGVSANHTSR	1
P51884	1	Lumican	LUM	338	35.2	ISETSLPPDMYECLR+KLHINHNNLTESVGPLPK+LHINHNNLTESVGPLPK+LSHNELADSGIPGNSFNVSSLVELDLSYNK+NIPTVNENLENYYLEVNQLEK+NNQIDHIDEK+SLEDLQLTHNK+SLEYLDLSFNQIAR	8

P52565	0.9407	Rho GDP-dissociation inhibitor 1	ARHGDIA	204	7.8	SIQEIQELDKDDESLR	1
P52566	1	Rho GDP-dissociation inhibitor 2	ARHGDIB	201	20.9	APEPHVEEDDDDELDSK+LTLVCESAPGPITMDLTGDLEALKK	2
P52907	0.9407	F-actin-capping protein subunit alpha-1	CAPZA1	286	7	TIDGQQTIIACIESHQFQPK	1
P54098	0.9407	DNA polymerase subunit gamma-1	POLG	1239	1.4	EVTMDCKTPSNPTGMER	1
P54108	0.9998	Cysteine-rich secretory protein 3	CRISP3	245	4.5	EIVNKHNELR+EIVNKHNELRR	2
P55056	1	Apolipoprotein C-IV	APOC4	127	18.1	GFMQTYYDDHLR+MKELLETVVNR	2
P55058	1	Phospholipid transfer protein	PLTP	493	7.3	FLEQELETITIPDLR+IYSNHSALESLALIPLQAPLK	2
P55072	0.9407	Transitional endoplasmic reticulum ATPase	VCP	806	3.3	VVETDPSPYCIVAPDTVIHCEGEPIKR	1
P55103	1	Inhibin beta C chain	INHBC	352	11.4	EQECEIISFAETGLSTINQTR+TALQHLHGVPQGALLEDNR	2
P55290	0.9407	Cadherin-13	CDH13	713	3.2	TLEGPVPLEVIVIDQNDNRPIFR	1
P60174	1	Triosephosphate isomerase	TPI1	286	14.7	ELASQPDVDGFLVGGASLKPEFVDIINAK+TATPQQAQEVHEK	2
P60660	1	Myosin light polypeptide 6	MYL6	151	10.6	VLDFEHFLPMLQTVAK	1
P60709	1	Actin, cytoplasmic 1	ACTB	375	44.5	AVFPSIVGRPR+CPEALFQPSFLGMESCGIHETTFNSIMK+DLYANTVLSGGTTMYPGIADR+DSYVGDEAQSKR+IWHHTFYNELR+KDLYANTVLSGGTTMYPGIADR+LCYVALDFEQEMATAASSSSLEK+QEYDESGPSIVHR+QEYDESGPSIVHRK+SYELPDGQVITIGNER+TTGIVMDSGDGVTHTVPIYEGYALPHAILR	11
P60900	0.9407	Proteasome subunit alpha type-6	PSMA6	246	6.9	ILTEAEIDAHLVALAER	1
P61129	0.9928	Zinc finger CCCH domain-containing protein 6	ZC3H6	1189	5.2	EYDEYSTYSDDNFGNYSDDNFGNYGQETEEDFANQLK+LSSSAGLPLGTSTSVLSGISLYDPR	2
P61626	1	Lysozyme C	LYZ	148	18.9	TPGAVNACHLSCSALLQDNIADAVACAK	1
P61769	1	Beta-2-microglobulin	B2M	119	18.5	SNFLNCYVSGFHPSDIEVDLLK	1
P61981	1	14-3-3 protein gamma	YWHAG	247	11.7	EHMQPTHPIR+TAFDDAIAELDTLNEDSYK	2
P62158	1	Calmodulin	CALM1	149	44.3	EADIDGDGQVNYEEFVQMMTAK+EAFSLFDKDGDGTITTK+MKDTDSEEEIR+VFDKDGNGYISAAELR	4
P62258	0.9977	14-3-3 protein epsilon	YWHAE	255	12.2	AAFDDAIAELDTLSEESYK+HLIPAANTGESK	2
P62805	0.9352	Histone H4	HIST1H4A	103	11.7	DNIQGITKPAIR	1
P62937	0.9407	Peptidyl-prolyl cis-trans isomerase A	PPIA	165	16.4	HTGPGILSMANAGPNTNGSQFFICTAK	1
P63104	1	14-3-3 protein zeta/delta	YWHAZ	245	27.3	DNLTLWTSDTQGDEAEAGEGGEN+GIVDQSQQAYQEAFEISK+KGIVDQSQQAYQEAFEISK+SVTEQGAELSNEER+YLAEVAAGDDKK	5
P63316	0.9352	Troponin C, slow skeletal and cardiac muscles	TNNC1	161	24.8	MLGQNPTPEELQEMIDEVDEDGSGTVDFDEFLVMMVRCMK	1
P67936	1	Tropomyosin alpha-4 chain	TPM4	248	23	EENVGLHQTLDQTLNELNCI+IQALQQQADEAEDR+KIQALQQQADEAEDR+KLVILEGELER+RIQLVEEELDR	5
P68032	1	Actin, alpha cardiac muscle 1	ACTC1	377	31.8	AVFPSIVGRPR+DSYVGDEAQSKR+IWHHTFYNELR+LCYVALDFENEMATAASSSSLEK+QEYDEAGPSIVHR+SYELPDGQVITIGNER+VAPEEHPTLLTEAPLNPK+YPIEHGIITNWDDMEK	8
P68104	0.9407	Elongation factor 1-alpha 1	EEF1A1	462	2.6	YYVTIIDAPGHR	1
P68363	0.9999	Tubulin alpha-1B chain	TUBA1B	451	17.8	AVFVDLEPTVIDEVR+FDGALNVDLTEFQTNLVPYPR+NLDIERPTYTNLNR+QLFHPEQLITGK+VGINYQPPTVVPGGDLAK	5

P68366	0.9999	Tubulin alpha-4A chain	TUBA4A	448	17.9	AVFVDLEPTVIDEIR+FDGALNVDLTEFQTNLVPYPR+NLDIERPTYTNLNR+QLFHPEQLITGK+VGINYQPPTVVPGGDLAK	5
P68871	1	Hemoglobin subunit beta	HBB	147	77.6	EFTPPVQAAYQK+FFESFGDLSTPDAVMGNPK+GTFATLSELHCDK+KVLGAFSDGLAHLDNLK+LLGNVLVCVLAHHFGK+LLVVYPWTQR+VLGAFSDGLAHLDNLK+VLGAFSDGLAHLDNLKGTFATLSELHCDK+VNVDEVGGEALGR+VVAGVANALAHKYH	10
P69905	1	Hemoglobin subunit alpha	HBA1	142	62.7	KVADALTNAVAHVDDMPNALSALSDLHAHK+LLSHCLLVTLAAHLPAEFTPAVHASLDK+TYFPHFDLSHGSAQVK+VADALTNAVAHVDDMPNALSALSDLHAHK+VGAHAGEYGAEALER	5
P78395	0.9873	Melanoma antigen preferentially expressed in tumors	PRAME	509	7.5	HSQTLKAMVQAWPFTCLPLGVLMK+NSHQDFWTVWSGNR	2
P78415	0.9352	Iroquois-class homeodomain protein IRX-3	IRX3	501	5.4	SRTDEEGNAYGSEREEEDEEEDEEDGK	1
P78536	0.9407	Disintegrin and metalloproteinase domain-containing protein 17	ADAM17	824	4.2	VDEGEECDPGIMYLNNDTCCNSDCTLKEGVQCSDR	1
P80108	1	Phosphatidylinositol-glycan-specific phospholipase D	GPLD1	840	15.8	ALEFLQLHNGR+ELLLEHQDAYQAGIVFPDCFYPSICK+FGGVLHLSDLDDDGLDEIIMAAPLR+FHDVSESTHWTPFLNASVHYIR+LGTSLSSGHVLMNGTLK+TMFIGGSQLSQK+VYLIYGNDLGLPPVDLDLDK	7
P80188	0.9136	Neutrophil gelatinase-associated lipocalin	LCN2	198	10.6	SLGLPENHIVFPVPIDQCIDG	1
P80723	1	Brain acid soluble protein 1	BASP1	227	15.4	KTEAPAAPAAQETK+SDGAPASDSKPGSSEAAPSSK	2
P82987	0.9407	ADAMTS-like protein 3	ADAMTSL3	1691	0.9	HTACQHNSSDSNCDDR	1
P98088	1	Mucin-5AC (Fragments)	MUC5AC	5030	2.4	AESFPNTPLADLGQDVICSHTEGLICLNK+SHPEVSIEHLGQVVQCSR	2
P98095	0.9954	Fibulin-2	FBLN2	1184	4.2	EGETCGAEDNDSCGISLYKQCCDCCGLGLR+TCRPEGHPPQPEAPQEPALK	2
P98160	1	Basement membrane-specific heparan sulfate proteoglycan core protein	HSPG2	4391	2	AELLVTEAPSKPITVTVEEQR+CSCHGHSEACEPETGACQGCQHHTEGPR+EDGRPVPSGTQQR+EQAWQRPDGQPATR+GGSLPPHTQVHGSR	5
Q00610	1	Clathrin heavy chain 1	CLTC	1675	3.9	ALEHFTDLYDIK+IHEGCEEPATHNALAK+NNRPSEGPLQTR+RPLIDQVVQTALSETQDPEEVSVTVK	4
Q01459	1	Di-N-acetylchitobiase	CTBS	385	12.5	DPAGHFHQVWYDNPQSISLK+HHPDFEVFVFDVGQK+QQTEEMWEVLKPK	3
Q01518	1	Adenylyl cyclase-associated protein 1	CAP1	475	12.4	EFHTTGLAWSK+KEPAVLELEGK+LEAVSHTSDMHR+SSEMNVLIPTEGGDFNEFPVPEQFK	4
Q02383	1	Semenogelin-2	SEMG2	582	5.5	GQLPSGSSQFPHGQK+SQNQVTIHSQDQEHGHK	2
Q02413	1	Desmoglein-1	DSG1	1049	9.8	ALNSMGQDLERPLELR+SSSDHHFNQTIGSASPSTAR+VIAPSSSLPTSLTIHHPR+VIQPTSGMIGSLSMHPELANAHNVIVTER+VVGPISGADLHGMLEMPDLR	5
Q02817	0.9995	Mucin-2	MUC2	5179	0.7	SCVCTNSSQVVCRPEEGK+TVLMNHCSGSCGTFVMYSAK	2
Q02985	1	Complement factor H-related protein 3	CFHR3	330	15.2	CIHPCIITEENMNK+CYFPYLENGYNQNYGR+FVQGNSTEVACHPGYGLPK+KFVQGNSTEVACHPGYGLPK	4
Q03164	0.995	Histone-lysine N-methyltransferase 2A	KMT2A	3969	1.4	DLDSASGPQVSNFTQTVDAPNSMGLEQNK+GNFCPLCDKCYDDDDYESKMMQCGK	2

Q03591	1	Complement factor H-related protein 1	CFHR1	330	41.8	CGPPPPIDNGDITSFPLSVYAPASSVEYQCQNLYQLEGNK+CGPPPPIDNGDITSFPLSVYAPASSVEYQCQNLYQLEGNKR+INHGILYDEEK+LCFFPFVENGHSESSGQTHLEGDTVQIICNTGYR+TGESAEFVCKR+TTCWDGKLEYPTCAK+YKPFSQVPTGEVFYYSCEYNFVSPSK	7
Q04756	1	Hepatocyte growth factor activator	HGFAC	655	17.1	CQIAGWGHLDENVSGYSSSLR+DSVSVVLGQHFFNR+KWCATTHNYDR+MLHACTSEGSAHR+QGHVEQCECFGGR+SQFVQPICLPEPGSTFPAGHK+VQLSPDLLATLPEPASPGR+WCATTHNYDR	8
Q04917	0.9999	14-3-3 protein eta	YWHAH	246	27.2	ELETVCNDVLSLLDK+EQMQPTHPIR+QAFDDAIAELDTLNEDSYK+YLAEVASGEKKNSVVEASEAAYK	4
Q05682	1	Caldesmon	CALD1	793	5.5	EAEGAPQVEAGKR+RGETESEEFEK+SAKPTKPAASDLPVPAEGVR	3
Q06033	1	Inter-alpha-trypsin inhibitor heavy chain H3	ITIH3	890	24.2	DFLGFYVVDSHR+DYIFGNYIER+EHLVQATPENLQEAR+ESPGNVQIVNGYFVHFFAPQGLPVVPK+GHGATNDLTFTEEVDMK+GHVSFKPSLDQQR+KGHVSFKPSLDQQR+SCPTCTDSLLNGDFTITYDVNR+STSIVIMLTDGDANVGESRPEK+TAFITNFTLTIDGVTYPGNVK+VSDIRPGSDPTKPDATLVVK+VTFELTYEELLKR+YHFVTPLTSMVVTKPEDNEDER	13
Q07954	1	Prolow-density lipoprotein receptor-related protein 1	LRP1	4544	2.6	CIPMSWTCDKEDDCEHGEDETHCNK+CNQFVDCEDASDEMNCSATDCSSYFR+HTVDQTRPGAFER+QTTAMDFSYANETVCWVHVGDSAAQTQLK+SCEGVTHVCDPSVK+TTLLAGDIEHPR	6
Q08188	0.9998	Protein-glutamine gamma-glutamyltransferase E	TGM3	693	4.8	SDLGPSYGGWQVLDATPQER+VITNFNSAHDTDR	2
Q08380	1	Galectin-3-binding protein	LGALS3BP	585	39.3	AAFGQGSGPIMLDEVQCTGTEASLADCK+AAIPSALDTNSSK+ALGFENATQALGR+ASHEEVEGLVEK+ELSEALGQIFDSQR+GLNLTEDTYKPR+GQWGTVCDNLWDLTDASVVCR+IYTSPTWSAFVTDSSWSAR+KTLQALEFHTVPFQLLAR+SDLAVPSELALLK+STSSFPCPAGHFNGFR+TLQALEFHTVPFQLLAR+TVIRPFYLTNSSGVD+YKGLNLTEDTYKPR+YSSDYFQAPSDYR+YYPYQSFQTPQHPSFLFQDK+YYPYQSFQTPQHPSFLFQDKR	17
Q08554	1	Desmocollin-1	DSC1	894	4.7	HCEDYVCSYNYEGK+VQDQDLPNTPHSK+VTATDLDEPDTLHTR	3
Q08830	0.9407	Fibrinogen-like protein 1	FGL1	312	4.5	NLHFLTTQEDYTLK	1
Q0ZGT2	0.9407	Nexilin	NEXN	675	2.5	EAENFHEEDDVDVRPAR	1
Q10588	0.999	ADP-ribosyl cyclase/cyclic ADP-ribose hydrolase 2	BST1	318	17	DMGFQYSCINDYRPVK+DSSGVIHVMLNGSEPTGAYPIK+LLQCVDHSTHPDCALK	3
Q12802	0.9967	A-kinase anchor protein 13	AKAP13	2813	0.8	NAASDAEMNHRSSMRVLGDVVR	1
Q12805	1	EGF-containing fibulin-like extracellular matrix protein 1	EFEMP1	493	21.9	CVNHYGGYLCLPK+EDEMCWNYHGGFR+EHIVDLEMLTVSSIGTFR+IQCAAGYEQSEHNVCQDIDECTAGTHNCR+RGEQCVDIDECTIPPYCHQR+TCQDINECETTNECR	6
Q12959	0.9407	Disks large homolog 1	DLG1	904	3.4	YQDEDTPPQEHISPQITNEVIGPELVHVSEK	1
Q13093	1	Platelet-activating factor acetylhydrolase	PLA2G7	441	6.1	GSVHQNFADFTFATGK+TLKQEEETHIR	2
Q13103	0.9243	Secreted phosphoprotein 24	SPP2	211	5.7	VSAQQVQGVHAR	1

Q13201	1	Multimerin-1	MMRN1	1228	11.4	AQEQQSLIHTNQAESHTAVGR+FVLVQENRPTLTDIVELR+GVAEQQQQQGCGDPEVMQK+HSWTIPEDGNSQK+KSNEQATSLNTVGGTGGIGGVGGTGGVGNR+QTHLEGALEQEHSR+SNEQATSLNTVGGTGGIGGVGGTGGVGNR+TLHEVLTMCHNASTSVSELNATIPK	8
Q13255	0.9352	Metabotropic glutamate receptor 1	GRM1	1194	3.4	FMSAWAQVIIASILISVQLTLVVTLIIMEPPMPILSYPSIK	1
Q13387	0.9826	C-Jun-amino-terminal kinase-interacting protein 2	MAPK8IP2	824	2.4	LSLSGGGPEFQRCSHFFQMK	1
Q13576	0.9407	Ras GTPase-activating-like protein IQGAP2	IQGAP2	1575	0.8	TLEQTGHVSSENK	1
Q13586	0.9407	Stromal interaction molecule 1	STIM1	685	1.8	IDKPLCHSEDEK	1
Q13790	0.9407	Apolipoprotein F	APOF	326	4.3	SGVQQLIQYYQDQK	1
Q13822	0.9997	Ectonucleotide pyrophosphatase/phosphodiesterase family member 2	ENPP2	863	2.7	DIEHLTSLDFFR+RIEDIHLLVER	2
Q14161	0.9407	ARF GTPase-activating protein GIT2	GIT2	759	3.3	QNSTPESDYDNTPNDMEPDGMGSSR	1
Q14247	1	Src substrate cortactin	CTTN	550	8.5	ASAGHAVSIAQDDAGADDWETDPDFVNDVSEK+TVQGSGHQEHINIHK	2
Q14515	1	SPARC-like protein 1	SPARCL1	664	10.1	EPGEVGTHNDNQER+ETAVSTEDDSHHK+NHGVDDDGDDDGDDGGTDGPR+VHENENIGTTEPGEHQEAK	4
Q14520	1	Hyaluronan-binding protein 2	HABP2	560	19.3	DEIPHNDIALLK+EEFHEQSFR+FCEIGSDDCYVGDGYSYR+HPYTGPSCSQVVPVCRPNPCQNGATCSR+KEEFHEQSFR+LKPVDGHCALESK+TVCLPDGSFPSGSECHISGWGVTETGK	7
Q14624	1	Inter-alpha-trypsin inhibitor heavy chain H4	ITIH4	930	50.3	AEAQAQYSAAVAK+AFITNFSMIIDGMTYPGIIK+AISGGSIQIENGYFVHYFAPEGLTTMPK+ANTVQEATFQMELPK+ANTVQEATFQMELPKK+DQFNLIVFSTEATQWRPSLVPASAENVNK+EKAEAQAQYSAAVAK+ETLFSVMPGLK+FKPTLSQQQK+FSSHVGGTLGQFYQEVLWGSPAASDDGR+FSSHVGGTLGQFYQEVLWGSPAASDDGRR+HRQGPVNLLSDPEQGVEVTGQYER+IPKPEASFSPR+KAFITNFSMIIDGMTYPGIIK+LDYQEGPPGVEISCWSVEL+LPEGSVSLIILLTDGDPTVGETNPR+LPTQNITFQTESSVAEQEAEFQSPK+MNFRPGVLSSR+MTMDKTGLLLLSDPDK+NGIDIYSLTVDSR+NMEQFQVSVSVAPNAK+NPLVWVHASPEHVVVTR+NQALNLSLAYSFVTPLTSMVVTKPDDQEQSQVAEKPMEGESR+QGPVNLLSDPEQGVEVTGQYER+QLGLPGPPDVPDHAAYHPFR+RLDYQEGPPGVEISCWSVEL+RLGVYELLLK+SFAAGIQALGGTNINDAMLMAVQLLDSSNQEER+SPEQQETVLDGNLIIR+WKETLFSVMPGLK+YIFHNFMER	31
Q14766	1	Latent-transforming growth factor beta-binding protein 1	LTBP1	1721	5.8	ASGLGDHCEDINECLEDK+CVDIDECTQVQHLCSQGR+DQCEDIDECQHR+GFVPAGESSSEAGGENYK+HPPEASVQIHQVSR+VQEGYTCDCFDGYHLDTAK	6
Q15063	1	Periostin	POSTN	836	16.1	DGHFTLFAPTNEAFEK+EGETITEVIHGEPIIK+GCPAVLPIDHVYGTLGIVGATTTQR+GSFTYFAPSNEAWDNLDSDIRR+IIHGNQIATNGVVHVIDR+LLYPADTPVGNDQLLEILNK+VGLNELYNGQILETIGGK	7

Q15084	0.9243	Protein disulfide-isomerase A6	PDIA6	440	3.2	TGEAIVDAALSALR	1
Q15113	1	Procollagen C-endopeptidase enhancer 1	PCOLCE	449	5.3	ATSGTEHQFCGGR+VFDLELHPACR	2
Q15485	0.9407	Ficolin-2	FCN2	313	7.3	LGEFWLGNDNIHALTAQGTSELR	1
Q15555	0.9407	Microtubule-associated protein RP/EB family member 2	MAPRE2	327	5.5	SSPAAKPGSTPSRPSSAK	1
Q15560	0.9407	Transcription elongation factor A protein 2	TCEA2	299	9.4	NCTYTQVQTRSSDEPMTTFVVCNECGNR	1
Q15582	1	Transforming growth factor-beta-induced protein ig-h3	TGFBI	683	12.2	AAVAASGLNTMLEGNGQYTLLAPTNEAFEK+ADHHATNGVVHLIDK+QAGLGNHLSGSER+QHGPNVCAVQK+YLYHGQTLETLGGK	5
Q15848	1	Adiponectin	ADIPOQ	244	15.6	IFYNQQNHYDGSTGK+NGLYADNDNDSTFTGFLLYHDTN	2
Q15911	0.9999	Zinc finger homeobox protein 3	ZFHX3	3703	0.4	SLGGHMTMMHSRNSCK	1
Q15942	1	Zyxin	ZYX	572	7.7	LGHPEALSAGTGSPQPPSFTYAQQR+VNPFRPGDSEPPPAPGAQR	2
Q16270	1	Insulin-like growth factor-binding protein 7	IGFBP7	282	24.8	DACGCCPMCARGEGEPCGGGGAGRGYCAPGMECVK+EDAGEYECHASNSQGQASASAK+ITVVDALHEIPVK	3
Q16280	0.9407	Cyclic nucleotide-gated olfactory channel	CNGA2	664	5.1	DDHRTSSRPHSAADDDTSSELQRLADVDAPQQGR	1
Q16610	1	Extracellular matrix protein 1	ECM1	540	37.2	ACPSHQPDISSGLELPFPPGVPTLDNIK+CCDLPFPEQACCAEEEK+EGTPAPFGDQSHPEPESWNAAQHCQQDR+ELPSLQHPNEQK+FSCFQEEAPQPHYQLR+HIPGLIHNMTAR+LDGFPPGRPSPDNLNQICLPNR+QHVVYGPWNLPQSSYSHLTR+SLPMDHPDSSQHGPPFEGQSQVQPPPSQEATPLQQEK+TRPHWCCTR	10
Q16643	0.9407	Drebrin	DBN1	649	2.5	LREDENAEPVGTTYQK	1
Q16787	0.9955	Laminin subunit alpha-3	LAMA3	3333	1.8	IESINQQLLPLGNISDNMDRIRELIQQAR+SNYFGCQGCQCDIGGALSSMCSGPSGVCQCR	2
Q17R98	0.9189	Zinc finger protein 827	ZNF827	1081	1.8	QEQNDQYTPSNRFIWNQGK	1
Q19T08	0.9407	Endothelial cell-specific chemotaxis regulator	ECSCR	205	22	EDATILPSPTSETVLTVAAFGVISFIVILVVVVIILVGVVSLRFK	1
Q2MKA7	0.9407	R-spondin-1	RSPO1	263	14.8	GRCYPACPEGSSAANGTMECSSPAQCEMSEWSPWGPCSK	1
Q4LE39	0.997	AT-rich interactive domain-containing protein 4B	ARID4B	1312	4.3	ALDEPPYLTVGTDVSAK+EDSSSSEAEEEEEEEDDEKEKEDNSSEEEEEIEPFPEER	2
Q587J7	0.9297	Putative ATP-dependent RNA helicase TDRD12	TDRD12	1177	1.3	DTNKRVESSVYWPAK	1
Q5D862	1	Filaggrin-2	FLG2	2391	5.9	HQEEESETEEDEEDTPGHK+HSSWSEGEEHGYSSGHSR+QSSYGQHGSGSSQSSGYGQYGSR+SGQSSYGQHSSGSSQSSGYGQHGSR+SGSGQSSGFGQHGSGSGQSSGFGQHESGSGK	5
Q5JQC9	0.9407	A-kinase anchor protein 4	AKAP4	854	2.9	MMAYSDTTMMSDDIDWLRSHRGVCK	1
Q5JQF7	0.9352	Putative uncharacterized protein encoded by LINC01556	LINC01556	62	30.6	YIYESPNHRGKDSSFLAQK	1
Q5PSV4	0.9407	Breast cancer metastasis-suppressor 1-like protein	BRMS1L	323	7.1	MECLDEMSNLEKQFTDLKDQLYK	1
Q5QJE6	0.9407	Deoxynucleotidyltransferase terminal-interacting protein 2	DNTTIP2	756	3.7	SEEDSSDHDENEDEFSDEEDFLNSTKAK	1
Q5T1H1	0.901	Protein eyes shut homolog	EYS	3165	0.221	FLNFGIR	1
Q5T749	0.9407	Keratinocyte proline-rich protein	KPRP	579	1.9	GRPAVCQPQGR	1
Q5T751	0.9407	Late cornified envelope protein 1C	LCE1C	118	28.8	SHCHRPQSSGCCSQPSGGSSCCGGGSGQHSGGCC	1

Q5T7N2	0.9024	LINE-1 type transposase domain-containing protein 1	L1TD1	865	1.04	DIAPVLDLK	1
Q5VWK0	0.9407	Neuroblastoma breakpoint family member 6	NBPF6	638	2.2	DQEEIEDQSPPCPR	1
Q66K66	0.9243	Transmembrane protein 198	TMEM198	360	10	SWALLALWPLLSLMGVLVQWRVTAEGDSHTEVVISR	1
Q6EMK4	1	Vasorin	VASN	673	5.8	ESHVTLASPEETR+HIQPGAFDTLDR+NLHDLDVSDNQLER	3
Q6JEL2	0.995	Kelch-like protein 10	KLHL10	608	6.1	NILCSCSSYFRALFTSGWNNTEK+TDEWYDAHDMSIYR	2
Q6JVE6	0.9407	Epididymal-specific lipocalin-10	LCN10	187	15	QGLLVLALVLVLVLVLAAGSQVQEWYPR	1
Q6T4R5	0.9407	Nance-Horan syndrome protein	NHS	1651	1.4	NCAFPTEGFQRVSAARPNDLDGK	1
Q6UWP8	1	Suprabasin	SBSN	590	20	FGQGAHHAAGQAGNEAGR+FGQGVHHAAGQAGNEAGR+LGQGVNHAADQAGK+VVQGLHHGVSQAGR	4
Q6UX71	0.9407	Plexin domain-containing protein 2	PLXDC2	529	2.8	HRQDWVDSGCPEESK	1
Q6UXB8	1	Peptidase inhibitor 16	PI16	463	17.7	EHYNLSAATCSPGQMCGHYTQVVWAK+GENLFAITDEGMDVPLAMEEWHHER+GKRPYQEGTPCSQCPSGYHCK+LMVELHNLYR+RPYQEGTPCSQCPSGYHCK	5
Q6W4X9	0.9407	Mucin-6	MUC6	2439	0.8	LDDPGEICTFQDIPSTHVR	1
Q6ZMR3	0.9029	L-lactate dehydrogenase A-like 6A	LDHAL6A	332	3.614	VIGSGCNLDSAR	1
Q6ZMY6	0.9407	WD repeat-containing protein 88	WDR88	472	3.4	GHNDWVMDVAISNNKK	1
Q6ZNH5	0.9189	Zinc finger protein 497	ZNF497	498	5.6	SELLSHRRTHTGERPYACGECGKPFSHR	1
Q6ZV73	1	FYVE, RhoGEF and PH domain-containing protein 6	FGD6	1430	4.1	KPTQEAYQNELKIESVER+QELAESTDNFNCKYEGNQSNDYISPMCSCSSECIHKLGHR	2
Q6ZVX7	0.9297	F-box only protein 50	NCCRP1	275	4	TVIAQHHVAPR	1
Q70CQ2	0.977	Ubiquitin carboxyl-terminal hydrolase 34	USP34	3546	1.6	DSSIIDPGTEQDLPSPENSSVK+VWELLMLLPTCPNMLMAFQNISDEQSNDGFNWK	2
Q75N90	0.9982	Fibrillin-3	FBN3	2809	1.4	LSDSSAACEDYR+NECREIPNVCSHGDCMDTEGSYMCLCHR	2
Q76LX8	0.995	A disintegrin and metalloproteinase with thrombospondin motifs 13	ADAMTS13	1427	2.3	SLVELTPIAAVHGR+WHVGTWMECSVSCGDGIQR	2
Q86TB3	0.9136	Alpha-protein kinase 2	ALPK2	2170	0.8	FTDGDLNNDGPHDEGLR	1
Q86TH1	1	ADAMTS-like protein 2	ADAMTSL2	951	4	LCDPNTRPVGEK+MLSPGFDSSVYSDLCEAAEAVRPEER	2
Q86TP1	0.9407	Protein prune homolog	PRUNE	453	4.6	SDTALEEAVAEVLDHRPIEPK	1
Q86UD1	0.9407	Out at first protein homolog	OAF	273	4.4	QVGDHGKPCVCR	1
Q86UE4	1	Protein LYRIC	MTDH	582	3.3	KTEPSAWSQDTGDANTNGK	1
Q86UX7	1	Fermitin family homolog 3	FERMT3	667	25.3	ARGEELDEDLFLQLTGGHEAF+EKEPEEELYDLSK+ILEAHQNVAQLSLAEAQLR+KDEILGIANNR+LEGSAPTDVLDSLTTIPELK+LSQSGEVGEPAGTDPGLDDLDVALSNLEVK+QDWSDHAIWWEQK+TGSGGPGNHPHGPDASAEGLNPYGLVAPR+VTGESHIGGVLLK	9
Q86VB7	1	Scavenger receptor cysteine-rich type 1 protein M130	CD163	1156	15	CKGNESSLWDCPAR+HNCDHAEDAGVICSK+HSNCTHQQDAGVTCSDGSNLEMR+HYCNHNEDAGVTCSDGSDLELR+IWMDHVSCR+NWQWGGLTCDHYEEAK+QLGCGEAINATGSAHFGEGTGPIWLDEMK+TLGAWGSLCNSHWDIEDAHVLCQQLK+WGTVCDDNFNIDHASVICR	9
Q86XX4	0.9407	Extracellular matrix protein FRAS1	FRAS1	4008	0.6	GSACEFCMCDHGQVTCQTGECAK	1
Q86YW5	0.9352	Trem-like transcript 1 protein	TREML1	311	5.1	VSLNILPPEEEEETHK	1

Q86YZ3	1	Hornerin	HRNR	2850	16.6	GEQHGSSSGSSSSYGQHGSGSR+GPYESGSG HSSGLGHR+GRHGSDFGHSSSYGQHGSGSG WSSSNGPHGSVSGQSSGFGHK+GSGSGQSPS SGQHGTGFGR+HGSGSGHSSSYGQHGSGSG WSSSSGR+HGSSSGSSSSYGQHGSGSR+QSLG HGQHGSGSGQSPSPSR+QSLGHSRHGSGSG QSPSPSR+SEQHGSSSGLSSSYGQHGSGSHQS SGHGR+SGSGQSSGYSQHGSGSSHSSGYR+SS SGQSSGYSQHGSGSGHSSGYGQHGSR+SSSG QSSGYTQHGSGSGHSSSYEQHGSR	12
Q8IWB6	0.9084	Inactive serine/threonine-protein kinase TEX14	TEX14	1497	1.1	MEREACCFGSEDESSSK	1
Q8IZP7	0.9407	Heparan-sulfate 6-O-sulfotransferase 3	HS6ST3	471	4.9	EGEEEEEEDEPDPEAPENGSLPR	1
Q8N5M9	0.9407	Protein jagunal homolog 1	JAGN1	183	10.9	YEIKKLIYVHLVIWLLLVAK	1
Q8N6C8	1	Leukocyte immunoglobulin-like receptor subfamily A member 3	LILRA3	439	7.7	EGEDEHPQCLNSHSHAR+KPSLSVQPGPVVA PGEK	2
Q8N961	0.9955	Ankyrin repeat and BTB/POZ domain-containing protein 2	ABTB2	1025	3.9	GAAWWCYSGSMNSRHNSWDTVNTVLPEDP EVADLFSRCPR	1
Q8NBJ4	1	Golgi membrane protein 1	GOLM1	401	6	EQVVEDRPVGGR+NIDVFNVEDQKR	2
Q8NBP7	0.9407	Proprotein convertase subtilisin/kexin type 9	PCSK9	692	2.5	EASIHASCCHAPGLECK	1
Q8NF64	0.9946	Zinc finger MIZ domain-containing protein 2	ZMIZ2	920	5.3	LQHNLAVSNHVFQLR+SIPGYPSSPLPGNPTP PMTPSSSVPYMSPNQEVK	2
Q8NGU4	0.9407	Putative olfactory receptor 2I1	OR2I1P	316	5.7	LACGGDGDTTENQMFAAR	1
Q8TBZ6	0.9407	tRNA methyltransferase 10 homolog A	TRMT10A	339	5	MEEGGSDSDSSEEEYSR	1
Q8TDX5	0.9924	2-amino-3-carboxymuconate-6-semialdehyde decarboxylase	ACMSD	336	4.5	CSLFVHPWDMQMDGR	1
Q8WU20	1	Fibroblast growth factor receptor substrate 2	FRS2	508	4.3	DQVSGSGANNTEWDTGYDSDER	1
Q8WUA4	0.9243	General transcription factor 3C polypeptide 2	GTF3C2	911	2.1	KIRQPAACPGGEEVDGAPR	1
Q8WUU4	0.901	Zinc finger protein 296	ZNF296	475	1.895	TLSSFSNLK	1
Q8WWQ8	0.9407	Stabilin-2	STAB2	2551	0.7	CNQGPLGDGSCDCDVGWR	1
Q8WYB5	1	Histone acetyltransferase KAT6B	KAT6B	2073	1.8	LDDHEEEEEEDEEPSHNEDHDADDEDDSHME SAEVEK	1
Q92496	0.9955	Complement factor H-related protein 4	CFHR4	578	6.4	CIHPCIITEENMNK+CISMKPCEFPEIQHGHLY YENTR	2
Q92686	0.9407	Neurogranin	NRGN	78	19.2	KGPGPGGPGGAGVAR	1
Q92820	1	Gamma-glutamyl hydrolase	GGH	318	7.9	NLDGISHAPNAVK+YPVYGVQWHPEK	2
Q92824	0.9989	Proprotein convertase subtilisin/kexin type 5	PCSK5	1860	2.4	DCPEGYYADEDSNRCAHCHSSCR+EWSLVLY GTSVQPYSPTNEFPK	2
Q92954	1	Proteoglycan 4	PRG4	1404	6	DATCNCDYNCQHYMECCPDFK+GFGGLTGQI VAALSTAK+GLPNVVTSAISLPNIR+ITEVWGIP SPIDTVFTR+VTTPDTSTTQHNK	5
Q92995	0.9407	Ubiquitin carboxyl-terminal hydrolase 13	USP13	863	3.5	LMNQLIDPSDIDESSVMQLAEMGFPLEACR	1
Q96AQ6	0.9407	Pre-B-cell leukemia transcription factor-interacting protein 1	PBXIP1	731	2.2	CSSSDDDTDVDMEGLR	1
Q96BR6	0.9407	Zinc finger protein 669	ZNF669	464	5.6	SHDIEAGCSDSAYNPSTLGGQGVWIA	1
Q96GA3	0.9407	Protein LTV1 homolog	LTV1	475	8.4	SENEDDSEWEDVDDEKGDSNDDYDSAGLLSD EDCMSVPGK	1
Q96HR3	0.903	Mediator of RNA polymerase II transcription subunit 30	MED30	178	5.056	TMEIFQLLR	1
Q96HV5	0.9352	Transmembrane protein 41A	TMEM41A	264	6.1	HLQLNETSTANHIHSR	1

Q96IY4	1	Carboxypeptidase B2	CPB2	423	23.2	DHEELSLVASEAVR+HWCEEGASSSSCSETYCGLYPESEPEVK+NAIWIDCGIHAR+SFYANNHCIGTDLNR+SKDHEELSLVASEAVR+YTHGHGSETLYLAPGGGDDWIYDLGIK	6
Q96KN2	1	Beta-Ala-His dipeptidase	CNDP1	507	16.4	AIHLDLEEYR+EEILMHLWR+EWVAIESDSVQPVPR+LVPHMNVSAVEK+SVVLIPLGAVDDGEHSQNEK+VFQYIDLHQDEFVQTLK	6
Q96KR1	0.9407	Zinc finger RNA-binding protein	ZFR	1074	1.4	RDSDGVDGFEAEGKK	1
Q96L73	0.9968	Histone-lysine N-methyltransferase, H3 lysine-36 and H4 lysine-20 specific	NSD1	2696	1.9	CCSSDTKGSPLASISKSGK+NHEHVNVSWCFVCSEGGSLLCCDSCPAAFHR	2
Q96NZ9	1	Proline-rich acidic protein 1	PRAP1	151	13.9	VVEPPEKDDQLVVLFPVQKPK	1
Q96PD5	1	N-acetylmuramoyl-L-alanine amidase	PGLYRP2	576	37.7	AGLLRPDYALLGHR+DGSPDVTTADIGANTPDATK+EFTEAFLGCPAIHPR+GFGVAIVGNYTAALPTEAALR+GSQTQSHPDLGTEGCWDQLSAPR+GWHWVGAHTLGHNSR+HTASAWLMSAPNSGPHNR+LEPVHLQLQCMSQEQLAQVAANATK+LLQLPLGFLYVHHTYVPAPPCTDFTR+TDCPGDALFDLLR+YHQDTQGWGDIGYSFVVGSDGYVYEGR	11
Q96ST2	1	Protein IWS1 homolog	IWS1	819	7.1	DSDSESEERAEPPASDSENEDVNQHGSDSESEETRK+ENKGEDTEMQNDSFHSDSHMDR+GEDTEMQNDSFHSDSHMDR	3
Q99784	1	Noelin	OLFM1	485	16.5	LDPVSLQTLQTWNTSYPK+MDELRPLIPVLEEYK+SMVDFMNTDNFTSHR+TSGSRFGSWMTDPLAPEGDNRVWYMDGYHNNR	4
Q99969	1	Retinoic acid receptor responder protein 2	RARRES2	163	21.5	EAEEHQETQCLR+GLQVALEEFHK+LVHCPIETQVLR	3
Q9BQI4	0.9297	Coiled-coil domain-containing protein 3	CCDC3	270	10	MDENYNLLPHGVNFQDAIFPDTQENRR	1
Q9BUN1	0.9407	Protein MENT	MENT	341	4.4	TEHKPCTYQQCPCNR	1
Q9BWP8	0.9407	Collectin-11	COLEC11	271	4.8	EGAFVYSDHSPMR	1
Q9BXL7	0.9352	Caspase recruitment domain-containing protein 11	CARD11	1154	2.6	MPGGGPEMDDYMETLKDEEDALWENVECNR	1
Q9BXR6	0.9407	Complement factor H-related protein 5	CFHR5	569	3	GECHVPILEANVDAQPK	1
Q9BXW9	0.9543	Fanconi anemia group D2 protein	FANCD2	1451	3.4	ATEDGEEDEVSAGEKEQDSDESYDDSD+NCLLSCERLQDEEASMGASYSK	2
Q9BY67	0.9407	Cell adhesion molecule 1	CADM1	442	5.4	EGDALELTCEAIGKPQPVMVTWVR	1
Q9BYE9	1	Cadherin-related family member 2	CDHR2	1310	2	TFVIIPELVLPNR+VIPSTGDSEHLFR	2
Q9BYX7	0.9828	Putative beta-actin-like protein 3	POTEKP	375	21.3	AVFPSIVGRPR+CPEALFQPCFLGMESCGIHKTTFNSIVK+IWHHTFYNELR+QEYDESGPSIVHR+QEYDESGPSIVHRK+SYELPDGQVITIGNER	6
Q9C0C6	0.9955	CLOCK-interacting pacemaker	CIPC	399	7.8	DSGFSDGSSECLSSAEQMESEDMLSALGWSR	1
Q9C0F0	0.9975	Putative Polycomb group protein ASXL3	ASXL3	2248	2.5	GCGAFCHDDCIGPSKLCVACLVVR+LKAMIVCKGCGAFCHDDCIGPSK+SEADTTCSNQYNPSNRICWNDDGMR	3
Q9C0F3	0.9136	Zinc finger protein 436	ZNF436	470	4	AHTGEKPYHCNECGENFSR	1
Q9GZQ6	0.9407	Neuropeptide FF receptor 1	NPFFR1	430	3.7	NRSYPLYSCWEAWPEK	1
Q9H079	0.9021	KATNB1-like protein 1	KATNBL1	304	3.289	ISNFTNKNMK	1
Q9H299	0.9025	SH3 domain-binding glutamic acid-rich-like protein 3	SH3BGRL3	93	26.882	IQYQLVDISQDNALR+VYSTSVTGSR	2
Q9H4B7	1	Tubulin beta-1 chain	TUBB1	451	13.1	FWEMIGEEHGIDLAGSDR+GHYTEGAELIENVLEVVR+LGALFQPDSFVHGNSGAGNNWAK	3

Q9H4F1	0.9407	Alpha-N-acetyl-neuraminyl-2,3-beta-galactosyl-1,3-N-acetyl-galactosaminide alpha-2,6-sialyltransferase	ST6GALNAC4	302	5	MMAYCDQIFQDETGK	1
Q9H6S1	0.9032	5-azacytidine-induced protein 2	AZI2	392	4.3	SIPNDGTCFQEHSSYGR	1
Q9HBI1	0.9998	Beta-parvin	PARVB	364	8	LQTVLEAVHDLLRPR+QLEEDLYDGQVLQK	2
Q9HC84	0.9999	Mucin-5B	MUC5B	5762	1	GATGGLCDLTCPPTKVYKPCGPIQPATCNSR+LDGPTEQCPDPLPLPAGNCTDEEGICHR	2
Q9HDC9	1	Adipocyte plasma membrane-associated protein	APMAP	416	9.9	EPPLLLGVLHPNTK+LFENQLVGPESIAHIGDVMFTGTADGR	2
Q9NP31	0.9407	SH2 domain-containing protein 2A	SH2D2A	389	6.9	RSCQNLGYTAASPQAPEAASNTGNAER	1
Q9NP50	0.9985	Protein FAM60A	FAM60A	221	17.6	RHNSDAHSTTSSASPAQSPCYSNQSDDGSDTEMASGSNR	1
Q9NPY3	0.9032	Complement component C1q receptor	CD93	652	4.6	EKAPDVFDWGSSGPLCVSPK+SKEEAQHVQR	2
Q9NQ79	1	Cartilage acidic protein 1	CRTAC1	661	6.7	GDGTFVDAAASAGVDDPHQHGR+IIDGGSGYLCEMEPVAHFGLGK	2
Q9NR09	0.9407	Baculoviral IAP repeat-containing protein 6	BIRC6	4857	0.4	IGLQSTRIGLKLIDILLR	1
Q9NTU7	0.9407	Cerebellin-4	CBLN4	201	6	STNHEPSEMSNK	1
Q9NY93	0.9988	Probable ATP-dependent RNA helicase DDX56	DDX56	547	5.7	FLLLYALLKLSLIR+MEDSEALGFEHMGLDPR	2
Q9NYQ6	1	Cadherin EGF LAG seven-pass G-type receptor 1	CELSR1	3014	1.8	ENGEVLPLKIVTYAAVSLSLAALLVAFVLLSLVRMLR+SLNCNTTFGDGPDMLR	2
Q9NZK5	1	Adenosine deaminase CECR1	CECR1	511	6.1	FVETHPEFIGIK+LLPVYELSGEHHDEEWSVK	2
Q9NZN3	1	EH domain-containing protein 3	EHD3	535	6.9	DKPMYDEIFYTLSPVDGK+LEGHELPNELPAHLLPPSK	2
Q9NZP8	1	Complement C1r subcomponent-like protein	C1RL	487	19.9	DRQDGEEVLQCMPVCGRPVTPIAQNQTTLGSSR+LGNFPWQAFTSIHGR+NQSVNVFLGHTAIDEMLK+QRPEVFSDNMFCVGDETQR+WILTAAHTIYPK	5
Q9NZR2	1	Low-density lipoprotein receptor-related protein 1B	LRP1B	4599	2	CVTKNSSCNAYSEFECGNGECIDYQLTCDGIPHCK+EDDCGDQTDEMASCEFPTCEPLTQFVCKSGRCISSK+WLCDGANDCGSNEDESNQTCTAR	3
Q9NZT1	1	Calmodulin-like protein 5	CALML5	146	26.7	AFDQDGDGHITVDELR+AFSAVDTDGNGTINAQELGAALK	2
Q9P2K9	0.9979	Patched domain-containing protein 2	PTCHD2	1392	3.7	APFGNFTKKLTACMSTVGLLQAASPSR+DLPLGSYSYCSPPSSLMTYFFPTER	2
Q9P2M4	0.9984	TBC1 domain family member 14	TBC1D14	693	2	LDKHNDLGWKLFGK	1
Q9UBW5	1	Bridging integrator 2	BIN2	565	8.7	DERFEQSASNFYQQQAEGHK+FEQSASNFYQQQAEGHK+TSAPPSRPPPPR+TSLEVSPNPEPPEKPVR	4
Q9UBZ9	0.9084	DNA repair protein REV1	REV1	1251	1.4	QLCPNLQAVPYDFHAYK	1
Q9UFB7	0.995	Zinc finger and BTB domain-containing protein 47	ZBTB47	747	4.3	EDGLQRHSDEEEEDDEEEEEEEEEEEGGGSGR+HSDEEEEDDEEEEEEEEEEEGGGSGR	2
Q9UFN0	0.9407	Protein NipSnap homolog 3A	NIPSNAP3A	247	7.3	DKEWQEQFLIPNLALIDK	1
Q9UGM5	1	Fetuin-B	FETUB	382	20.9	GCNDSDVLAVAGFALR+GSVQYLPDLDDKNSQEK+IYMTCPDCPSSIPTDSSNHQVLEAATESLAK+VLYLAAYNCTLRPVSK	4
Q9UHG3	1	Prenylcysteine oxidase 1	PCYOX1	505	8.9	EKEDPEPSTDGTYVWK+LLHALGGDDFLGMLNR+YQSHDYAFSSVEK	3
Q9UK55	0.9407	Protein Z-dependent protease inhibitor	SERPINA10	444	3.6	NLELGLTQGSFAFIHK	1
Q9ULI3	1	Protein HEG homolog 1	HEG1	1381	4.4	ASRWPPPLLLLLLPLLLLPPAAPGTR+NSSGPDLSWLHFYR+SHAASDAPENLTLLAETADAR	3

Q9ULV4	0.9407	Coronin-1C	CORO1C	474	6.1	KSDLFQDDLYPDTAGPEAALEAEEWFEGK	1
Q9UM73	0.9955	ALK tyrosine kinase receptor	ALK	1620	2.4	MALQSSFTCWNGTVLQLGQACDFHQDCAQGEDESQMCR+MALQSSFTCWNGTVLQLGQACDFHQDCAQGEDESQMCRK	2
Q9UN37	0.9407	Vacuolar protein sorting-associated protein 4A	VPS4A	437	4.6	ENQSEGKGSDSDSEGDNPEK	1
Q9UNW1	1	Multiple inositol polyphosphate phosphatase 1	MINPP1	487	2.5	NATALYHVEAFK	1
Q9UPT8	0.9407	Zinc finger CCCH domain-containing protein 4	ZC3H4	1303	2	SYGMYEDYENEQYGEYEGDEEEDMGK	1
Q9Y277	0.9407	Voltage-dependent anion-selective channel protein 3	VDAC3	283	7.1	VNNASLIGLGYTQTLRPGVK	1
Q9Y279	0.9407	V-set and immunoglobulin domain-containing protein 4	VSIG4	399	3.3	GQVGSEQHSDIVK	1
Q9Y2I2	0.9407	Netrin-G1	NTNG1	539	3	CKCNLHATVCVYDNSK	1
Q9Y490	1	Talin-1	TLN1	2541	31.1	[illegible] QAAYLVGVSDPNSQAGQQGLVEPTQFAR+ANQAIQMACQSLGEPGCTQAQVLSAATIVAK+AQEACGPLEMDSALSVVQNLEK+ASVPTIQDQASAMQLSQCAK+AVAEQIPLLVQGVR+AVTDSINQLITMCTQQAPGQK+DKAPGQLECETAIAALNSCLR+DLDQASLAAVSQQLAPR+DPPSWSVLAGHSR+DPVQLNLLYVQAR+EAAYHPEVAPDVR+ELLENPVQPINDMSYFGCLDSVMENSK+EQGVEEHETLLLR+ERIPEAPAGPPSDFGLFLSDDDPK+FGQDFSTFLEAGVEMAGQAPSQEDR+GAAAHPDSEEQQQR+GSQAQPDSPSAQLALIAASQSFLQPGGK+GTPQDLARASGRFGQDFSTFLEAGVEMAGQAPSQEDR+IGITNHDEYSLVR+LGAASLGAEDPETQVVLINAVK+LKPLPGETMEK+LLAALLEDEGGSGRPLLQAAK+LNEAAAGLNQAATELVQASR+MVAAATNNLCEAANAAVQGHASQEK+MVGGIAQIIAAQEEMLR+QAAASATQTIAAAQHAASTPK+SGASGPENFQVGSMPPAQQQITSGQMHR+SNTSPEELGPLANQLTSDYGR+TEDSGLQTQVIAAATQCALSTSQLVACTK+TLAESALQLLYTAK+TLSHPQQMALLDQTK+TMLESAGGLIQTAR+TVTDMLMTICAR+VAGSVTELIQAAEAMK+VEHGSVALPAIMR+VGAIPANALDDGQWSQGLISAAR+VQELGHGCAALVTK+VSHVLAALQAGNR+VSQMAQYFEPLTLAAVGAASK+VVAPTISSPVCQEQLVEAGR	41
Q9Y4F3	0.9955	Meiosis arrest female protein 1	KIAA0430	1742	1.8	MMEGNGTENSCSR+QHCSNKDFSEHEFDPDSYK	2
Q9Y6C2	0.9297	EMILIN-1	EMILIN1	1016	2.5	VSTHDQELGHLNNHHGGSSSSGGSR	1
Q9Y6R7	1	IgGFc-binding protein	FCGBP	5405	1.4	CTCNGATHQVTCR+DPCHGVTCRPQETCK+LEDGVQACHATGCGR	3
P22626	0.8981	Heterogeneous nuclear ribonucleoproteins A2/B1	HNRNPA2B1	353	5.9	NYYEQWGKLTDCVVMRDPASK	1
P23443	0.8981	Ribosomal protein S6 kinase beta-1	RPS6KB1	525	5	AVDWWSLGALMYDMLTGAPPFTGENR	1
P25786	0.8981	Proteasome subunit alpha type-1	PSMA1	263	4.2	FVFDRPLPVSR	1
Q12907	0.8981	Vesicular integral-membrane protein VIP36	LMAN2	356	7.3	LPTGYYFGASAGTGDLSDNHDIISMK	1
Q86W92	0.8939	Liprin-beta-1	PPFIBP1	1011	1.5	DLGQSNSDLDMPFAK	1
Q9NTM9	0.893	Copper homeostasis protein cutC homolog	CUTC	273	8.8	AGAANGFLMEVCVDSVESAVNAER	1
O00622	0.8909	Protein CYR61	CYR61	381	7.3	DGCGCCKVCAK+VTGQCCEEWVCDEDSIK	2
Q14643	0.8899	Inositol 1,4,5-trisphosphate receptor type 1	ITPR1	2758	2.5	CQDMVMAELVNSGEDVLVFYNDRASFQTLIQMMR+GGGFLPMTPMAAAPEGNVKQAEPEKEDIMVMDTK	2

Q5HYK7	0.888	SH3 domain-containing protein 19	SH3D19	790	2	MNIMNTEQSQNSIVSR	1
Q8NGA1	0.888	Olfactory receptor 1M1	OR1M1	313	10.2	AISYPCCLIQMYFFHFFGIVDSVIIAMMAYDR	1
Q96JM7	0.888	Lethal(3)malignant brain tumor-like protein 3	L3MBTL3	780	3.5	TDANESSSSPEIRDQHADDVKEDFEER	1
Q9Y586	0.888	Protein mab-21-like 2	MAB21L2	359	4.2	HPRETDWDESCLGDR	1
Q4KMG0	0.8831	Cell adhesion molecule-related/down-regulated by oncogenes	CDON	1287	1.9	MQCFNEGGESEFSNVMICETK	1
Q14584	0.8782	Zinc finger protein 266	ZNF266	549	6	AFDCSDSGKSFINHSHLQGHLRTHNGESLHEWK	1
P29728	0.8685	2'-5'-oligoadenylate synthase 2	OAS2	719	5.4	VLNESVSFDVLPAFNALGQLSSGSTPSPEVYAGLIDLYK	1
P04083	0.8638	Annexin A1	ANXA1	346	4.6	GLGTDEDTLIEILASR	1
P63252	0.8638	Inward rectifier potassium channel 2	KCNJ2	427	6.6	QDIDNADFEIVVILEGMVEATAMTTQCR	1
Q96DU3	0.8638	SLAM family member 6	SLAMF6	332	9.3	IQYTDTKMILFMVSGICIVFGFIILLLLVLR	1
Q96RW7	0.8638	Hemicentin-1	HMCN1	5635	0.4	TCQDIDECLEQNVHCGPNRMCFNMR	1
Q96ND8	0.8545	Zinc finger protein 583	ZNF583	569	4.7	NQLGSQEVHLSQLIITHKEILPEVQNK	1
Q9ULE0	0.8545	Protein WWC3	WWC3	1092	2.7	LDNESWPSTAEADRDR+QSCRSSLAELMAR	2
O43286	0.8453	Beta-1,4-galactosyltransferase 5	B4GALT5	388	9	DLDWDCLIFHDVDHIPESDRNYYGCGQMPRHFATK	1
Q00G26	0.8453	Perilipin-5	PLIN5	463	6.5	DGSGNGDGDRMGVAGDICEQEPETPSCPVK	1
Q86U42	0.8453	Polyadenylate-binding protein 2	PABPN1	306	9.5	ELQNEVEKQMNMSPPPGNAGPVIMSIEEK	1
O14976	0.8408	Cyclin-G-associated kinase	GAK	1311	1.1	QDHKNVCVVHCMDGR	1
Q9UPU5	0.8408	Ubiquitin carboxyl-terminal hydrolase 24	USP24	2620	0.8	EQSGSSNGSESSPANENGDR	1
O95622	0.8391	Adenylate cyclase type 5	ADCY5	1261	4.4	IQVTTDMYQVLAANTYQLECR+WQFDVWSNDVTLANHMEAGGK+YINEHSFNNFQMK	3
Q04721	0.8375	Neurogenic locus notch homolog protein 2	NOTCH2	2471	2.3	GADCTEDVDECAMANSNPCEHAGK+HCETFVDVCPQMPCLNGGTCAVASNMPDGFICR	2
P22459	0.8364	Potassium voltage-gated channel subfamily A member 4	KCNA4	653	5.8	FYYSEDDHGDECSYTDLLPQDEGGGGYSSVRYSDCCER	1
