# Supplementary material for: Predictive proteomic signatures for response of pancreatic cancer patients receiving chemotherapy
Source: Clin Proteomics. 2019 Jul 17;16:31. doi: 10.1186/s12014-019-9251-3 (PMC6636003; doi:10.1186/s12014-019-9251-3)
Supplement: Supplementary file 4 — Additional file 4: Table S3. The HQ peptide list. [file 12014_2019_9251_MOESM4_ESM.pdf]

Table S3. List of HQ peptides

Protein Name	Protein Description	Protein Gene	Peptide Modified Sequence	Peptide Sequence	Precursor Charge	Precursor m/z
sp|A5A3E0|POTEF_HUMAN	POTE ankyrin domain family member F OS=Homo sapiens GN=POTEF PE=1 SV=2	POTEF	QEYDESGPSIVHR	QEYDESGPSIVHR	2	758.855
sp|O14791|APOL1_HUMAN	Apolipoprotein L1 OS=Homo sapiens GN=APOL1 PE=1 SV=5	APOL1	VTEPISAESGEQVER	VTEPISAESGEQVER	2	815.8996
sp|O14950|ML12B_HUMAN	Myosin regulatory light chain 12B OS=Homo sapiens GN=MYL12B PE=1 SV=2	MYL12B	FTDEEVDELYR	FTDEEVDELYR	2	708.3199
sp|O43866|CD5L_HUMAN	CD5 antigen-like OS=Homo sapiens GN=CD5L PE=1 SV=1	CD5L	ELGC[+57]GAASGTPSGILYEPPAEK	ELGCGAASGTPSGILYEPPAEK	2	1102.531
sp|O60333|KIF1B_HUMAN	Kinesin-like protein KIF1B OS=Homo sapiens GN=KIF1B PE=1 SV=5	KIF1B	IN[+1]DLDLK	INDLDLK	2	416.2266
sp|O75636|FCN3_HUMAN	Ficolin-3 OS=Homo sapiens GN=FCN3 PE=1 SV=2	FCN3	QDGSVDFFR	QDGSVDFFR	2	535.7487
sp|O75636|FCN3_HUMAN	Ficolin-3 OS=Homo sapiens GN=FCN3 PE=1 SV=2	FCN3	LLGEVDHYQLALGK	LLGEVDHYQLALGK	2	778.4276
sp|O75636|FCN3_HUMAN	Ficolin-3 OS=Homo sapiens GN=FCN3 PE=1 SV=2	FCN3	YGIDWASGR	YGIDWASGR	2	512.746
sp|O75882|ATRN_HUMAN	Attractin OS=Homo sapiens GN=ATRN PE=1 SV=2	ATRN	LTGSSGFVTDGPGNYK	LTGSSGFVTDGPGNYK	2	800.3861
sp|O75882|ATRN_HUMAN	Attractin OS=Homo sapiens GN=ATRN PE=1 SV=2	ATRN	C[+57]TWLIEGQPNR	CTWLIEGQPNR	2	687.3352
sp|O75882|ATRN_HUMAN	Attractin OS=Homo sapiens GN=ATRN PE=1 SV=2	ATRN	ISN[+1]SSDTVEC[+57]EC[+57]SENWK	ISNSSDTVECECSENWK	2	1023.407
sp|O75882|ATRN_HUMAN	Attractin OS=Homo sapiens GN=ATRN PE=1 SV=2	ATRN	GEAC[+57]DIPHC[+57]TDNC[+57]GFPHR	GEACDIPHCTDNCGFPHR	3	714.9561
sp|O75882|ATRN_HUMAN	Attractin OS=Homo sapiens GN=ATRN PE=1 SV=2	ATRN	EEYSNLK	EEYSNLK	2	441.7138
sp|O75882|ATRN_HUMAN	Attractin OS=Homo sapiens GN=ATRN PE=1 SV=2	ATRN	SVNNVVVR	SVNNVVVR	2	443.7589
sp|O75882|ATRN_HUMAN	Attractin OS=Homo sapiens GN=ATRN PE=1 SV=2	ATRN	IDSTGN[+1]VTNELR	IDSTGNVTNELR	2	660.3255
sp|O75882|ATRN_HUMAN	Attractin OS=Homo sapiens GN=ATRN PE=1 SV=2	ATRN	C[+57]IN[+1]QSIC[+57]EK	CINQSICEK	2	576.7548
sp|O75882|ATRN_HUMAN	Attractin OS=Homo sapiens GN=ATRN PE=1 SV=2	ATRN	GDEC[+57]QLC[+57]EVENR	GDECQLCEVENR	2	754.8088
sp|O95445|APOM_HUMAN	Apolipoprotein M OS=Homo sapiens GN=APOM PE=1 SV=2	APOM	DGLC[+57]VPR	DGLCVPR	2	408.7053
sp|O95445|APOM_HUMAN	Apolipoprotein M OS=Homo sapiens GN=APOM PE=1 SV=2	APOM	AFLLTPR	AFLLTPR	2	409.2502
sp|P00450|CERU_HUMAN	Ceruloplasmin OS=Homo sapiens GN=CP PE=1 SV=1	CP	DIASGLIGPLIIC[+57]K	DIASGLIGPLIICK	2	735.4234
sp|P00450|CERU_HUMAN	Ceruloplasmin OS=Homo sapiens GN=CP PE=1 SV=1	CP	TYC[+57]SEPEK	TYCSEPEK	2	507.2159
sp|P00450|CERU_HUMAN	Ceruloplasmin OS=Homo sapiens GN=CP PE=1 SV=1	CP	EYTDASFTNR	EYTDASFTNR	2	602.2675
sp|P00450|CERU_HUMAN	Ceruloplasmin OS=Homo sapiens GN=CP PE=1 SV=1	CP	GAYPLSIEPIGVR	GAYPLSIEPIGVR	2	686.3852
sp|P00450|CERU_HUMAN	Ceruloplasmin OS=Homo sapiens GN=CP PE=1 SV=1	CP	NNEGTYYSPNYNPQSR	NNEGTYYSPNYNPQSR	2	952.4139
sp|P00450|CERU_HUMAN	Ceruloplasmin OS=Homo sapiens GN=CP PE=1 SV=1	CP	NNEGTYYSPN[+1]YNPQSR	NNEGTYYSPNYNPQSR	2	952.9059
sp|P00450|CERU_HUMAN	Ceruloplasmin OS=Homo sapiens GN=CP PE=1 SV=1	CP	EVGPTN[+1]ADPVC[+57]LAK	EVGPTNADPVCLAK	2	736.3585
sp|P00450|CERU_HUMAN	Ceruloplasmin OS=Homo sapiens GN=CP PE=1 SV=1	CP	EVGPTNADPVC[+57]LAK	EVGPTNADPVCLAK	2	735.8665
sp|P00450|CERU_HUMAN	Ceruloplasmin OS=Homo sapiens GN=CP PE=1 SV=1	CP	EDEDFQESNK	EDEDFQESNK	2	620.7518
sp|P00450|CERU_HUMAN	Ceruloplasmin OS=Homo sapiens GN=CP PE=1 SV=1	CP	YTVNQC[+57]R	YTVNQCR	2	470.7189
sp|P00450|CERU_HUMAN	Ceruloplasmin OS=Homo sapiens GN=CP PE=1 SV=1	CP	ELHHLQEQN[+1]VSN[+1]AFLDK	ELHHLQEQNVSNAFLDK	2	1012.49
sp|P00450|CERU_HUMAN	Ceruloplasmin OS=Homo sapiens GN=CP PE=1 SV=1	CP	ELHHLQEQN[+1]VSNAFLDK	ELHHLQEQNVSNAFLDK	2	1011.998
sp|P00450|CERU_HUMAN	Ceruloplasmin OS=Homo sapiens GN=CP PE=1 SV=1	CP	GEFYIGSK	GEFYIGSK	2	450.7267
sp|P00450|CERU_HUMAN	Ceruloplasmin OS=Homo sapiens GN=CP PE=1 SV=1	CP	QYTDSTFR	QYTDSTFR	2	509.2354
sp|P00450|CERU_HUMAN	Ceruloplasmin OS=Homo sapiens GN=CP PE=1 SV=1	CP	SGAGTEDSAC[+57]IPWAYYSTVDQVK	SGAGTEDSACIPWAYYSTVDQVK	3	835.7128
sp|P00450|CERU_HUMAN	Ceruloplasmin OS=Homo sapiens GN=CP PE=1 SV=1	CP	DLYSGLIGPLIVC[+57]R	DLYSGLIGPLIVCR	2	788.4318
sp|P00450|CERU_HUMAN	Ceruloplasmin OS=Homo sapiens GN=CP PE=1 SV=1	CP	TYSDHPEK	TYSDHPEK	2	488.7222
sp|P00450|CERU_HUMAN	Ceruloplasmin OS=Homo sapiens GN=CP PE=1 SV=1	CP	DDEEFIESNK	DDEEFIESNK	2	613.2646
sp|P00734|THRB_HUMAN	Prothrombin OS=Homo sapiens GN=F2 PE=1 SV=2	F2	YTAC[+57]ETAR	YTACETAR	2	486.2162
sp|P00734|THRB_HUMAN	Prothrombin OS=Homo sapiens GN=F2 PE=1 SV=2	F2	NPDSSTTGPWC[+57]YTTDPTVR	NPDSSTTGPWCYTTDPTVR	2	1077.973
sp|P00734|THRB_HUMAN	Prothrombin OS=Homo sapiens GN=F2 PE=1 SV=2	F2	GQQYQGR	GQQYQGR	2	418.7041
sp|P00734|THRB_HUMAN	Prothrombin OS=Homo sapiens GN=F2 PE=1 SV=2	F2	LAVTTHGLPC[+57]LAWASAQAK	LAVTTHGLPCLAWASAQAK	2	998.0277
sp|P00734|THRB_HUMAN	Prothrombin OS=Homo sapiens GN=F2 PE=1 SV=2	F2	HQDFNSAVQLVENFC[+57]R	HQDFNSAVQLVENFCR	2	982.4576
sp|P00734|THRB_HUMAN	Prothrombin OS=Homo sapiens GN=F2 PE=1 SV=2	F2	TATSEYQTFFNPR	TATSEYQTFFNPR	3	521.2476
sp|P00734|THRB_HUMAN	Prothrombin OS=Homo sapiens GN=F2 PE=1 SV=2	F2	TFGSGEADC[+57]GLRPLFEK	TFGSGEADCGLRPLFEK	2	942.4515
sp|P00734|THRB_HUMAN	Prothrombin OS=Homo sapiens GN=F2 PE=1 SV=2	F2	KPVAFSDYIHPVC[+57]LPDR	KPVAFSDYIHPVCLPDR	3	672.012
sp|P00734|THRB_HUMAN	Prothrombin OS=Homo sapiens GN=F2 PE=1 SV=2	F2	ETAASLLQAGYK	ETAASLLQAGYK	2	626.3326
sp|P00734|THRB_HUMAN	Prothrombin OS=Homo sapiens GN=F2 PE=1 SV=2	F2	ETWTANVGK	ETWTANVGK	2	503.2536
sp|P00734|THRB_HUMAN	Prothrombin OS=Homo sapiens GN=F2 PE=1 SV=2	F2	ETWTAN[+1]VGK	ETWTANVGK	2	503.7456
sp|P00734|THRB_HUMAN	Prothrombin OS=Homo sapiens GN=F2 PE=1 SV=2	F2	GQPSVLQVVNLPIVERPVC[+57]K	GQPSVLQVVNLPIVERPVCK	2	1116.63
sp|P00736|C1R_HUMAN	Complement C1r subcomponent OS=Homo sapiens GN=C1R PE=1 SV=2	C1R	FC[+57]GQLGSPLGNPPGK	FCGQLGSPLGNPPGK	2	764.8825
sp|P00736|C1R_HUMAN	Complement C1r subcomponent OS=Homo sapiens GN=C1R PE=1 SV=2	C1R	NIGEFC[+57]GK	NIGEFCGK	2	462.7158
sp|P00736|C1R_HUMAN	Complement C1r subcomponent OS=Homo sapiens GN=C1R PE=1 SV=2	C1R	YTTEIIK	YTTEIIK	2	434.2447
sp|P00736|C1R_HUMAN	Complement C1r subcomponent OS=Homo sapiens GN=C1R PE=1 SV=2	C1R	NLPNGDFR	NLPNGDFR	2	466.7329
sp|P00736|C1R_HUMAN	Complement C1r subcomponent OS=Homo sapiens GN=C1R PE=1 SV=2	C1R	IQYYC[+57]HEPYYK	IQYYCHEPYYK	2	782.3505
sp|P00736|C1R_HUMAN	Complement C1r subcomponent OS=Homo sapiens GN=C1R PE=1 SV=2	C1R	ESEQGVYTC[+57]TAQGIWK	ESEQGVYTCTAQGIWK	2	928.9278
sp|P00736|C1R_HUMAN	Complement C1r subcomponent OS=Homo sapiens GN=C1R PE=1 SV=2	C1R	C[+57]LPVC[+57]GKPVNPVEQR	CLPVCGKPVNPVEQR	3	584.9676
sp|P00736|C1R_HUMAN	Complement C1r subcomponent OS=Homo sapiens GN=C1R PE=1 SV=2	C1R	VSVHPDYR	VSVHPDYR	2	486.7485
sp|P00736|C1R_HUMAN	Complement C1r subcomponent OS=Homo sapiens GN=C1R PE=1 SV=2	C1R	QDAC[+57]QGDSGGVFAVR	QDACQGDSGGVFAVR	2	783.8519
sp|P00736|C1R_HUMAN	Complement C1r subcomponent OS=Homo sapiens GN=C1R PE=1 SV=2	C1R	VLNYVDWIK	VLNYVDWIK	2	575.3188
sp|P00739|HPTR_HUMAN	Haptoglobin-related protein OS=Homo sapiens GN=HPR PE=1 SV=2	HPR	TEGDGVYTLNDK	TEGDGVYTLNDK	2	656.3068
sp|P00739|HPTR_HUMAN	Haptoglobin-related protein OS=Homo sapiens GN=HPR PE=1 SV=2	HPR	LPEC[+57]EAVC[+57]GKPK	LPECEAVCGKPK	2	694.3391
sp|P00739|HPTR_HUMAN	Haptoglobin-related protein OS=Homo sapiens GN=HPR PE=1 SV=2	HPR	NPANPVQR	NPANPVQR	2	448.2409
sp|P00739|HPTR_HUMAN	Haptoglobin-related protein OS=Homo sapiens GN=HPR PE=1 SV=2	HPR	GSFPWQAK	GSFPWQAK	2	460.7349
sp|P00739|HPTR_HUMAN	Haptoglobin-related protein OS=Homo sapiens GN=HPR PE=1 SV=2	HPR	NLFLN[+1]HSENATAK	NLFLNHSENATAK	2	730.3624
sp|P00739|HPTR_HUMAN	Haptoglobin-related protein OS=Homo sapiens GN=HPR PE=1 SV=2	HPR	QLVEIEK	QLVEIEK	2	429.7502
sp|P00740|FA9_HUMAN	Coagulation factor IX OS=Homo sapiens GN=F9 PE=1 SV=2	F9	NC[+57]ELDVTC[+57]NIK	NCELDVTCNIK	2	683.3105
sp|P00740|FA9_HUMAN	Coagulation factor IX OS=Homo sapiens GN=F9 PE=1 SV=2	F9	VVC[+57]SC[+57]TEGYR	VVCSCTEGYR	2	615.7657
sp|P00740|FA9_HUMAN	Coagulation factor IX OS=Homo sapiens GN=F9 PE=1 SV=2	F9	VDAFC[+57]GGSIVNEK	VDAFCGGSIVNEK	2	698.3323
sp|P00742|FA10_HUMAN	Coagulation factor X OS=Homo sapiens GN=F10 PE=1 SV=2	F10	NC[+57]ELFTR	NCELFTR	2	470.2213
sp|P00742|FA10_HUMAN	Coagulation factor X OS=Homo sapiens GN=F10 PE=1 SV=2	F10	NTEQEEGGEAVHEVEVVIK	NTEQEEGGEAVHEVEVVIK	3	699.3429
sp|P00742|FA10_HUMAN	Coagulation factor X OS=Homo sapiens GN=F10 PE=1 SV=2	F10	TGIVSGFGR	TGIVSGFGR	2	447.2456
sp|P00742|FA10_HUMAN	Coagulation factor X OS=Homo sapiens GN=F10 PE=1 SV=2	F10	QEDAC[+57]QGDSGGPHVTR	QEDACQGDSGGPHVTR	3	571.913
sp|P00747|PLMN_HUMAN	Plasminogen OS=Homo sapiens GN=PLG PE=1 SV=2	PLG	QLGAGSIEEC[+57]AAK	QLGAGSIEECAAK	2	667.3245
sp|P00747|PLMN_HUMAN	Plasminogen OS=Homo sapiens GN=PLG PE=1 SV=2	PLG	DVVLFEK	DVVLFEK	2	425.2395
sp|P00747|PLMN_HUMAN	Plasminogen OS=Homo sapiens GN=PLG PE=1 SV=2	PLG	VYLSEC[+57]K	VYLSECK	2	449.7206
sp|P00747|PLMN_HUMAN	Plasminogen OS=Homo sapiens GN=PLG PE=1 SV=2	PLG	WELC[+57]DIPR	WELCDIPR	2	544.7633
sp|P00747|PLMN_HUMAN	Plasminogen OS=Homo sapiens GN=PLG PE=1 SV=2	PLG	NLDENYC[+57]R	NLDENYCR	2	542.2298
sp|P00747|PLMN_HUMAN	Plasminogen OS=Homo sapiens GN=PLG PE=1 SV=2	PLG	WEYC[+57]NLK	WEYCNLK	2	506.7315
sp|P00747|PLMN_HUMAN	Plasminogen OS=Homo sapiens GN=PLG PE=1 SV=2	PLG	ATTVTGTPC[+57]QDWAAQEPHR	ATTVTGTPCQDWAAQEPHR	2	1063.49
sp|P00747|PLMN_HUMAN	Plasminogen OS=Homo sapiens GN=PLG PE=1 SV=2	PLG	LFLEPTR	LFLEPTR	2	438.2529
sp|P00747|PLMN_HUMAN	Plasminogen OS=Homo sapiens GN=PLG PE=1 SV=2	PLG	LSSPAVITDK	LSSPAVITDK	2	515.7926
sp|P00747|PLMN_HUMAN	Plasminogen OS=Homo sapiens GN=PLG PE=1 SV=2	PLG	VIPAC[+57]LPSPNYVVADR	VIPACLPSPNYVVADR	3	590.9784
sp|P00747|PLMN_HUMAN	Plasminogen OS=Homo sapiens GN=PLG PE=1 SV=2	PLG	EAQLPVIENK	EAQLPVIENK	2	570.8166
sp|P00748|FA12_HUMAN	Coagulation factor XII OS=Homo sapiens GN=F12 PE=1 SV=3	F12	GRPGPQPWC[+57]ATTPNFDQDQR	GRPGPQPWCATTPNFDQDQR	3	776.6907
sp|P00748|FA12_HUMAN	Coagulation factor XII OS=Homo sapiens GN=F12 PE=1 SV=3	F12	EQPPSLTR	EQPPSLTR	2	464.2483
sp|P00748|FA12_HUMAN	Coagulation factor XII OS=Homo sapiens GN=F12 PE=1 SV=3	F12	N[+1]HSC[+57]EPC[+57]QTLAVR	NHSCEPCQTLAVR	2	786.8483
sp|P00751|CFAB_HUMAN	Complement factor B OS=Homo sapiens GN=CFB PE=1 SV=2	CFB	LLQEGQALEYVC[+57]PSGFYPYPVQTR	LLQEGQALEYVCPSGFYPYPVQTR	3	939.1302
sp|P00751|CFAB_HUMAN	Complement factor B OS=Homo sapiens GN=CFB PE=1 SV=2	CFB	STGSWSTLK	STGSWSTLK	2	483.7482
sp|P00751|CFAB_HUMAN	Complement factor B OS=Homo sapiens GN=CFB PE=1 SV=2	CFB	WSGQTAIC[+57]DN[+1]GAGYC[+57]SNPGIPIGTR	WSGQTAICDNGAGYCSNPGIPIGTR	3	885.0638
sp|P00751|CFAB_HUMAN	Complement factor B OS=Homo sapiens GN=CFB PE=1 SV=2	CFB	WSGQTAIC[+57]DNGAGYC[+57]SNPGIPIGTR	WSGQTAICDNGAGYCSNPGIPIGTR	3	884.7358
sp|P00751|CFAB_HUMAN	Complement factor B OS=Homo sapiens GN=CFB PE=1 SV=2	CFB	C[+57]LVNLIEK	CLVNLIEK	2	494.7784
sp|P00751|CFAB_HUMAN	Complement factor B OS=Homo sapiens GN=CFB PE=1 SV=2	CFB	VASYGVKPR	VASYGVKPR	2	488.7824

sp|P00751|CFAB_HUMAN	Complement factor B OS=Homo sapiens GN=CFB PE=1 SV=2	CFB	QLNEINYEDHK	QLNEINYEDHK	2	701.8335
sp|P00751|CFAB_HUMAN	Complement factor B OS=Homo sapiens GN=CFB PE=1 SV=2	CFB	DLLYIGK	DLLYIGK	2	411.242
sp|P00751|CFAB_HUMAN	Complement factor B OS=Homo sapiens GN=CFB PE=1 SV=2	CFB	YGQTIRPIC[+57]LPC[+57]TEGTTR	YGQTIRPICLPCTEGTTR	2	1062.022
sp|P00751|CFAB_HUMAN	Complement factor B OS=Homo sapiens GN=CFB PE=1 SV=2	CFB	EELLPAQDIK	EELLPAQDIK	2	578.3164
sp|P00751|CFAB_HUMAN	Complement factor B OS=Homo sapiens GN=CFB PE=1 SV=2	CFB	DAQYAPGYDK	DAQYAPGYDK	2	564.2538
sp|P00751|CFAB_HUMAN	Complement factor B OS=Homo sapiens GN=CFB PE=1 SV=2	CFB	DISEVVTPR	DISEVVTPR	2	508.2746
sp|P00751|CFAB_HUMAN	Complement factor B OS=Homo sapiens GN=CFB PE=1 SV=2	CFB	FLC[+57]TGGVSPYADPNTC[+57]R	FLCTGGVSPYADPNTCR	2	957.9273
sp|P00751|CFAB_HUMAN	Complement factor B OS=Homo sapiens GN=CFB PE=1 SV=2	CFB	GDSGGPLIVHK	GDSGGPLIVHK	2	540.2958
sp|P00915|CAH1_HUMAN	Carbonic anhydrase 1 OS=Homo sapiens GN=CA1 PE=1 SV=2	CA1	NGPEQWSK	NGPEQWSK	2	473.2249
sp|P00915|CAH1_HUMAN	Carbonic anhydrase 1 OS=Homo sapiens GN=CA1 PE=1 SV=2	CA1	YSSLAEAASK	YSSLAEAASK	2	513.7587
sp|P00915|CAH1_HUMAN	Carbonic anhydrase 1 OS=Homo sapiens GN=CA1 PE=1 SV=2	CA1	VLDALQAIK	VLDALQAIK	2	485.8002
sp|P01008|ANT3_HUMAN	Antithrombin-III OS=Homo sapiens GN=SERPINC1 PE=1 SV=1	SERPINC1	IPEATNR	IPEATNR	2	400.7167
sp|P01008|ANT3_HUMAN	Antithrombin-III OS=Homo sapiens GN=SERPINC1 PE=1 SV=1	SERPINC1	TSDQIHFFFAK	TSDQIHFFFAK	2	670.8353
sp|P01008|ANT3_HUMAN	Antithrombin-III OS=Homo sapiens GN=SERPINC1 PE=1 SV=1	SERPINC1	LQPLDFK	LQPLDFK	2	430.7475
sp|P01008|ANT3_HUMAN	Antithrombin-III OS=Homo sapiens GN=SERPINC1 PE=1 SV=1	SERPINC1	FSPENTR	FSPENTR	2	425.7063
sp|P01008|ANT3_HUMAN	Antithrombin-III OS=Homo sapiens GN=SERPINC1 PE=1 SV=1	SERPINC1	FSPEN[+1]TR	FSPENTR	2	426.1983
sp|P01008|ANT3_HUMAN	Antithrombin-III OS=Homo sapiens GN=SERPINC1 PE=1 SV=1	SERPINC1	ANRPFLVFIR	ANRPFLVFIR	2	616.8668
sp|P01011|AACT_HUMAN	Alpha-1-antichymotrypsin OS=Homo sapiens GN=SERPINA3 PE=1 SV=2	SERPINA3	EQLSLLDR	EQLSLLDR	2	487.2693
sp|P01011|AACT_HUMAN	Alpha-1-antichymotrypsin OS=Homo sapiens GN=SERPINA3 PE=1 SV=2	SERPINA3	LINDYVK	LINDYVK	2	432.7449
sp|P01011|AACT_HUMAN	Alpha-1-antichymotrypsin OS=Homo sapiens GN=SERPINA3 PE=1 SV=2	SERPINA3	EIGELYLPK	EIGELYLPK	2	531.2975
sp|P01011|AACT_HUMAN	Alpha-1-antichymotrypsin OS=Homo sapiens GN=SERPINA3 PE=1 SV=2	SERPINA3	NLAVSQVVHK	NLAVSQVVHK	2	547.8195
sp|P01019|ANGT_HUMAN	Angiotensinogen OS=Homo sapiens GN=AGT PE=1 SV=1	AGT	DPTFIPAPIQAK	DPTFIPAPIQAK	2	649.3612
sp|P01019|ANGT_HUMAN	Angiotensinogen OS=Homo sapiens GN=AGT PE=1 SV=1	AGT	LQAILGVPWK	LQAILGVPWK	2	562.845
sp|P01019|ANGT_HUMAN	Angiotensinogen OS=Homo sapiens GN=AGT PE=1 SV=1	AGT	QPFVQGLALYTPVVLPR	QPFVQGLALYTPVVLPR	2	949.5486
sp|P01019|ANGT_HUMAN	Angiotensinogen OS=Homo sapiens GN=AGT PE=1 SV=1	AGT	SLDFTELDVAAEK	SLDFTELDVAAEK	3	479.9084
sp|P01024|CO3_HUMAN	Complement C3 OS=Homo sapiens GN=C3 PE=1 SV=2	C3	TIYTPGSTVLYR	TIYTPGSTVLYR	3	457.582
sp|P01024|CO3_HUMAN	Complement C3 OS=Homo sapiens GN=C3 PE=1 SV=2	C3	IFTVNHK	IFTVNHK	2	429.7452
sp|P01024|CO3_HUMAN	Complement C3 OS=Homo sapiens GN=C3 PE=1 SV=2	C3	IFTVN[+1]HK	IFTVNHK	2	430.2373
sp|P01024|CO3_HUMAN	Complement C3 OS=Homo sapiens GN=C3 PE=1 SV=2	C3	FYYIYNEK	FYYIYNEK	2	570.274
sp|P01024|CO3_HUMAN	Complement C3 OS=Homo sapiens GN=C3 PE=1 SV=2	C3	ISLPESLK	ISLPESLK	2	443.7658
sp|P01024|CO3_HUMAN	Complement C3 OS=Homo sapiens GN=C3 PE=1 SV=2	C3	VLLDGVQNPR	VLLDGVQNPR	2	555.8169
sp|P01024|CO3_HUMAN	Complement C3 OS=Homo sapiens GN=C3 PE=1 SV=2	C3	QELSEAEQATR	QELSEAEQATR	2	631.3046
sp|P01024|CO3_HUMAN	Complement C3 OS=Homo sapiens GN=C3 PE=1 SV=2	C3	DSC[+57]VGSLVVK	DSCVGSLVVK	2	532.2762
sp|P01024|CO3_HUMAN	Complement C3 OS=Homo sapiens GN=C3 PE=1 SV=2	C3	VVLVAVDK	VVLVAVDK	2	421.7709
sp|P01024|CO3_HUMAN	Complement C3 OS=Homo sapiens GN=C3 PE=1 SV=2	C3	IWDVVEK	IWDVVEK	2	444.7449
sp|P01024|CO3_HUMAN	Complement C3 OS=Homo sapiens GN=C3 PE=1 SV=2	C3	ADIGC[+57]TPGSGK	ADIGCTPGSGK	2	531.7479
sp|P01024|CO3_HUMAN	Complement C3 OS=Homo sapiens GN=C3 PE=1 SV=2	C3	AELQC[+57]PQPAAR	AELQCPQPAAR	2	620.8088
sp|P01024|CO3_HUMAN	Complement C3 OS=Homo sapiens GN=C3 PE=1 SV=2	C3	ASHLGLAR	ASHLGLAR	2	412.7405
sp|P01024|CO3_HUMAN	Complement C3 OS=Homo sapiens GN=C3 PE=1 SV=2	C3	LPYSVVR	LPYSVVR	2	417.2476
sp|P01024|CO3_HUMAN	Complement C3 OS=Homo sapiens GN=C3 PE=1 SV=2	C3	NEQVEIR	NEQVEIR	2	444.2327
sp|P01024|CO3_HUMAN	Complement C3 OS=Homo sapiens GN=C3 PE=1 SV=2	C3	AVLYNYR	AVLYNYR	2	449.7427
sp|P01024|CO3_HUMAN	Complement C3 OS=Homo sapiens GN=C3 PE=1 SV=2	C3	SSLSVPYVIVPLK	SSLSVPYVIVPLK	2	701.4212
sp|P01024|CO3_HUMAN	Complement C3 OS=Homo sapiens GN=C3 PE=1 SV=2	C3	TGLQEVEVK	TGLQEVEVK	2	501.7769
sp|P01024|CO3_HUMAN	Complement C3 OS=Homo sapiens GN=C3 PE=1 SV=2	C3	QGALELIK	QGALELIK	2	436.266
sp|P01024|CO3_HUMAN	Complement C3 OS=Homo sapiens GN=C3 PE=1 SV=2	C3	QPSSAFAAFVK	QPSSAFAAFVK	2	576.806
sp|P01024|CO3_HUMAN	Complement C3 OS=Homo sapiens GN=C3 PE=1 SV=2	C3	APSTWLTAYVVK	APSTWLTAYVVK	2	668.369
sp|P01024|CO3_HUMAN	Complement C3 OS=Homo sapiens GN=C3 PE=1 SV=2	C3	DIC[+57]EEQVNSLPGSITK	DICEEQVNSLPGSITK	2	895.4355
sp|P01024|CO3_HUMAN	Complement C3 OS=Homo sapiens GN=C3 PE=1 SV=2	C3	VTIKPAPETEK	VTIKPAPETEK	2	606.8454
sp|P01024|CO3_HUMAN	Complement C3 OS=Homo sapiens GN=C3 PE=1 SV=2	C3	N[+1]TLIIYLDK	NTLIIYLDK	2	547.3106
sp|P01024|CO3_HUMAN	Complement C3 OS=Homo sapiens GN=C3 PE=1 SV=2	C3	VHQYFNVELIQPGAVK	VHQYFNVELIQPGAVK	2	921.4991
sp|P01024|CO3_HUMAN	Complement C3 OS=Homo sapiens GN=C3 PE=1 SV=2	C3	AC[+57]EPGVDYVYK	ACEPGVDYVYK	2	650.7975
sp|P01031|CO5_HUMAN	Complement C5 OS=Homo sapiens GN=C5 PE=1 SV=4	C5	FSYSSGHVHLSSENK	FSYSSGHVHLSSENK	3	560.2655
sp|P01031|CO5_HUMAN	Complement C5 OS=Homo sapiens GN=C5 PE=1 SV=4	C5	FQN[+1]SAILTIQPK	FQNSAILTIQPK	2	680.8772
sp|P01031|CO5_HUMAN	Complement C5 OS=Homo sapiens GN=C5 PE=1 SV=4	C5	FQNSAILTIQPK	FQNSAILTIQPK	2	680.3852
sp|P01031|CO5_HUMAN	Complement C5 OS=Homo sapiens GN=C5 PE=1 SV=4	C5	VYSLNDDLKPAK	VYSLNDDLKPAK	2	681.8668
sp|P01031|CO5_HUMAN	Complement C5 OS=Homo sapiens GN=C5 PE=1 SV=4	C5	EYVLPHFSVSIEPEYNFIGYK	EYVLPHFSVSIEPEYNFIGYK	3	844.4213
sp|P01031|CO5_HUMAN	Complement C5 OS=Homo sapiens GN=C5 PE=1 SV=4	C5	NFEITIK	NFEITIK	2	432.7449
sp|P01031|CO5_HUMAN	Complement C5 OS=Homo sapiens GN=C5 PE=1 SV=4	C5	ELSYYSLEDLNNK	ELSYYSLEDLNNK	2	794.3805
sp|P01031|CO5_HUMAN	Complement C5 OS=Homo sapiens GN=C5 PE=1 SV=4	C5	YVLSPYK	YVLSPYK	2	435.242
sp|P01031|CO5_HUMAN	Complement C5 OS=Homo sapiens GN=C5 PE=1 SV=4	C5	LNLVATPLFLKPGIPYPIK	LNLVATPLFLKPGIPYPIK	3	698.7624
sp|P01031|CO5_HUMAN	Complement C5 OS=Homo sapiens GN=C5 PE=1 SV=4	C5	ITHYNYLILSK	ITHYNYLILSK	2	682.8823
sp|P01031|CO5_HUMAN	Complement C5 OS=Homo sapiens GN=C5 PE=1 SV=4	C5	C[+57]C[+57]YDGAC[+57]VNNDETC[+57]EQR	CCYDGACVNNDETCEQR	2	1075.885
sp|P01031|CO5_HUMAN	Complement C5 OS=Homo sapiens GN=C5 PE=1 SV=4	C5	AFTEC[+57]C[+57]VVASQLR	AFTECCVVASQLR	2	770.866
sp|P01031|CO5_HUMAN	Complement C5 OS=Homo sapiens GN=C5 PE=1 SV=4	C5	TLLPVSKPEIR	TLLPVSKPEIR	2	626.8848
sp|P01031|CO5_HUMAN	Complement C5 OS=Homo sapiens GN=C5 PE=1 SV=4	C5	GTVYNYR	GTVYNYR	2	436.7167
sp|P01031|CO5_HUMAN	Complement C5 OS=Homo sapiens GN=C5 PE=1 SV=4	C5	ESYSGVTLDPR	ESYSGVTLDPR	2	612.2988
sp|P01031|CO5_HUMAN	Complement C5 OS=Homo sapiens GN=C5 PE=1 SV=4	C5	IPLDLVPK	IPLDLVPK	2	447.7866
sp|P01031|CO5_HUMAN	Complement C5 OS=Homo sapiens GN=C5 PE=1 SV=4	C5	NADYSYSVWK	NADYSYSVWK	2	616.7828
sp|P01031|CO5_HUMAN	Complement C5 OS=Homo sapiens GN=C5 PE=1 SV=4	C5	LQGTLPVEAR	LQGTLPVEAR	2	542.3115
sp|P01031|CO5_HUMAN	Complement C5 OS=Homo sapiens GN=C5 PE=1 SV=4	C5	AFDIC[+57]PLVK	AFDICPLVK	2	531.7863
sp|P01031|CO5_HUMAN	Complement C5 OS=Homo sapiens GN=C5 PE=1 SV=4	C5	GNPPIYR	GNPPIYR	2	408.7218
sp|P01031|CO5_HUMAN	Complement C5 OS=Homo sapiens GN=C5 PE=1 SV=4	C5	DINYVNPVIK	DINYVNPVIK	2	587.827
sp|P01031|CO5_HUMAN	Complement C5 OS=Homo sapiens GN=C5 PE=1 SV=4	C5	WLSEEQR	WLSEEQR	2	474.2327
sp|P01031|CO5_HUMAN	Complement C5 OS=Homo sapiens GN=C5 PE=1 SV=4	C5	TSTSEEVC[+57]SFYLK	TSTSEEVCSFYLK	2	775.8558
sp|P01031|CO5_HUMAN	Complement C5 OS=Homo sapiens GN=C5 PE=1 SV=4	C5	IDTQDIEASHYR	IDTQDIEASHYR	2	724.3442
sp|P01031|CO5_HUMAN	Complement C5 OS=Homo sapiens GN=C5 PE=1 SV=4	C5	IVAC[+57]ASYKPSR	IVACASYKPSR	2	626.3293
sp|P01031|CO5_HUMAN	Complement C5 OS=Homo sapiens GN=C5 PE=1 SV=4	C5	QTAC[+57]KPEIAYAYK	QTACKPEIAYAYK	2	771.8847
sp|P01031|CO5_HUMAN	Complement C5 OS=Homo sapiens GN=C5 PE=1 SV=4	C5	ATLLDIYK	ATLLDIYK	2	468.7737
sp|P01031|CO5_HUMAN	Complement C5 OS=Homo sapiens GN=C5 PE=1 SV=4	C5	DSEITFIK	DSEITFIK	2	476.7529
sp|P01031|CO5_HUMAN	Complement C5 OS=Homo sapiens GN=C5 PE=1 SV=4	C5	VTC[+57]TNAELVK	VTCTNAELVK	2	567.7948
sp|P01042|KNG1_HUMAN	Kininogen-1 OS=Homo sapiens GN=KNG1 PE=1 SV=2	KNG1	QVVAGLNFR	QVVAGLNFR	2	502.2878
sp|P01042|KNG1_HUMAN	Kininogen-1 OS=Homo sapiens GN=KNG1 PE=1 SV=2	KNG1	ITYSIVQTN[+1]C[+57]SK	ITYSIVQTNCSK	2	707.8478
sp|P01042|KNG1_HUMAN	Kininogen-1 OS=Homo sapiens GN=KNG1 PE=1 SV=2	KNG1	ENFLFLTPDC[+57]K	ENFLFLTPDCK	2	692.3343
sp|P01042|KNG1_HUMAN	Kininogen-1 OS=Homo sapiens GN=KNG1 PE=1 SV=2	KNG1	IASFSQNC[+57]DIYPGK	IASFSQNCDIYPGK	2	800.3772
sp|P01042|KNG1_HUMAN	Kininogen-1 OS=Homo sapiens GN=KNG1 PE=1 SV=2	KNG1	IC[+57]VGC[+57]PR	ICVGCPR	2	431.2071
sp|P01042|KNG1_HUMAN	Kininogen-1 OS=Homo sapiens GN=KNG1 PE=1 SV=2	KNG1	YFIDFVAR	YFIDFVAR	2	515.7715
sp|P01042|KNG1_HUMAN	Kininogen-1 OS=Homo sapiens GN=KNG1 PE=1 SV=2	KNG1	ESNEELTESC[+57]ETK	ESNEELTESCETK	2	778.3251
sp|P01344|IGF2_HUMAN	Insulin-like growth factor II OS=Homo sapiens GN=IGF2 PE=1 SV=1	IGF2	GIVEEC[+57]C[+57]FR	GIVEECCFR	2	585.2575
sp|P02042|HBD_HUMAN	Hemoglobin subunit delta OS=Homo sapiens GN=HBD PE=1 SV=2	HBD	LLVVYPWTQR	LLVVYPWTQR	2	637.8664
sp|P02042|HBD_HUMAN	Hemoglobin subunit delta OS=Homo sapiens GN=HBD PE=1 SV=2	HBD	GTFSQLSELHC[+57]DK	GTFSQLSELHCDK	2	761.3537
sp|P02042|HBD_HUMAN	Hemoglobin subunit delta OS=Homo sapiens GN=HBD PE=1 SV=2	HBD	LHVDPENFR	LHVDPENFR	2	563.7856
sp|P02042|HBD_HUMAN	Hemoglobin subunit delta OS=Homo sapiens GN=HBD PE=1 SV=2	HBD	VVAGVANALAHK	VVAGVANALAHK	2	575.3406
sp|P02649|APOE_HUMAN	Apolipoprotein E OS=Homo sapiens GN=APOE PE=1 SV=1	APOE	WELALGR	WELALGR	2	422.7374
sp|P02649|APOE_HUMAN	Apolipoprotein E OS=Homo sapiens GN=APOE PE=1 SV=1	APOE	SELEEQLTPVAEETR	SELEEQLTPVAEETR	2	865.9258
sp|P02649|APOE_HUMAN	Apolipoprotein E OS=Homo sapiens GN=APOE PE=1 SV=1	APOE	DADDLQK	DADDLQK	2	402.6903
sp|P02649|APOE_HUMAN	Apolipoprotein E OS=Homo sapiens GN=APOE PE=1 SV=1	APOE	LAVYQAGAR	LAVYQAGAR	2	474.7667
sp|P02649|APOE_HUMAN	Apolipoprotein E OS=Homo sapiens GN=APOE PE=1 SV=1	APOE	AATVGSLAGQPLQER	AATVGSLAGQPLQER	2	749.4046
sp|P02649|APOE_HUMAN	Apolipoprotein E OS=Homo sapiens GN=APOE PE=1 SV=1	APOE	AQAWGER	AQAWGER	2	409.2012

sp|P02649|APOE_HUMAN	Apolipoprotein E OS=Homo sapiens GN=APOE PE=1 SV=1	APOE	EQVAEVR	EQVAEVR	2	415.722
sp|P02649|APOE_HUMAN	Apolipoprotein E OS=Homo sapiens GN=APOE PE=1 SV=1	APOE	LEEQAQQIR	LEEQAQQIR	2	557.7962
sp|P02649|APOE_HUMAN	Apolipoprotein E OS=Homo sapiens GN=APOE PE=1 SV=1	APOE	LQAEAFQAR	LQAEAFQAR	2	517.2749
sp|P02649|APOE_HUMAN	Apolipoprotein E OS=Homo sapiens GN=APOE PE=1 SV=1	APOE	QWAGLVEK	QWAGLVEK	2	465.7558
sp|P02649|APOE_HUMAN	Apolipoprotein E OS=Homo sapiens GN=APOE PE=1 SV=1	APOE	VQAAVGTSAAPVPSDNH	VQAAVGTSAAPVPSDNH	2	810.9025
sp|P02654|APOC1_HUMAN	Apolipoprotein C-I OS=Homo sapiens GN=APOC1 PE=1 SV=1	APOC1	EFGNTLEDK	EFGNTLEDK	2	526.7484
sp|P02654|APOC1_HUMAN	Apolipoprotein C-I OS=Homo sapiens GN=APOC1 PE=1 SV=1	APOC1	EFGN[+1]TLEDK	EFGNTLEDK	2	527.2404
sp|P02655|APOC2_HUMAN	Apolipoprotein C-II OS=Homo sapiens GN=APOC2 PE=1 SV=1	APOC2	ESLSSYWESAK	ESLSSYWESAK	2	643.7986
sp|P02655|APOC2_HUMAN	Apolipoprotein C-II OS=Homo sapiens GN=APOC2 PE=1 SV=1	APOC2	TYLPAVDEK	TYLPAVDEK	2	518.2715
sp|P02656|APOC3_HUMAN	Apolipoprotein C-III OS=Homo sapiens GN=APOC3 PE=1 SV=1	APOC3	DYWSTVK	DYWSTVK	2	449.7189
sp|P02741|CRP_HUMAN	C-reactive protein OS=Homo sapiens GN=CRP PE=1 SV=1	CRP	ESDTSYVSLK	ESDTSYVSLK	2	564.7746
sp|P02741|CRP_HUMAN	C-reactive protein OS=Homo sapiens GN=CRP PE=1 SV=1	CRP	GYSIFSYATK	GYSIFSYATK	2	568.7848
sp|P02743|SAMP_HUMAN	Serum amyloid P-component OS=Homo sapiens GN=APCS PE=1 SV=2	APCS	AYSDLSR	AYSDLSR	2	406.2009
sp|P02743|SAMP_HUMAN	Serum amyloid P-component OS=Homo sapiens GN=APCS PE=1 SV=2	APCS	AYSLFSYNTQGR	AYSLFSYNTQGR	2	703.8386
sp|P02743|SAMP_HUMAN	Serum amyloid P-component OS=Homo sapiens GN=APCS PE=1 SV=2	APCS	DNELLVYK	DNELLVYK	2	497.2662
sp|P02743|SAMP_HUMAN	Serum amyloid P-component OS=Homo sapiens GN=APCS PE=1 SV=2	APCS	VGEYSLYIGR	VGEYSLYIGR	2	578.8035
sp|P02743|SAMP_HUMAN	Serum amyloid P-component OS=Homo sapiens GN=APCS PE=1 SV=2	APCS	QGYFVEAQPK	QGYFVEAQPK	2	583.7957
sp|P02743|SAMP_HUMAN	Serum amyloid P-component OS=Homo sapiens GN=APCS PE=1 SV=2	APCS	IVLGQEQDSYGGK	IVLGQEQDSYGGK	2	697.3515
sp|P02743|SAMP_HUMAN	Serum amyloid P-component OS=Homo sapiens GN=APCS PE=1 SV=2	APCS	GYVIIKPLVWV	GYVIIKPLVWV	2	643.8972
sp|P02746|C1QB_HUMAN	Complement C1q subcomponent subunit B OS=Homo sapiens GN=C1QB PE=1 SV=3	C1QB	TINVPLR	TINVPLR	2	406.7531
sp|P02747|C1QC_HUMAN	Complement C1q subcomponent subunit C OS=Homo sapiens GN=C1QC PE=1 SV=3	C1QC	FQSVFTVTR	FQSVFTVTR	2	542.7929
sp|P02747|C1QC_HUMAN	Complement C1q subcomponent subunit C OS=Homo sapiens GN=C1QC PE=1 SV=3	C1QC	QTHQPPAPNSLIR	QTHQPPAPNSLIR	2	729.8942
sp|P02747|C1QC_HUMAN	Complement C1q subcomponent subunit C OS=Homo sapiens GN=C1QC PE=1 SV=3	C1QC	FNAVLTNPQGDYDTSTGK	FNAVLTNPQGDYDTSTGK	2	964.4552
sp|P02747|C1QC_HUMAN	Complement C1q subcomponent subunit C OS=Homo sapiens GN=C1QC PE=1 SV=3	C1QC	TNQVNSGGVLLR	TNQVNSGGVLLR	2	629.3491
sp|P02748|CO9_HUMAN	Complement component C9 OS=Homo sapiens GN=C9 PE=1 SV=2	C9	VVEESELAR	VVEESELAR	2	516.272
sp|P02748|CO9_HUMAN	Complement component C9 OS=Homo sapiens GN=C9 PE=1 SV=2	C9	DGNTLTYYR	DGNTLTYYR	2	551.7618
sp|P02748|CO9_HUMAN	Complement component C9 OS=Homo sapiens GN=C9 PE=1 SV=2	C9	TEHYEEQIEAFK	TEHYEEQIEAFK	2	762.3543
sp|P02748|CO9_HUMAN	Complement component C9 OS=Homo sapiens GN=C9 PE=1 SV=2	C9	ALPTTYEK	ALPTTYEK	2	461.7477
sp|P02748|CO9_HUMAN	Complement component C9 OS=Homo sapiens GN=C9 PE=1 SV=2	C9	LSPIYNLVPVK	LSPIYNLVPVK	2	621.8765
sp|P02748|CO9_HUMAN	Complement component C9 OS=Homo sapiens GN=C9 PE=1 SV=2	C9	AIEDYINEFSVR	AIEDYINEFSVR	3	485.9087
sp|P02748|CO9_HUMAN	Complement component C9 OS=Homo sapiens GN=C9 PE=1 SV=2	C9	C[+57]LC[+57]AC[+57]PFK	CLCACPFK	2	528.2272
sp|P02748|CO9_HUMAN	Complement component C9 OS=Homo sapiens GN=C9 PE=1 SV=2	C9	FEGIAC[+57]EISK	FEGIACEISK	2	577.2815
sp|P02749|APOH_HUMAN	Beta-2-glycoprotein 1 OS=Homo sapiens GN=APOH PE=1 SV=3	APOH	VYKPSAGN[+1]NSLYR	VYKPSAGNNSLYR	2	735.3728
sp|P02749|APOH_HUMAN	Beta-2-glycoprotein 1 OS=Homo sapiens GN=APOH PE=1 SV=3	APOH	C[+57]PFPSRPDNGFVNYPAKPTLYYK	CPFPSRPDNGFVNYPAKPTLYYK	3	911.1161
sp|P02749|APOH_HUMAN	Beta-2-glycoprotein 1 OS=Homo sapiens GN=APOH PE=1 SV=3	APOH	ATVVYQGER	ATVVYQGER	2	511.7669
sp|P02749|APOH_HUMAN	Beta-2-glycoprotein 1 OS=Homo sapiens GN=APOH PE=1 SV=3	APOH	EHSSLAFWK	EHSSLAFWK	2	552.7773
sp|P02750|A2GL_HUMAN	Leucine-rich alpha-2-glycoprotein OS=Homo sapiens GN=LRG1 PE=1 SV=2	LRG1	LQELHLSSNGLESLSPEFLRPVPQLR	LQELHLSSNGLESLSPEFLRPVPQLR	3	987.2048
sp|P02750|A2GL_HUMAN	Leucine-rich alpha-2-glycoprotein OS=Homo sapiens GN=LRG1 PE=1 SV=2	LRG1	ALGHLDLSGNR	ALGHLDLSGNR	2	576.8096
sp|P02750|A2GL_HUMAN	Leucine-rich alpha-2-glycoprotein OS=Homo sapiens GN=LRG1 PE=1 SV=2	LRG1	GPLQLER	GPLQLER	2	406.7349
sp|P02750|A2GL_HUMAN	Leucine-rich alpha-2-glycoprotein OS=Homo sapiens GN=LRG1 PE=1 SV=2	LRG1	DLLLPQPDLR	DLLLPQPDLR	2	590.3402
sp|P02750|A2GL_HUMAN	Leucine-rich alpha-2-glycoprotein OS=Homo sapiens GN=LRG1 PE=1 SV=2	LRG1	YLFLNGNK	YLFLNGNK	2	484.7636
sp|P02750|A2GL_HUMAN	Leucine-rich alpha-2-glycoprotein OS=Homo sapiens GN=LRG1 PE=1 SV=2	LRG1	DGFDISGNPWIC[+57]DQNLSDLYR	DGFDISGNPWICDQNLSDLYR	3	829.0411
sp|P02750|A2GL_HUMAN	Leucine-rich alpha-2-glycoprotein OS=Homo sapiens GN=LRG1 PE=1 SV=2	LRG1	GQTLLAVAK	GQTLLAVAK	2	450.7793
sp|P02753|RET4_HUMAN	Retinol-binding protein 4 OS=Homo sapiens GN=RBP4 PE=1 SV=3	RBP4	YWGVASFLQK	YWGVASFLQK	2	599.8164
sp|P02753|RET4_HUMAN	Retinol-binding protein 4 OS=Homo sapiens GN=RBP4 PE=1 SV=3	RBP4	QEELC[+57]LAR	QEELCLAR	2	509.7529
sp|P02760|AMBP_HUMAN	Protein AMBP OS=Homo sapiens GN=AMBP PE=1 SV=1	AMBP	ETLLQDFR	ETLLQDFR	2	511.2693
sp|P02760|AMBP_HUMAN	Protein AMBP OS=Homo sapiens GN=AMBP PE=1 SV=1	AMBP	GEC[+57]VPGEQEPEPILIPR	GECVPGEQEPEPILIPR	2	960.4802
sp|P02760|AMBP_HUMAN	Protein AMBP OS=Homo sapiens GN=AMBP PE=1 SV=1	AMBP	EC[+57]LQTC[+57]R	ECLQTCR	2	483.7102
sp|P02760|AMBP_HUMAN	Protein AMBP OS=Homo sapiens GN=AMBP PE=1 SV=1	AMBP	TVAAC[+57]NLPIVR	TVAACNLPIVR	2	607.3397
sp|P02760|AMBP_HUMAN	Protein AMBP OS=Homo sapiens GN=AMBP PE=1 SV=1	AMBP	C[+57]VLFPYGGC[+57]QGNGNK	CVLFPYGGCQGNGNK	2	835.8743
sp|P02765|FETUA_HUMAN	Alpha-2-HS-glycoprotein OS=Homo sapiens GN=AHSG PE=1 SV=1	AHSG	C[+57]NLLAEK	CNLLAEK	2	424.2207
sp|P02766|TTHY_HUMAN	Transthyretin OS=Homo sapiens GN=TTR PE=1 SV=1	TTR	YTIAALLSPYSYSTTAVVTNPK	YTIAALLSPYSYSTTAVVTNPK	2	1180.623
sp|P02774|VTDB_HUMAN	Vitamin D-binding protein OS=Homo sapiens GN=GC PE=1 SV=1	GC	EFSHLGK	EFSHLGK	2	409.2138
sp|P02774|VTDB_HUMAN	Vitamin D-binding protein OS=Homo sapiens GN=GC PE=1 SV=1	GC	ELPEHTVK	ELPEHTVK	2	476.7585
sp|P02774|VTDB_HUMAN	Vitamin D-binding protein OS=Homo sapiens GN=GC PE=1 SV=1	GC	DVC[+57]DPGNTK	DVCDPGNTK	2	503.2189
sp|P02774|VTDB_HUMAN	Vitamin D-binding protein OS=Homo sapiens GN=GC PE=1 SV=1	GC	YTFELSR	YTFELSR	2	458.2322
sp|P02774|VTDB_HUMAN	Vitamin D-binding protein OS=Homo sapiens GN=GC PE=1 SV=1	GC	THLPEVFLSK	THLPEVFLSK	2	585.8295
sp|P02774|VTDB_HUMAN	Vitamin D-binding protein OS=Homo sapiens GN=GC PE=1 SV=1	GC	VLEPTLK	VLEPTLK	2	400.2498
sp|P02774|VTDB_HUMAN	Vitamin D-binding protein OS=Homo sapiens GN=GC PE=1 SV=1	GC	ELSSFIDK	ELSSFIDK	2	469.7451
sp|P02775|CXCL7_HUMAN	Platelet basic protein OS=Homo sapiens GN=PPBP PE=1 SV=3	PPBP	IC[+57]LDPDAPR	ICLDPDAPR	2	528.7608
sp|P02790|HEMO_HUMAN	Hemopexin OS=Homo sapiens GN=HPX PE=1 SV=2	HPX	N[+1]FPSPVDAAFR	NFPSPVDAAFR	2	611.2986
sp|P02790|HEMO_HUMAN	Hemopexin OS=Homo sapiens GN=HPX PE=1 SV=2	HPX	GGYTLVSGYPK	GGYTLVSGYPK	2	571.298
sp|P02790|HEMO_HUMAN	Hemopexin OS=Homo sapiens GN=HPX PE=1 SV=2	HPX	LWWLDLK	LWWLDLK	2	487.2789
sp|P03951|FA11_HUMAN	Coagulation factor XI OS=Homo sapiens GN=F11 PE=1 SV=1	F11	IVGGTASVR	IVGGTASVR	2	430.2534
sp|P03951|FA11_HUMAN	Coagulation factor XI OS=Homo sapiens GN=F11 PE=1 SV=1	F11	VYSGILN[+1]QSEIK	VYSGILNQSEIK	2	676.3588
sp|P03952|KLKB1_HUMAN	Plasma kallikrein OS=Homo sapiens GN=KLKB1 PE=1 SV=1	KLKB1	DSVTGTLPK	DSVTGTLPK	2	459.2506
sp|P03952|KLKB1_HUMAN	Plasma kallikrein OS=Homo sapiens GN=KLKB1 PE=1 SV=1	KLKB1	GVNFN[+1]VSK	GVNFNVSK	2	433.2243
sp|P03952|KLKB1_HUMAN	Plasma kallikrein OS=Homo sapiens GN=KLKB1 PE=1 SV=1	KLKB1	YSPGGTPTAIK	YSPGGTPTAIK	2	546.2902
sp|P03952|KLKB1_HUMAN	Plasma kallikrein OS=Homo sapiens GN=KLKB1 PE=1 SV=1	KLKB1	VLTPDAFVC[+57]R	VLTPDAFVCR	2	589.3053
sp|P03952|KLKB1_HUMAN	Plasma kallikrein OS=Homo sapiens GN=KLKB1 PE=1 SV=1	KLKB1	GVNVC[+57]QETC[+57]TK	GVNVCQETCTK	2	648.2896
sp|P03952|KLKB1_HUMAN	Plasma kallikrein OS=Homo sapiens GN=KLKB1 PE=1 SV=1	KLKB1	GVN[+1]VC[+57]QETC[+57]TK	GVNVCQETCTK	2	648.7816
sp|P03952|KLKB1_HUMAN	Plasma kallikrein OS=Homo sapiens GN=KLKB1 PE=1 SV=1	KLKB1	IYSGILN[+1]LSDITK	IYSGILNLSDITK	2	719.3954
sp|P03952|KLKB1_HUMAN	Plasma kallikrein OS=Homo sapiens GN=KLKB1 PE=1 SV=1	KLKB1	DTPFSQIK	DTPFSQIK	2	468.2453
sp|P03952|KLKB1_HUMAN	Plasma kallikrein OS=Homo sapiens GN=KLKB1 PE=1 SV=1	KLKB1	EIIIHQNYK	EIIIHQNYK	2	579.3193
sp|P03952|KLKB1_HUMAN	Plasma kallikrein OS=Homo sapiens GN=KLKB1 PE=1 SV=1	KLKB1	LQAPLN[+1]YTEFQKPIC[+57]LPSK	LQAPLNYTEFQKPICLPSK	2	1124.588
sp|P03952|KLKB1_HUMAN	Plasma kallikrein OS=Homo sapiens GN=KLKB1 PE=1 SV=1	KLKB1	GEIQNILQK	GEIQNILQK	2	521.7982
sp|P03952|KLKB1_HUMAN	Plasma kallikrein OS=Homo sapiens GN=KLKB1 PE=1 SV=1	KLKB1	EQPGVYTK	EQPGVYTK	2	461.2375
sp|P04003|C4BPA_HUMAN	C4b-binding protein alpha chain OS=Homo sapiens GN=C4BPA PE=1 SV=2	C4BPA	YTC[+57]LPGYVR	YTCLPGYVR	2	564.779
sp|P04003|C4BPA_HUMAN	C4b-binding protein alpha chain OS=Homo sapiens GN=C4BPA PE=1 SV=2	C4BPA	GSSVIHC[+57]DADSK	GSSVIHCDADSK	2	638.2853
sp|P04003|C4BPA_HUMAN	C4b-binding protein alpha chain OS=Homo sapiens GN=C4BPA PE=1 SV=2	C4BPA	C[+57]HPGYKPTTDEPTTVIC[+57]QK	CHPGYKPTTDEPTTVICQK	3	744.6856
sp|P04003|C4BPA_HUMAN	C4b-binding protein alpha chain OS=Homo sapiens GN=C4BPA PE=1 SV=2	C4BPA	WTPYQGC[+57]EALC[+57]C[+57]PEPK	WTPYQGCEALCCPEPK	2	998.4235

sp|P04003|C4BPA_HUMAN	C4b-binding protein alpha chain OS=Homo sapiens GN=C4BPA PE=1 SV=2	C4BPA	LNNGEITQHR	LNNGEITQHR	2	591.3047
sp|P04003|C4BPA_HUMAN	C4b-binding protein alpha chain OS=Homo sapiens GN=C4BPA PE=1 SV=2	C4BPA	EEIIYEC[+57]DK	EEIIYECDK	2	599.7685
sp|P04003|C4BPA_HUMAN	C4b-binding protein alpha chain OS=Homo sapiens GN=C4BPA PE=1 SV=2	C4BPA	GYILVGQAK	GYILVGQAK	2	474.7793
sp|P04003|C4BPA_HUMAN	C4b-binding protein alpha chain OS=Homo sapiens GN=C4BPA PE=1 SV=2	C4BPA	LSC[+57]SYSHWSAPAPQC[+57]K	LSCSYSHWSAPAPQCK	2	939.9167
sp|P04003|C4BPA_HUMAN	C4b-binding protein alpha chain OS=Homo sapiens GN=C4BPA PE=1 SV=2	C4BPA	DQYVEPEN[+1]VTIQC[+57]DSGYGVVGPQSITC[+57]SGN[+1]R	DQYVEPENVTIQCDSGYGVVGPQSITCSGNR	3	1144.505
sp|P04003|C4BPA_HUMAN	C4b-binding protein alpha chain OS=Homo sapiens GN=C4BPA PE=1 SV=2	C4BPA	TWYPEVPK	TWYPEVPK	2	510.2635
sp|P04003|C4BPA_HUMAN	C4b-binding protein alpha chain OS=Homo sapiens GN=C4BPA PE=1 SV=2	C4BPA	LSLEIEQLELQR	LSLEIEQLELQR	2	735.9118
sp|P04004|VTNC_HUMAN	Vitronectin OS=Homo sapiens GN=VTN PE=1 SV=1	VTN	C[+57]TEGFNVDK	CTEGFNVDK	2	535.2346
sp|P04004|VTNC_HUMAN	Vitronectin OS=Homo sapiens GN=VTN PE=1 SV=1	VTN	AVRPGYPK	AVRPGYPK	2	444.2585
sp|P04004|VTNC_HUMAN	Vitronectin OS=Homo sapiens GN=VTN PE=1 SV=1	VTN	DVWGIEGPIDAAFTR	DVWGIEGPIDAAFTR	2	823.9123
sp|P04004|VTNC_HUMAN	Vitronectin OS=Homo sapiens GN=VTN PE=1 SV=1	VTN	QPQFISR	QPQFISR	2	438.2403
sp|P04004|VTNC_HUMAN	Vitronectin OS=Homo sapiens GN=VTN PE=1 SV=1	VTN	SIAQYWLGC[+57]PAPGHL	SIAQYWLGCPAPGHL	2	835.4114
sp|P04070|PROC_HUMAN	Vitamin K-dependent protein C OS=Homo sapiens GN=PROC PE=1 SV=1	PROC	DTEDQEDQVDPR	DTEDQEDQVDPR	2	723.8026
sp|P04070|PROC_HUMAN	Vitamin K-dependent protein C OS=Homo sapiens GN=PROC PE=1 SV=1	PROC	EVFVHPN[+1]YSK	EVFVHPNYSK	2	610.8009
sp|P04114|APOB_HUMAN	Apolipoprotein B-100 OS=Homo sapiens GN=APOB PE=1 SV=2	APOB	YTYNYEAESSSGVPGTADSR	YTYNYEAESSSGVPGTADSR	2	1077.467
sp|P04114|APOB_HUMAN	Apolipoprotein B-100 OS=Homo sapiens GN=APOB PE=1 SV=2	APOB	EVYGFNPEGK	EVYGFNPEGK	2	570.272
sp|P04114|APOB_HUMAN	Apolipoprotein B-100 OS=Homo sapiens GN=APOB PE=1 SV=2	APOB	QVFLYPEK	QVFLYPEK	2	512.2791
sp|P04114|APOB_HUMAN	Apolipoprotein B-100 OS=Homo sapiens GN=APOB PE=1 SV=2	APOB	DEPTYILNIK	DEPTYILNIK	2	603.3243
sp|P04114|APOB_HUMAN	Apolipoprotein B-100 OS=Homo sapiens GN=APOB PE=1 SV=2	APOB	GIISALLVPPETEEAK	GIISALLVPPETEEAK	2	833.9667
sp|P04114|APOB_HUMAN	Apolipoprotein B-100 OS=Homo sapiens GN=APOB PE=1 SV=2	APOB	GNVATEISTER	GNVATEISTER	2	588.7964
sp|P04114|APOB_HUMAN	Apolipoprotein B-100 OS=Homo sapiens GN=APOB PE=1 SV=2	APOB	DLGQC[+57]DR	DLGQCDR	2	432.1874
sp|P04114|APOB_HUMAN	Apolipoprotein B-100 OS=Homo sapiens GN=APOB PE=1 SV=2	APOB	TGISPLALIK	TGISPLALIK	2	506.8237
sp|P04114|APOB_HUMAN	Apolipoprotein B-100 OS=Homo sapiens GN=APOB PE=1 SV=2	APOB	EQHLFLPFSYK	EQHLFLPFSYK	2	704.8666
sp|P04114|APOB_HUMAN	Apolipoprotein B-100 OS=Homo sapiens GN=APOB PE=1 SV=2	APOB	LTISEQNIQR	LTISEQNIQR	2	601.3304
sp|P04114|APOB_HUMAN	Apolipoprotein B-100 OS=Homo sapiens GN=APOB PE=1 SV=2	APOB	ATLYALSHAVNNYHK	ATLYALSHAVNNYHK	2	851.439
sp|P04114|APOB_HUMAN	Apolipoprotein B-100 OS=Homo sapiens GN=APOB PE=1 SV=2	APOB	DQEVLLQTFLDDASPGDK	DQEVLLQTFLDDASPGDK	2	995.9838
sp|P04114|APOB_HUMAN	Apolipoprotein B-100 OS=Homo sapiens GN=APOB PE=1 SV=2	APOB	IVQILPWEQNEQVK	IVQILPWEQNEQVK	2	862.4725
sp|P04114|APOB_HUMAN	Apolipoprotein B-100 OS=Homo sapiens GN=APOB PE=1 SV=2	APOB	SVSLPSLDPASAK	SVSLPSLDPASAK	2	636.3457
sp|P04114|APOB_HUMAN	Apolipoprotein B-100 OS=Homo sapiens GN=APOB PE=1 SV=2	APOB	IEGNLIFDPNNYLPK	IEGNLIFDPNNYLPK	2	873.9567
sp|P04114|APOB_HUMAN	Apolipoprotein B-100 OS=Homo sapiens GN=APOB PE=1 SV=2	APOB	ALYWVNGQVPDGVSK	ALYWVNGQVPDGVSK	2	816.9227
sp|P04114|APOB_HUMAN	Apolipoprotein B-100 OS=Homo sapiens GN=APOB PE=1 SV=2	APOB	VLVDHFGYTK	VLVDHFGYTK	2	589.8139
sp|P04114|APOB_HUMAN	Apolipoprotein B-100 OS=Homo sapiens GN=APOB PE=1 SV=2	APOB	FIIPSPK	FIIPSPK	2	401.2471
sp|P04114|APOB_HUMAN	Apolipoprotein B-100 OS=Homo sapiens GN=APOB PE=1 SV=2	APOB	TEVIPPLIENR	TEVIPPLIENR	2	640.8641
sp|P04114|APOB_HUMAN	Apolipoprotein B-100 OS=Homo sapiens GN=APOB PE=1 SV=2	APOB	FVTQAEGAK	FVTQAEGAK	2	475.7507
sp|P04114|APOB_HUMAN	Apolipoprotein B-100 OS=Homo sapiens GN=APOB PE=1 SV=2	APOB	SEILAHWSPAK	SEILAHWSPAK	2	619.83
sp|P04114|APOB_HUMAN	Apolipoprotein B-100 OS=Homo sapiens GN=APOB PE=1 SV=2	APOB	VAWHYDEEK	VAWHYDEEK	2	588.7696
sp|P04114|APOB_HUMAN	Apolipoprotein B-100 OS=Homo sapiens GN=APOB PE=1 SV=2	APOB	IEIPLPFGGK	IEIPLPFGGK	2	535.8159
sp|P04114|APOB_HUMAN	Apolipoprotein B-100 OS=Homo sapiens GN=APOB PE=1 SV=2	APOB	TPALHFK	TPALHFK	2	407.2345
sp|P04114|APOB_HUMAN	Apolipoprotein B-100 OS=Homo sapiens GN=APOB PE=1 SV=2	APOB	EFQVPTFTIPK	EFQVPTFTIPK	2	653.8557
sp|P04114|APOB_HUMAN	Apolipoprotein B-100 OS=Homo sapiens GN=APOB PE=1 SV=2	APOB	FLDSNIK	FLDSNIK	2	418.7293
sp|P04114|APOB_HUMAN	Apolipoprotein B-100 OS=Homo sapiens GN=APOB PE=1 SV=2	APOB	LGNNPVSK	LGNNPVSK	2	414.7323
sp|P04114|APOB_HUMAN	Apolipoprotein B-100 OS=Homo sapiens GN=APOB PE=1 SV=2	APOB	VSSFYAK	VSSFYAK	2	401.2107
sp|P04114|APOB_HUMAN	Apolipoprotein B-100 OS=Homo sapiens GN=APOB PE=1 SV=2	APOB	LNGESNLR	LNGESNLR	2	451.7381
sp|P04114|APOB_HUMAN	Apolipoprotein B-100 OS=Homo sapiens GN=APOB PE=1 SV=2	APOB	FN[+1]SSYLQGTNQITGR	FNSSYLQGTNQITGR	2	843.9077
sp|P04114|APOB_HUMAN	Apolipoprotein B-100 OS=Homo sapiens GN=APOB PE=1 SV=2	APOB	FN[+1]SSYLQGTN[+1]QITGR	FNSSYLQGTNQITGR	2	844.3997
sp|P04114|APOB_HUMAN	Apolipoprotein B-100 OS=Homo sapiens GN=APOB PE=1 SV=2	APOB	YENYELTLK	YENYELTLK	2	586.7953
sp|P04114|APOB_HUMAN	Apolipoprotein B-100 OS=Homo sapiens GN=APOB PE=1 SV=2	APOB	SEYQADYESLR	SEYQADYESLR	2	680.8044
sp|P04114|APOB_HUMAN	Apolipoprotein B-100 OS=Homo sapiens GN=APOB PE=1 SV=2	APOB	IGQDGISTSATTNLK	IGQDGISTSATTNLK	2	753.3939
sp|P04114|APOB_HUMAN	Apolipoprotein B-100 OS=Homo sapiens GN=APOB PE=1 SV=2	APOB	LDNIYSSDK	LDNIYSSDK	2	527.7562
sp|P04114|APOB_HUMAN	Apolipoprotein B-100 OS=Homo sapiens GN=APOB PE=1 SV=2	APOB	QTVNLQLQPYSLVTTLNSDLK	QTVNLQLQPYSLVTTLNSDLK	2	1188.144
sp|P04114|APOB_HUMAN	Apolipoprotein B-100 OS=Homo sapiens GN=APOB PE=1 SV=2	APOB	YNALDLTNNGK	YNALDLTNNGK	2	611.8068
sp|P04114|APOB_HUMAN	Apolipoprotein B-100 OS=Homo sapiens GN=APOB PE=1 SV=2	APOB	GAYQNNEIK	GAYQNNEIK	2	518.7565
sp|P04114|APOB_HUMAN	Apolipoprotein B-100 OS=Homo sapiens GN=APOB PE=1 SV=2	APOB	VQGVEFSHR	VQGVEFSHR	2	529.7725
sp|P04114|APOB_HUMAN	Apolipoprotein B-100 OS=Homo sapiens GN=APOB PE=1 SV=2	APOB	AEPLAFTFSHDYK	AEPLAFTFSHDYK	2	763.3697
sp|P04114|APOB_HUMAN	Apolipoprotein B-100 OS=Homo sapiens GN=APOB PE=1 SV=2	APOB	SISAALEHK	SISAALEHK	2	478.264
sp|P04114|APOB_HUMAN	Apolipoprotein B-100 OS=Homo sapiens GN=APOB PE=1 SV=2	APOB	VSALLTPAEQTGTWK	VSALLTPAEQTGTWK	2	801.4303
sp|P04114|APOB_HUMAN	Apolipoprotein B-100 OS=Homo sapiens GN=APOB PE=1 SV=2	APOB	TQFNNNEYSQDLDAYNTK	TQFNNNEYSQDLDAYNTK	2	1082.975
sp|P04114|APOB_HUMAN	Apolipoprotein B-100 OS=Homo sapiens GN=APOB PE=1 SV=2	APOB	TQFN[+1]NNEYSQDLDAYNTK	TQFNNNEYSQDLDAYNTK	2	1083.467
sp|P04114|APOB_HUMAN	Apolipoprotein B-100 OS=Homo sapiens GN=APOB PE=1 SV=2	APOB	IGVELTGR	IGVELTGR	2	422.748
sp|P04114|APOB_HUMAN	Apolipoprotein B-100 OS=Homo sapiens GN=APOB PE=1 SV=2	APOB	TLADLTLLDSPIK	TLADLTLLDSPIK	2	700.4058
sp|P04114|APOB_HUMAN	Apolipoprotein B-100 OS=Homo sapiens GN=APOB PE=1 SV=2	APOB	DAVEKPQEFTIVAFVK	DAVEKPQEFTIVAFVK	2	910.9933
sp|P04114|APOB_HUMAN	Apolipoprotein B-100 OS=Homo sapiens GN=APOB PE=1 SV=2	APOB	ITENDIQIALDDAK	ITENDIQIALDDAK	2	779.9016
sp|P04114|APOB_HUMAN	Apolipoprotein B-100 OS=Homo sapiens GN=APOB PE=1 SV=2	APOB	DSYDLHDLK	DSYDLHDLK	2	553.2617
sp|P04114|APOB_HUMAN	Apolipoprotein B-100 OS=Homo sapiens GN=APOB PE=1 SV=2	APOB	TIHDLHLFIENIDFNK	TIHDLHLFIENIDFNK	2	985.0125
sp|P04114|APOB_HUMAN	Apolipoprotein B-100 OS=Homo sapiens GN=APOB PE=1 SV=2	APOB	SGSSTASWIQNVDTK	SGSSTASWIQNVDTK	2	790.8812
sp|P04114|APOB_HUMAN	Apolipoprotein B-100 OS=Homo sapiens GN=APOB PE=1 SV=2	APOB	QHIEAIDVR	QHIEAIDVR	2	540.7935
sp|P04114|APOB_HUMAN	Apolipoprotein B-100 OS=Homo sapiens GN=APOB PE=1 SV=2	APOB	LSNVLQQVK	LSNVLQQVK	2	514.8086
sp|P04114|APOB_HUMAN	Apolipoprotein B-100 OS=Homo sapiens GN=APOB PE=1 SV=2	APOB	SFDYHQFVDETNDK	SFDYHQFVDETNDK	2	872.8761
sp|P04114|APOB_HUMAN	Apolipoprotein B-100 OS=Homo sapiens GN=APOB PE=1 SV=2	APOB	LFLEETK	LFLEETK	2	440.2447
sp|P04114|APOB_HUMAN	Apolipoprotein B-100 OS=Homo sapiens GN=APOB PE=1 SV=2	APOB	ETLEDTR	ETLEDTR	2	432.2089
sp|P04114|APOB_HUMAN	Apolipoprotein B-100 OS=Homo sapiens GN=APOB PE=1 SV=2	APOB	NLTDFAEQYSIQDWAK	NLTDFAEQYSIQDWAK	2	964.9549
sp|P04114|APOB_HUMAN	Apolipoprotein B-100 OS=Homo sapiens GN=APOB PE=1 SV=2	APOB	ALVEQGFTVPEIK	ALVEQGFTVPEIK	2	715.8981
sp|P04114|APOB_HUMAN	Apolipoprotein B-100 OS=Homo sapiens GN=APOB PE=1 SV=2	APOB	ATFQTPDFIVPLTDLR	ATFQTPDFIVPLTDLR	2	917.4909
sp|P04114|APOB_HUMAN	Apolipoprotein B-100 OS=Homo sapiens GN=APOB PE=1 SV=2	APOB	IPSVQINFK	IPSVQINFK	2	523.3057
sp|P04114|APOB_HUMAN	Apolipoprotein B-100 OS=Homo sapiens GN=APOB PE=1 SV=2	APOB	VEDIPLAR	VEDIPLAR	2	456.7611
sp|P04114|APOB_HUMAN	Apolipoprotein B-100 OS=Homo sapiens GN=APOB PE=1 SV=2	APOB	ITLPDFR	ITLPDFR	2	431.2451
sp|P04114|APOB_HUMAN	Apolipoprotein B-100 OS=Homo sapiens GN=APOB PE=1 SV=2	APOB	SNTVASLHTEK	SNTVASLHTEK	2	593.8068
sp|P04114|APOB_HUMAN	Apolipoprotein B-100 OS=Homo sapiens GN=APOB PE=1 SV=2	APOB	NTLELSN[+1]GVIVK	NTLELSNGVIVK	2	644.3614
sp|P04114|APOB_HUMAN	Apolipoprotein B-100 OS=Homo sapiens GN=APOB PE=1 SV=2	APOB	INNQLTLDSNTK	INNQLTLDSNTK	2	680.857
sp|P04114|APOB_HUMAN	Apolipoprotein B-100 OS=Homo sapiens GN=APOB PE=1 SV=2	APOB	AGHIAWTSSGK	AGHIAWTSSGK	2	557.7856
sp|P04114|APOB_HUMAN	Apolipoprotein B-100 OS=Homo sapiens GN=APOB PE=1 SV=2	APOB	LPYTIITTPPLK	LPYTIITTPPLK	2	678.9105
sp|P04114|APOB_HUMAN	Apolipoprotein B-100 OS=Homo sapiens GN=APOB PE=1 SV=2	APOB	DFSLWEK	DFSLWEK	2	462.7267
sp|P04114|APOB_HUMAN	Apolipoprotein B-100 OS=Homo sapiens GN=APOB PE=1 SV=2	APOB	QSFDLSVK	QSFDLSVK	2	462.2453
sp|P04114|APOB_HUMAN	Apolipoprotein B-100 OS=Homo sapiens GN=APOB PE=1 SV=2	APOB	NNALDFVTK	NNALDFVTK	2	511.2693
sp|P04114|APOB_HUMAN	Apolipoprotein B-100 OS=Homo sapiens GN=APOB PE=1 SV=2	APOB	LSLPDFK	LSLPDFK	2	410.2342
sp|P04114|APOB_HUMAN	Apolipoprotein B-100 OS=Homo sapiens GN=APOB PE=1 SV=2	APOB	AQIPILR	AQIPILR	2	405.7634
sp|P04114|APOB_HUMAN	Apolipoprotein B-100 OS=Homo sapiens GN=APOB PE=1 SV=2	APOB	EYSGTIASEANTYLNSK	EYSGTIASEANTYLNSK	3	616.6268
sp|P04114|APOB_HUMAN	Apolipoprotein B-100 OS=Homo sapiens GN=APOB PE=1 SV=2	APOB	IDDIWNLEVK	IDDIWNLEVK	2	622.8297
sp|P04114|APOB_HUMAN	Apolipoprotein B-100 OS=Homo sapiens GN=APOB PE=1 SV=2	APOB	ENFAGEATLQR	ENFAGEATLQR	2	618.3044
sp|P04114|APOB_HUMAN	Apolipoprotein B-100 OS=Homo sapiens GN=APOB PE=1 SV=2	APOB	NHLQLEGLFFTNGEHTSK	NHLQLEGLFFTNGEHTSK	3	691.3447
sp|P04114|APOB_HUMAN	Apolipoprotein B-100 OS=Homo sapiens GN=APOB PE=1 SV=2	APOB	NIILPVYDK	NIILPVYDK	2	537.8133

sp|P04114|APOB_HUMAN	Apolipoprotein B-100 OS=Homo sapiens GN=APOB PE=1 SV=2	APOB	SLWDFLK	SLWDFLK	2	454.7475
sp|P04114|APOB_HUMAN	Apolipoprotein B-100 OS=Homo sapiens GN=APOB PE=1 SV=2	APOB	LDVTTSIGR	LDVTTSIGR	2	481.2693
sp|P04114|APOB_HUMAN	Apolipoprotein B-100 OS=Homo sapiens GN=APOB PE=1 SV=2	APOB	VSTAFVYTK	VSTAFVYTK	2	508.2766
sp|P04114|APOB_HUMAN	Apolipoprotein B-100 OS=Homo sapiens GN=APOB PE=1 SV=2	APOB	NPNGYSFSIPVK	NPNGYSFSIPVK	2	661.8406
sp|P04114|APOB_HUMAN	Apolipoprotein B-100 OS=Homo sapiens GN=APOB PE=1 SV=2	APOB	TSSFALNLPTLPEVK	TSSFALNLPTLPEVK	2	808.9483
sp|P04114|APOB_HUMAN	Apolipoprotein B-100 OS=Homo sapiens GN=APOB PE=1 SV=2	APOB	FPEVDVLTK	FPEVDVLTK	2	524.2897
sp|P04114|APOB_HUMAN	Apolipoprotein B-100 OS=Homo sapiens GN=APOB PE=1 SV=2	APOB	FSVPAGIVIPSFQALTAR	FSVPAGIVIPSFQALTAR	2	937.5304
sp|P04114|APOB_HUMAN	Apolipoprotein B-100 OS=Homo sapiens GN=APOB PE=1 SV=2	APOB	IEDGTLASK	IEDGTLASK	2	467.248
sp|P04114|APOB_HUMAN	Apolipoprotein B-100 OS=Homo sapiens GN=APOB PE=1 SV=2	APOB	YEGLQEWEGK	YEGLQEWEGK	2	619.788
sp|P04114|APOB_HUMAN	Apolipoprotein B-100 OS=Homo sapiens GN=APOB PE=1 SV=2	APOB	ESDEETQIK	ESDEETQIK	2	539.7486
sp|P04114|APOB_HUMAN	Apolipoprotein B-100 OS=Homo sapiens GN=APOB PE=1 SV=2	APOB	ATGVLYDYVNK	ATGVLYDYVNK	2	621.8219
sp|P04114|APOB_HUMAN	Apolipoprotein B-100 OS=Homo sapiens GN=APOB PE=1 SV=2	APOB	QIDDIDVR	QIDDIDVR	2	487.2511
sp|P04114|APOB_HUMAN	Apolipoprotein B-100 OS=Homo sapiens GN=APOB PE=1 SV=2	APOB	AASGTTGTYQEWK	AASGTTGTYQEWK	2	700.3281
sp|P04114|APOB_HUMAN	Apolipoprotein B-100 OS=Homo sapiens GN=APOB PE=1 SV=2	APOB	AQNLYQELLTQEGQASFQGLK	AQNLYQELLTQEGQASFQGLK	2	1183.603
sp|P04114|APOB_HUMAN	Apolipoprotein B-100 OS=Homo sapiens GN=APOB PE=1 SV=2	APOB	FQFPGKPGIYTR	FQFPGKPGIYTR	2	705.8801
sp|P04114|APOB_HUMAN	Apolipoprotein B-100 OS=Homo sapiens GN=APOB PE=1 SV=2	APOB	EVGTVLSQVYSK	EVGTVLSQVYSK	2	655.3535
sp|P04114|APOB_HUMAN	Apolipoprotein B-100 OS=Homo sapiens GN=APOB PE=1 SV=2	APOB	IISDYHQQFR	IISDYHQQFR	2	653.8306
sp|P04114|APOB_HUMAN	Apolipoprotein B-100 OS=Homo sapiens GN=APOB PE=1 SV=2	APOB	LQDFSDQLSDYYEK	LQDFSDQLSDYYEK	2	875.894
sp|P04180|LCAT_HUMAN	Phosphatidylcholine-sterol acyltransferase OS=Homo sapiens GN=LCAT PE=1 SV=1	LCAT	STELC[+57]GLWQGR	STELCGLWQGR	2	653.8141
sp|P04196|HRG_HUMAN	Histidine-rich glycoprotein OS=Homo sapiens GN=HRG PE=1 SV=1	HRG	VIDFN[+1]C[+57]TTSSVSSALANTK	VIDFNCTTSSVSSALANTK	2	1008.483
sp|P04196|HRG_HUMAN	Histidine-rich glycoprotein OS=Homo sapiens GN=HRG PE=1 SV=1	HRG	EENDDFASFR	EENDDFASFR	2	615.2571
sp|P04196|HRG_HUMAN	Histidine-rich glycoprotein OS=Homo sapiens GN=HRG PE=1 SV=1	HRG	QIGSVYR	QIGSVYR	2	411.7271
sp|P04196|HRG_HUMAN	Histidine-rich glycoprotein OS=Homo sapiens GN=HRG PE=1 SV=1	HRG	GEVLPLPEANFPSFPLPHHK	GEVLPLPEANFPSFPLPHHK	2	1113.589
sp|P04196|HRG_HUMAN	Histidine-rich glycoprotein OS=Homo sapiens GN=HRG PE=1 SV=1	HRG	HPLKPDNQPFPQSVSESC[+57]PGK	HPLKPDNQPFPQSVSESCPGK	3	783.7146
sp|P04217|A1BG_HUMAN	Alpha-1B-glycoprotein OS=Homo sapiens GN=A1BG PE=1 SV=4	A1BG	NGVAQEPVHLDSPAIK	NGVAQEPVHLDSPAIK	2	837.9441
sp|P04217|A1BG_HUMAN	Alpha-1B-glycoprotein OS=Homo sapiens GN=A1BG PE=1 SV=4	A1BG	LLELTGPK	LLELTGPK	2	435.7684
sp|P04217|A1BG_HUMAN	Alpha-1B-glycoprotein OS=Homo sapiens GN=A1BG PE=1 SV=4	A1BG	GVTFLLR	GVTFLLR	2	403.2502
sp|P04217|A1BG_HUMAN	Alpha-1B-glycoprotein OS=Homo sapiens GN=A1BG PE=1 SV=4	A1BG	C[+57]EGPIPDVTFELLR	CEGPIPDVTFELLR	2	823.4163
sp|P04275|VWF_HUMAN	von Willebrand factor OS=Homo sapiens GN=VWF PE=1 SV=4	VWF	IGEADFN[+1]R	IGEADFNR	2	461.7169
sp|P04275|VWF_HUMAN	von Willebrand factor OS=Homo sapiens GN=VWF PE=1 SV=4	VWF	YDAAQLR	YDAAQLR	2	418.7167
sp|P04275|VWF_HUMAN	von Willebrand factor OS=Homo sapiens GN=VWF PE=1 SV=4	VWF	ILAGPAGDSNVVK	ILAGPAGDSNVVK	2	620.8484
sp|P04275|VWF_HUMAN	von Willebrand factor OS=Homo sapiens GN=VWF PE=1 SV=4	VWF	GLRPSC[+57]PNSQSPVK	GLRPSCPNSQSPVK	3	509.5963
sp|P04278|SHBG_HUMAN	Sex hormone-binding globulin OS=Homo sapiens GN=SHBG PE=1 SV=2	SHBG	TSSSFEVR	TSSSFEVR	2	456.7247
sp|P04406|G3P_HUMAN	Glyceraldehyde-3-phosphate dehydrogenase OS=Homo sapiens GN=GAPDH PE=1 SV=3	GAPDH	GALQNIIPASTGAAK	GALQNIIPASTGAAK	2	706.3988
sp|P05062|ALDOB_HUMAN	Fructose-bisphosphate aldolase B OS=Homo sapiens GN=ALDOB PE=1 SV=2	ALDOB	LDQGGAPLAGTNK	LDQGGAPLAGTNK	2	621.3279
sp|P05090|APOD_HUMAN	Apolipoprotein D OS=Homo sapiens GN=APOD PE=1 SV=1	APOD	ADGTVNQIEGEATPVN[+1]LTEPAK	ADGTVNQIEGEATPVNLTEPAK	2	1128.056
sp|P05154|IPSP_HUMAN	Plasma serine protease inhibitor OS=Homo sapiens GN=SERPINA5 PE=1 SV=3	SERPINA5	GFQQLLQELNQPR	GFQQLLQELNQPR	2	785.9204
sp|P05154|IPSP_HUMAN	Plasma serine protease inhibitor OS=Homo sapiens GN=SERPINA5 PE=1 SV=3	SERPINA5	TLYLADTFPTNFR	TLYLADTFPTNFR	2	779.8986
sp|P05154|IPSP_HUMAN	Plasma serine protease inhibitor OS=Homo sapiens GN=SERPINA5 PE=1 SV=3	SERPINA5	VVGVPYQGN[+1]ATALFILPSEGK	VVGVPYQGNATALFILPSEGK	2	1081.081
sp|P05155|IC1_HUMAN	Plasma protease C1 inhibitor OS=Homo sapiens GN=SERPING1 PE=1 SV=2	SERPING1	DFTC[+57]VHQALK	DFTCVHQALK	2	609.8004
sp|P05155|IC1_HUMAN	Plasma protease C1 inhibitor OS=Homo sapiens GN=SERPING1 PE=1 SV=2	SERPING1	TLYSSSPR	TLYSSSPR	2	455.7351
sp|P05155|IC1_HUMAN	Plasma protease C1 inhibitor OS=Homo sapiens GN=SERPING1 PE=1 SV=2	SERPING1	FQPTLLTLPR	FQPTLLTLPR	2	593.3531
sp|P05156|CFAI_HUMAN	Complement factor I OS=Homo sapiens GN=CFI PE=1 SV=2	CFI	VTYTSQEDLVEK	VTYTSQEDLVEK	2	706.3512
sp|P05156|CFAI_HUMAN	Complement factor I OS=Homo sapiens GN=CFI PE=1 SV=2	CFI	C[+57]IEGTC[+57]VC[+57]K	CIEGTCVCK	2	563.7381
sp|P05156|CFAI_HUMAN	Complement factor I OS=Homo sapiens GN=CFI PE=1 SV=2	CFI	SFPTYC[+57]QQK	SFPTYCQQK	2	579.766
sp|P05156|CFAI_HUMAN	Complement factor I OS=Homo sapiens GN=CFI PE=1 SV=2	CFI	SLEC[+57]LHPGTK	SLECLHPGTK	2	571.2871
sp|P05156|CFAI_HUMAN	Complement factor I OS=Homo sapiens GN=CFI PE=1 SV=2	CFI	FLNN[+1]GTC[+57]TAEGK	FLNNGTCTAEGK	2	656.7955
sp|P05156|CFAI_HUMAN	Complement factor I OS=Homo sapiens GN=CFI PE=1 SV=2	CFI	AC[+57]DGINDC[+57]GDQSDELC[+57]C[+57]K	ACDGINDCGDQSDELCCK	2	1058.887
sp|P05156|CFAI_HUMAN	Complement factor I OS=Homo sapiens GN=CFI PE=1 SV=2	CFI	AQLGDLPWQVAIK	AQLGDLPWQVAIK	2	719.9063
sp|P05156|CFAI_HUMAN	Complement factor I OS=Homo sapiens GN=CFI PE=1 SV=2	CFI	IVIEYVDR	IVIEYVDR	2	503.782
sp|P05156|CFAI_HUMAN	Complement factor I OS=Homo sapiens GN=CFI PE=1 SV=2	CFI	VFSLQWGEVK	VFSLQWGEVK	2	596.8217
sp|P05452|TETN_HUMAN	Tetranectin OS=Homo sapiens GN=CLEC3B PE=1 SV=3	CLEC3B	EQQALQTVC[+57]LK	EQQALQTVCLK	2	659.3452
sp|P05452|TETN_HUMAN	Tetranectin OS=Homo sapiens GN=CLEC3B PE=1 SV=3	CLEC3B	TFHEASEDC[+57]ISR	TFHEASEDCISR	2	726.3146
sp|P05452|TETN_HUMAN	Tetranectin OS=Homo sapiens GN=CLEC3B PE=1 SV=3	CLEC3B	NWETEITAQPDGGK	NWETEITAQPDGGK	2	773.3626
sp|P05543|THBG_HUMAN	Thyroxine-binding globulin OS=Homo sapiens GN=SERPINA7 PE=1 SV=2	SERPINA7	AQWANPFDPSK	AQWANPFDPSK	2	630.804
sp|P05543|THBG_HUMAN	Thyroxine-binding globulin OS=Homo sapiens GN=SERPINA7 PE=1 SV=2	SERPINA7	TEDSSSFLIDK	TEDSSSFLIDK	2	621.2984
sp|P05543|THBG_HUMAN	Thyroxine-binding globulin OS=Homo sapiens GN=SERPINA7 PE=1 SV=2	SERPINA7	NALALFVLPK	NALALFVLPK	2	543.3395
sp|P05543|THBG_HUMAN	Thyroxine-binding globulin OS=Homo sapiens GN=SERPINA7 PE=1 SV=2	SERPINA7	GWVDLFVPK	GWVDLFVPK	2	530.7949
sp|P05543|THBG_HUMAN	Thyroxine-binding globulin OS=Homo sapiens GN=SERPINA7 PE=1 SV=2	SERPINA7	AVLHIGEK	AVLHIGEK	2	433.7584
sp|P05543|THBG_HUMAN	Thyroxine-binding globulin OS=Homo sapiens GN=SERPINA7 PE=1 SV=2	SERPINA7	GTEAAAVPEVELSDQPENTFLHPIIQIDR	GTEAAAVPEVELSDQPENTFLHPIIQIDR	3	1063.874
sp|P05546|HEP2_HUMAN	Heparin cofactor 2 OS=Homo sapiens GN=SERPIND1 PE=1 SV=3	SERPIND1	GGETAQSADPQWEQLNNK	GGETAQSADPQWEQLNNK	2	986.9534
sp|P05546|HEP2_HUMAN	Heparin cofactor 2 OS=Homo sapiens GN=SERPIND1 PE=1 SV=3	SERPIND1	FAFNLYR	FAFNLYR	2	465.7452
sp|P05546|HEP2_HUMAN	Heparin cofactor 2 OS=Homo sapiens GN=SERPIND1 PE=1 SV=3	SERPIND1	YEITTIHNLFR	YEITTIHNLFR	2	703.875
sp|P05546|HEP2_HUMAN	Heparin cofactor 2 OS=Homo sapiens GN=SERPIND1 PE=1 SV=3	SERPIND1	NFGYTLR	NFGYTLR	2	435.7271
sp|P05546|HEP2_HUMAN	Heparin cofactor 2 OS=Homo sapiens GN=SERPIND1 PE=1 SV=3	SERPIND1	QFPILLDFK	QFPILLDFK	2	560.8237
sp|P05546|HEP2_HUMAN	Heparin cofactor 2 OS=Homo sapiens GN=SERPIND1 PE=1 SV=3	SERPIND1	N[+1]YNLVESLK	NYNLVESLK	2	540.7822
sp|P06276|CHLE_HUMAN	Cholinesterase OS=Homo sapiens GN=BCHE PE=1 SV=1	BCHE	DEGTAFLVYGAPGFSK	DEGTAFLVYGAPGFSK	2	829.9067
sp|P06396|GELS_HUMAN	Gelsolin OS=Homo sapiens GN=GSN PE=1 SV=1	GSN	HVVPNEVVVQR	HVVPNEVVVQR	2	638.362
sp|P06396|GELS_HUMAN	Gelsolin OS=Homo sapiens GN=GSN PE=1 SV=1	GSN	SEDC[+57]FILDHGK	SEDCFILDHGK	2	660.7981
sp|P06396|GELS_HUMAN	Gelsolin OS=Homo sapiens GN=GSN PE=1 SV=1	GSN	TASDFITK	TASDFITK	2	441.732
sp|P06396|GELS_HUMAN	Gelsolin OS=Homo sapiens GN=GSN PE=1 SV=1	GSN	QTQVSVLPEGGETPLFK	QTQVSVLPEGGETPLFK	2	915.4858
sp|P06396|GELS_HUMAN	Gelsolin OS=Homo sapiens GN=GSN PE=1 SV=1	GSN	DPDQTDGLGLSYLSSHIANVER	DPDQTDGLGLSYLSSHIANVER	2	1194.077
sp|P06396|GELS_HUMAN	Gelsolin OS=Homo sapiens GN=GSN PE=1 SV=1	GSN	AGALNSNDAFVLK	AGALNSNDAFVLK	2	660.3513
sp|P06681|CO2_HUMAN	Complement C2 OS=Homo sapiens GN=C2 PE=1 SV=2	C2	C[+57]SSNLVLTGSSER	CSSNLVLTGSSER	2	705.3381
sp|P06681|CO2_HUMAN	Complement C2 OS=Homo sapiens GN=C2 PE=1 SV=2	C2	EC[+57]QGN[+1]GVWSGTEPIC[+57]R	ECQGNGVWSGTEPICR	3	617.5981
sp|P06681|CO2_HUMAN	Complement C2 OS=Homo sapiens GN=C2 PE=1 SV=2	C2	EC[+57]QGNGVWSGTEPIC[+57]R	ECQGNGVWSGTEPICR	2	925.4014
sp|P06681|CO2_HUMAN	Complement C2 OS=Homo sapiens GN=C2 PE=1 SV=2	C2	HAIILTDGK	HAIILTDGK	2	540.8242
sp|P06681|CO2_HUMAN	Complement C2 OS=Homo sapiens GN=C2 PE=1 SV=2	C2	EILNINQK	EILNINQK	2	486.2796
sp|P06681|CO2_HUMAN	Complement C2 OS=Homo sapiens GN=C2 PE=1 SV=2	C2	ELNELGSK	ELNELGSK	2	445.2349
sp|P06681|CO2_HUMAN	Complement C2 OS=Homo sapiens GN=C2 PE=1 SV=2	C2	ELN[+1]ELGSK	ELNELGSK	2	445.7269
sp|P06681|CO2_HUMAN	Complement C2 OS=Homo sapiens GN=C2 PE=1 SV=2	C2	TPWHVTIKPK	TPWHVTIKPK	2	603.8533
sp|P06681|CO2_HUMAN	Complement C2 OS=Homo sapiens GN=C2 PE=1 SV=2	C2	DGNDHSLWR	DGNDHSLWR	2	550.2494
sp|P06681|CO2_HUMAN	Complement C2 OS=Homo sapiens GN=C2 PE=1 SV=2	C2	EVVTDQFLC[+57]SGTQEDESPC[+57]K	EVVTDQFLCSGTQEDESPCK	2	1165.002
sp|P06681|CO2_HUMAN	Complement C2 OS=Homo sapiens GN=C2 PE=1 SV=2	C2	GESGGAVFLER	GESGGAVFLER	2	561.2829
sp|P06727|APOA4_HUMAN	Apolipoprotein A-IV OS=Homo sapiens GN=APOA4 PE=1 SV=3	APOA4	SELTQQLNALFQDK	SELTQQLNALFQDK	2	817.9229
sp|P06727|APOA4_HUMAN	Apolipoprotein A-IV OS=Homo sapiens GN=APOA4 PE=1 SV=3	APOA4	LGEVNTYAGDLQK	LGEVNTYAGDLQK	2	704.3594
sp|P06727|APOA4_HUMAN	Apolipoprotein A-IV OS=Homo sapiens GN=APOA4 PE=1 SV=3	APOA4	LGEVN[+1]TYAGDLQK	LGEVNTYAGDLQK	2	704.8514

sp|P06727|APOA4_HUMAN	Apolipoprotein A-IV OS=Homo sapiens GN=APOA4 PE=1 SV=3	APOA4	LVPFATELHER	LVPFATELHER	3	437.9067
sp|P06727|APOA4_HUMAN	Apolipoprotein A-IV OS=Homo sapiens GN=APOA4 PE=1 SV=3	APOA4	LLPHANEVSQK	LLPHANEVSQK	2	618.3408
sp|P06727|APOA4_HUMAN	Apolipoprotein A-IV OS=Homo sapiens GN=APOA4 PE=1 SV=3	APOA4	LLPHAN[+1]EVSQK	LLPHANEVSQK	2	618.8328
sp|P06727|APOA4_HUMAN	Apolipoprotein A-IV OS=Homo sapiens GN=APOA4 PE=1 SV=3	APOA4	LEPYADQLR	LEPYADQLR	2	552.7878
sp|P06727|APOA4_HUMAN	Apolipoprotein A-IV OS=Homo sapiens GN=APOA4 PE=1 SV=3	APOA4	ENADSLQASLRPHADELK	ENADSLQASLRPHADELK	2	997.5005
sp|P06727|APOA4_HUMAN	Apolipoprotein A-IV OS=Homo sapiens GN=APOA4 PE=1 SV=3	APOA4	IDQNVEELK	IDQNVEELK	2	544.2851
sp|P06727|APOA4_HUMAN	Apolipoprotein A-IV OS=Homo sapiens GN=APOA4 PE=1 SV=3	APOA4	IDQTVEELR	IDQTVEELR	2	551.7906
sp|P06727|APOA4_HUMAN	Apolipoprotein A-IV OS=Homo sapiens GN=APOA4 PE=1 SV=3	APOA4	SLAPYAQDTQEK	SLAPYAQDTQEK	2	675.8304
sp|P06727|APOA4_HUMAN	Apolipoprotein A-IV OS=Homo sapiens GN=APOA4 PE=1 SV=3	APOA4	ISASAEELR	ISASAEELR	2	488.2589
sp|P06727|APOA4_HUMAN	Apolipoprotein A-IV OS=Homo sapiens GN=APOA4 PE=1 SV=3	APOA4	LAPLAEDVR	LAPLAEDVR	2	492.2796
sp|P06727|APOA4_HUMAN	Apolipoprotein A-IV OS=Homo sapiens GN=APOA4 PE=1 SV=3	APOA4	GNTEGLQK	GNTEGLQK	2	423.7194
sp|P06727|APOA4_HUMAN	Apolipoprotein A-IV OS=Homo sapiens GN=APOA4 PE=1 SV=3	APOA4	VEPYGENFNK	VEPYGENFNK	2	598.7828
sp|P06727|APOA4_HUMAN	Apolipoprotein A-IV OS=Homo sapiens GN=APOA4 PE=1 SV=3	APOA4	VNSFFSTFK	VNSFFSTFK	2	538.7742
sp|P06753|TPM3_HUMAN	Tropomyosin alpha-3 chain OS=Homo sapiens GN=TPM3 PE=1 SV=2	TPM3	IQLVEEELDR	IQLVEEELDR	2	622.3301
sp|P07225|PROS_HUMAN	Vitamin K-dependent protein S OS=Homo sapiens GN=PROS1 PE=1 SV=1	PROS1	ASFTC[+57]TC[+57]KPGWQGEK	ASFTCTCKPGWQGEK	2	878.8927
sp|P07225|PROS_HUMAN	Vitamin K-dependent protein S OS=Homo sapiens GN=PROS1 PE=1 SV=1	PROS1	DVDEC[+57]SLKPSIC[+57]GTAVC[+57]K	DVDECSLKPSICGTAVCK	2	1019.966
sp|P07225|PROS_HUMAN	Vitamin K-dependent protein S OS=Homo sapiens GN=PROS1 PE=1 SV=1	PROS1	IETISHEDLQR	IETISHEDLQR	2	670.8439
sp|P07357|CO8A_HUMAN	Complement component C8 alpha chain OS=Homo sapiens GN=C8A PE=1 SV=2	C8A	FGGTIC[+57]SGDIWDQASC[+57]SSSTTC[+57]VR	FGGTICSGDIWDQASCSSSTTCVR	3	884.709
sp|P07357|CO8A_HUMAN	Complement component C8 alpha chain OS=Homo sapiens GN=C8A PE=1 SV=2	C8A	HLVC[+57]NGDQDC[+57]LDGSDEDDC[+57]EDV R	HLVCNGDQDCLDGSDEDDCEDVR	3	908.3465
sp|P07357|CO8A_HUMAN	Complement component C8 alpha chain OS=Homo sapiens GN=C8A PE=1 SV=2	C8A	AIDEDC[+57]SQYEPIPGSQK	AIDEDCSQYEPIPGSQK	2	968.9333
sp|P07357|CO8A_HUMAN	Complement component C8 alpha chain OS=Homo sapiens GN=C8A PE=1 SV=2	C8A	INVGGGLSGDHC[+57]K	INVGGGLSGDHCK	2	657.317
sp|P07357|CO8A_HUMAN	Complement component C8 alpha chain OS=Homo sapiens GN=C8A PE=1 SV=2	C8A	GGSSGWSGGLAQN[+1]R	GGSSGWSGGLAQNR	2	667.8078
sp|P07357|CO8A_HUMAN	Complement component C8 alpha chain OS=Homo sapiens GN=C8A PE=1 SV=2	C8A	HTSLGPLEAK	HTSLGPLEAK	2	526.7904
sp|P07357|CO8A_HUMAN	Complement component C8 alpha chain OS=Homo sapiens GN=C8A PE=1 SV=2	C8A	LGSLGAAC[+57]EQTQTEGAK	LGSLGAACEQTQTEGAK	2	860.9122
sp|P07358|CO8B_HUMAN	Complement component C8 beta chain OS=Homo sapiens GN=C8B PE=1 SV=3	C8B	YAYLLQPSQFHGEPC[+57]NFSDK	YAYLLQPSQFHGEPCNFSDK	3	801.0354
sp|P07358|CO8B_HUMAN	Complement component C8 beta chain OS=Homo sapiens GN=C8B PE=1 SV=3	C8B	C[+57]EGFVC[+57]AQTGR	CEGFVCAQTGR	2	642.7766
sp|P07358|CO8B_HUMAN	Complement component C8 beta chain OS=Homo sapiens GN=C8B PE=1 SV=3	C8B	LLC[+57]NGDNDC[+57]GDQSDEANC[+57]R	LLCNGDNDCGDQSDEANCR	2	1106.918
sp|P07358|CO8B_HUMAN	Complement component C8 beta chain OS=Homo sapiens GN=C8B PE=1 SV=3	C8B	SGFSFGFK	SGFSFGFK	2	438.7162
sp|P07358|CO8B_HUMAN	Complement component C8 beta chain OS=Homo sapiens GN=C8B PE=1 SV=3	C8B	SVFLHAR	SVFLHAR	2	415.2376
sp|P07358|CO8B_HUMAN	Complement component C8 beta chain OS=Homo sapiens GN=C8B PE=1 SV=3	C8B	SDLEVAHYK	SDLEVAHYK	2	531.2667
sp|P07358|CO8B_HUMAN	Complement component C8 beta chain OS=Homo sapiens GN=C8B PE=1 SV=3	C8B	GDYTLNNVHAC[+57]AK	GDYTLNNVHACAK	2	731.8408
sp|P07358|CO8B_HUMAN	Complement component C8 beta chain OS=Homo sapiens GN=C8B PE=1 SV=3	C8B	QALEEFQK	QALEEFQK	2	496.756
sp|P07358|CO8B_HUMAN	Complement component C8 beta chain OS=Homo sapiens GN=C8B PE=1 SV=3	C8B	C[+57]DC[+57]IC[+57]PVGSQGLAC[+57]EVSYR	CDCICPVGSQGLACEVSYR	2	1115.971
sp|P07359|GP1BA_HUMAN	Platelet glycoprotein Ib alpha chain OS=Homo sapiens GN=GP1BA PE=1 SV=2	GP1BA	LTSLPLGALR	LTSLPLGALR	2	520.8268
sp|P07359|GP1BA_HUMAN	Platelet glycoprotein Ib alpha chain OS=Homo sapiens GN=GP1BA PE=1 SV=2	GP1BA	GLGELQELYLK	GLGELQELYLK	2	631.8532
sp|P07360|CO8G_HUMAN	Complement component C8 gamma chain OS=Homo sapiens GN=C8G PE=1 SV=3	C8G	RPASPISTIQPK	RPASPISTIQPK	2	647.8775
sp|P07360|CO8G_HUMAN	Complement component C8 gamma chain OS=Homo sapiens GN=C8G PE=1 SV=3	C8G	FLQEQGHR	FLQEQGHR	2	507.7594
sp|P07360|CO8G_HUMAN	Complement component C8 gamma chain OS=Homo sapiens GN=C8G PE=1 SV=3	C8G	QLYGDTGVLGR	QLYGDTGVLGR	2	589.8118
sp|P07360|CO8G_HUMAN	Complement component C8 gamma chain OS=Homo sapiens GN=C8G PE=1 SV=3	C8G	SLPVSDSVLSGFEQR	SLPVSDSVLSGFEQR	2	810.915
sp|P07360|CO8G_HUMAN	Complement component C8 gamma chain OS=Homo sapiens GN=C8G PE=1 SV=3	C8G	VQEAHLTEDQIFYFPK	VQEAHLTEDQIFYFPK	2	982.9913
sp|P07737|PROF1_HUMAN	Profilin-1 OS=Homo sapiens GN=PFN1 PE=1 SV=2	PFN1	EGVHGGLINK	EGVHGGLINK	2	512.2827
sp|P07996|TSP1_HUMAN	Thrombospondin-1 OS=Homo sapiens GN=THBS1 PE=1 SV=2	THBS1	GTLLALER	GTLLALER	2	436.7636
sp|P07996|TSP1_HUMAN	Thrombospondin-1 OS=Homo sapiens GN=THBS1 PE=1 SV=2	THBS1	GGVNDNFQGVLQNVR	GGVNDNFQGVLQNVR	2	808.9106
sp|P07996|TSP1_HUMAN	Thrombospondin-1 OS=Homo sapiens GN=THBS1 PE=1 SV=2	THBS1	AQGYSGLSVK	AQGYSGLSVK	2	505.2693
sp|P08185|CBG_HUMAN	Corticosteroid-binding globulin OS=Homo sapiens GN=SERPINA6 PE=1 SV=1	SERPINA6	HLVALSPK	HLVALSPK	2	432.7687
sp|P08185|CBG_HUMAN	Corticosteroid-binding globulin OS=Homo sapiens GN=SERPINA6 PE=1 SV=1	SERPINA6	QINSYVK	QINSYVK	2	426.2347
sp|P08571|CD14_HUMAN	Monocyte differentiation antigen CD14 OS=Homo sapiens GN=CD14 PE=1 SV=2	CD14	VLSIAQAHSPAFSC[+57]EQVR	VLSIAQAHSPAFSCEQVR	3	667.3388
sp|P08571|CD14_HUMAN	Monocyte differentiation antigen CD14 OS=Homo sapiens GN=CD14 PE=1 SV=2	CD14	AFPALTSLDLSDNPGLGER	AFPALTSLDLSDNPGLGER	2	987.0024
sp|P08571|CD14_HUMAN	Monocyte differentiation antigen CD14 OS=Homo sapiens GN=CD14 PE=1 SV=2	CD14	ATVNPSAPR	ATVNPSAPR	2	456.7485
sp|P08603|CFAH_HUMAN	Complement factor H OS=Homo sapiens GN=CFH PE=1 SV=4	CFH	NTEILTGSWSDQTYPEGTQAIYK	NTEILTGSWSDQTYPEGTQAIYK	3	868.0818
sp|P08603|CFAH_HUMAN	Complement factor H OS=Homo sapiens GN=CFH PE=1 SV=4	CFH	GEWVALNPLR	GEWVALNPLR	2	577.8195
sp|P08603|CFAH_HUMAN	Complement factor H OS=Homo sapiens GN=CFH PE=1 SV=4	CFH	EC[+57]DTDGWTNDIPIC[+57]EVVK	ECDTDGWTNDIPICEVVK	2	1075.972
sp|P08603|CFAH_HUMAN	Complement factor H OS=Homo sapiens GN=CFH PE=1 SV=4	CFH	C[+57]LPVTAPENGK	CLPVTAPENGK	2	593.3003
sp|P08603|CFAH_HUMAN	Complement factor H OS=Homo sapiens GN=CFH PE=1 SV=4	CFH	C[+57]LPVTAPEN[+1]GK	CLPVTAPENGK	2	593.7923
sp|P08603|CFAH_HUMAN	Complement factor H OS=Homo sapiens GN=CFH PE=1 SV=4	CFH	C[+57]VEISC[+57]K	CVEISCK	2	448.2042
sp|P08603|CFAH_HUMAN	Complement factor H OS=Homo sapiens GN=CFH PE=1 SV=4	CFH	SPDVINGSPISQK	SPDVINGSPISQK	2	671.3541
sp|P08603|CFAH_HUMAN	Complement factor H OS=Homo sapiens GN=CFH PE=1 SV=4	CFH	GDAVC[+57]TESGWRPLPSC[+57]EEK	GDAVCTESGWRPLPSCEEK	2	1089.483
sp|P08603|CFAH_HUMAN	Complement factor H OS=Homo sapiens GN=CFH PE=1 SV=4	CFH	TGDEITYQC[+57]R	TGDEITYQCR	2	621.7746
sp|P08603|CFAH_HUMAN	Complement factor H OS=Homo sapiens GN=CFH PE=1 SV=4	CFH	C[+57]TSTGWIPAPR	CTSTGWIPAPR	2	623.3059
sp|P08603|CFAH_HUMAN	Complement factor H OS=Homo sapiens GN=CFH PE=1 SV=4	CFH	C[+57]TLKPC[+57]DYPDIK	CTLKPCDYPDIK	2	755.3574
sp|P08603|CFAH_HUMAN	Complement factor H OS=Homo sapiens GN=CFH PE=1 SV=4	CFH	SIDVAC[+57]HPGYALPK	SIDVACHPGYALPK	2	764.3849
sp|P08603|CFAH_HUMAN	Complement factor H OS=Homo sapiens GN=CFH PE=1 SV=4	CFH	NDFTWFK	NDFTWFK	2	479.2269
sp|P08603|CFAH_HUMAN	Complement factor H OS=Homo sapiens GN=CFH PE=1 SV=4	CFH	IDVHLVPDR	IDVHLVPDR	2	532.2984
sp|P08603|CFAH_HUMAN	Complement factor H OS=Homo sapiens GN=CFH PE=1 SV=4	CFH	EEYGHSEVVEYYC[+57]NPR	EEYGHSEVVEYYCNPR	2	1015.931
sp|P08603|CFAH_HUMAN	Complement factor H OS=Homo sapiens GN=CFH PE=1 SV=4	CFH	SSNLIILEEHLK	SSNLIILEEHLK	2	698.3957
sp|P08603|CFAH_HUMAN	Complement factor H OS=Homo sapiens GN=CFH PE=1 SV=4	CFH	EGWIHTVC[+57]IN[+1]GR	EGWIHTVCINGR	2	721.8459
sp|P08603|CFAH_HUMAN	Complement factor H OS=Homo sapiens GN=CFH PE=1 SV=4	CFH	EGWIHTVC[+57]INGR	EGWIHTVCINGR	2	721.3539

sp|P08603|CFAH_HUMAN	Complement factor H OS=Homo sapiens GN=CFH PE=1 SV=4	CFH	WQSIPLC[+57]VEK	WQSIPLCVEK	2	630.3263
sp|P08603|CFAH_HUMAN	Complement factor H OS=Homo sapiens GN=CFH PE=1 SV=4	CFH	IPC[+57]SQPPQIEHGTIN[+1]SSR	IPCSQPPQIEHGTINSSR	3	674.6618
sp|P08603|CFAH_HUMAN	Complement factor H OS=Homo sapiens GN=CFH PE=1 SV=4	CFH	LSYTC[+57]EGGFR	LSYTCEGGFR	2	595.269
sp|P08603|CFAH_HUMAN	Complement factor H OS=Homo sapiens GN=CFH PE=1 SV=4	CFH	WSSPPQC[+57]EGLPC[+57]K	WSSPPQCEGLPCK	2	773.3449
sp|P08603|CFAH_HUMAN	Complement factor H OS=Homo sapiens GN=CFH PE=1 SV=4	CFH	DTSC[+57]VNPPTVQNAYIVSR	DTSCVNPPTVQNAYIVSR	2	1010.991
sp|P08603|CFAH_HUMAN	Complement factor H OS=Homo sapiens GN=CFH PE=1 SV=4	CFH	C[+57]LHPC[+57]VISR	CLHPCVISR	2	571.2839
sp|P08603|CFAH_HUMAN	Complement factor H OS=Homo sapiens GN=CFH PE=1 SV=4	CFH	TGESVEFVC[+57]K	TGESVEFVCK	2	578.2712
sp|P08603|CFAH_HUMAN	Complement factor H OS=Homo sapiens GN=CFH PE=1 SV=4	CFH	TTC[+57]WDGK	TTCWDGK	2	434.1869
sp|P08697|A2AP_HUMAN	Alpha-2-antiplasmin OS=Homo sapiens GN=SERPINF2 PE=1 SV=3	SERPINF2	QLTSGPNQEQVSPLTLLK	QLTSGPNQEQVSPLTLLK	2	977.0362
sp|P08697|A2AP_HUMAN	Alpha-2-antiplasmin OS=Homo sapiens GN=SERPINF2 PE=1 SV=3	SERPINF2	LGNQEPGGQTALK	LGNQEPGGQTALK	2	656.8464
sp|P08697|A2AP_HUMAN	Alpha-2-antiplasmin OS=Homo sapiens GN=SERPINF2 PE=1 SV=3	SERPINF2	LGN[+1]QEPGGQTALK	LGNQEPGGQTALK	2	657.3384
sp|P08697|A2AP_HUMAN	Alpha-2-antiplasmin OS=Homo sapiens GN=SERPINF2 PE=1 SV=3	SERPINF2	SPPGVC[+57]SR	SPPGVCSR	2	430.2082
sp|P08697|A2AP_HUMAN	Alpha-2-antiplasmin OS=Homo sapiens GN=SERPINF2 PE=1 SV=3	SERPINF2	LQQVLHAGSGPC[+57]LPHLLSR	LQQVLHAGSGPCLPHLLSR	3	695.0458
sp|P08697|A2AP_HUMAN	Alpha-2-antiplasmin OS=Homo sapiens GN=SERPINF2 PE=1 SV=3	SERPINF2	EDFLEQSEQLFGAKPVSLTGK	EDFLEQSEQLFGAKPVSLTGK	3	775.0654
sp|P08697|A2AP_HUMAN	Alpha-2-antiplasmin OS=Homo sapiens GN=SERPINF2 PE=1 SV=3	SERPINF2	QEDDLANINQWVK	QEDDLANINQWVK	2	786.8863
sp|P08697|A2AP_HUMAN	Alpha-2-antiplasmin OS=Homo sapiens GN=SERPINF2 PE=1 SV=3	SERPINF2	FDPSLTQR	FDPSLTQR	2	482.2483
sp|P08697|A2AP_HUMAN	Alpha-2-antiplasmin OS=Homo sapiens GN=SERPINF2 PE=1 SV=3	SERPINF2	DFLQSLK	DFLQSLK	2	425.7371
sp|P09871|C1S_HUMAN	Complement C1s subcomponent OS=Homo sapiens GN=C1S PE=1 SV=1	C1S	SDFSNEER	SDFSNEER	2	492.2069
sp|P09871|C1S_HUMAN	Complement C1s subcomponent OS=Homo sapiens GN=C1S PE=1 SV=1	C1S	SDFSN[+1]EER	SDFSNEER	2	492.6989
sp|P09871|C1S_HUMAN	Complement C1s subcomponent OS=Homo sapiens GN=C1S PE=1 SV=1	C1S	QFGPYC[+57]GHGFPGPLNIETK	QFGPYCGHGFPGPLNIETK	3	707.007
sp|P09871|C1S_HUMAN	Complement C1s subcomponent OS=Homo sapiens GN=C1S PE=1 SV=1	C1S	EDTPNSVWEPAK	EDTPNSVWEPAK	2	686.8226
sp|P09871|C1S_HUMAN	Complement C1s subcomponent OS=Homo sapiens GN=C1S PE=1 SV=1	C1S	VGATSFYSTC[+57]QSNGK	VGATSFYSTCQSNGK	2	803.8619
sp|P09871|C1S_HUMAN	Complement C1s subcomponent OS=Homo sapiens GN=C1S PE=1 SV=1	C1S	C[+57]QPVDC[+57]GIPESIENGK	CQPVDCGIPESIENGK	2	901.906
sp|P09871|C1S_HUMAN	Complement C1s subcomponent OS=Homo sapiens GN=C1S PE=1 SV=1	C1S	VEDPESTLFGSVIR	VEDPESTLFGSVIR	3	516.935
sp|P09871|C1S_HUMAN	Complement C1s subcomponent OS=Homo sapiens GN=C1S PE=1 SV=1	C1S	C[+57]VPVC[+57]GVPR	CVPVCGVPR	2	522.2599
sp|P09871|C1S_HUMAN	Complement C1s subcomponent OS=Homo sapiens GN=C1S PE=1 SV=1	C1S	IIGGSDADIK	IIGGSDADIK	2	494.7691
sp|P09871|C1S_HUMAN	Complement C1s subcomponent OS=Homo sapiens GN=C1S PE=1 SV=1	C1S	LLEVPEGR	LLEVPEGR	2	456.7611
sp|P09871|C1S_HUMAN	Complement C1s subcomponent OS=Homo sapiens GN=C1S PE=1 SV=1	C1S	GDSGGAFAVQDPNDK	GDSGGAFAVQDPNDK	2	739.3313
sp|P0C0L4|CO4A_HUMAN	Complement C4-A OS=Homo sapiens GN=C4A PE=1 SV=2	C4A	NNVPC[+57]SPK	NNVPCSPK	2	458.2213
sp|P0C0L4|CO4A_HUMAN	Complement C4-A OS=Homo sapiens GN=C4A PE=1 SV=2	C4A	N[+1]NVPC[+57]SPK	NNVPCSPK	2	458.7133
sp|P0C0L4|CO4A_HUMAN	Complement C4-A OS=Homo sapiens GN=C4A PE=1 SV=2	C4A	VDFTLSSER	VDFTLSSER	2	527.2642
sp|P0C0L5|CO4B_HUMAN	Complement C4-B OS=Homo sapiens GN=C4B PE=1 SV=2	C4B	C[+57]SVFYGAPSK	CSVFYGAPSK	2	558.2631
sp|P0C0L5|CO4B_HUMAN	Complement C4-B OS=Homo sapiens GN=C4B PE=1 SV=2	C4B	FAC[+57]YYPR	FACYYPR	2	488.7209
sp|P0C0L5|CO4B_HUMAN	Complement C4-B OS=Homo sapiens GN=C4B PE=1 SV=2	C4B	VEYGFQVK	VEYGFQVK	2	485.2556
sp|P0C0L5|CO4B_HUMAN	Complement C4-B OS=Homo sapiens GN=C4B PE=1 SV=2	C4B	ITQVLHFTK	ITQVLHFTK	2	543.8189
sp|P0C0L5|CO4B_HUMAN	Complement C4-B OS=Homo sapiens GN=C4B PE=1 SV=2	C4B	AAC[+57]AQLNDFLQEYGTQGC[+57]QV	AACAQLNDFLQEYGTQGCQV	2	1137.002
sp|P0CG47|UBB_HUMAN	Polyubiquitin-B OS=Homo sapiens GN=UBB PE=1 SV=1	UBB	TITLEVEPSDTIENVK	TITLEVEPSDTIENVK	2	894.4673
sp|P0DJI8|SAA1_HUMAN	Serum amyloid A-1 protein OS=Homo sapiens GN=SAA1 PE=1 SV=1	SAA1	EANYIGSDK	EANYIGSDK	2	498.7353
sp|P0DJI8|SAA1_HUMAN	Serum amyloid A-1 protein OS=Homo sapiens GN=SAA1 PE=1 SV=1	SAA1	EAN[+1]YIGSDK	EANYIGSDK	2	499.2273
sp|P0DJI8|SAA1_HUMAN	Serum amyloid A-1 protein OS=Homo sapiens GN=SAA1 PE=1 SV=1	SAA1	FFGHGAEDSLADQAANEWGR	FFGHGAEDSLADQAANEWGR	2	1089.485
sp|P10643|CO7_HUMAN	Complement component C7 OS=Homo sapiens GN=C7 PE=1 SV=2	C7	GC[+57]PTEEGC[+57]GER	GCPTEEGCGER	2	626.2401
sp|P10643|CO7_HUMAN	Complement component C7 OS=Homo sapiens GN=C7 PE=1 SV=2	C7	SLVC[+57]NGDSDC[+57]DEDSADEDR	SLVCNGDSDCDEDSADEDR	2	1079.892
sp|P10643|CO7_HUMAN	Complement component C7 OS=Homo sapiens GN=C7 PE=1 SV=2	C7	SLVC[+57]N[+1]GDSDC[+57]DEDSADEDR	SLVCNGDSDCDEDSADEDR	2	1080.384
sp|P10643|CO7_HUMAN	Complement component C7 OS=Homo sapiens GN=C7 PE=1 SV=2	C7	SYTSHTNEIHK	SYTSHTNEIHK	2	658.8151
sp|P10643|CO7_HUMAN	Complement component C7 OS=Homo sapiens GN=C7 PE=1 SV=2	C7	ELSHLPSLYDYSAYR	ELSHLPSLYDYSAYR	3	605.2967
sp|P10643|CO7_HUMAN	Complement component C7 OS=Homo sapiens GN=C7 PE=1 SV=2	C7	QNDFNSVEEK	QNDFNSVEEK	2	605.2727
sp|P10643|CO7_HUMAN	Complement component C7 OS=Homo sapiens GN=C7 PE=1 SV=2	C7	SSGWHFVVK	SSGWHFVVK	2	523.7745
sp|P10643|CO7_HUMAN	Complement component C7 OS=Homo sapiens GN=C7 PE=1 SV=2	C7	ELENALK	ELENALK	2	408.7267
sp|P10643|CO7_HUMAN	Complement component C7 OS=Homo sapiens GN=C7 PE=1 SV=2	C7	AASGTQNNVLR	AASGTQNNVLR	2	565.7993
sp|P10643|CO7_HUMAN	Complement component C7 OS=Homo sapiens GN=C7 PE=1 SV=2	C7	YSAWAESVTNLPQVIK	YSAWAESVTNLPQVIK	2	903.4753
sp|P10643|CO7_HUMAN	Complement component C7 OS=Homo sapiens GN=C7 PE=1 SV=2	C7	NVVYTC[+57]NEGYSLIGNPVAR	NVVYTCNEGYSLIGNPVAR	2	1063.52
sp|P10643|CO7_HUMAN	Complement component C7 OS=Homo sapiens GN=C7 PE=1 SV=2	C7	N[+1]YTLTGR	NYTLTGR	2	413.2087
sp|P10643|CO7_HUMAN	Complement component C7 OS=Homo sapiens GN=C7 PE=1 SV=2	C7	DSC[+57]TLPASAEK	DSCTLPASAEK	2	589.7715
sp|P10909|CLUS_HUMAN	Clusterin OS=Homo sapiens GN=CLU PE=1 SV=1	CLU	EIQNAVNGVK	EIQNAVNGVK	2	536.2933
sp|P10909|CLUS_HUMAN	Clusterin OS=Homo sapiens GN=CLU PE=1 SV=1	CLU	TLLSNLEEAK	TLLSNLEEAK	2	559.3086
sp|P10909|CLUS_HUMAN	Clusterin OS=Homo sapiens GN=CLU PE=1 SV=1	CLU	EDALN[+1]ETR	EDALNETR	2	474.7171
sp|P10909|CLUS_HUMAN	Clusterin OS=Homo sapiens GN=CLU PE=1 SV=1	CLU	IDSLLENDR	IDSLLENDR	2	537.7749
sp|P10909|CLUS_HUMAN	Clusterin OS=Homo sapiens GN=CLU PE=1 SV=1	CLU	ASSIIDELFQDR	ASSIIDELFQDR	2	697.3515
sp|P10909|CLUS_HUMAN	Clusterin OS=Homo sapiens GN=CLU PE=1 SV=1	CLU	EILSVDC[+57]STNNPSQAK	EILSVDCSTNNPSQAK	2	881.9174
sp|P10909|CLUS_HUMAN	Clusterin OS=Homo sapiens GN=CLU PE=1 SV=1	CLU	LAN[+1]LTQGEDQYYLR	LANLTQGEDQYYLR	2	842.9125
sp|P10909|CLUS_HUMAN	Clusterin OS=Homo sapiens GN=CLU PE=1 SV=1	CLU	LFDSDPITVTVPVEVSR	LFDSDPITVTVPVEVSR	2	937.4989
sp|P11226|MBL2_HUMAN	Mannose-binding protein C OS=Homo sapiens GN=MBL2 PE=1 SV=2	MBL2	EEAFLGITDEK	EEAFLGITDEK	2	626.3088
sp|P12111|CO6A3_HUMAN	Collagen alpha-3(VI) chain OS=Homo sapiens GN=COL6A3 PE=1 SV=5	COL6A3	VAVVQYSDR	VAVVQYSDR	2	518.7747
sp|P13671|CO6_HUMAN	Complement component C6 OS=Homo sapiens GN=C6 PE=1 SV=3	C6	LEC[+57]NGENDC[+57]GDNSDER	LECNGENDCGDNSDER	2	942.342
sp|P13671|CO6_HUMAN	Complement component C6 OS=Homo sapiens GN=C6 PE=1 SV=3	C6	VPANLENVGFEVQTAEDDLK	VPANLENVGFEVQTAEDDLK	2	1094.542
sp|P13671|CO6_HUMAN	Complement component C6 OS=Homo sapiens GN=C6 PE=1 SV=3	C6	DLTSLGHNENQQGSFSSQGGSSFSVPIFYSSK	DLTSLGHNENQQGSFSSQGGSSFSVPIFY SSK	3	1131.527
sp|P13671|CO6_HUMAN	Complement component C6 OS=Homo sapiens GN=C6 PE=1 SV=3	C6	SENINHNSAFK	SENINHNSAFK	2	630.802
sp|P13671|CO6_HUMAN	Complement component C6 OS=Homo sapiens GN=C6 PE=1 SV=3	C6	ALNHLPLEYNSALYSR	ALNHLPLEYNSALYSR	2	930.9838
sp|P13671|CO6_HUMAN	Complement component C6 OS=Homo sapiens GN=C6 PE=1 SV=3	C6	SEYGAALAWEK	SEYGAALAWEK	2	612.7984
sp|P13671|CO6_HUMAN	Complement component C6 OS=Homo sapiens GN=C6 PE=1 SV=3	C6	C[+57]LPDGTWR	CLPDGTWR	2	502.7346
sp|P13671|CO6_HUMAN	Complement component C6 OS=Homo sapiens GN=C6 PE=1 SV=3	C6	IGESIELTC[+57]PK	IGESIELTCPK	2	623.821
sp|P13671|CO6_HUMAN	Complement component C6 OS=Homo sapiens GN=C6 PE=1 SV=3	C6	YTC[+57]QGNSWTPPISNSLTC[+57]EK	YTCQGNSWTPPISNSLTCEK	2	1172.023
sp|P13671|CO6_HUMAN	Complement component C6 OS=Homo sapiens GN=C6 PE=1 SV=3	C6	QLEWGLER	QLEWGLER	2	515.7694
sp|P13671|CO6_HUMAN	Complement component C6 OS=Homo sapiens GN=C6 PE=1 SV=3	C6	ESC[+57]GYDTC[+57]YDWEK	ESCGYDTCYDWEK	2	856.8138
sp|P13671|CO6_HUMAN	Complement component C6 OS=Homo sapiens GN=C6 PE=1 SV=3	C6	GGNQLYC[+57]VK	GGNQLYCVK	2	519.7555
sp|P13984|T2FB_HUMAN	General transcription factor IIF subunit 2 OS=Homo sapiens GN=GTF2F2 PE=1 SV=2	GTF2F2	DLVDITK	DLVDITK	2	402.2291
sp|P14151|LYAM1_HUMAN	L-selectin OS=Homo sapiens GN=SELL PE=1 SV=2	SELL	DN[+1]YTDLVAIQNK	DNYTDLVAIQNK	2	697.8435
sp|P14151|LYAM1_HUMAN	L-selectin OS=Homo sapiens GN=SELL PE=1 SV=2	SELL	AEIEYLEK	AEIEYLEK	2	497.7582
sp|P15169|CBPN_HUMAN	Carboxypeptidase N catalytic chain OS=Homo sapiens GN=CPN1 PE=1 SV=1	CPN1	IVQLIQDTR	IVQLIQDTR	2	543.3193
sp|P15169|CBPN_HUMAN	Carboxypeptidase N catalytic chain OS=Homo sapiens GN=CPN1 PE=1 SV=1	CPN1	SIPQVSPVR	SIPQVSPVR	2	491.7876
sp|P15924|DESP_HUMAN	Desmoplakin OS=Homo sapiens GN=DSP PE=1 SV=3	DSP	QQIYAAEK	QQIYAAEK	2	475.7507
sp|P18428|LBP_HUMAN	Lipopolysaccharide-binding protein OS=Homo sapiens GN=LBP PE=1 SV=3	LBP	LAEGFPLPLLK	LAEGFPLPLLK	2	599.3657
sp|P19823|ITIH2_HUMAN	Inter-alpha-trypsin inhibitor heavy chain H2 OS=Homo sapiens GN=ITIH2 PE=1 SV=2	ITIH2	VQSTITSR	VQSTITSR	2	446.2483
sp|P19823|ITIH2_HUMAN	Inter-alpha-trypsin inhibitor heavy chain H2 OS=Homo sapiens GN=ITIH2 PE=1 SV=2	ITIH2	VVNNSPQPQNVVFDVQIPK	VVNNSPQPQNVVFDVQIPK	2	1061.568
sp|P19823|ITIH2_HUMAN	Inter-alpha-trypsin inhibitor heavy chain H2 OS=Homo sapiens GN=ITIH2 PE=1 SV=2	ITIH2	VQFELHYQEVK	VQFELHYQEVK	2	710.367
sp|P19823|ITIH2_HUMAN	Inter-alpha-trypsin inhibitor heavy chain H2 OS=Homo sapiens GN=ITIH2 PE=1 SV=2	ITIH2	IYLQPGR	IYLQPGR	2	423.7452
sp|P19823|ITIH2_HUMAN	Inter-alpha-trypsin inhibitor heavy chain H2 OS=Homo sapiens GN=ITIH2 PE=1 SV=2	ITIH2	TILDDLR	TILDDLR	2	423.24

sp|P19823|ITIH2_HUMAN	Inter-alpha-trypsin inhibitor heavy chain H2 OS=Homo sapiens GN=ITIH2 PE=1 SV=2	ITIH2	AEDHFSVIDFNQNIR	AEDHFSVIDFNQNIR	2	902.9343
sp|P19823|ITIH2_HUMAN	Inter-alpha-trypsin inhibitor heavy chain H2 OS=Homo sapiens GN=ITIH2 PE=1 SV=2	ITIH2	NDLISATK	NDLISATK	2	431.2375
sp|P19823|ITIH2_HUMAN	Inter-alpha-trypsin inhibitor heavy chain H2 OS=Homo sapiens GN=ITIH2 PE=1 SV=2	ITIH2	IQPSGGTNINEALLR	IQPSGGTNINEALLR	2	791.931
sp|P19823|ITIH2_HUMAN	Inter-alpha-trypsin inhibitor heavy chain H2 OS=Homo sapiens GN=ITIH2 PE=1 SV=2	ITIH2	LSNENHGIAQR	LSNENHGIAQR	2	619.8155
sp|P19823|ITIH2_HUMAN	Inter-alpha-trypsin inhibitor heavy chain H2 OS=Homo sapiens GN=ITIH2 PE=1 SV=2	ITIH2	IYGNQDTSSQLK	IYGNQDTSSQLK	2	677.3359
sp|P19823|ITIH2_HUMAN	Inter-alpha-trypsin inhibitor heavy chain H2 OS=Homo sapiens GN=ITIH2 PE=1 SV=2	ITIH2	FYNQVSTPLLR	FYNQVSTPLLR	2	669.3642
sp|P19823|ITIH2_HUMAN	Inter-alpha-trypsin inhibitor heavy chain H2 OS=Homo sapiens GN=ITIH2 PE=1 SV=2	ITIH2	HADPDFTR	HADPDFTR	2	479.7225
sp|P19823|ITIH2_HUMAN	Inter-alpha-trypsin inhibitor heavy chain H2 OS=Homo sapiens GN=ITIH2 PE=1 SV=2	ITIH2	SLAPTAAAK	SLAPTAAAK	2	415.2425
sp|P19827|ITIH1_HUMAN	Inter-alpha-trypsin inhibitor heavy chain H1 OS=Homo sapiens GN=ITIH1 PE=1 SV=3	ITIH1	FAHYVVTSQVVNTANEAR	FAHYVVTSQVVNTANEAR	2	1003.508
sp|P19827|ITIH1_HUMAN	Inter-alpha-trypsin inhibitor heavy chain H1 OS=Homo sapiens GN=ITIH1 PE=1 SV=3	ITIH1	EVAFDLEIPK	EVAFDLEIPK	2	580.8135
sp|P19827|ITIH1_HUMAN	Inter-alpha-trypsin inhibitor heavy chain H1 OS=Homo sapiens GN=ITIH1 PE=1 SV=3	ITIH1	AAISGENAGLVR	AAISGENAGLVR	2	579.3173
sp|P19827|ITIH1_HUMAN	Inter-alpha-trypsin inhibitor heavy chain H1 OS=Homo sapiens GN=ITIH1 PE=1 SV=3	ITIH1	LDAQASFLPK	LDAQASFLPK	2	545.3006
sp|P19827|ITIH1_HUMAN	Inter-alpha-trypsin inhibitor heavy chain H1 OS=Homo sapiens GN=ITIH1 PE=1 SV=3	ITIH1	VTYDVSR	VTYDVSR	2	420.2165
sp|P20742|PZP_HUMAN	Pregnancy zone protein OS=Homo sapiens GN=PZP PE=1 SV=4	PZP	ISEITNIVSK	ISEITNIVSK	2	552.319
sp|P20742|PZP_HUMAN	Pregnancy zone protein OS=Homo sapiens GN=PZP PE=1 SV=4	PZP	ATVLNYLPK	ATVLNYLPK	2	509.8002
sp|P20742|PZP_HUMAN	Pregnancy zone protein OS=Homo sapiens GN=PZP PE=1 SV=4	PZP	YGAATFTR	YGAATFTR	2	443.7245
sp|P20851|C4BPB_HUMAN	C4b-binding protein beta chain OS=Homo sapiens GN=C4BPB PE=1 SV=1	C4BPB	EWDN[+1]TTTEC[+57]R	EWDNTTTECR	2	656.7591
sp|P20851|C4BPB_HUMAN	C4b-binding protein beta chain OS=Homo sapiens GN=C4BPB PE=1 SV=1	C4BPB	LGHC[+57]PDPVLVNGEFSSSGPVN[+1]VSDK	LGHCPDPVLVNGEFSSSGPVNVSDK	3	871.4148
sp|P20851|C4BPB_HUMAN	C4b-binding protein beta chain OS=Homo sapiens GN=C4BPB PE=1 SV=1	C4BPB	LGHC[+57]PDPVLVN[+1]GEFSSSGPVN[+1]VSDK	LGHCPDPVLVNGEFSSSGPVNVSDK	3	871.7428
sp|P20851|C4BPB_HUMAN	C4b-binding protein beta chain OS=Homo sapiens GN=C4BPB PE=1 SV=1	C4BPB	SQC[+57]LEDHTWAPPFPIC[+57]K	SQCLEDHTWAPPFPICK	3	695.9889
sp|P20851|C4BPB_HUMAN	C4b-binding protein beta chain OS=Homo sapiens GN=C4BPB PE=1 SV=1	C4BPB	LIQEAPKPEC[+57]EK	LIQEAPKPECEK	2	721.3714
sp|P20851|C4BPB_HUMAN	C4b-binding protein beta chain OS=Homo sapiens GN=C4BPB PE=1 SV=1	C4BPB	ALLAFQESK	ALLAFQESK	2	503.782
sp|P21333|FLNA_HUMAN	Filamin-A OS=Homo sapiens GN=FLNA PE=1 SV=4	FLNA	FGGEHVPNSPFQVTALAGDQPSVQPPLR	FGGEHVPNSPFQVTALAGDQPSVQPPLR	3	982.5017
sp|P21333|FLNA_HUMAN	Filamin-A OS=Homo sapiens GN=FLNA PE=1 SV=4	FLNA	ATC[+57]APQHGAPGPGPADASK	ATCAPQHGAPGPGPADASK	3	597.2811
sp|P22352|GPX3_HUMAN	Glutathione peroxidase 3 OS=Homo sapiens GN=GPX3 PE=1 SV=2	GPX3	QEPGENSEILPTLK	QEPGENSEILPTLK	2	777.9041
sp|P22792|CPN2_HUMAN	Carboxypeptidase N subunit 2 OS=Homo sapiens GN=CPN2 PE=1 SV=3	CPN2	AFGSNPN[+1]LTK	AFGSNPNLTK	2	525.2667
sp|P22792|CPN2_HUMAN	Carboxypeptidase N subunit 2 OS=Homo sapiens GN=CPN2 PE=1 SV=3	CPN2	LSNNALSGLPQGVFGK	LSNNALSGLPQGVFGK	2	801.4359
sp|P22792|CPN2_HUMAN	Carboxypeptidase N subunit 2 OS=Homo sapiens GN=CPN2 PE=1 SV=3	CPN2	DLEELVK	DLEELVK	2	423.2344
sp|P22792|CPN2_HUMAN	Carboxypeptidase N subunit 2 OS=Homo sapiens GN=CPN2 PE=1 SV=3	CPN2	LYLGSNN[+1]LTALHPALFQN[+1]LSK	LYLGSNNLTALHPALFQNLSK	3	772.746
sp|P22792|CPN2_HUMAN	Carboxypeptidase N subunit 2 OS=Homo sapiens GN=CPN2 PE=1 SV=3	CPN2	LELLSLSK	LELLSLSK	2	451.7815
sp|P22792|CPN2_HUMAN	Carboxypeptidase N subunit 2 OS=Homo sapiens GN=CPN2 PE=1 SV=3	CPN2	LLNIQTYC[+57]AGPAYLK	LLNIQTYCAGPAYLK	2	862.9556
sp|P22792|CPN2_HUMAN	Carboxypeptidase N subunit 2 OS=Homo sapiens GN=CPN2 PE=1 SV=3	CPN2	QLVC[+57]PVTR	QLVCPVTR	2	486.7684
sp|P22792|CPN2_HUMAN	Carboxypeptidase N subunit 2 OS=Homo sapiens GN=CPN2 PE=1 SV=3	CPN2	SQC[+57]TYSNPEGTVVLAC[+57]DQAQC[+57]R	SQCTYSNPEGTVVLACDQAQCR	3	848.7019
sp|P23142|FBLN1_HUMAN	Fibulin-1 OS=Homo sapiens GN=FBLN1 PE=1 SV=4	FBLN1	SQETGDLDVGGLQETDK	SQETGDLDVGGLQETDK	2	896.4158
sp|P23142|FBLN1_HUMAN	Fibulin-1 OS=Homo sapiens GN=FBLN1 PE=1 SV=4	FBLN1	IIEVEEEQEDPYLNDR	IIEVEEEQEDPYLNDR	2	995.9656
sp|P23142|FBLN1_HUMAN	Fibulin-1 OS=Homo sapiens GN=FBLN1 PE=1 SV=4	FBLN1	DSSC[+57]GTGYELTEDNSC[+57]K	DSSCGTGYELTEDNSCK	2	961.8726
sp|P25311|ZA2G_HUMAN	Zinc-alpha-2-glycoprotein OS=Homo sapiens GN=AZGP1 PE=1 SV=2	AZGP1	DYIEFNK	DYIEFNK	2	464.7242
sp|P25311|ZA2G_HUMAN	Zinc-alpha-2-glycoprotein OS=Homo sapiens GN=AZGP1 PE=1 SV=2	AZGP1	EIPAWVPFDPAAQITK	EIPAWVPFDPAAQITK	2	891.9749
sp|P25311|ZA2G_HUMAN	Zinc-alpha-2-glycoprotein OS=Homo sapiens GN=AZGP1 PE=1 SV=2	AZGP1	AGEVQEPELR	AGEVQEPELR	2	564.2882
sp|P26927|HGFL_HUMAN	Hepatocyte growth factor-like protein OS=Homo sapiens GN=MST1 PE=1 SV=2	MST1	EFC[+57]DLPR	EFCDLPR	2	468.7158
sp|P26927|HGFL_HUMAN	Hepatocyte growth factor-like protein OS=Homo sapiens GN=MST1 PE=1 SV=2	MST1	GTAN[+1]TTTAGVPC[+57]QR	GTANTTTAGVPCQR	2	717.8357
sp|P27169|PON1_HUMAN	Serum paraoxonase/arylesterase 1 OS=Homo sapiens GN=PON1 PE=1 SV=3	PON1	EVQPVELPNC[+57]NLVK	EVQPVELPNCNLVK	2	819.9296
sp|P27169|PON1_HUMAN	Serum paraoxonase/arylesterase 1 OS=Homo sapiens GN=PON1 PE=1 SV=3	PON1	SFNPNSPGK	SFNPNSPGK	2	474.2327
sp|P27169|PON1_HUMAN	Serum paraoxonase/arylesterase 1 OS=Homo sapiens GN=PON1 PE=1 SV=3	PON1	YVYIAELLAHK	YVYIAELLAHK	2	660.3715
sp|P27169|PON1_HUMAN	Serum paraoxonase/arylesterase 1 OS=Homo sapiens GN=PON1 PE=1 SV=3	PON1	IFFYDSENPPASEVLR	IFFYDSENPPASEVLR	2	942.4623
sp|P27169|PON1_HUMAN	Serum paraoxonase/arylesterase 1 OS=Homo sapiens GN=PON1 PE=1 SV=3	PON1	IQNILTEEPK	IQNILTEEPK	2	592.8297
sp|P27348|1433T_HUMAN	14-3-3 protein theta OS=Homo sapiens GN=YWHAQ PE=1 SV=1	YWHAQ	NLLSVAYK	NLLSVAYK	2	454.266
sp|P27918|PROP_HUMAN	Properdin OS=Homo sapiens GN=CFP PE=1 SV=2	CFP	SISC[+57]QEIPGQQSR	SISCQEIPGQQSR	2	745.3568
sp|P27918|PROP_HUMAN	Properdin OS=Homo sapiens GN=CFP PE=1 SV=2	CFP	LC[+57]TPLLPK	LCTPLLPK	2	471.2781
sp|P29622|KAIN_HUMAN	Kallistatin OS=Homo sapiens GN=SERPINA4 PE=1 SV=3	SERPINA4	IAPANADFAFR	IAPANADFAFR	2	596.8091
sp|P29622|KAIN_HUMAN	Kallistatin OS=Homo sapiens GN=SERPINA4 PE=1 SV=3	SERPINA4	ALWEKPFISSR	ALWEKPFISSR	2	667.3668
sp|P29622|KAIN_HUMAN	Kallistatin OS=Homo sapiens GN=SERPINA4 PE=1 SV=3	SERPINA4	LGFTDLFSK	LGFTDLFSK	2	514.2766
sp|P29622|KAIN_HUMAN	Kallistatin OS=Homo sapiens GN=SERPINA4 PE=1 SV=3	SERPINA4	WADLSGITK	WADLSGITK	2	495.7664
sp|P29622|KAIN_HUMAN	Kallistatin OS=Homo sapiens GN=SERPINA4 PE=1 SV=3	SERPINA4	FFSAQTNR	FFSAQTNR	2	485.7407
sp|P35542|SAA4_HUMAN	Serum amyloid A-4 protein OS=Homo sapiens GN=SAA4 PE=1 SV=2	SAA4	GNYDAAQR	GNYDAAQR	2	447.7068
sp|P35858|ALS_HUMAN	Insulin-like growth factor-binding protein complex acid labile subunit OS=Homo sapiens GN=IGFALS PE=1 SV=1	IGFALS	ELVLAGNR	ELVLAGNR	2	436.2534
sp|P35858|ALS_HUMAN	Insulin-like growth factor-binding protein complex acid labile subunit OS=Homo sapiens GN=IGFALS PE=1 SV=1	IGFALS	ANVFVQLPR	ANVFVQLPR	2	522.3035
sp|P35858|ALS_HUMAN	Insulin-like growth factor-binding protein complex acid labile subunit OS=Homo sapiens GN=IGFALS PE=1 SV=1	IGFALS	LEYLLLSR	LEYLLLSR	2	503.8002
sp|P35858|ALS_HUMAN	Insulin-like growth factor-binding protein complex acid labile subunit OS=Homo sapiens GN=IGFALS PE=1 SV=1	IGFALS	DFALQNPSAVPR	DFALQNPSAVPR	2	657.8437
sp|P35858|ALS_HUMAN	Insulin-like growth factor-binding protein complex acid labile subunit OS=Homo sapiens GN=IGFALS PE=1 SV=1	IGFALS	DLSEAHFAPC[+57]	DLSEAHFAPC	2	573.7479
sp|P36955|PEDF_HUMAN	Pigment epithelium-derived factor OS=Homo sapiens GN=SERPINF1 PE=1 SV=4	SERPINF1	TESIIHR	TESIIHR	2	428.2378
sp|P36955|PEDF_HUMAN	Pigment epithelium-derived factor OS=Homo sapiens GN=SERPINF1 PE=1 SV=4	SERPINF1	ALYYDLISSPDIHGTYK	ALYYDLISSPDIHGTYK	2	978.4911

sp|P36955|PEDF_HUMAN	Pigment epithelium-derived factor OS=Homo sapiens GN=SERPINF1 PE=1 SV=4	SERPINF1	ELLDTVTAPQK	ELLDTVTAPQK	2	607.835
sp|P36955|PEDF_HUMAN	Pigment epithelium-derived factor OS=Homo sapiens GN=SERPINF1 PE=1 SV=4	SERPINF1	SSFVAPLEK	SSFVAPLEK	2	489.2688
sp|P36955|PEDF_HUMAN	Pigment epithelium-derived factor OS=Homo sapiens GN=SERPINF1 PE=1 SV=4	SERPINF1	TSLEDFYLDEER	TSLEDFYLDEER	2	758.8437
sp|P36955|PEDF_HUMAN	Pigment epithelium-derived factor OS=Homo sapiens GN=SERPINF1 PE=1 SV=4	SERPINF1	TVQAVLTVPK	TVQAVLTVPK	2	528.3266
sp|P36980|FHR2_HUMAN	Complement factor H-related protein 2 OS=Homo sapiens GN=CFHR2 PE=1 SV=1	CFHR2	INHGILYDEEK	INHGILYDEEK	2	665.8355
sp|P41222|PTGDS_HUMAN	Prostaglandin-H2 D-isomerase OS=Homo sapiens GN=PTGDS PE=1 SV=1	PTGDS	AQGFTEDTIVFLPQTDK	AQGFTEDTIVFLPQTDK	2	955.4807
sp|P43251|BTD_HUMAN	Biotinidase OS=Homo sapiens GN=BTD PE=1 SV=2	BTD	SHLIIAQVAK	SHLIIAQVAK	2	540.3322
sp|P43251|BTD_HUMAN	Biotinidase OS=Homo sapiens GN=BTD PE=1 SV=2	BTD	NPVGLIGAEN[+1]ATGETDPSHSK	NPVGLIGAENATGETDPSHSK	2	1048.001
sp|P43251|BTD_HUMAN	Biotinidase OS=Homo sapiens GN=BTD PE=1 SV=2	BTD	LSSGLVTAALYGR	LSSGLVTAALYGR	2	654.3695
sp|P43652|AFAM_HUMAN	Afamin OS=Homo sapiens GN=AFM PE=1 SV=1	AFM	SDVGFLPPFPTLDPEEK	SDVGFLPPFPTLDPEEK	3	629.984
sp|P43652|AFAM_HUMAN	Afamin OS=Homo sapiens GN=AFM PE=1 SV=1	AFM	ESLLNHFLYEVAR	ESLLNHFLYEVAR	2	795.9174
sp|P43652|AFAM_HUMAN	Afamin OS=Homo sapiens GN=AFM PE=1 SV=1	AFM	AIPVTQYLK	AIPVTQYLK	2	516.808
sp|P43652|AFAM_HUMAN	Afamin OS=Homo sapiens GN=AFM PE=1 SV=1	AFM	AFSSYQK	AFSSYQK	2	415.7058
sp|P43652|AFAM_HUMAN	Afamin OS=Homo sapiens GN=AFM PE=1 SV=1	AFM	HVC[+57]GALLK	HVCGALLK	2	449.2524
sp|P43652|AFAM_HUMAN	Afamin OS=Homo sapiens GN=AFM PE=1 SV=1	AFM	GQC[+57]IINSNK	GQCIINSNK	2	517.2584
sp|P43652|AFAM_HUMAN	Afamin OS=Homo sapiens GN=AFM PE=1 SV=1	AFM	FTDSENVC[+57]QER	FTDSENVCQER	2	692.7935
sp|P43652|AFAM_HUMAN	Afamin OS=Homo sapiens GN=AFM PE=1 SV=1	AFM	DADPDTFFAK	DADPDTFFAK	2	563.7562
sp|P43652|AFAM_HUMAN	Afamin OS=Homo sapiens GN=AFM PE=1 SV=1	AFM	FTFEYSR	FTFEYSR	2	475.2243
sp|P43652|AFAM_HUMAN	Afamin OS=Homo sapiens GN=AFM PE=1 SV=1	AFM	N[+1]C[+57]C[+57]NTENPPGC[+57]YR	NCCNTENPPGCYR	2	821.8058
sp|P43652|AFAM_HUMAN	Afamin OS=Homo sapiens GN=AFM PE=1 SV=1	AFM	HFQNLGK	HFQNLGK	2	422.2272
sp|P43652|AFAM_HUMAN	Afamin OS=Homo sapiens GN=AFM PE=1 SV=1	AFM	IAPQLSTEELVSLGEK	IAPQLSTEELVSLGEK	2	857.4671
sp|P43652|AFAM_HUMAN	Afamin OS=Homo sapiens GN=AFM PE=1 SV=1	AFM	TINPAVDHC[+57]C[+57]K	TINPAVDHCCK	2	657.8001
sp|P43652|AFAM_HUMAN	Afamin OS=Homo sapiens GN=AFM PE=1 SV=1	AFM	FLVNLVK	FLVNLVK	2	416.7682
sp|P43652|AFAM_HUMAN	Afamin OS=Homo sapiens GN=AFM PE=1 SV=1	AFM	AESPEVC[+57]FNEESPK	AESPEVCFNEESPK	2	811.8538
sp|P49747|COMP_HUMAN	Cartilage oligomeric matrix protein OS=Homo sapiens GN=COMP PE=1 SV=2	COMP	LVPNPGQEDADR	LVPNPGQEDADR	2	655.8204
sp|P49908|SEPP1_HUMAN	Selenoprotein P OS=Homo sapiens GN=SEPP1 PE=1 SV=3	SEPP1	EGYSN[+1]ISYIVVNHQGISSR	EGYSNISYIVVNHQGISSR	3	708.6834
sp|P49908|SEPP1_HUMAN	Selenoprotein P OS=Homo sapiens GN=SEPP1 PE=1 SV=3	SEPP1	DDFLIYDR	DDFLIYDR	2	528.7535
sp|P51884|LUM_HUMAN	Lumican OS=Homo sapiens GN=LUM PE=1 SV=2	LUM	NNQIDHIDEK	NNQIDHIDEK	2	613.294
sp|P51884|LUM_HUMAN	Lumican OS=Homo sapiens GN=LUM PE=1 SV=2	LUM	LHINHNN[+1]LTESVGPLPK	LHINHNNLTESVGPLPK	3	628.6706
sp|P51884|LUM_HUMAN	Lumican OS=Homo sapiens GN=LUM PE=1 SV=2	LUM	SLEDLQLTHNK	SLEDLQLTHNK	2	649.341
sp|P51884|LUM_HUMAN	Lumican OS=Homo sapiens GN=LUM PE=1 SV=2	LUM	ISNIPDEYFK	ISNIPDEYFK	2	613.3086
sp|P51884|LUM_HUMAN	Lumican OS=Homo sapiens GN=LUM PE=1 SV=2	LUM	FNALQYLR	FNALQYLR	2	512.7824
sp|P51884|LUM_HUMAN	Lumican OS=Homo sapiens GN=LUM PE=1 SV=2	LUM	NIPTVNENLENYYLEVNQLEK	NIPTVNENLENYYLEVNQLEK	3	846.0904
sp|P51884|LUM_HUMAN	Lumican OS=Homo sapiens GN=LUM PE=1 SV=2	LUM	ILGPLSYSK	ILGPLSYSK	2	489.2869
sp|P55056|APOC4_HUMAN	Apolipoprotein C-IV OS=Homo sapiens GN=APOC4 PE=1 SV=1	APOC4	ELLETVVNR	ELLETVVNR	2	536.8035
sp|P55056|APOC4_HUMAN	Apolipoprotein C-IV OS=Homo sapiens GN=APOC4 PE=1 SV=1	APOC4	ELLETVVN[+1]R	ELLETVVNR	2	537.2955
sp|P55056|APOC4_HUMAN	Apolipoprotein C-IV OS=Homo sapiens GN=APOC4 PE=1 SV=1	APOC4	AWFLESK	AWFLESK	2	440.7318
sp|P55058|PLTP_HUMAN	Phospholipid transfer protein OS=Homo sapiens GN=PLTP PE=1 SV=1	PLTP	VSN[+1]VSC[+57]QASVSR	VSNVSCQASVSR	2	647.8064
sp|P60174|TPIS_HUMAN	Triosephosphate isomerase OS=Homo sapiens GN=TPI1 PE=1 SV=3	TPI1	TATPQQAQEVHEK	TATPQQAQEVHEK	3	489.5793
sp|P61626|LYSC_HUMAN	Lysozyme C OS=Homo sapiens GN=LYZ PE=1 SV=1	LYZ	WESGYNTR	WESGYNTR	2	506.7278
sp|P62328|TYB4_HUMAN	Thymosin beta-4 OS=Homo sapiens GN=TMSB4X PE=1 SV=2	TMSB4X	ETIEQEK	ETIEQEK	2	438.7191
sp|P62937|PPIA_HUMAN	Peptidyl-prolyl cis-trans isomerase A OS=Homo sapiens GN=PPIA PE=1 SV=2	PPIA	FEDENFILK	FEDENFILK	2	577.79
sp|P63104|1433Z_HUMAN	14-3-3 protein zeta/delta OS=Homo sapiens GN=YWHAZ PE=1 SV=1	YWHAZ	FLIPNASQAESK	FLIPNASQAESK	2	652.8459
sp|P67936|TPM4_HUMAN	Tropomyosin alpha-4 chain OS=Homo sapiens GN=TPM4 PE=1 SV=3	TPM4	AEGDVAALNR	AEGDVAALNR	2	508.262
sp|P68431|H31_HUMAN	Histone H3.1 OS=Homo sapiens GN=HIST1H3A PE=1 SV=2	HIST1H3A	STELLIR	STELLIR	2	416.2504
sp|P68871|HBB_HUMAN	Hemoglobin subunit beta OS=Homo sapiens GN=HBB PE=1 SV=2	HBB	SAVTALWGK	SAVTALWGK	2	466.7636
sp|P68871|HBB_HUMAN	Hemoglobin subunit beta OS=Homo sapiens GN=HBB PE=1 SV=2	HBB	GTFATLSELHC[+57]DK	GTFATLSELHCDK	2	739.8508
sp|P68871|HBB_HUMAN	Hemoglobin subunit beta OS=Homo sapiens GN=HBB PE=1 SV=2	HBB	EFTPPVQAAYQK	EFTPPVQAAYQK	2	689.8537
sp|P69905|HBA_HUMAN	Hemoglobin subunit alpha OS=Homo sapiens GN=HBA1 PE=1 SV=2	HBA1	TYFPHFDLSHGSAQVK	TYFPHFDLSHGSAQVK	2	917.4496
sp|P80108|PHLD_HUMAN	Phosphatidylinositol-glycan-specific phospholipase D OS=Homo sapiens GN=GPLD1 PE=1 SV=3	GPLD1	N[+1]LTTSLTESVDR	NLTTSLTESVDR	2	668.8332
sp|P80108|PHLD_HUMAN	Phosphatidylinositol-glycan-specific phospholipase D OS=Homo sapiens GN=GPLD1 PE=1 SV=3	GPLD1	QVLLVGAPTYDDVSK	QVLLVGAPTYDDVSK	2	802.9301
sp|P80108|PHLD_HUMAN	Phosphatidylinositol-glycan-specific phospholipase D OS=Homo sapiens GN=GPLD1 PE=1 SV=3	GPLD1	SWITPC[+57]PEEK	SWITPCPEEK	2	623.7923
sp|Q02153|GCYB1_HUMAN	Guanylate cyclase soluble subunit beta-1 OS=Homo sapiens GN=GUCY1B3 PE=1 SV=1	GUCY1B3	FDTLTDSR	FDTLTDSR	2	477.73
sp|Q03591|FHR1_HUMAN	Complement factor H-related protein 1 OS=Homo sapiens GN=CFHR1 PE=1 SV=2	CFHR1	ITC[+57]TEEGWSPTPK	ITCTEEGWSPTPK	2	753.3507
sp|Q03591|FHR1_HUMAN	Complement factor H-related protein 1 OS=Homo sapiens GN=CFHR1 PE=1 SV=2	CFHR1	TGESAEFVC[+57]K	TGESAEFVCK	2	564.2555
sp|Q04756|HGFA_HUMAN	Hepatocyte growth factor activator OS=Homo sapiens GN=HGFAC PE=1 SV=1	HGFAC	YEYLEGGDR	YEYLEGGDR	2	551.246
sp|Q04756|HGFA_HUMAN	Hepatocyte growth factor activator OS=Homo sapiens GN=HGFAC PE=1 SV=1	HGFAC	TWC[+57]EGTR	TWCEGTR	2	455.1978
sp|Q04756|HGFA_HUMAN	Hepatocyte growth factor activator OS=Homo sapiens GN=HGFAC PE=1 SV=1	HGFAC	DSALSWEYC[+57]R	DSALSWEYCR	2	643.7771
sp|Q04756|HGFA_HUMAN	Hepatocyte growth factor activator OS=Homo sapiens GN=HGFAC PE=1 SV=1	HGFAC	SQFVQPIC[+57]LPEPGSTFPAGHK	SQFVQPICLPEPGSTFPAGHK	3	766.3843
sp|Q06033|ITIH3_HUMAN	Inter-alpha-trypsin inhibitor heavy chain H3 OS=Homo sapiens GN=ITIH3 PE=1 SV=2	ITIH3	SLPEGVANGIEVYSTK	SLPEGVANGIEVYSTK	2	832.4305
sp|Q06033|ITIH3_HUMAN	Inter-alpha-trypsin inhibitor heavy chain H3 OS=Homo sapiens GN=ITIH3 PE=1 SV=2	ITIH3	SLPEGVAN[+1]GIEVYSTK	SLPEGVANGIEVYSTK	2	832.9225
sp|Q06033|ITIH3_HUMAN	Inter-alpha-trypsin inhibitor heavy chain H3 OS=Homo sapiens GN=ITIH3 PE=1 SV=2	ITIH3	EVSFDVELPK	EVSFDVELPK	2	581.8032
sp|Q06033|ITIH3_HUMAN	Inter-alpha-trypsin inhibitor heavy chain H3 OS=Homo sapiens GN=ITIH3 PE=1 SV=2	ITIH3	VTFELTYEELLK	VTFELTYEELLK	2	742.8978
sp|Q06033|ITIH3_HUMAN	Inter-alpha-trypsin inhibitor heavy chain H3 OS=Homo sapiens GN=ITIH3 PE=1 SV=2	ITIH3	GHVSFKPSLDQQR	GHVSFKPSLDQQR	2	749.8917
sp|Q06033|ITIH3_HUMAN	Inter-alpha-trypsin inhibitor heavy chain H3 OS=Homo sapiens GN=ITIH3 PE=1 SV=2	ITIH3	EHLVQATPENLQEAR	EHLVQATPENLQEAR	2	867.9421
sp|Q06033|ITIH3_HUMAN	Inter-alpha-trypsin inhibitor heavy chain H3 OS=Homo sapiens GN=ITIH3 PE=1 SV=2	ITIH3	DYIFGNYIER	DYIFGNYIER	2	645.3117
sp|Q06033|ITIH3_HUMAN	Inter-alpha-trypsin inhibitor heavy chain H3 OS=Homo sapiens GN=ITIH3 PE=1 SV=2	ITIH3	VSDIRPGSDPTKPDATLVVK	VSDIRPGSDPTKPDATLVVK	3	699.0513
sp|Q06033|ITIH3_HUMAN	Inter-alpha-trypsin inhibitor heavy chain H3 OS=Homo sapiens GN=ITIH3 PE=1 SV=2	ITIH3	NHQLIVTR	NHQLIVTR	2	490.7854
sp|Q08380|LG3BP_HUMAN	Galectin-3-binding protein OS=Homo sapiens GN=LGALS3BP PE=1 SV=1	LGALS3BP	DAGVVC[+57]TN[+1]ETR	DAGVVCTNETR	2	611.7721
sp|Q08380|LG3BP_HUMAN	Galectin-3-binding protein OS=Homo sapiens GN=LGALS3BP PE=1 SV=1	LGALS3BP	IDITLSSVK	IDITLSSVK	2	488.2897
sp|Q08380|LG3BP_HUMAN	Galectin-3-binding protein OS=Homo sapiens GN=LGALS3BP PE=1 SV=1	LGALS3BP	AVDTWSWGER	AVDTWSWGER	2	603.7805
sp|Q08380|LG3BP_HUMAN	Galectin-3-binding protein OS=Homo sapiens GN=LGALS3BP PE=1 SV=1	LGALS3BP	ASHEEVEGLVEK	ASHEEVEGLVEK	2	663.8304

sp|Q08380|LG3BP_HUMAN	Galectin-3-binding protein OS=Homo sapiens GN=LGALS3BP PE=1 SV=1	LGALS3BP	GLN[+1]LTEDTYKPR	GLNLTEDTYKPR	2	704.3594
sp|Q08380|LG3BP_HUMAN	Galectin-3-binding protein OS=Homo sapiens GN=LGALS3BP PE=1 SV=1	LGALS3BP	IYTSPTWSAFVTDSSWSAR	IYTSPTWSAFVTDSSWSAR	2	1081.513
sp|Q08380|LG3BP_HUMAN	Galectin-3-binding protein OS=Homo sapiens GN=LGALS3BP PE=1 SV=1	LGALS3BP	AAIPSALDTN[+1]SSK	AAIPSALDTNSSK	2	638.325
sp|Q12805|FBLN3_HUMAN	EGF-containing fibulin-like extracellular matrix protein 1 OS=Homo sapiens GN=EFEMP1 PE=1 SV=2	EFEMP1	ADQVC[+57]INLR	ADQVCINLR	2	544.7795
sp|Q12805|FBLN3_HUMAN	EGF-containing fibulin-like extracellular matrix protein 1 OS=Homo sapiens GN=EFEMP1 PE=1 SV=2	EFEMP1	GSFAC[+57]QC[+57]PPGYQK	GSFACQCPPGYQK	2	750.3239
sp|Q13201|MMRN1_HUMAN	Multimerin-1 OS=Homo sapiens GN=MMRN1 PE=1 SV=3	MMRN1	FNPGAESVVLSN[+1]STLK	FNPGAESVVLSNSTLK	2	832.4305
sp|Q13790|APOF_HUMAN	Apolipoprotein F OS=Homo sapiens GN=APOF PE=1 SV=2	APOF	SLPTEDC[+57]ENEK	SLPTEDCENEK	2	661.2825
sp|Q14520|HABP2_HUMAN	Hyaluronan-binding protein 2 OS=Homo sapiens GN=HABP2 PE=1 SV=1	HABP2	FC[+57]EIGSDDC[+57]YVGDGYSYR	FCEIGSDDCYVGDGYSYR	2	1081.925
sp|Q14520|HABP2_HUMAN	Hyaluronan-binding protein 2 OS=Homo sapiens GN=HABP2 PE=1 SV=1	HABP2	VVLGDQDLK	VVLGDQDLK	2	493.7795
sp|Q14520|HABP2_HUMAN	Hyaluronan-binding protein 2 OS=Homo sapiens GN=HABP2 PE=1 SV=1	HABP2	DEIPHNDIALLK	DEIPHNDIALLK	2	689.3723
sp|Q14624|ITIH4_HUMAN	Inter-alpha-trypsin inhibitor heavy chain H4 OS=Homo sapiens GN=ITIH4 PE=1 SV=4	ITIH4	NGIDIYSLTVDSR	NGIDIYSLTVDSR	2	726.8701
sp|Q14624|ITIH4_HUMAN	Inter-alpha-trypsin inhibitor heavy chain H4 OS=Homo sapiens GN=ITIH4 PE=1 SV=4	ITIH4	FAHTVVTSR	FAHTVVTSR	2	509.2774
sp|Q14624|ITIH4_HUMAN	Inter-alpha-trypsin inhibitor heavy chain H4 OS=Homo sapiens GN=ITIH4 PE=1 SV=4	ITIH4	LGVYELLLK	LGVYELLLK	2	524.3261
sp|Q14624|ITIH4_HUMAN	Inter-alpha-trypsin inhibitor heavy chain H4 OS=Homo sapiens GN=ITIH4 PE=1 SV=4	ITIH4	VRPQQLVK	VRPQQLVK	2	484.306
sp|Q14624|ITIH4_HUMAN	Inter-alpha-trypsin inhibitor heavy chain H4 OS=Homo sapiens GN=ITIH4 PE=1 SV=4	ITIH4	FKPTLSQQQK	FKPTLSQQQK	2	602.8379
sp|Q14624|ITIH4_HUMAN	Inter-alpha-trypsin inhibitor heavy chain H4 OS=Homo sapiens GN=ITIH4 PE=1 SV=4	ITIH4	NVVFVIDK	NVVFVIDK	2	467.2738
sp|Q14624|ITIH4_HUMAN	Inter-alpha-trypsin inhibitor heavy chain H4 OS=Homo sapiens GN=ITIH4 PE=1 SV=4	ITIH4	ILDDLSPR	ILDDLSPR	2	464.7585
sp|Q14624|ITIH4_HUMAN	Inter-alpha-trypsin inhibitor heavy chain H4 OS=Homo sapiens GN=ITIH4 PE=1 SV=4	ITIH4	SIQNNVR	SIQNNVR	2	415.7276
sp|Q14624|ITIH4_HUMAN	Inter-alpha-trypsin inhibitor heavy chain H4 OS=Homo sapiens GN=ITIH4 PE=1 SV=4	ITIH4	LALDNGGLAR	LALDNGGLAR	2	500.2827
sp|Q14624|ITIH4_HUMAN	Inter-alpha-trypsin inhibitor heavy chain H4 OS=Homo sapiens GN=ITIH4 PE=1 SV=4	ITIH4	GPDVLTATVSGK	GPDVLTATVSGK	2	572.8141
sp|Q14624|ITIH4_HUMAN	Inter-alpha-trypsin inhibitor heavy chain H4 OS=Homo sapiens GN=ITIH4 PE=1 SV=4	ITIH4	NVHSGSTFFK	NVHSGSTFFK	2	562.2802
sp|Q14624|ITIH4_HUMAN	Inter-alpha-trypsin inhibitor heavy chain H4 OS=Homo sapiens GN=ITIH4 PE=1 SV=4	ITIH4	YYLQGAK	YYLQGAK	2	421.724
sp|Q14624|ITIH4_HUMAN	Inter-alpha-trypsin inhibitor heavy chain H4 OS=Homo sapiens GN=ITIH4 PE=1 SV=4	ITIH4	QGPVNLLSDPEQGVEVTGQYER	QGPVNLLSDPEQGVEVTGQYER	3	805.7311
sp|Q14624|ITIH4_HUMAN	Inter-alpha-trypsin inhibitor heavy chain H4 OS=Homo sapiens GN=ITIH4 PE=1 SV=4	ITIH4	TGLLLLSDPDK	TGLLLLSDPDK	2	586.3321
sp|Q15848|ADIPO_HUMAN	Adiponectin OS=Homo sapiens GN=ADIPOQ PE=1 SV=1	ADIPOQ	GDIGETGVPGAEGPR	GDIGETGVPGAEGPR	2	706.3442
sp|Q15848|ADIPO_HUMAN	Adiponectin OS=Homo sapiens GN=ADIPOQ PE=1 SV=1	ADIPOQ	IFYNQQNHYDGSTGK	IFYNQQNHYDGSTGK	3	591.2727
sp|Q16610|ECM1_HUMAN	Extracellular matrix protein 1 OS=Homo sapiens GN=ECM1 PE=1 SV=2	ECM1	LLPAQLPAEK	LLPAQLPAEK	2	540.3266
sp|Q16610|ECM1_HUMAN	Extracellular matrix protein 1 OS=Homo sapiens GN=ECM1 PE=1 SV=2	ECM1	EVGPPLPQEAVPLQK	EVGPPLPQEAVPLQK	2	801.4485
sp|Q16610|ECM1_HUMAN	Extracellular matrix protein 1 OS=Homo sapiens GN=ECM1 PE=1 SV=2	ECM1	ELPSLQHPNEQK	ELPSLQHPNEQK	2	710.365
sp|Q16610|ECM1_HUMAN	Extracellular matrix protein 1 OS=Homo sapiens GN=ECM1 PE=1 SV=2	ECM1	FSC[+57]FQEEAPQPHYQLR	FSCFQEEAPQPHYQLR	3	679.6476
sp|Q16610|ECM1_HUMAN	Extracellular matrix protein 1 OS=Homo sapiens GN=ECM1 PE=1 SV=2	ECM1	NVALVSGDTENAK	NVALVSGDTENAK	2	659.3359
sp|Q16635|TAZ_HUMAN	Tafazzin OS=Homo sapiens GN=TAZ PE=1 SV=1	TAZ	EVLYELIEK	EVLYELIEK	2	568.3159
sp|Q5T1H1|EYS_HUMAN	Protein eyes shut homolog OS=Homo sapiens GN=EYS PE=1 SV=5	EYS	FLN[+1]FGIR	FLNFGIR	2	434.2398
sp|Q5T7N2|LITD1_HUMAN	LINE-1 type transposase domain-containing protein 1 OS=Homo sapiens GN=L1TD1 PE=1 SV=1	L1TD1	DIAPVLDLK	DIAPVLDLK	2	492.2922
sp|Q5TCS8|KAD9_HUMAN	Adenylate kinase 9 OS=Homo sapiens GN=AK9 PE=1 SV=2	AK9	ITSEYGLK	ITSEYGLK	2	455.7477
sp|Q8N3Z3|GTPB8_HUMAN	GTP-binding protein 8 OS=Homo sapiens GN=GTPBP8 PE=2 SV=1	GTPBP8	C[+57]FIASVTGSLD	CFIASVTGSLD	2	585.279
sp|Q8NF91|SYNE1_HUMAN	Nesprin-1 OS=Homo sapiens GN=SYNE1 PE=1 SV=4	SYNE1	LSEFSLLK	LSEFSLLK	2	468.7737
sp|Q8TD26|CHD6_HUMAN	Chromodomain-helicase-DNA-binding protein 6 OS=Homo sapiens GN=CHD6 PE=1 SV=4	CHD6	AVLQDINR	AVLQDINR	2	464.7642
sp|Q8WUU4|ZN296_HUMAN	Zinc finger protein 296 OS=Homo sapiens GN=ZNF296 PE=2 SV=1	ZNF296	TLSSFSN[+1]LK	TLSSFSNLK	2	499.2637
sp|Q92820|GGH_HUMAN	Gamma-glutamyl hydrolase OS=Homo sapiens GN=GGH PE=1 SV=2	GGH	SINGILFPGGSVDLR	SINGILFPGGSVDLR	2	772.9252
sp|Q92820|GGH_HUMAN	Gamma-glutamyl hydrolase OS=Homo sapiens GN=GGH PE=1 SV=2	GGH	FFNVLTTNTDGK	FFNVLTTNTDGK	2	678.8433
sp|Q92820|GGH_HUMAN	Gamma-glutamyl hydrolase OS=Homo sapiens GN=GGH PE=1 SV=2	GGH	NLDGISHAPNAVK	NLDGISHAPNAVK	2	668.3544
sp|Q92954|PRG4_HUMAN	Proteoglycan 4 OS=Homo sapiens GN=PRG4 PE=1 SV=2	PRG4	C[+57]FESFER	CFESFER	2	487.7055
sp|Q96PD5|PGRP2_HUMAN	N-acetylmuramoyl-L-alanine amidase OS=Homo sapiens GN=PGLYRP2 PE=1 SV=1	PGLYRP2	GC[+57]PDVQASLPDAK	GCPDVQASLPDAK	2	679.3245
sp|Q96PD5|PGRP2_HUMAN	N-acetylmuramoyl-L-alanine amidase OS=Homo sapiens GN=PGLYRP2 PE=1 SV=1	PGLYRP2	TFTLLDPK	TFTLLDPK	2	467.7658
sp|Q96PD5|PGRP2_HUMAN	N-acetylmuramoyl-L-alanine amidase OS=Homo sapiens GN=PGLYRP2 PE=1 SV=1	PGLYRP2	DTLPSC[+57]AVR	DTLPSCAVR	2	509.7529
sp|Q9BQI4|CCDC3_HUMAN	Coiled-coil domain-containing protein 3 OS=Homo sapiens GN=CCDC3 PE=2 SV=1	CCDC3	HLELANQK	HLELANQK	2	476.7642
sp|Q9BVG8|KIFC3_HUMAN	Kinesin-like protein KIFC3 OS=Homo sapiens GN=KIFC3 PE=1 SV=4	KIFC3	GVDC[+57]STGLR	GVDCSTGLR	2	482.7295
sp|Q9C0F0|ASXL3_HUMAN	Putative Polycomb group protein ASXL3 OS=Homo sapiens GN=ASXL3 PE=2 SV=3	ASXL3	IEDDQSTR	IEDDQSTR	2	482.2225
sp|Q9NZP8|C1RL_HUMAN	Complement C1r subcomponent-like protein OS=Homo sapiens GN=C1RL PE=1 SV=2	C1RL	GSEAINAPGDNPAK	GSEAINAPGDNPAK	2	670.8257
sp|Q9UGM5|FETUB_HUMAN	Fetuin-B OS=Homo sapiens GN=FETUB PE=1 SV=2	FETUB	GSVQYLPDLDDK	GSVQYLPDLDDK	2	675.3328
sp|Q9UK55|ZPI_HUMAN	Protein Z-dependent protease inhibitor OS=Homo sapiens GN=SERPINA10 PE=1 SV=1	SERPINA10	ETFFN[+1]LSK	ETFFNLSK	2	493.7451
sp|Q9UK55|ZPI_HUMAN	Protein Z-dependent protease inhibitor OS=Homo sapiens GN=SERPINA10 PE=1 SV=1	SERPINA10	TVIEVDER	TVIEVDER	2	480.7535
sp|Q9ULV4|COR1C_HUMAN	Coronin-1C OS=Homo sapiens GN=CORO1C PE=1 SV=1	CORO1C	NDQC[+57]YDDIR	NDQCYDDIR	2	599.7433
sp|Q9Y2M2|SSUH2_HUMAN	Protein SSUH2 homolog OS=Homo sapiens GN=SSUH2 PE=1 SV=1	SSUH2	DISLASQR	DISLASQR	2	445.2405
sp|Q9Y5Y7|LYVE1_HUMAN	Lymphatic vessel endothelial hyaluronic acid receptor 1 OS=Homo sapiens GN=LYVE1 PE=1 SV=2	LYVE1	DQVETALK	DQVETALK	2	452.2427
