# Supplementary figures and images for: Predictive proteomic signatures for response of pancreatic cancer patients receiving chemotherapy
Source: Clin Proteomics. 2019 Jul 17;16:31. doi: 10.1186/s12014-019-9251-3 (PMC6636003; doi:10.1186/s12014-019-9251-3)

**Figure S2.** Histograms for the mass deviations of the HQ peptides.

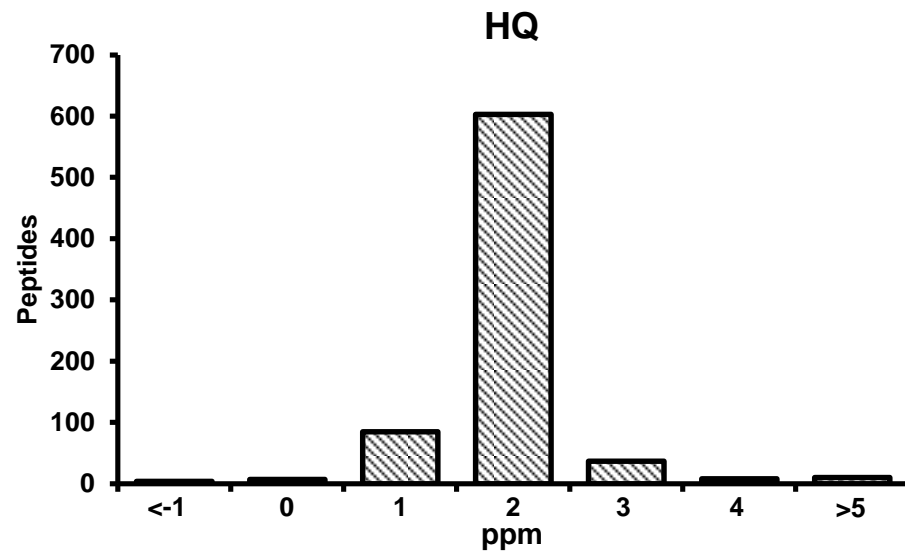

Supplement: Supplementary file 5 — Additional file 5: Figure S2. Histograms for the mass deviations of the HQ peptides. [file 12014_2019_9251_MOESM5_ESM.pdf]
