# Supplementary material for: Predictive proteomic signatures for response of pancreatic cancer patients receiving chemotherapy
Source: Clin Proteomics. 2019 Jul 17;16:31. doi: 10.1186/s12014-019-9251-3 (PMC6636003; doi:10.1186/s12014-019-9251-3)

**Figure S3.** The correlation of the intensities and retention time of the HQ peptides, and the intensities of their resulting proteins for the analytical (A-C) and biological replicates (D-F). AR: analytical replicate; BR: biological replicate.

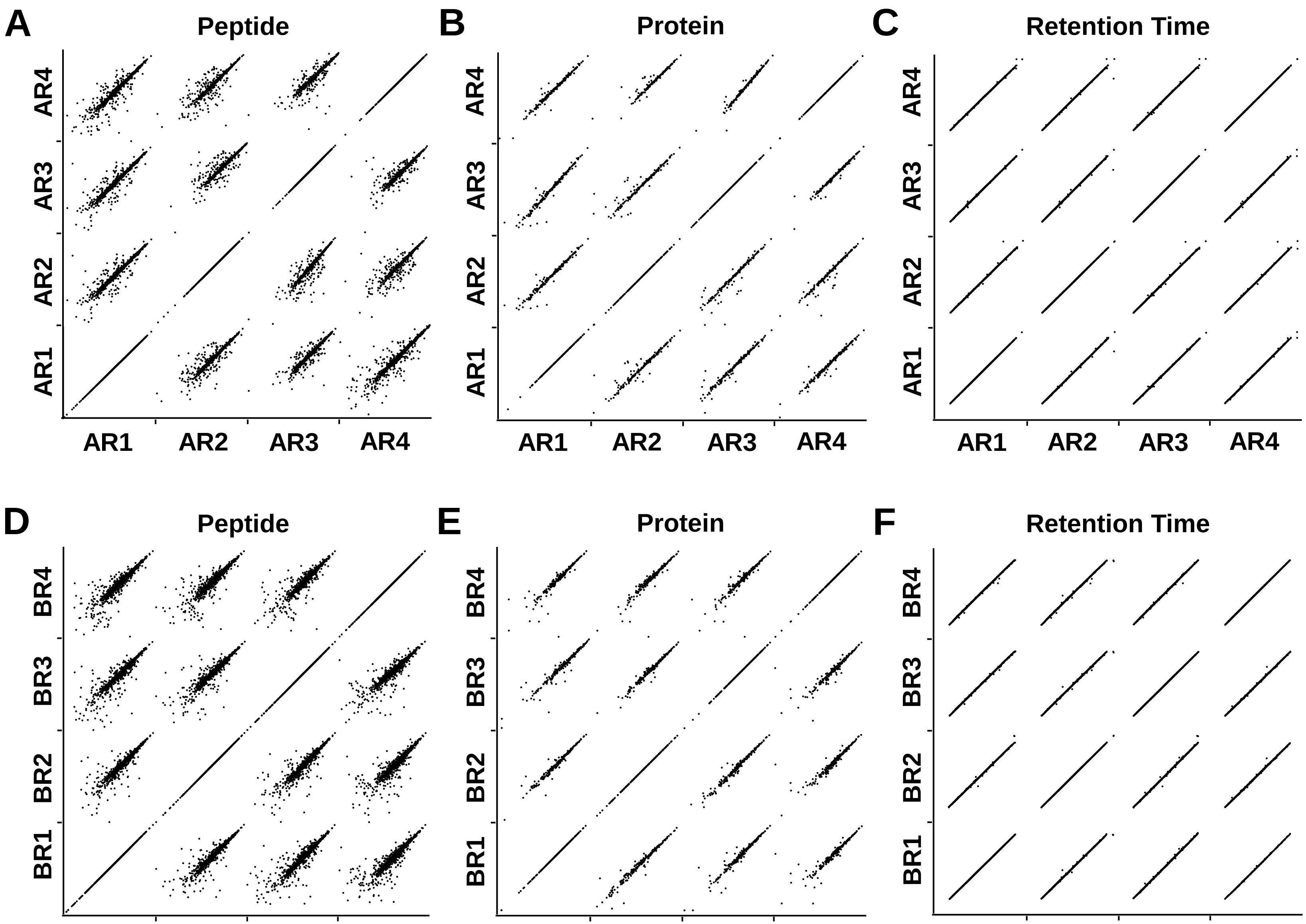

Supplement: Supplementary file 6 — Additional file 6: Figure S3. The correlation of the intensities and retention time of the HQ peptides, and the intensities of their resulting proteins for the analytical (A-C) and biological replicates (D-F). [file 12014_2019_9251_MOESM6_ESM.pdf]
