# Supplementary material for: Predictive proteomic signatures for response of pancreatic cancer patients receiving chemotherapy
Source: Clin Proteomics. 2019 Jul 17;16:31. doi: 10.1186/s12014-019-9251-3 (PMC6636003; doi:10.1186/s12014-019-9251-3)
Supplement: Supplementary file 7 — Additional file 7: Table S4. The BD proteins between Good-responders and Limited-responders. [file 12014_2019_9251_MOESM7_ESM.pdf]

Table S4. The *BD* proteins between PDAC Good-responders and Limited-responders.

UniProtKB	Protein Description	Ratio (GR/LR) (Mean±SD)	P-value	Glycosylation
Q96HR3	Mediator of RNA polymerase II transcription subunit 30	2.94±1.83	0.038	
O75319	RNA/RNP complex-1-interacting phosphatase	2.40±1.50	0.016	Yes
P22891	Vitamin K-dependent protein Z	2.04±0.61	0.024	Yes
P07864	L-lactate dehydrogenase C chain	2.00±1.29	0.027	
Q6ZMR3	L-lactate dehydrogenase A-like 6A	2.00±1.29	0.027	
Q66K66	Transmembrane protein 198	1.94±1.70	0.061	
O94929	Actin-binding LIM protein 3	1.86±0.95	0.096	
Q9H299	SH3 domain-binding glutamic acid-rich-like protein 3	1.72±1.12	0.088	
P04075	Fructose-bisphosphate aldolase A	1.61±0.46	0.019	
P05154	Plasma serine protease inhibitor	1.55±0.47	0.044	Yes
P00739	Haptoglobin-related protein	1.51±0.11	0.014	
P43251	Biotinidase	1.41±0.21	0.037	Yes
P25311	Zinc-alpha-2-glycoprotein	1.41±0.13	0.040	Yes
P02753	Retinol-binding protein 4	1.39±0.13	0.008	
P00338	L-lactate dehydrogenase A chain	1.39±0.49	0.092	
Q5T7N2	LINE-1 type transposase domain-containing protein 1	1.34±0.66	0.050	
P14151	L-selectin	1.33±0.38	0.073	Yes
Q04756	Hepatocyte growth factor activator	1.31±0.26	0.043	Yes
P02749	Beta-2-glycoprotein 1	1.31±0.12	0.057	Yes
P36955	Pigment epithelium-derived factor	1.24±0.07	0.072	Yes
Q14624	Inter-alpha-trypsin inhibitor heavy chain H4	1.21±0.05	0.097	Yes
P00734	Prothrombin	1.17±0.04	0.089	Yes
P02748	Complement component C9	0.86±0.04	0.062	Yes
P07357	Complement component C8 alpha chain	0.81±0.06	0.080	Yes
Q06033	Inter-alpha-trypsin inhibitor heavy chain H3	0.80±0.06	0.083	Yes
P07358	Complement component C8 beta chain	0.79±0.08	0.080	Yes
P00736	Complement C1r subcomponent	0.75±0.09	0.056	Yes
P20851	C4b-binding protein beta chain	0.73±0.07	0.002	Yes
Q12805	EGF-containing fibulin-like extracellular matrix protein 1	0.72±0.20	0.010	Yes
P08571	Monocyte differentiation antigen CD14	0.72±0.23	0.052	Yes
P20718	Granzyme H	0.68±0.20	0.023	Yes
P04278	Sex hormone-binding globulin	0.50±0.21	0.010	Yes
P07195	L-lactate dehydrogenase B chain	0.50±0.65	0.027	
P18428	Lipopolysaccharide-binding protein	0.48±0.39	0.034	Yes
Q5T1H1	Protein eyes shut homolog	0.39±0.30	0.055	Yes
Q8WUU4	Zinc finger protein 296	0.20±0.33	0.043	
P04275	von Willebrand factor	0.17±0.38	0.051	Yes

GR: Good-responder, LR: Limited-responder
