# Supplementary material for: Predictive proteomic signatures for response of pancreatic cancer patients receiving chemotherapy
Source: Clin Proteomics. 2019 Jul 17;16:31. doi: 10.1186/s12014-019-9251-3 (PMC6636003; doi:10.1186/s12014-019-9251-3)
Supplement: Supplementary file 8 — Additional file 8: Table S5. KEGG pathway analysis of the BD proteins. [file 12014_2019_9251_MOESM8_ESM.pdf]

Table S5. The *BD* proteins between PDAC Good-responders and Limited-responders are involved in the coagulation and complement cascades, and glycolysis/gluconeogenesis indicated by the KEGG pathway analysis.

Pathways	Proteins	P-value
complement and coagulation cascades	Prothrombin, Complement component C9, Complement component C8 alpha chain, Complement component C8 beta chain, Complement C1r subcomponent, C4b-binding protein beta chain, Plasma serine protease inhibitor, von Willebrand factor	3.32E-10
glycolysis/gluconeogenesis	Fructose-bisphosphate aldolase A, L-lactate dehydrogenase C chain, L-lactate dehydrogenase A-like 6A, L-lactate dehydrogenase A chain, L-lactate dehydrogenase B chain	2.80E-05
