# Supplementary material for: Predictive proteomic signatures for response of pancreatic cancer patients receiving chemotherapy
Source: Clin Proteomics. 2019 Jul 17;16:31. doi: 10.1186/s12014-019-9251-3 (PMC6636003; doi:10.1186/s12014-019-9251-3)
Supplement: Supplementary file 9 — Additional file 9: Table S6. The TID proteins between Good-responders and Limited-responders. [file 12014_2019_9251_MOESM9_ESM.pdf]

Table S6. The *TID* proteins between PDAC Good-responders and Limited-responders. The abundance ratios of the proteins were calculated by dividing the normalized intensities after the chemotherapy by the baseline intensity.

UniProtKB	Protein	Ratio (Mean±SD)		P-value	Glycosylation
		GR	LR		
Q9UGM5	Fetuin-B	1.28±0.37	0.77±0.23	0.005	Yes
P04180	Phosphatidylcholine-sterol acyltransferase	1.48±0.48	0.80±0.48	0.013	Yes
P00739	Haptoglobin-related protein	0.71±0.27	1.47±0.66	0.014	
P27169	Serum paraoxonase/arylesterase 1	1.43±0.49	0.84±0.36	0.015	Yes
P05452	Tetranectin	1.21±0.35	0.85±0.14	0.016	Yes
P06276	Cholinesterase	1.21±0.40	0.80±0.21	0.020	Yes
P00734	Prothrombin	0.87±0.10	1.07±0.20	0.024	Yes
Q9H079	KATNB1-like protein 1	1.98±1.00	0.90±0.67	0.024	
O14791	Apolipoprotein L1	1.51±0.68	0.86±0.31	0.026	Yes
P07357	Complement component C8 alpha chain	1.23±0.33	0.89±0.20	0.028	Yes
P00748	Coagulation factor XII	1.61±0.82	0.86±0.30	0.038	Yes
P20718	Granzyme H	1.70±1.11	0.80±0.49	0.053	Yes
P06396	Gelsolin	1.21±0.34	0.89±0.26	0.055	
Q9UK55	Protein Z-dependent protease inhibitor	1.43±0.70	0.83±0.40	0.057	Yes
P07195	L-lactate dehydrogenase B chain	1.02±0.50	2.91±2.48	0.069	
P07864	L-lactate dehydrogenase C chain	1.01±0.51	2.85±2.54	0.082	
Q6ZMR3	L-lactate dehydrogenase A-like 6A	1.01±0.51	2.85±2.54	0.082	
P07358	Complement component C8 beta chain	1.19±0.35	0.89±0.31	0.086	Yes
P02749	Beta-2-glycoprotein 1	1.02±0.36	1.40±0.47	0.090	Yes
P68871	Hemoglobin subunit beta	0.78±0.53	1.25±0.51	0.091	Yes
P04075	Fructose-bisphosphate aldolase A	0.83±0.50	1.57±1.05	0.093	
P03952	Plasma kallikrein	1.07±0.21	0.86±0.24	0.097	Yes

GR: Good-responder, LR: Limited-responder
