# Supplementary material for: Predictive proteomic signatures for response of pancreatic cancer patients receiving chemotherapy
Source: Clin Proteomics. 2019 Jul 17;16:31. doi: 10.1186/s12014-019-9251-3 (PMC6636003; doi:10.1186/s12014-019-9251-3)
Supplement: Supplementary file 11 — Additional file 11: Table S8. The BD N-linked glycopeptides. [file 12014_2019_9251_MOESM11_ESM.pdf]

Table S8. The BD glycopeptides between PDAC Good-responders and Limited-responders.

UniProtKB	Protein Description	Glycopeptides	Ratio (GR/LR) (Mean ± SD)	P-value
P43251	Biotinidase	NPVGLIGAEN[+1]ATGETDPSHSK	1.50±0.75	0.01
P05155	Plasma protease C1 inhibitor	DTFVN[+1]ASR	0.82±0.04	0.07
P00450	Ceruloplasmin	ELHHLQEQN[+1]VSNAFLDK	0.81±0.07	0.01
P0C0L4	Complement C4-A	GLN[+1]VTLSSTGR	0.78±0.09	0.06
P00450	Ceruloplasmin	EHEGAIYPDN[+1]TTDFQR	0.81±0.06	0.06
P00739	Haptoglobin-related protein	NLFLN[+1]HSEN[+1]ATAK	0.41±0.06	0.03

GR: Good-responder, LR: Limited-responder
