# Supplementary material for: Predictive proteomic signatures for response of pancreatic cancer patients receiving chemotherapy
Source: Clin Proteomics. 2019 Jul 17;16:31. doi: 10.1186/s12014-019-9251-3 (PMC6636003; doi:10.1186/s12014-019-9251-3)
Supplement: Supplementary file 12 — Additional file 12: Table S9. The TIC N-linked glycopeptides. [file 12014_2019_9251_MOESM12_ESM.pdf]

Table S9. The *TID* glycopeptides between PDAC Good-responders and Limited-responders after chemotherapy.

UniProtKB	Protein Description	Glycopeptides	Ratio (after/before) (Mean ± SD)		P-value
			GR	LR	
P04114	Apolipoprotein B-100	YDFN[+1]SSMLYSTAK	2.08±1.08	0.74±0.35	0.01
P06276	Cholinesterase	EN[+1]ETEIIK	1.28±0.51	0.76±0.17	0.02
P80108	Phosphatidylinositol-glycan-specific phospholipase D	LGTSLSSGHVLMN[+1]GTLK	6.30±6.82	1.23±0.78	0.06
P03952	Plasma kallikrein	GVNFN[+1]VSK	1.37±0.41	0.81±0.23	0.01
Q13201	Multimerin-1	FNPGAESVVLSN[+1]STLK	0.74±0.25	1.37±0.63	0.02
Q9UK55	Protein Z-dependent protease inhibitor	ETFFN[+1]LSK	1.24±0.28	0.78±0.30	0.01
P05546	Heparin cofactor 2	N[+1]LSMPLLPADFHK	1.24±0.42	0.71±0.29	0.01

R: Good-responder, LR: Limited-responder
