# Supplementary material for: Predictive proteomic signatures for response of pancreatic cancer patients receiving chemotherapy
Source: Clin Proteomics. 2019 Jul 17;16:31. doi: 10.1186/s12014-019-9251-3 (PMC6636003; doi:10.1186/s12014-019-9251-3)

Figure S4. PCA analysis of differential N-linked glycopeptides. (A) BD glycopeptides, (B) TIC glycopeptides. GR: Good-responders; LR: Limited-responders;

A. BD glycopeptides

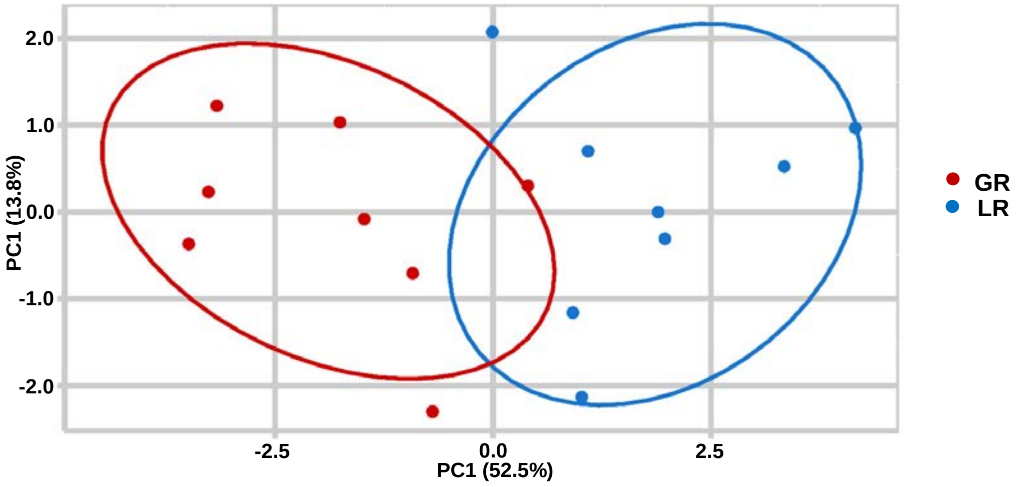

B. TIC glycopeptides

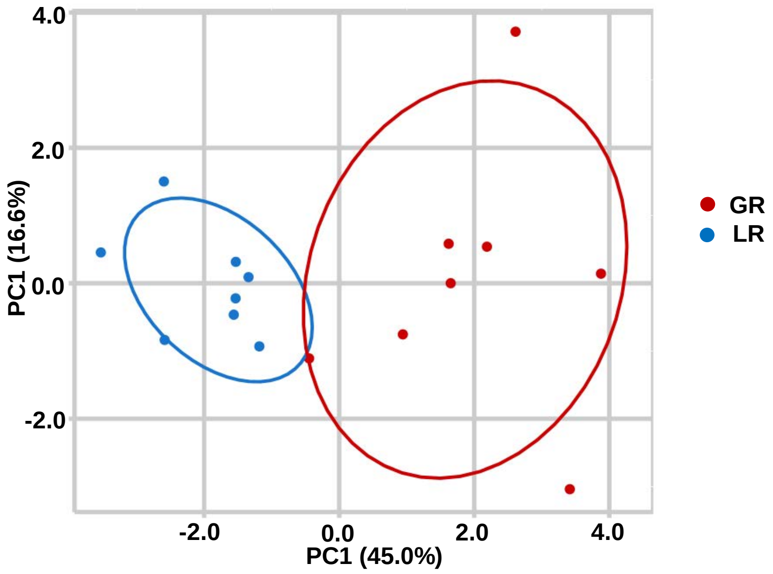

Supplement: Supplementary file 13 — Additional file 13: Figure S4. PCA analysis of glycopeptides. [file 12014_2019_9251_MOESM13_ESM.pdf]
