# Supplementary material for: Predictive proteomic signatures for response of pancreatic cancer patients receiving chemotherapy
Source: Clin Proteomics. 2019 Jul 17;16:31. doi: 10.1186/s12014-019-9251-3 (PMC6636003; doi:10.1186/s12014-019-9251-3)
Supplement: Supplementary file 14 — Additional file 14: Table S10. The variant peptides with significant difference (p < 0.05) between Good-responders and Limited-responders. [file 12014_2019_9251_MOESM14_ESM.pdf]

Table S10. The variant peptides with significant difference ($p<0.05$) between Good-responders and Limited-responders.

Variant Peptide	Uniprot ID	Protein Name	Position	Substitution	Spectral Counts GR (Mean±SD)	Spectral Counts LR (Mean±SD)	Fold change (GR/LR)	P-value
R.CNPGTGECVCPAGWVGEQCQHCGGR.F	O75882	Attractin	7	Q→E	0.63±0.74	0±0	NA	0.032
K.AEEEHLGILGPKLHADVGDK.V	P00450	Ceruloplasmin	12	Q→K	9.63±1.85	12.50±2.07	0.77	0.011
K.AEEQHLGILGPQLHADVGDK.V	P00450	Ceruloplasmin	4	E→Q	12.38±2.50	15.75±2.66	0.79	0.020
R.KAEEEHLGILGPKLHADVGDK.V	P00450	Ceruloplasmin	13	Q→K	28.63±5.97	35.25±4.17	0.81	0.022
R.KAEEQHLGILGPQLHADVGDK.V	P00450	Ceruloplasmin	5	E→Q	10.13±2.30	13.25±2.92	0.76	0.032
R.SGAGTENSACIPWAYYSTVDQVK.D	P00450	Ceruloplasmin	7	D→N	4.00±1.20	6.38±2.50	0.63	0.030
R.SGAGTQDSACIPWAYYSTVDQVK.D	P00450	Ceruloplasmin	6	E→Q	3.75±1.28	6.00±1.93	0.63	0.016
R.AYPLSIEPIGVR.F	P00450	Ceruloplasmin	0	G→R	0.88±0.83	1.88±0.83	0.47	0.031
R.RNTANLFPQTSLTLHMWPDTEGTFNVECLTTDHYTGGMK.Q	P00450	Ceruloplasmin	2	D→N	0.25±0.71	1.25±0.89	0.20	0.026
R.KFCSLDNGDCDQFCHEEQNSVVCSCAR.G	P00742	Coagulation factor X	2	L→F	4.38±1.06	2.75±0.89	1.59	0.005
R.NTEEEEGGEAVHEVEVVIK.H	P00742	Coagulation factor X	4	Q→E	1.38±0.74	0.63±0.52	2.19	0.035
K.CSGTEASVVAPPPVVLLPDVETPSKEDCMFGNGK.G	P00747	Plasminogen	25	E→K	1.75±1.28	0.50±0.76	3.50	0.032
R.TECFITGWGETEGTFGAGLLK.E	P00747	Plasminogen	12	Q→E	9.63±5.26	5.25±2.05	1.83	0.046
R.VQSTELCAGHLAGGTDSCQGESGGPLVCFEK.D	P00747	Plasminogen	21	D→E	0.63±0.52	0.13±0.35	4.85	0.041
K.CEENEEFTCR.A	P00747	Plasminogen	4	D→N	1.63±1.06	2.88±0.35	0.57	0.007
K.TMSGLECQAWNSQSPHAHGYIPSK.F	P00747	Plasminogen	11	D→N	0.38±0.52	1.13±0.83	0.34	0.049
R.SEFPESWLWNVEDLKEPPKNGNSTK.L	P01024	Complement C3	22	I→N	0.75±1.04	2.63±0.74	0.29	0.001
K.CCYNGACVNNDETCEQR.A	P01031	Complement C5	4	D→N	5.63±0.74	3.75±0.71	1.50	0.000
K.ELSCYSLEDLNNK.Y	P01031	Complement C5	4	Y→C	2.13±1.13	1.00±0.76	2.13	0.034
R.CNGDNDCGDFSDEDNCESEPRPPCR.D	P02748	Complement component C9	15	D→N	2.13±1.55	3.38±0.52	0.63	0.049
K.CSCTEDAQCIDGTIEVPK.C	P02749	Beta-2-glycoprotein	3	Y→C	8.88±2.23	6.75±1.39	1.32	0.038
K.GNDDHWIVDTDCDTYAVQYSCR.L	P02753	Retinol-binding protein 4	12	Y→C	7.13±2.53	3.63±1.85	1.96	0.007
K.GNDDHWIVDTDYNTYAVQYSCR.L	P02753	Retinol-binding protein 4	13	D→N	16.38±5.32	10.00±4.41	1.64	0.021
K.GNDDHWIVDTNYDTYAVQYSCR.L	P02753	Retinol-binding protein 4	11	D→N	16.00±5.48	9.63±4.44	1.66	0.023
K.GNDDHWIVNTDYDTYAVQYSCR.L	P02753	Retinol-binding protein 4	9	D→N	14.00±5.15	8.75±4.37	1.60	0.045
K.ALGISPFHKHAEVVFTANDSGPR.R	P02766	Transthyretin	9	E→K	15.13±4.97	7.88±5.17	1.92	0.013
K.ALGISPFHQHAEVVFTANDSGPR.R	P02766	Transthyretin	9	E→Q	94.00±22.77	44.25±22.85	2.12	0.001
K.ALGNSPFHEHAEVVFTANDSGPR.R	P02766	Transthyretin	4	I→N	51.88±9.76	34.13±15.97	1.52	0.018
R.RYTIAALLSPCSYSTTAVVTNPK.E	P02766	Transthyretin	11	Y→C	7.13±2.30	4.00±1.60	1.78	0.007
R.RYTIAALLSPYSCSTTAVVTNPK.E	P02766	Transthyretin	13	Y→C	7.13±2.30	4.00±1.60	1.78	0.007
R.YTIAALLSPCSYSTTAVVTNPKE.-	P02766	Transthyretin	10	Y→C	4.00±0.93	2.63±1.19	1.52	0.022
R.YTIAALLSPYSCSTTAVVTNPKE.-	P02766	Transthyretin	12	Y→C	3.88±0.99	2.63±1.19	1.48	0.038
K.HQPQEFPTYVEPTNNEICEAFR.K	P02774	Vitamin D-binding protein	15	D→N	12.13±1.89	9.88±1.89	1.23	0.032
K.SYLSVVGSCCTSASPTVCFLK.E	P02774	Vitamin D-binding protein	5	M→V	0.50±0.53	0±0	NA	0.019
R.DGWHSWPIAHQWPQGPSAVDAAFSWEQK.L	P02790	Hemopexin	27	E→Q	1.13±0.99	3.63±2.56	0.31	0.022
K.LSCSYSHWSAPAPECK.A	P04003	C4b-binding protein alpha chain	14	Q→E	0.25±0.46	0.75±0.46	0.33	0.049
K.CQCDKLCSYYQSCCTDYTAECKPQVTR.G	P04004	Vitronectin	5	E→K	8.25±0.89	6.25±1.28	1.32	0.003
K.IQSPLFTLDANADIGNGTTSANEAGNAASITAK.G	P04114	Apolipoprotein B-100	26	I→N	2.13±1.13	4.50±2.83	0.47	0.045
K.NFVASHIANILNSEELDIQNLKK.L	P04114	Apolipoprotein B-100	20	D→N	1.13±0.64	0.38±0.74	2.97	0.049
K.TQFNNNEYSQNLDAYNTK.D	P04114	Apolipoprotein B-100	11	D→N	4.38±0.52	2.88±0.64	1.52	0.000
K.YGMIAQVTQTLK.L	P04114	Apolipoprotein B-100	4	V→I	0.88±1.13	0±0	NA	0.045
K.KGISTSAASPAVGTVGMDMDKDDDFSK.W	P04114	Apolipoprotein B-100	21	E→K	0.00±0.00	0.50±0.53	0	0.019
K.HPLKPDIQPFPQSVSESCPGK.F	P04196	Histidine-rich glycoprotein	7	N→I	12.50±3.07	9.13±2.36	1.37	0.027
K.QAAGSGHLLALGTPQNPSWLSLHLQDQK.V	P04278	Sex hormone-binding globulin	15	E→Q	0.13±0.35	1.00±0.93	0.13	0.026
K.ENTVTNNWIPEGEEDDDYLDLEK.I	P05546	Heparin cofactor 2	7	D→N	0.50±0.76	1.63±1.19	0.31	0.040
K.YGFCEAAEQFHVLDEVR.R	P07360	Complement component C8 gamma chain	8	D→E	0.75±0.71	0.13±0.35	5.77	0.042
R.WPAGLTSSQVDLYIPK.V	P08185	Corticosteroid-binding globulin	2	S→P	1.75±1.28	0.63±0.74	2.78	0.050
R.NVVQITCLDGFEVVEGR.V	P09871	Complement C1s subcomponent	1	D→N	2.75±1.04	6.50±4.28	0.42	0.030
R.GGGAGFISGLTYLELDNPAGNK.R	P10643	Complement component C7	11	S→T	2.25±1.83	4.63±2.26	0.49	0.037
K.IACVLPVLMNGIQSHPQKPFYTVGEK.V	P10643	Complement component C7	10	D→N	1.88±0.99	3.50±1.77	0.54	0.040
R.CFPLSLVPTEFCPSPPALK.D	P10643	Complement component C7	0	H→R	2.00±1.77	3.75±1.39	0.53	0.045
K.IACVLPVLKDGIQSHPQKPFYTVGEK.V	P10643	Complement component C7	9	M→K	0.63±0.52	0±0	NA	0.004
R.LTYTNWNEGKPNNAGSDEDCVLLLK.N	P11226	Mannose-binding protein C	10	E→K	0.63±0.74	1.63±0.92	0.39	0.031
R.LTYTNWNKGEPNNAGSDEDCVLLLK.N	P11226	Mannose-binding protein C	8	E→K	0.63±0.74	1.63±0.92	0.39	0.031
R.KLECNGENDCGNNSDER.D	P13671	Complement component C6	12	D→N	3.13±0.64	4.50±1.20	0.70	0.012
K.YNPIPSVQLMGDGFHFLAGEPR.G	P13671	Complement component C6	12	N→D	0.50±0.93	1.75±1.28	0.29	0.042
R.CSLTYIYTGLSK.H	P25311	Zinc-alpha-2-glycoprotein	1	Y→C	2.75±1.28	1.38±1.06	1.99	0.035
K.NPVGLIGAENATGETNPSHSK.F	P43251	Biotinidase	16	D→N	2.75±0.89	1.75±0.71	1.57	0.026
R.DIENFDSTQK.F	P43652	Afamin	6	N→D	0.63±0.52	0±0	NA	0.004
R.YMAVDVDECK.E	P48740	Mannan-binding lectin serine protease 1	0	H→R	0±0	0.50±0.53	0.00	0.019
K.EGYSDISYIVVNHQGISSR.L	P49908	Selenoprotein	5	N→D	3.38±0.52	2.50±0.93	1.35	0.035
R.QLGLLGPPDVPDHAAYHPFR.R	Q14624	Inter-alpha-trypsin inhibitor heavy chain H4	5	P→L	8.50±3.42	12.50±3.78	0.68	0.044
K.FGQGAHHAAGKAGNEAGR.F	Q6UWP8	Suprabasin	11	Q→K	0.13±0.35	1.50±1.07	0.09	0.004
K.LSQSGKVGEPAGTDPGLDDLDVALSNLEVK.L	Q86UX7	Fermitin family homolog 3	6	E→K	0.75±0.89	0±0	NA	0.031
