# Supplementary material for: Predictive proteomic signatures for response of pancreatic cancer patients receiving chemotherapy
Source: Clin Proteomics. 2019 Jul 17;16:31. doi: 10.1186/s12014-019-9251-3 (PMC6636003; doi:10.1186/s12014-019-9251-3)

Figure S5. PCA analysis of significant variant peptides. PCA showed that PDAC Good-responders and Limited-responders were segregated based on the spectral counts of the variant peptides.

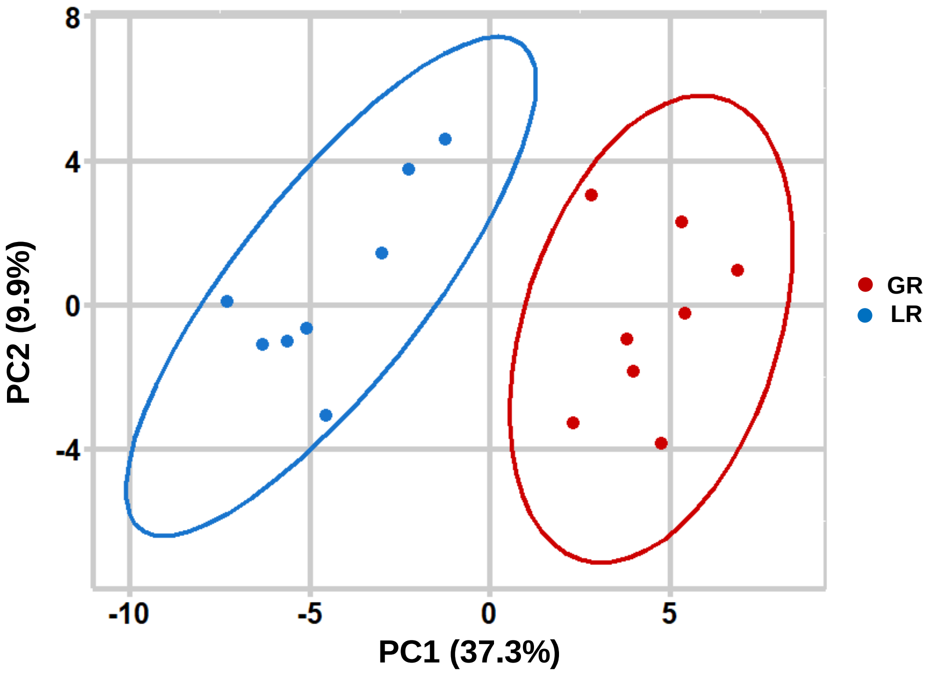

Supplement: Supplementary file 15 — Additional file 15: Figure S5. PCA analysis of significant variant peptides. [file 12014_2019_9251_MOESM15_ESM.pdf]
