# Supplementary material for: Predictive proteomic signatures for response of pancreatic cancer patients receiving chemotherapy
Source: Clin Proteomics. 2019 Jul 17;16:31. doi: 10.1186/s12014-019-9251-3 (PMC6636003; doi:10.1186/s12014-019-9251-3)

Figure S6 Four plasma proteins PZ, AZGP1, SHBG, and VWF (orange dots) showed statistically significance in the volcano plot.

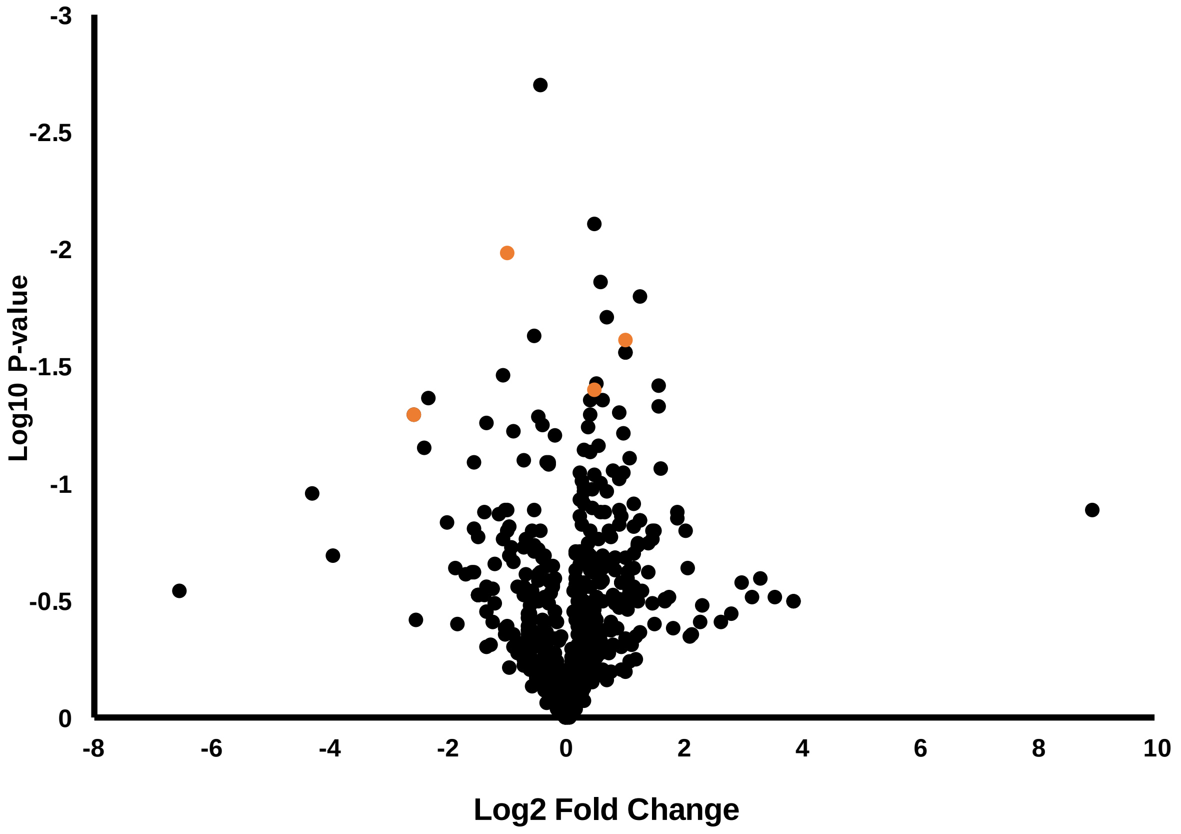

Supplement: Supplementary file 16 — Additional file 16: Figure S6. Four plasma proteins PZ, AZGP1, SHBG, and VWF showed statistically significance in the volcano plot. [file 12014_2019_9251_MOESM16_ESM.pdf]
