# Supplementary material for: Predictive proteomic signatures for response of pancreatic cancer patients receiving chemotherapy
Source: Clin Proteomics. 2019 Jul 17;16:31. doi: 10.1186/s12014-019-9251-3 (PMC6636003; doi:10.1186/s12014-019-9251-3)
Supplement: Supplementary file 17 — Additional file 17: Table S11. The cut points of candidate biomarkers for stratifying PDAC patients. [file 12014_2019_9251_MOESM17_ESM.pdf]

Table S11. The cut points of candidate biomarkers in the best-performing biomarkers to stratify PDAC patients into potential Good-responders and Limited-responders. If a composite biomarker meets the cut point criteria, i.e. “Biomarker-positive”, it suggests a potential Limited-responder, and vice versa.

Stages	Cut points for Biomarker-positive patients
III&IV	PZ < 0.6 µg/ml, SHBG > 4.5 µg/ml, VWF > 12 ng/ml, CA19-9 > 6100 U/ml
III	PZ < 1.8 µg/ml, SHBG > 4.5 µg/ml, VWF > 12 ng/ml, CA19-9 > 800 U/ml
IV	PZ < 0.6 µg/ml, SHBG > 1.9 µg/ml, VWF > 11 ng/ml, CA19-9 > 6100 U/ml
