# Supplementary material for: Predictive proteomic signatures for response of pancreatic cancer patients receiving chemotherapy
Source: Clin Proteomics. 2019 Jul 17;16:31. doi: 10.1186/s12014-019-9251-3 (PMC6636003; doi:10.1186/s12014-019-9251-3)

Figure S7. Relative abundances of PZ, SHBG and VWF between Good-responders and Limited-responders at baseline by proteomics and ELISA. The proteomics study cohort included 16 stage IV PDAC patients, and the validation study cohort included19 stage III and 33 stage IV PDAC patients.

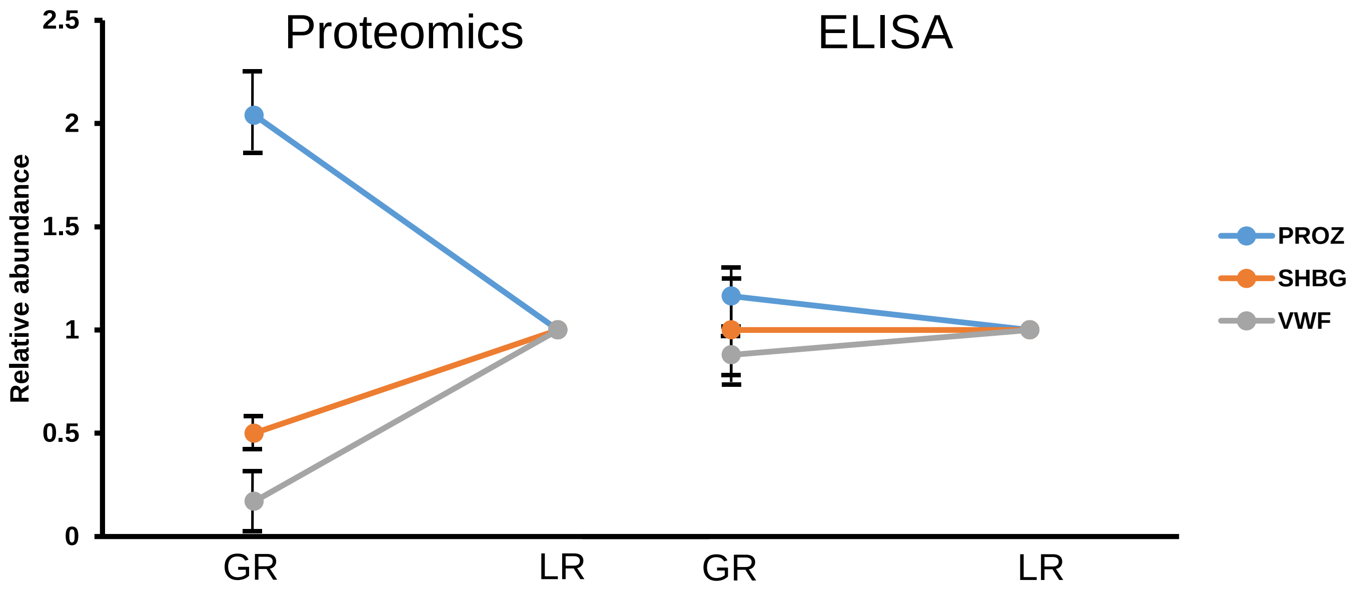

Supplement: Supplementary file 18 — Additional file 18: Figure S7. Concentration ratios of PZ, SHBG and VWF between Good-responders and Limited-responders at baseline by proteomics and ELISA. [file 12014_2019_9251_MOESM18_ESM.pdf]
